# The Natural Product Esculetin as an Efficient Photoacid for the Visible‐Light‐Mediated Acetalization/Ketalization Reaction

**DOI:** 10.1002/cssc.71042

**Published:** 2026-09-26

**Authors:** Maria A. Theodoropoulou, E. Alexandros Routsi, Christiana Mantzourani, Olga G. Mountanea, Demeter Tzeli, Christoforos G. Kokotos, George Kokotos

**Affiliations:** ^1^ Laboratory of Organic Chemistry, Department of Chemistry National and Kapodistrian University of Athens Athens Greece; ^2^ Laboratory of Physical Chemistry, Department of Chemistry National and Kapodistrian University of Athens Athens Greece; ^3^ Theoretical and Physical Chemistry Institute, National Hellenic Research Foundation Athens Greece

**Keywords:** acetalization, esculetin, ketals, photoacid, visible light photocatalysis

## Abstract

The development of photochemical protocols for the production of fine chemicals and industrially interesting products, employing sustainable catalysts under mild conditions, is of high interest. We demonstrate herein that esculetin, a naturally occurring coumarin widespread in plants, is an efficient photoacid, catalyzing acetalization/ketalization reactions under visible light irradiation. A variety of aldehydes and alcohols produce acetals in high yields, using a low catalyst loading of esculetin under 427‐nm LED irradiation. In addition, various ketals, including industrially interesting products, such as solketal, fructone, and levulinate‐based ketals, are generated in high yields. Esculetin has been proven to be an excellent, efficient photocatalyst for the production of acetals and ketals under mild and environmentally friendly conditions, highlighting that natural products offer a platform for investigation in an effort to discover sustainable renewable photocatalysts.

## Graphical abstract

Esculetin: an efficient photoacid catalyzing acetalization/ketalization reactions.

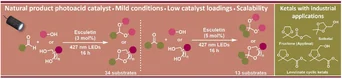

## 1 Introduction

Photochemistry—the use of light to promote chemical transformations—offers significant advantages in modern organic synthesis [1]. It enables access to otherwise challenging or inaccessible reactions, often under mild conditions, which enhances chemoselectivity and functional group tolerance. Moreover, photochemical processes typically align with green chemistry principles by reducing the use of reagents, minimizing byproduct formation, and, in some cases, even utilizing sunlight, a renewable source of energy [2, 3, 4, 5, 6, 7, 8]. Photoacids are weak acids that, upon irradiation, transition to an excited state, in which their acidity is significantly enhanced, allowing them to engage in proton‐transfer reactions [9, 10, 11]. Photoacids can be classified into two categories: photoacid generators (PAGs) and photoacids (PAHs). Upon irradiation, PAGs release protons, forming strong acids through an irreversible process, accompanied by the decomposition of the original molecules. In contrast, PAHs can return to their initial state once irradiation ceases, by regaining the released proton [11]. Brønsted photoacids, including hydroxynaphthalenes and pyranine derivatives, undergo reversible excited‐state proton transfer, enabling an acidity increase under light irradiation. These unique properties of photoacids have found diverse applications, including applications in catalysis, proton‐conducting materials, nanotechnology, and CO_2_ capture [12]. Furthermore, the redox benzoquinone/hydroquinone couple in conjunction with TiO_2_ has recently been shown to promote the photocatalytic synthesis of solketal, where photoexcited hydroquinone acts as a photoacid [13].

Acetalization/ketalization of aldehydes or ketones is commonly employed as a strategy for protecting carbonyl groups in multistep organic synthesis [14]. However, acetals have also found diverse applications, beyond protection, since they have been utilized as starting materials in various synthetic applications [15], as flavoring and fragrance additives [16], and as antifreeze additives in biodiesel fuels [17]. Ketals, in particular cyclic ketals, have attracted special interest, due to their potential industrial applications. For example, cyclic ketals from glycerol may find use as green solvents and fuel additives, while ethyl acetoacetate ketals as fragrances [18, 19, 20, 21, 22]. Conventionally, acetalization/ketalization reactions are catalyzed by Brønsted or Lewis acids, including strong mineral acids, trifluoromethanesulfonic acid, acidic resins or polymers, or various metal‐based catalysts [23, 24, 25, 26, 27, 28, 29].

**SCHEME 1:**
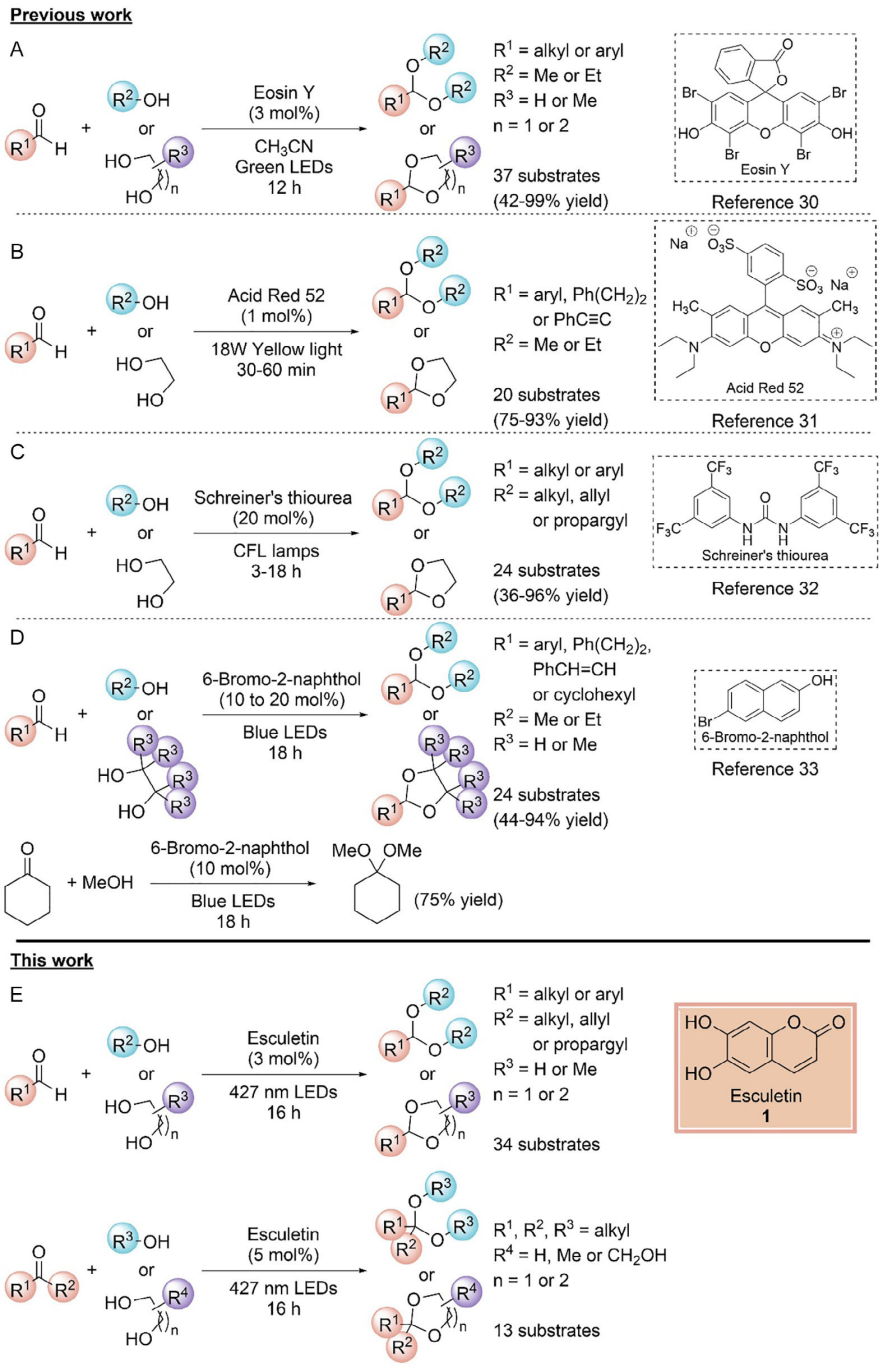
Previous examples on photoacid‐catalyzed acetalization/ketalization of aldehydes and ketones and this work.

Recently, photochemical methods have emerged employing photoacids as effective catalysts for the acetalization of carbonyl compounds, providing milder reaction conditions (Scheme 1). In 2017, Yi et al. first demonstrated that under green LED irradiation and in alcoholic solutions, Eosin Y can function as a photoacid, promoting the acetalization of carbonyl compounds (Scheme 1A) [30]. Similarly, the organic dye acid red 52 was shown to promote aldehyde acetalization under yellow light irradiation, most likely by acting as a photoacid (Scheme 1B) [31]. In the same year, Kokotos and coworkers proposed that Schreiner’s thiourea can act as a photoacid under irradiation, catalyzing acetalization reactions (Scheme 1C) [32]. More recently, 6‐bromo‐2‐naphthol was identified as a photoacid upon blue LED irradiation, effectively catalyzing the acetalization of carbonyl compounds, including the ketalization of cyclohexanone with methanol (Scheme 1D) [33].

Despite these important advances, the number of organic photoacids that have been successfully employed as catalysts for acetalization reactions remains limited. The reported systems mainly rely on synthetic organic dyes and photoacidic catalysts, while naturally occurring photoacids have received little attention. The identification of sustainable, readily available, metal‐free photoacids with enhanced excited‐state acidity therefore remains highly desirable for expanding the scope of photocatalytic acid‐mediated transformations. Aiming at expanding the toolbox of photoacids for synthetic applications, our interest has been attracted by coumarins, a family of natural products widely distributed in the plant kingdom [34]. Coumarins have been extensively studied for their fluorescence properties and have thus found applications in cell imaging and chemosensors [35]. Among the various natural hydroxycoumarins, esculetin (6,7‐dihydroxycoumarin) has attracted interest for its biological properties [36]. In 2022, Knoor et al. reported that esculetin exhibits a strong photoacid character [37]. They demonstrated that esculetin has a ground‐state p*K*_a1_ of approximately 7.3, which significantly drops to a pK^*^_a1_ of about −1 in the excited state, confirming its photoacid character. We envisioned that such properties make esculetin an ideal candidate as a photoacidic catalyst for synthetic applications. In this work, we present a novel photochemical protocol, utilizing the natural product esculetin as a photoacid to promote the acetalization/ketalization of aldehydes and ketones (Scheme 1E). Extensive experimental studies optimized the reaction conditions, including the irradiation source, while a broad substrate scope was investigated, including the synthesis of industrially interesting ketals. In addition, theoretical calculations on the reaction mechanism are presented.

## 2 Results and Discussion

**FIGURE 1:**
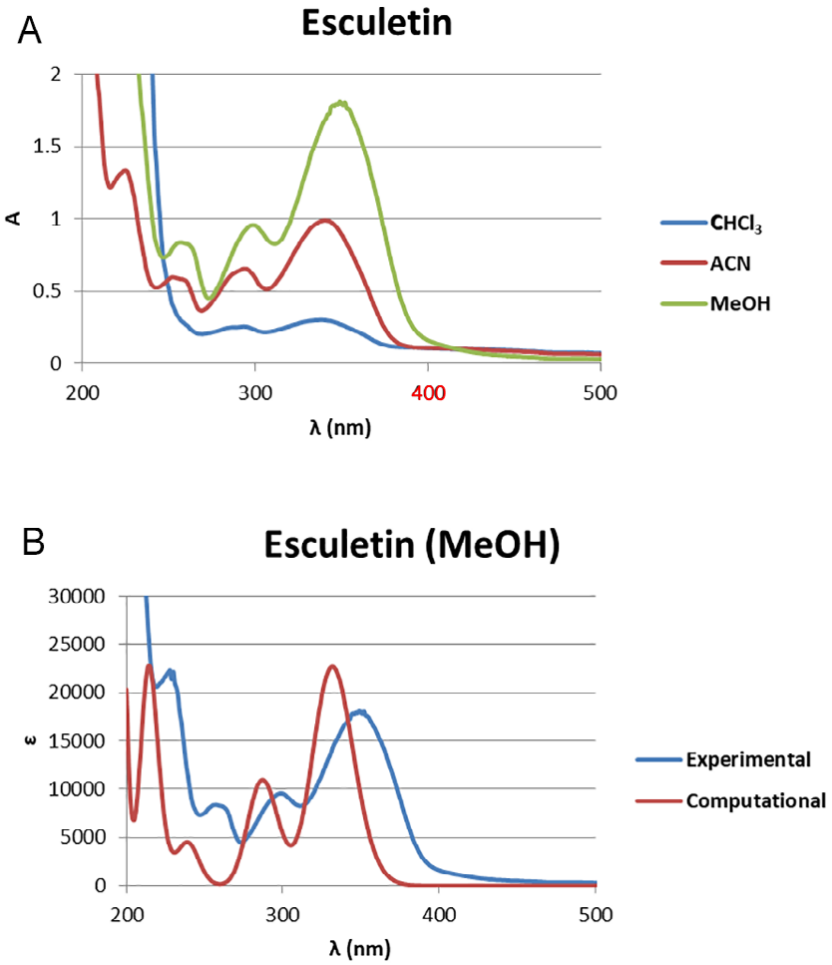
(A) Experimental UV–Vis spectra of esculetin (in CHCl_3_, ACN, and MeOH, *c* = 1.0 × 10^−4^ M) and (B) its comparison with the corresponding calculated (TD‐B3LYP/6‐31G(d,p) methodology) UV–Vis spectrum in MeOH (SMD solvation model).

The UV–Vis absorption spectra of esculetin were recorded in MeOH, ACN, and CHCl_3_ at a concentration of 1.0 × 10^−4^ M (Figure 1). The experimental data reveal absorption features in the 230–400 nm region across all solvents. A dominant broad band between 300 and 400 nm is observed, with *λ*_max_ values at 355 nm in MeOH, 345 nm in ACN, and 341 nm in CHCl_3_ (Figure 1A). Three higher‐energy bands are also evident in MeOH (303, 263, and 230 nm) and ACN solvents (300, 261, and 228 nm), whereas these transitions are not clearly distinguished in the CHCl_3_ solvent at the same concentration, likely due to differences in solvent polarity affecting spectral intensity rather than peak position. Only minor solvatochromic shifts were detected, although the absorption intensity of esculetin in CHCl_3_ was substantially reduced compared to that of MeOH and ACN as the solvent. Conversely, esculetin exhibited the strongest absorption in MeOH. To rationalize these experimental findings, time‐dependent DFT [38] calculations were performed at the TD‐B3LYP [39]/6‐31G(d,p) [40] level using the SMD solvation model [41] in MeOH. As shown in Figure 1B, the theoretical spectrum closely matches the experimental results, supporting the fact that this computational approach may be suitable for elucidating and evaluating the steps of photochemical transformations, in which esculetin functions as the photoacid catalyst.

We began our investigation by focusing on the acetalization reaction between 3‐phenylpropanal (**2a**) and methanol (**3a**). A detailed description of the optimization studies—including catalyst loading, solvent quantities, reaction time, and alcohol equivalents—is provided in the Supporting Information [42]. Optimization studies commenced with the determination of the optimal irradiation wavelength (Table 1). A series of Kessil LED lamps emitting at different wavelengths was tested, and 427 nm was identified as optimal for this transformation (Table 1, entry 4). Reactions were conducted under argon to prevent aerobic oxidation of the aldehyde [43]. Notably, when the reaction was performed under ambient air (the tube was left open), ^1^H‐NMR analysis revealed the formation of both carboxylic acid and hemiacetal as byproducts (Table 1, entry 5). A loading of 3 mol% of esculetin (**1**) afforded the highest conversion (97%), and **4a** was isolated in 91% yield (Table 1, entry 6). Further reducing the catalyst loading to 1 mol% resulted in a decrease in the conversion and isolated yield (Table 1, entry 7). Therefore, 3 mol% was selected as the optimal catalyst loading. The reaction progress over time was then studied, revealing that 16 h was indeed required to achieve maximum conversion (Tables S3 and S10) [42].

**TABLE 1:** Optimization studies for the determination of the optimal wavelength for the irradiation of the acetalization of 3‐phenylpropanal (**2a**) with methanol (**3a**) in the presence of esculetin (**1**).^a^

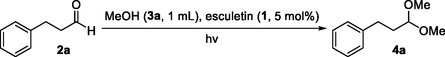			
Entry	LED lamp, *λ*, nm	Conversion (%) of **2a** to **4a** b	Variation from standard conditions
1^c^	—	0	Under dark
2	370	12	
3	390	50 (20)	
4	427	90 (82)	
5^d^	427	37	Open air
**6** **e**	**427**	**97 (91)**	**3 mol% esculetin**
7^f^	427	92 (80)	1 mol% esculetin
8^g^	427	38	6 h
9	440	85 (50)	
10	456	16	
11	467	6	

*Note:* Bold represents the optimal reaction conditions.

a A solution of 3‐phenylpropanal (**2a**, 67 mg, 0.50 mmol) and esculetin (**1**, 4.5 mg, 5 mol%, 0.025 mmol) in MeOH (**3a**, 1 mL) was irradiated for 16 h by a Kessil lamp, under Ar.

b (%) Conversion determined by ^1^H‐NMR; in parentheses the yield of **4a** after isolation by column chromatography.

c The reaction was carried out in the dark.

d The reaction was carried out in the open air, and conversion to carboxylic acid (18%) and hemiacetal (20%) was also observed, while around 25% of the aldehyde remained unreacted; MeOH was partially evaporated.

e 3 mol% of esculetin (**1**, 2.7 mg, 0.015 mmol) was used instead of 5 mol%.

f 1 mol% of esculetin (**1**, 0.9 mg, 0.005 mmol) was used instead of 5 mol%.

g Reaction time: 6 h.

With the optimal reaction conditions in a methanolic solution established, we explored whether the reaction could also be performed in alternative solvents, while using reduced equivalents of methanol (Table 2). This would be particularly advantageous, when the alcohol is a solid or highly viscous reagent and from a green chemistry perspective (lowering the equivalents). Among the solvents tested, only acetonitrile (Table 2, entry 1) or chloroform (Table 2, entry 7) provided satisfactory yields. Given that acetonitrile is an overall greener and less toxic solvent, compared to chloroform, it was selected for subsequent studies. We then examined whether the equivalents of methanol utilized, when employing acetonitrile as the solvent, could be further reduced (Table S7) [42]. However, when less than 10 equivalents of methanol were used (Table 2, entry 2), the conversion to **4a** dropped significantly. Catalyst loading was also re‐evaluated under these new conditions (Table S8) [42]. Reducing esculetin loading from 5 mol% (Table 2, entry 1) to 3 mol% (Table 2, entry 3) resulted in only a subtle decrease in conversion (81%) and isolated yield (75%). Thus, 3 mol% was selected as the optimal catalyst loading for esculetin. Finally, we assessed whether the quantity of acetonitrile could also be reduced (Table S9) [42]. Interestingly, using 670 μL of acetonitrile led to an increase in conversion (86%) and yield (78%) of **4a** (Table 2, entry 4). However, further reduction in solvent volume led to diminished conversion (Table 2, entry 6). In an attempt to remove water produced during the reaction, 2 equivalents of anhydrous Na_2_SO_4_ were added (Table 2, entry 5). This led to a decrease in conversion to **4a**, possibly due to the solution becoming turbid, therefore impeding light penetration. Thus, the optimized reaction conditions are as follows: 3‐phenylpropanal (**2a**, 67 mg, 0.50 mmol) and esculetin (**1**, 2.7 mg, 3 mol%, 0.015 mmol) were dissolved either in methanol (**3a**, 1.0 mL) or in a mixture of methanol (**3a**, 202 μL, 5.00 mmol) and acetonitrile (670 μL), and the resulting solution was irradiated with a 40 W Kessil lamp at 427 nm, under argon, for 16 h.

**TABLE 2:** Solvent screening for the acetalization of 3‐phenylpropanal (**2a**) with methanol (**3a**) in the presence of esculetin (**1**).^a^

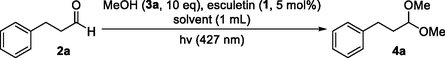			
Entry	Solvents	Conversion (%) of **2a** to **4a** b	Variation from standard conditions
1	CH_3_CN	85 (77)	
2^c^	CH_3_CN	79 (69)	8 eq. MeOH
3^d^	CH_3_CN	81 (75)	3 mol% esculetin
**4** **e**	**CH**_**3**_ **CN**	**86 (78)**	**3 mol% esculetin/0.67 mL CH**_**3**_ **CN**
5^f^	CH_3_CN	48	3 mol% esculetin/0.67 mL CH_3_CN/addition of Na_2_SO_4_ (2 eq.)
6^g^	CH_3_CN	50	3 mol% esculetin/0.50 mL CH_3_CN
7	CHCl_3_	95 (80)	
8	CH_2_Cl_2_	69 (60)	
9	Petroleum ether	28	
10	EtOAc	24	
11	Et_2_O	32	
12	Methyl *tert*‐butyl ether	41 (33)	
13	2‐Me‐THF	13	
14	1,4‐Dioxane	3	

*Note:* Bold represents the optimal reaction conditions.

a A solution of 3‐phenylpropanal (**2a**, 67 mg, 0.50 mmol), MeOH (**3a**, 202 μL, 160 mg, 5.00 mmol), and esculetin (**1**, 4.5 mg, 5 mol%, 0.025 mmol) in various solvents (1 mL) was irradiated for 16 h by a Kessil lamp (427 nm), under Ar.

b (%) Conversion determined by ^1^H‐NMR; in parentheses the yield of **4a** after isolation by column chromatography.

c 8 equivalents of MeOH were used instead of 10 equivalents.

d 3 mol% of esculetin (**1**, 2.7 mg, 0.015 mmol) was used instead of 5 mol%.

e 3 mol% of esculetin (**1**, 2.7 mg, 0.015 mmol) was used instead of 5 mol%, and 0.67 mL of CH_3_CN was used instead of 1.0 mL.

f 3 mol% of esculetin (**1**, 2.7 mg, 0.015 mmol) was used instead of 5 mol%, 0.67 mL of CH_3_CN was used instead of 1.0 mL, and 2 equivalents of anhydrous Na_2_SO_4_ were added to remove water.

g 3 mol% of esculetin (**1**, 2.7 mg, 0.015 mmol) was used instead of 5 mol%, and 0.50 mL of CH_3_CN was used instead of 1.0 mL.

In order to verify the acid‐catalyzed nature of the process, we decided to perform a number of studies. Initially, pH measurements revealed a substantial decrease in the pH of an esculetin solution following irradiation, while an even lower pH value was recorded, when the solution was monitored during irradiation (Table S11) [42]. Next, we performed a reaction, adding NaHCO_3_ (5 mol%), which shut down the process (Table S1, entry 9) [42]. We then performed a light on/off experiment (Table S10 and Figure S1) [42], in which product formation continued during the dark periods, however, at a reduced rate compared to continuous irradiation, which accelerated the reaction. To investigate whether continuous irradiation is required, esculetin was first irradiated in methanol for 4 h, after which the aldehyde was added, and the reaction mixture was maintained in the dark for another 16 h. Under these conditions, 34% conversion to the corresponding acetal was observed. Then, esculetin was first irradiated in methanol for 16 h, after which the aldehyde was added, and the reaction mixture was maintained in the dark for another 16 h. Under these conditions, 71% conversion to the corresponding acetal was observed, indicating that prolonged irradiation generates a persistent acidic species, capable of promoting the reaction without further light exposure. Collectively, these experiments suggest that irradiation produces a catalytically active acidic species that contributes to the observed catalytic activity, whereas continuous light irradiation enhances the overall reaction efficiency. Thus, in addition to excited‐state photoacid catalysis, the reaction may also involve catalysis by a nonreversible acidic species formed upon irradiation.

**SCHEME 2:**
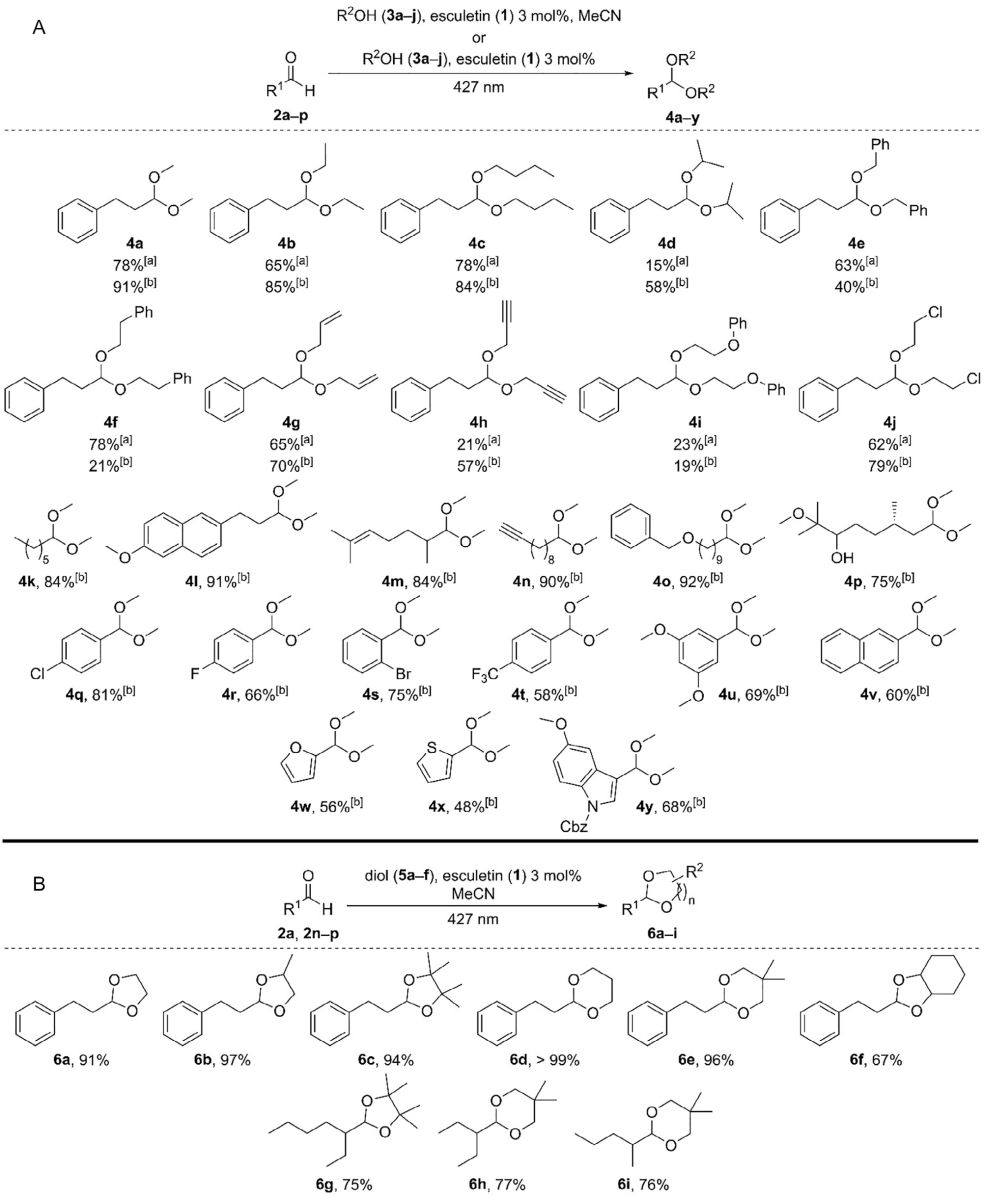
Substrate scope. (A) Acetalization of aldehydes with alcohols. (B) Acetalization of aldehydes with diols to afford cyclic acetals. ^a^The reaction was carried out in acetonitrile. ^b^The reaction was carried out using the alcohol as the solvent.

Next, we explored the substrate scope (Scheme 2). Using 3‐phenylpropanal (**2a**) as the aldehyde, a variety of alcohols were tested (Scheme 2A, **4a**–**j**). In each case, two sets of conditions were compared: one using acetonitrile as the solvent and the other using the alcohol itself as the solvent (Scheme 2A, **4a**–**j**). For most substrates, reactions carried out in neat alcohol afforded significantly higher isolated yields (**4b**–**4d**, **4g**, **4h**, **4j**). However, when alcohols containing a phenyl ring were employed, the use of acetonitrile as the solvent resulted in higher yields (**4e**, **4f**, **4i**). In general, acetals derived from primary alcohols, such as **4b**, **4c**, **4e**, or **4f**, were obtained in very good to excellent yields, whereas the more sterically hindered isopropyl alcohol afforded acetal **4d** in a moderate yield.

The methodology also tolerated a range of functional groups. Allyl alcohol (**4g**), propargyl alcohol (**4h**), and 2‐chloroethanol (**4j**) led to high to moderate yields, demonstrating tolerance toward alkenes, alkynes, and halogens. However, acetal **4i**, derived from 2‐phenoxyethanol, was isolated in a lower yield. We then explored the substrate scope of aliphatic aldehydes (Scheme 2A, **4k**–**4p**). All acetals were obtained in excellent yields. Aldehydes containing branched aliphatic chains (**4m**), ether functionalities (**4l**, **4o**), and unsaturated systems (**4m** and **4n**) were all well‐tolerated under the reaction conditions. Notably, when (3*S*)‐5‐(3,3‐dimethyloxiran‐2‐yl)‐3‐methylpentanal, bearing an epoxide ring, was employed, epoxide ring opening occurred in addition to acetalization. The ring opening took place at the more hindered position, affording acetal **4p** in a very good yield. To further broaden the aldehyde scope, a series of aromatic aldehydes was also tested (Scheme 2A, **4q**–**4v**). Acetals bearing halogen substituents on the phenyl ring (**4q**–**4s**) were obtained in moderate to excellent yields, no matter the substituent pattern (*para‐* or *ortho‐*). Acetals **4t** and **4u**, bearing electron‐withdrawing (trifluoromethyl) or electron‐donating (methoxy) substituents, respectively, were isolated in moderate yields. The lower efficiency observed for these substrates may arise from electronic effects that influence the electrophilicity of the carbonyl group and, consequently, the rate of the acid‐catalyzed acetalization. Substituents at all positions on the phenyl ring (*ortho*‐, *meta*‐, and *para*‐) were well‐tolerated. Notably, *ortho*‐ and *para*‐halogenated benzaldehydes afforded consistently high yields, indicating that moderate electron‐withdrawing substituents have little influence on the efficiency of the transformation under the optimized conditions. Additionally, 2‐naphthaldehyde afforded acetal **4v** in a moderate yield. To further evaluate the scope of the methodology, heteroaromatic aldehydes were also investigated. Furfural and thiophene‐2‐carboxaldehyde afforded the corresponding dimethyl acetals (**4w** and **4x**) in moderate isolated yields (56% and 48%, respectively), while *N*‐Cbz‐5‐methoxy‐1*H*‐indole‐3‐carbaldehyde furnished acetal **4y** in a 68% yield. These results demonstrate that the protocol is also applicable to heteroaromatic substrates, although diminished efficiencies were observed depending on the heterocyclic scaffold. We next investigated the compatibility of diols with the reaction conditions (Scheme 2B). Since most of the diols tested were solids or highly viscous liquids, all reactions involving diols were conducted using acetonitrile as the solvent. Additionally, since each diol contains two hydroxyl groups, the amount used was adjusted to half of plain alcohols, thus, 2.50 mmol. Acetals **6a**–**6e** were obtained in excellent yields. For acetal **6f**, *cis*/*trans*‐1,2‐cyclohexanediol was employed. The lower isolated yield (67%) of acetal **6f** may be attributed to the reduced reactivity of the *trans*‐isomer, due to steric reasons. Finally, short aliphatic aldehydes with high volatility were also subjected to acetalization with diols, aiming to reduce the volatility of the resulting acetals (Scheme 2B, **6g**–**6i**). Although acetals **6g**–**6i** remained relatively volatile, they could be concentrated under reduced pressure at low temperatures (10–15 °C), affording good isolated yields. The limitations of the methodology were also examined. Sterically hindered or poorly nucleophilic alcohols, including *tert*‐butanol, 2,2,2‐trichloro‐1‐ethanol, 2,2,2‐trifluoro‐1‐ethanol, and 2‐methoxyethanol, exhibited very low reactivity under the optimized conditions. Likewise, the electron‐deficient 4‐nitrobenzaldehyde proved unsuitable for the present protocol, whereas conjugated or photochemically sensitive aldehydes, such as cinnamaldehyde or phenylacetaldehyde, underwent competing side reactions, preventing the efficient formation of the corresponding acetals. Furthermore, aldehydes bearing basic moieties, such as indole‐5‐carboxaldehyde, 4‐pyridine‐carboxaldehyde, and 4‐dimethylamino‐benzaldehyde, are not compatible with this protocol.

Having established optimal reaction conditions for acetal synthesis, we decided to explore whether this protocol could also be extended to ketal formation. In most literature precedent, acetalization of aldehydes is common practice, but transition to ketones is usually highly problematic. In this regard, a short optimization was performed, using as a model the reaction between cyclohexanone (**7a**, 0.50 mmol) and methanol (**3a**, 1 mL). The optimal catalyst loading for esculetin was found to be 5 mol% in this case (Table 3, entry 2), instead of the 3 mol% used for acetals, affording ketal **8a** in a 74% yield, while drying additives resulted in turbid solutions and lower yields (Table 3, entries 4 and 5). Since ketones are not prone to aerobic oxidation, the ketalization reactions were performed in air.

**TABLE 3:** Optimization studies for the light‐mediated ketal formation between cyclohexanone (**7a**) and methanol (**3a**) in the presence of esculetin (**1**).^a^

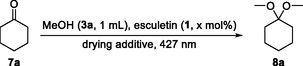			
Entry	Esculetin, mol%	Drying additive^b^	Isolated yield (%) of 8a
1	3	n. a.	51
**2**	**5**	**n. a.**	**74**
3	10	n. a.	52
4	5	Na_2_SO_4_ (1.2 eq.)	65
5	5	3 Å molecular sieves (100 mg/mL)	17

*Note:* Bold represents the optimal reaction conditions.

a A solution of cyclohexanone (**7a**, 49 mg, 0.50 mmol) and esculetin (**1**) in MeOH (**3a**, 1 mL) was irradiated for 16 h by a 40 W Kessil lamp.

b n. a., no additive.

**SCHEME 3:**
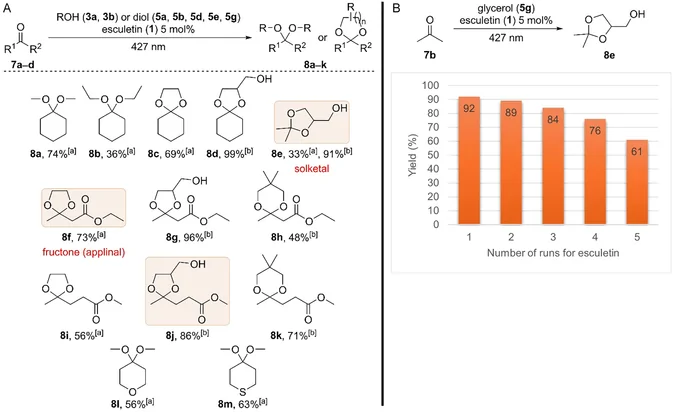
Visible‐light‐mediated synthesis of ketals. (A) Substrate scope. (B) Yield (%) of solketal (**8e**) after consecutive runs utilizing recycled esculetin. ^a^0.50 mmol of ketone and 1 mL of alcohol, diol, or glycerol were used. ^b^0.50 mmol of diol or glycerol and 1 mL of ketone were used.

The reaction between cyclohexanone and ethanol resulted in a relatively low 36% yield of **8b**. Nonetheless, the reaction between cyclohexanone and ethylene glycol or glycerol produced ketals **8c** and **8d** in high and quantitative yield, respectively (Scheme 3A). However, in the case of glycerol (**8d**), which is quite viscous, the ratio of reactants had to be reversed, using 0.50 mmol of alcohol and 1 mL of ketone. Interestingly, solely the formation of the five‐membered cyclic glycerol ketal was detected and isolated, whereas no formation of the corresponding six‐membered cyclic ketal was observed. We next examined the conversion of glycerol to solketal, an attractive industrial transformation, due to the excess glycerol produced as a byproduct of biodiesel and the use of solketal as a fuel additive and a fine chemical [21, 44, 45]. When 0.50 mmol of acetone and 1 mL of glycerol were employed, **8e** was isolated in a low yield (33%), due to the high viscosity of glycerol, which prevented good stirring and uniform irradiation of the reaction mixture. However, when the ratio was altered to 0.50 mmol of glycerol and 1 mL of acetone, solketal (**8e**) was obtained in excellent yield (91%). In this case, no formation of the six‐membered glycerol ketal was detected either. The ketal derived from the condensation of ethyl acetoacetate and ethylene glycol is another example of industrially interesting product, since it finds applications as a commercial fragrance [22]. This product, **8f**, known under the name fructone or applinal, was obtained in a 73% yield under our photochemical conditions. Further transformations of ethyl acetoacetate afforded ketal **8g** in almost quantitative yield, but **8h** in moderate yield. Finally, methyl levulinate was transformed into ketals **8i**, **8j**, and **8k** in high to very high yields. Levulinate cyclic ketals, such as **8i** or **8j**, have also found industrial applications, including their use as fuel additives and applications in plasticizers and polymers [46, 47, 48]. Overall, the proposed photochemical protocol may be used for the production of industrially interesting ketals under mild and environmentally friendly conditions. Finally, heterocyclic ketones tetrahydro‐4*H*‐pyran‐4‐one and tetrahydro‐4*H*‐thiopyran‐4‐one afforded their corresponding methyl ketals **8l** and **8m** in moderate isolated yields (56% and 63%, respectively), demonstrating that the present methodology is also applicable to heterocyclic ketone substrates.

To further explore the potential applicability of this protocol, we attempted the reaction leading to the formation of the solketal (**8e**) on a larger scale. Thus, glycerol (**5g**, 1.00 g, 10.86 mmol) and esculetin (**1**, 97 mg, 0.54 mmol) were dissolved in acetone (22 mL). After 22 h, solketal was isolated in a 92% yield. Furthermore, on a larger scale, **8e** was readily isolated after removal of the solvent, addition of approximately 1–2 mL of methanol, and precipitation of esculetin with diethyl ether, affording pure **8e**, with no need for chromatographic purification (Scheme 3B). The precipitated esculetin was then recycled and reused in the same reaction. Using the recycled esculetin in consecutive runs, solketal was obtained in comparable yields until the 4^th^ run, as presented in Scheme 3B. After the 4^th^ run, the yield dropped significantly to 61%, which could be attributed to the possible irreversible photodegradation of esculetin upon extended irradiation periods.

**SCHEME 4:**
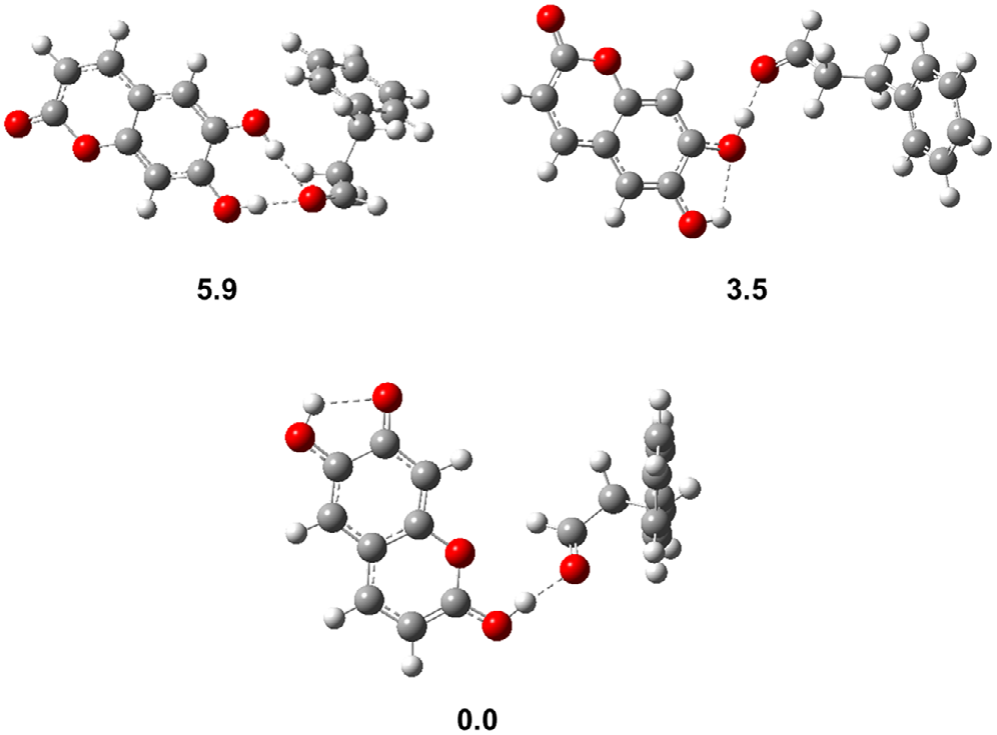
Relative energy ordering of the three possible conformers (kcal/mol) of esculetin(T_1_)‐3‐phenylpropanal complex (B3LYP/6‐31G(d,p) methodology, in MeOH, SMD Model).

In order to probe the esculetin‐photocatalyzed acetalization of 3‐phenylpropanal with methanol, DFT calculations were performed, and the results are presented in Scheme 4 and Supporting Information [42]. All steps were calculated using B3LYP/6‐31G(d,p) methodology in MeOH using the SMD solvation model, which is suitable for calculating Δ*G*. Initially, upon photoexcitation, esculetin is promoted from its ground state S_0_ to the first singlet excited state (S), with a calculated Δ*G*_exc_ = 73.5 kcal/mol, followed by intersystem crossing (ISC), leading to the more stable first triplet excited state (T_1_, see Figure S10 [42]), which lies 19.9 kcal/mol below its S_1_ state in free energy, corresponding to the tautomer conformation, which is in line with previous experimental studies on similar systems [37, 49]. Note that the lowest‐energy conformations of the S_0_ and S_1_ state of esculetin were used as starting geometries for the computations, as it has been previously reported by Yang and Liu [50]. Subsequently, esculetin, being in the T_1_ state, forms a hydrogen‐bonded complex with 3‐phenylpropanal, for which three conformations were examined (Scheme 4): a doubly intermolecular hydrogen‐bonded structure, a single intermolecular hydrogen bond via the more acidic O─H group of esculetin accompanied by an intramolecular O─H···O interaction, and one featuring a hydrogen‐bonded complex between the phototautomer and 3‐phenylpropanal, via the transferred proton, accompanied by an intramolecular O─H···O^−^ interaction. Consistent with previous related studies [37, 49, 51], the latter conformation is favored and was therefore used for subsequent mechanistic analysis. The resulting complex exhibited Δ*E*_bind_ = −8.5 kcal/mol, while Δ*G*_bind_ = 1.7 kcal/mol, showcasing that this interaction may take place in our experimental conditions. The positive value of Δ*G*_bind_ is attributed to entropic contributions, since entropy decreases upon complex formation. After proton transfer (see Figure S10 [42]), the following steps involve the classical acetalization reaction of the reactive protonated 3‐phenylpropanal with methanol.

Esculetin combines several attractive features as a photoacid catalyst, including its natural origin, commercial availability, metal‐free structure, and strong excited‐state photoacidity. Additionally, it operates efficiently at low catalyst loadings (3–5 mol%), comparable to Eosin Y and lower than those required for Schreiner’s thiourea and 6‐bromo‐naphthol. Importantly, esculetin significantly expands the applicability of photoacid‐catalyzed carbonyl protection to ketalization reactions, providing an extensive ketone substrate scope that surpasses previously reported organic photoacid catalysts. Together with the broad substrate scope demonstrated herein, these characteristics establish esculetin as an efficient and sustainable photoacid catalyst for acetalization and ketalization reactions.

## 3 Conclusion

In summary, we have developed a simple and efficient protocol for the photoacid‐catalyzed acetalization of aldehydes with alcohols under visible‐light irradiation. Esculetin, a small, naturally occurring, organic molecule, was shown to function as an efficient photoacid catalyst under irradiation at 427 nm, enabling the reaction to proceed employing a low catalyst loading. This methodology is compatible with a wide range of alcohols and aldehydes. Acetonitrile may be employed as the solvent, facilitating the effective incorporation of solid alcohols and diols in this protocol. A number of ketals, including chemicals with industrial applications, such as solketal, fructone, and levulinate‐based ketals, were successfully produced under mild conditions. Overall, this study highlights that the natural product esculetin is an efficient sustainable catalyst for acetalization/ketalization reactions under mild and environmentally friendly conditions, indicating that natural products constitute a wide pool for investigation toward the discovery of sustainable photocatalysts.

## Funding

This work was supported by the Hellenic Foundation for Research and Innovation (Grants 655 and 21044).

## Conflicts of Interest

The authors declare no conflicts of interest.

## Supporting information

Supplementary Material

## Data Availability

The data that support the findings of this study are available in the Supporting Information of this article.
