# Supplementary material for: The Natural Product Esculetin as an Efficient Photoacid for the Visible‐Light‐Mediated Acetalization/Ketalization Reaction
Source: ChemSusChem. 2026 Sep 26;19(19):e71042. doi: 10.1002/cssc.71042 (PMC13615548; doi:10.1002/cssc.71042)
Supplement: Supplementary file 1 [file CSSC-19-e71042-s001.pdf]

The Natural Product Esculetin as an Efficient Photoacid for the Visible Light-Mediated Acetalization/Ketalization Reaction

Maria A. Theodoropoulou,[a] E. Alexandros Routsi,[a] Christiana Mantzourani,[a] Olga G. Mountanea,[a] Demeter Tzeli,[b,c] Christoforos G. Kokotos*[a] and George Kokotos*[a]

[a] *Laboratory of Organic Chemistry, Department of Chemistry, National and Kapodistrian University of Athens, Panepistimiopolis, Athens 15771, Greece*

[b] *Laboratory of Physical Chemistry, Department of Chemistry, National and Kapodistrian University of Athens, Panepistimiopolis, Athens 15771, Greece*

[c] *Theoretical and Physical Chemistry Institute, National Hellenic Research Foundation, 48 Vassileos Constantinou Ave., Athens 11635, Greece*

*To whom correspondence should be addressed.

For C.G.K.:

E-mail: ckokotos@chem.uoa.gr.

For G.K.:

E-mail: gkokotos@chem.uoa.gr.

SUPPORTING INFORMATION

Table of Contents

	Page
General Remarks	S2
Optimization of the Reaction Conditions for the Photochemical Reaction of 3-Phenylpropanal with Methanol	S3
Optimization of the Reaction Conditions for the Photochemcial Reaction of Cyclohexanone with Ethanol	S8
Experimental Procedures	S9
Computational Studies	S27
References	S31
NMR Spectra	S34

General Remarks

Forced-flow chromatography on Merck® (Merck, Darmstadt, Germany) Kieselgel 60 F_{254} 230–400 mesh was used for the purification of the products, while aluminum-backed silica plates (0.2 mm, 60 F_{254}) were used for thin-layer chromatography (TLC). The visualization of the developed chromatograms was performed by UV light and/or using phosphomolybdic acid. The melting points were determined on a Buchi® 530 apparatus (Buchi, Flawil, Switzerland) and were uncorrected. ^{1}H-, ^{13}C-, and ^{19}F-NMR spectra were recorded on a Bruker Avance Neo (Bruker, Faellanden, Switzerland) (400 MHz, 100 MHz, and 377 MHz, respectively) and were internally referenced to residual solvent signals. The data for ^{1}H-NMR are reported as follows: chemical shift (δ ppm), multiplicity (s = singlet, d = doublet, t = triplet, q = quartet, quint = quintet, m = multiplet, and br = broad signal), coupling constant, integration and assignment. The data for ^{13}C-NMR are reported in terms of the chemical shift (δ ppm). The data for ^{19}F-NMR are reported in terms of the chemical shift (δ ppm), and are internally referenced to trifluoroacetic acid. High-resolution mass spectrometry (HRMS) spectra were recorded on a Bruker® Maxis Impact QTOF (Bruker Daltonics, Bremen, Germany) spectrometer. For all experiments, the intensity of the Kessil lamps was controlled at the maximum level with power consumption: 370 nm (max 43W), 390 nm (max 52W), 427 nm and 440 nm (max 45W), 456 nm (max 50W) and 467 nm (max 44W).

Optimization of the Reaction Conditions for the Photochemical Reaction of 3-Phenylpropanal with Methanol

Supplementary Table S1. Optimization studies for the determination of the optimal wavelength for the acetalization of 3-phenylpropanal (**2a**) with methanol (**3a**) in the presence of esculetin (**1**).[a]

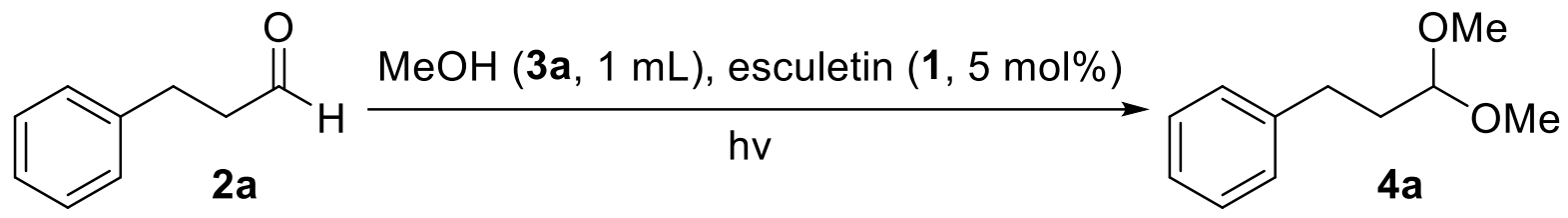

Entry	40W Kessil lamp, λ (nm)	Conversion (%) of **2a** to **4a**[b]	Variation
1[c]	-	0	under dark
2	370	12	
3	390	50 (20)	
**4**	**427**	**90 (82)**	
5[d]	427	37	open air
6	440	85 (50)	
7	456	16	
8	467	6	
9[e]	427	13	addition of $NaHCO_3$ (5 mol%)

[a] A solution of 3-phenylpropanal (**2a**, 67 mg, 0.50 mmol) and esculetin (**1**, 4.5 mg, 5 mol%, 0.025 mmol) in MeOH (**3a**, 1 mL) was irradiated for 16 h by a 40W Kessil lamp, under Ar. [b] (%) Conversion determined by ^{1}H-NMR; in parentheses the yield of **4a** after isolation by column chromatography. [c] The reaction was carried out under dark. [d] The reaction was carried out open air, and conversion to carboxylic acid (18%) and hemiacetal (20%) was also observed, while around 25% of the aldehyde remained unreacted; MeOH was partially evaporated. [e] 5 mol% $NaHCO_3$ was added.

Supplementary Table S2. Optimization studies for the determination of the catalyst loading of esculetin (**1**) for the acetalization of 3-phenylpropanal (**2a**) with methanol (**3a**).[a]

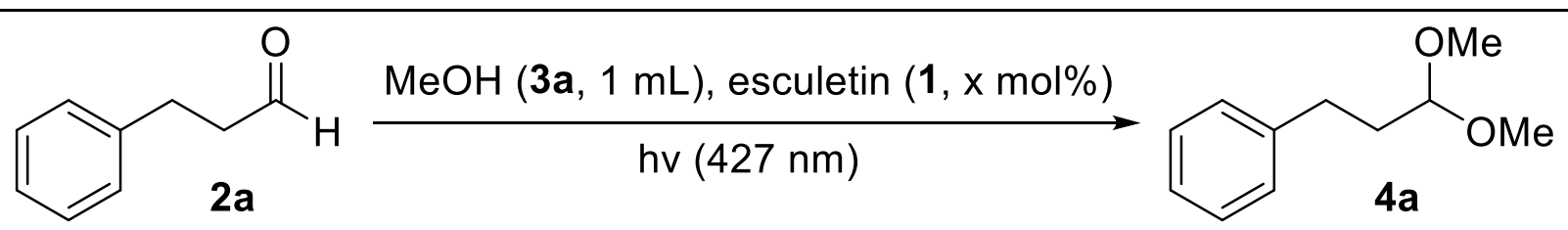

Entry	Esculetin (mol%)	Conversion (%) of **2a** to **4a**[b]
1	0	16
2	1	92 (80)
**3**	**3**	**97 (91)**
4	5	90 (82)
5	10	90 (83)

[a] A solution of 3-phenylpropanal (**2a**, 67 mg, 0.50 mmol) and different catalyst loadings of esculetin (**1**) in MeOH (**3a**, 1 mL) was irradiated for 16 h by a 40W Kessil lamp (427 nm), under Ar. [b] (%) Conversion determined by ^{1}H-NMR; in parentheses the yield of **4a** after isolation by column chromatography.

Supplementary Table S3. Optimization studies for the determination of the reaction time needed for the acetalization of 3-phenylpropanal (**2a**) with methanol (**3a**) in the presence of esculetin (**1**).[a]

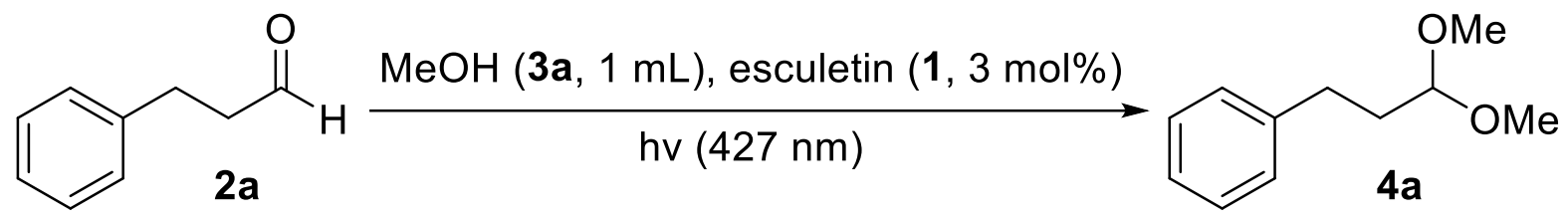

Entry	Reaction Time (hours)	Conversion (%) of **2a** to **4a**[b]
1	1	traces
2	3	11
3	6	38
**4**	**16**	**97**

[a] A solution of 3-phenylpropanal (**2a**, 67 mg, 0.50 mmol) and esculetin (**1**, 2.7 mg, 3 mol%, 0.015 mmol) in MeOH (**3a**, 1 mL) was irradiated by a 40W Kessil lamp (427 nm), under Ar. [b] (%) Conversion determined by ^{1}H-NMR.

Supplementary Table S4. Optimization studies for the determination of light intensity for the acetalization of 3-phenylpropanal (**2a**) with methanol (**3a**) in the presence of esculetin (**1**).[a]

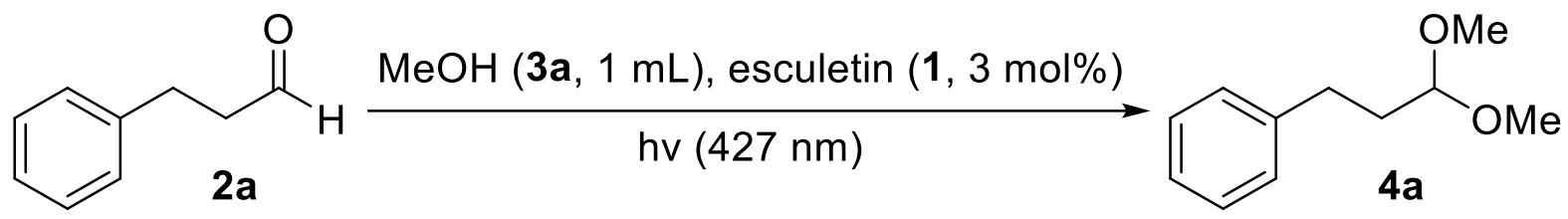

Entry	Intensity (%)	Conversion (%) of **2a** to **4a**[b]
1	25	49 (43)
2	50	89 (81)
3	75	94 (85)
**4**	**100**	**97 (91)**

[a] A solution of 3-phenylpropanal (**2a**, 67 mg, 0.50 mmol) and esculetin (**1**, 2.7 mg, 3 mol%, 0.015 mmol) in MeOH (**3a**, 1 mL) was irradiated by a 40W Kessil lamp (427 nm), under Ar. [b] (%) Conversion determined by ^{1}H-NMR; in parentheses the yield of **4a** after isolation by column chromatography.

Supplementary Table S5. Optimization studies for the determination of optimal lamp distance for the acetalization of 3-phenylpropanal (**2a**) with methanol (**3a**) in the presence of esculetin (**1**).[a]

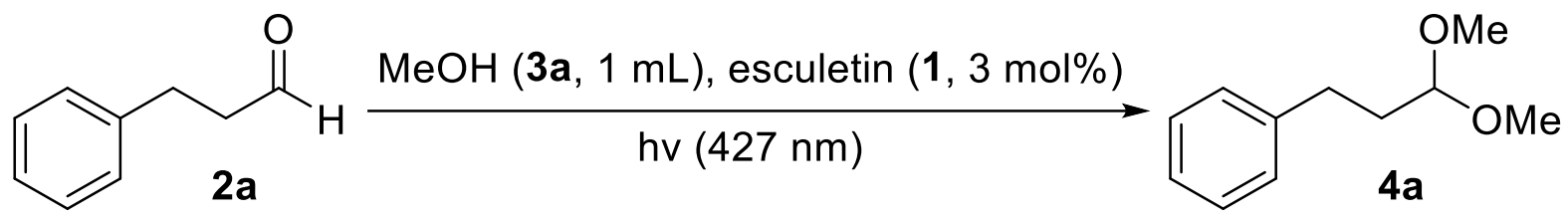

Entry	Distance (cm)	Conversion (%) of **2a** to **4a**[b]
1	2	97 (88)
**2**	**5**	**97 (91)**
3	8	84 (73)

[a] A solution of 3-phenylpropanal (**2a**, 67 mg, 0.50 mmol) and esculetin (**1**, 2.7 mg, 3 mol%, 0.015 mmol) in MeOH (**3a**, 1 mL) was irradiated by a 40W Kessil lamp (427 nm), under Ar. [b] (%) Conversion determined by ^{1}H-NMR; in parentheses the yield of **4a** after isolation by column chromatography.

Supplementary Table S6. Solvent screening for the acetalization of 3-phenylpropanal (**2a**) with methanol (**3a**) in the presence of esculetin (**1**).[a]

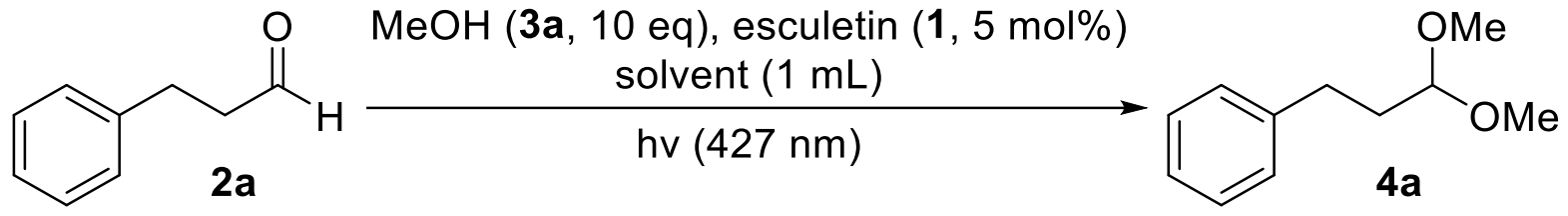

Entry	Solvents	Conversion (%) of **2a** to **4a**[b]
**1**	**CH_3CN**	**85 (77)**
2	$CHCl_3$	95 (80)
3	CH_2Cl_2	69 (60)
4	Petroleum ether	28
5	EtOAc	24
6	Et_2O	32
7	Methyl *tert*-butyl ether	41 (33)
8	2-Me-THF	13
9	1,4-Dioxane	3

[a] A solution of 3-phenylpropanal (**2a**, 67 mg, 0.50 mmol), MeOH (**3a**, 202 μL, 160 mg, 5.00 mmol) and esculetin (**1**, 4.5 mg, 5 mol%, 0.025 mmol) in various solvents (1 mL) was irradiated for 16 h by a 40W Kessil lamp (427 nm), under Ar. [b] (%) Conversion determined by ^{1}H-NMR; in parentheses the yield of **4a** after isolation by column chromatography.

Supplementary Table S7. Optimization studies for the determination of the equivalents of methanol needed for the acetalization of 3-phenylpropanal (**2a**) in the presence of esculetin (**1**) in acetonitrile.[a]

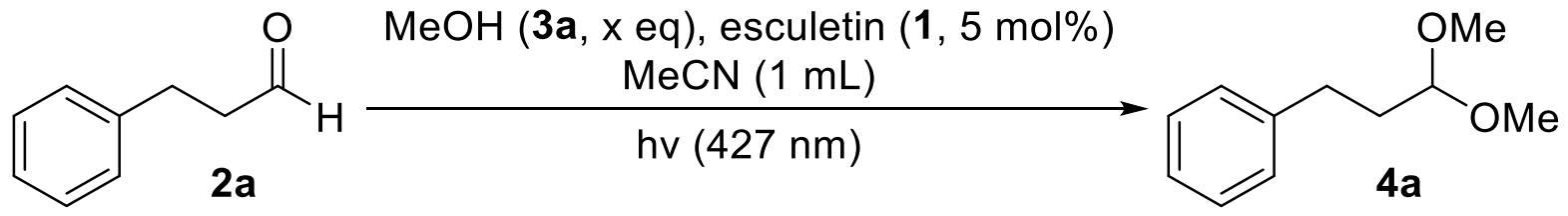

Entry	MeOH (equivalents)	Conversion (%) of **2a** to **4a**[b]
1	5.0	65 (49)
2	6.0	68 (57)
3	8.0	79 (69)
**4**	**10.0**	**85 (77)**

[a] A solution of 3-phenylpropanal (**2a**, 67 mg, 0.50 mmol), different quantities of MeOH (**3a**) and esculetin (**1**, 2.7 mg, 5 mol%, 0.015 mmol) in CH_3CN (1 mL) was irradiated for 16 h by a 40W Kessil lamp (427 nm), under Ar. [b] (%) Conversion determined by ^{1}H-NMR; in parentheses the yield of **4a** after isolation by column chromatography.

Supplementary Table S8. Optimization studies for the determination of the catalyst loading of esculetin (**1**) for the acetalization of 3-phenylpropanal (**2a**) with methanol (**3a**) in acetonitrile.[a]

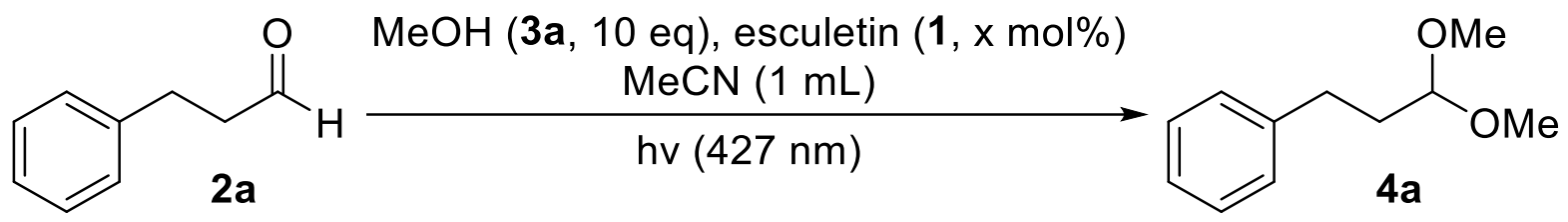

Entry	Esculetin (mol%)	Conversion (%) of **2a** to **4a**[b]
1	2.0	78 (69)
**2**	**3.0**	**81 (75)**
3	5.0	85 (77)

[a] A solution of 3-phenylpropanal (**2a**, 67 mg, 0.50 mmol), MeOH (**3a**, 202 μL, 160 mg, 5.00 mmol) and different catalyst loadings of esculetin (**1**) in CH_3CN (1 mL) was irradiated for 16 h by a 40W Kessil lamp (427 nm), under Ar. [b] (%) Conversion determined by ^{1}H-NMR; in parentheses the yield of **4a** after isolation by column chromatography.

Supplementary Table S9. Optimization studies for the determination of optimal quantity of acetonitrile for the acetalization of 3-phenylpropanal (**2a**) with methanol (**3a**) in the presence of esculetin (**1**).[a]

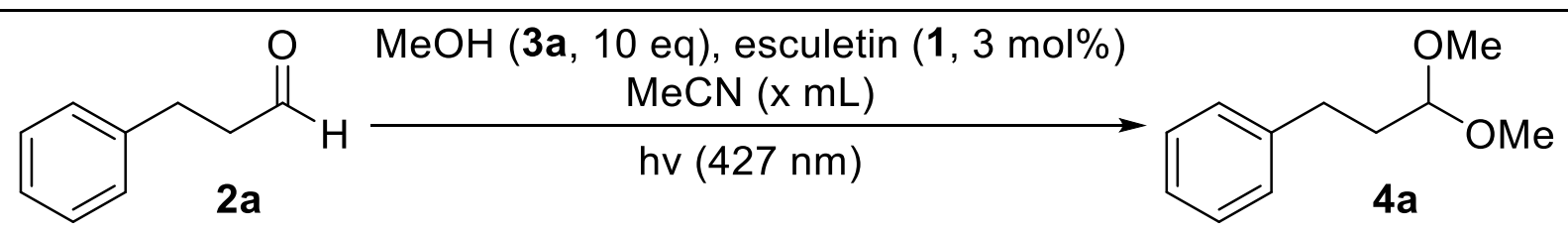

Entry	CH_3CN (mL)	Conversion (%) of **2a** to **4a**[b]	Variation
1	1.0	81 (75)	
**2**	**0.67**	**86 (78)**	
3[c]	0.67	48	addition of Na_2SO_4 (2 eq)
4	0.50	50	
5	0.25	43	

[a] A solution of 3-phenylpropanal (**2a**, 67 mg, 0.50 mmol), different quantities of MeOH (**3a**, 202 μL, 160 mg, 5.00 mmol) and esculetin (**1**, 2.7 mg, 3 mol%, 0.015 mmol) in CH_3CN was irradiated for 16 h by a 40W Kessil lamp (427 nm), under Ar. [b] (%) Conversion determined by ^{1}H-NMR; in parentheses the yield of **4a** after isolation by column chromatography. [c] 2 equivalents of anhydrous Na_2SO_4 were added to remove water.

Supplementary Table S10. Continuous irradiation and light-on/off experiments for the acetalization of 3-phenylpropanal (**2a**) with methanol (**3a**) in the presence of esculetin (**1**).[a]

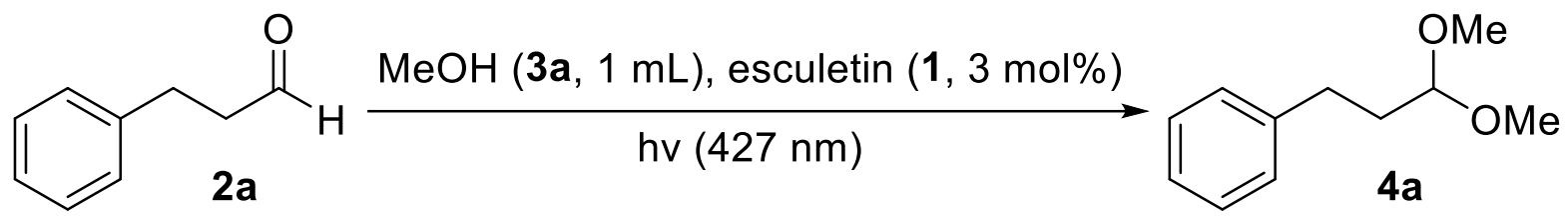

Entry	Reaction Time (hours)	Conversion (%) of **2a** to **4a**[b]	
		Continuous Irradiation	Light-On/Off
1	0	0	0
2	3	11	13
3	6	37	15
4	9	73	36
5	12	90	42
6	15	96	67
7	18	97	70

[a] A solution of 3-phenylpropanal (**2a**, 67 mg, 0.50 mmol) and esculetin (**1**, 2.7 mg, 3 mol%, 0.015 mmol) in MeOH (**3a**, 1 mL) was irradiated by a 40W Kessil lamp (427 nm), under Ar. [b] (%) Conversion determined by ^{1}H-NMR.

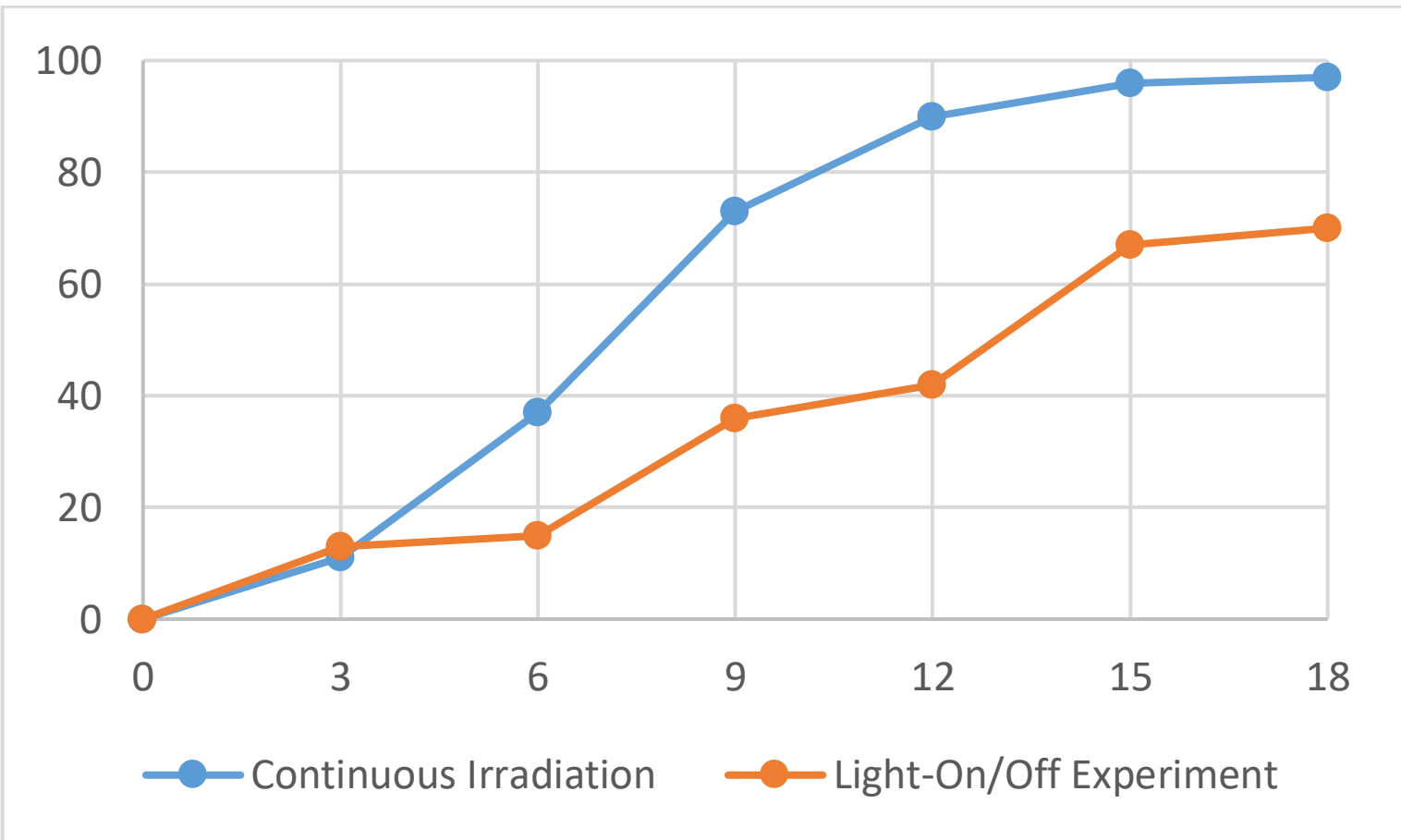

Supplementary Figure S1. Continuous irradiation and light-on/off experiments for the acetalization of 3-phenylpropanal (**2a**) with methanol (**3a**) in the presence of esculetin (**1**).

Supplementary Table S11. pH measurements of esculetin solutions under different irradiation conditions.

Entry	Conditions[a]	pH Value
1	No irradiation	4.60
2	Irradiation for 4 h, then measurement of pH within 10 min in the dark	3.39
3	Measurement of pH **during** irradiation for 3 min	3.31[b]

[a] In all cases, esculetin (17.8 mg, 0.10 mmol) was dissolved in MeOH (2.0 mL). Prior to pH measurement, H_2O was added (6.7 mL) to the solution. [b] The pH was monitored continuously during irradiation; since the pH gradually decreased overtime, the value recorded after 3 min of irradiation is reported.

Optimization of the Reaction Conditions for the Photochemical Reaction of Cyclohexanone with Ethanol

Supplementary Table S12. Optimization studies for the visible light-mediated ketal formation between cyclohexanone (**7a**) and ethanol (**3b**) in the presence of esculetin (**1**).[a]

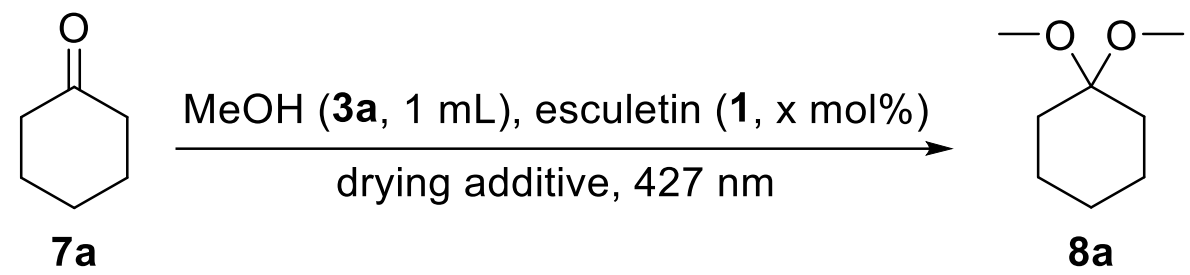

Entry	Esculetin (mol%)	Drying additive[b]	Isolated yield (%) of **8a**
1	3	n. a.	51
**2**	**5**	**n. a.**	**74**
3	10	n. a.	52
4	5	Na_2SO_4 (1.2 eq)	65
5	5	3 Å molecular sieves (100 mg/mL)	17

[a] A solution of cyclohexanone (**7a**, 49 mg, 0.50 mmol) and esculetin (**1**) in MeOH (**3a**, 1 mL) was irradiated for 16 h by a 40W Kessil lamp. [b] n. a.: no additive.

Experimental Procedures

Synthesis of acetals

Aldehyde (0.50 mmol), alcohol (5.00 mmol or 2.50 mmol in the case of diols) and esculetin (2.7 mg, 0.015 mmol) were dissolved in MeCN (670 µL). Alternatively, aldehyde (0.50 mmol) and esculetin (2.7 mg, 0.015 mmol) were dissolved in the appropriate alcohol (1.0 mL). The reaction mixture was left stirring at 427 nm (40 W Kessil lamp) at a distance of approximately 4.5 cm for 16 hours, under argon. The solvent was then evaporated under reduced pressure and ^{1}H-NMR spectra were obtained to determine the (%) conversion. The reaction mixture was dissolved in Et_2O (10 mL) and washed consecutively with a saturated aqueous solution of $NaHCO_3$ (10 mL) and a saturated aqueous solution of NaCl (10 mL). The organic layer was collected, dried over anhydrous Na_2SO_4, and concentrated under reduced pressure. The resulting residue was purified by column chromatography.

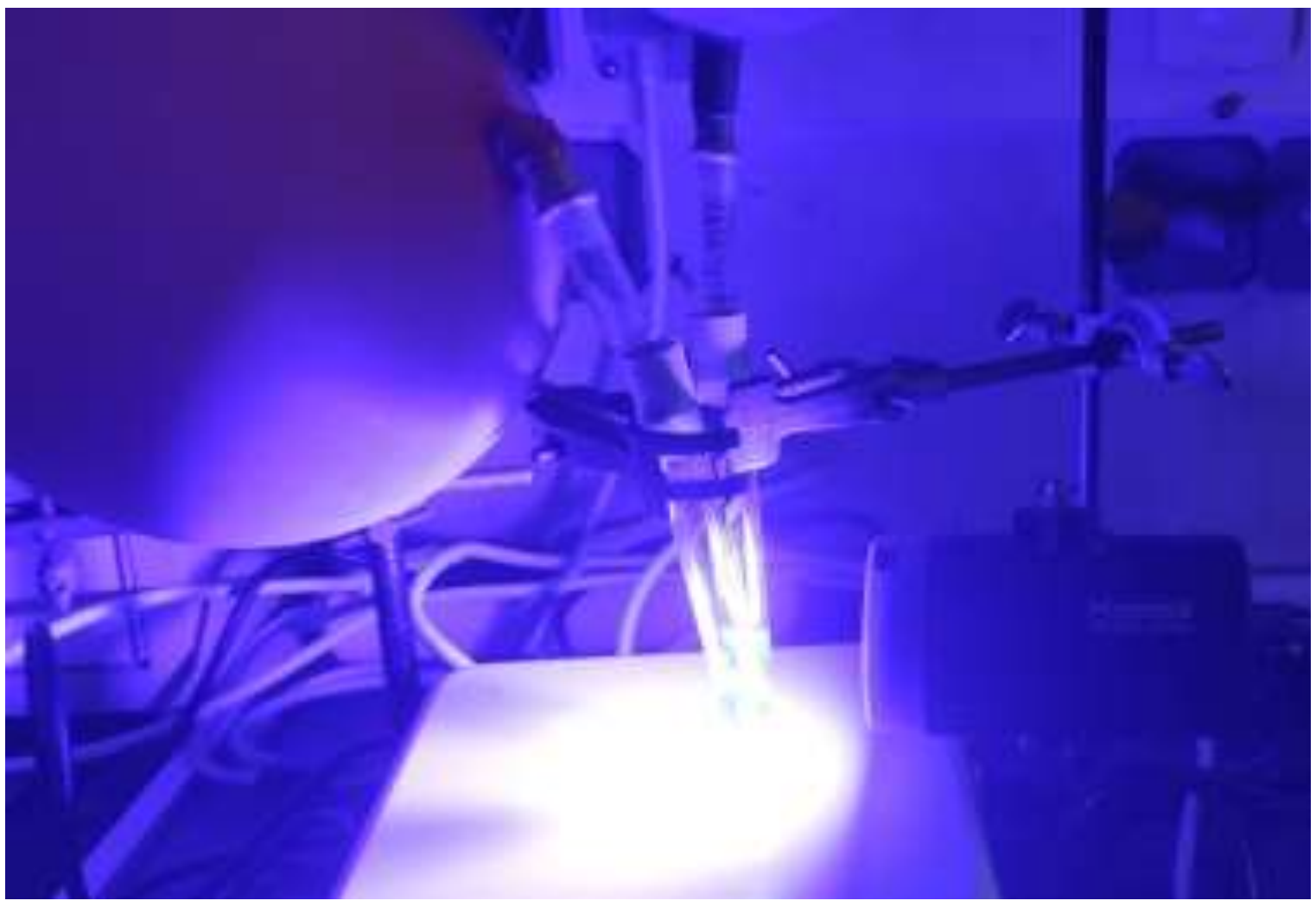

Supplementary Figure S2. Representative experimental setup for the synthesis of acetals.

(3,3-Dimethoxypropyl)benzene (4a) [1]

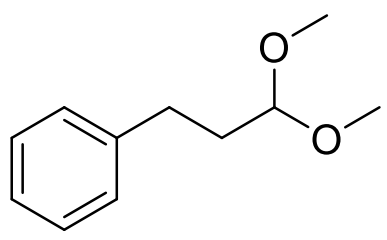

Elution solvent system: petroleum ether:EtOAc 95:5; Yield 78% (70 mg) in MeCN; Yield 82% (74 mg) in MeOH; Colorless oil; ^{1}H-NMR (400 MHz, $CDCl_3$): δ = 7.35–7.27 (m, 2H, 2 × ArH), 7.26–7.18 (m, 3H, 3 × ArH), 4.40 (t, J = 5.7 Hz, 1H, CH), 3.37 (s, 6H, 2 × OCH_3), 2.71 (t, J = 7.9 Hz, 2H, CH_2Ph), 1.99–1.92 (m, 2H, CH_2); ^{13}C-NMR

(100 MHz, $CDCl_3$): δ = 141.6, 128.4, 125.9, 103.8, 52.7, 34.1, 30.8; HRMS (ESI) $[M + Na]^+$ *m/z*: 203.1043; calculated for $[C_{11}H_{16}NaO_2]^+$ 203.1043.

(3,3-Diethoxypropyl)benzene (4b) [2]

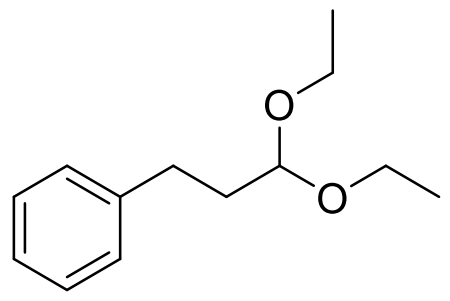

Elution solvent system: petroleum ether:EtOAc 95:5; Yield 65% (68 mg) in MeCN; Yield 85% (89 mg) in EtOH; Colorless oil; ^{1}H-NMR (400 MHz, $CDCl_3$): δ = 7.36–7.28 (m, 2H, 2 × ArH), 7.27–7.17 (m, 3H, 3 × ArH), 4.52 (t, *J* = 5.7 Hz, 1H, CH), 3.75–3.63 (m, 2H, 2 × OC*H*H), 3.58–3.48 (m, 2H, 2 × OCH*H*), 2.73 (t, *J* = 7.7 Hz, 2H, $PhCH_2$), 2.04–1.93 (m, 2H, CH_2), 1.25 (t, *J* = 7.1 Hz, 6H, 2 × CH_3); ^{13}C-NMR (100 MHz, $CDCl_3$): δ = 141.7, 128.4, 128.3, 125.8, 102.1, 61.0, 35.0, 31.0, 15.3; HRMS (ESI) $[M + Na]^+$ *m/z*: 231.1356; calculated for $[C_{13}H_{20}NaO_2]^+$ 231.1356.

(3,3-Dibutoxypropyl)benzene (4c) [2]

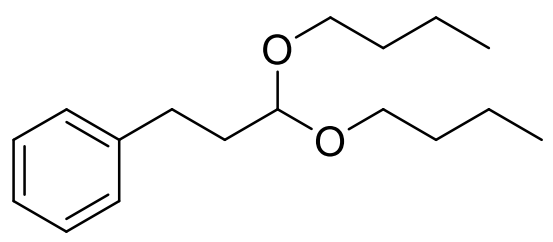

Elution solvent system: petroleum ether:EtOAc 95:5; Yield 78% (103 mg) in MeCN; Yield 84% (111 mg) in *n*-BuOH; Colorless oil; ^{1}H-NMR (400 MHz, $CDCl_3$): δ = 7.34–7.28 (m, 2H, 2 × ArH), 7.26–7.17 (m, 3H, 3 × ArH), 4.51 (t, *J* = 5.8 Hz, 1H, CH), 3.66–3.59 (m, 2H, 2 × OC*H*H), 3.49–3.41 (m, 2H, 2 × OCH*H*), 2.72 (t, *J* = 7.8 Hz, 2H, $PhCH_2$), 2.03–1.92 (m, 2H, CH_2), 1.64–1.55 (m, 4H, 2 × CH_2), 1.48–1.38 (m, 4H, 2 × CH_2), 0.96 (t, *J* = 7.1 Hz, 6H, 2 × CH_3); ^{13}C-NMR (100 MHz, $CDCl_3$): δ = 141.8, 128.4, 128.3, 125.8, 102.3, 65.3, 35.0, 32.0, 31.0, 19.4, 13.9; HRMS (ESI) $[M + Na]^+$ *m/z*: 287.1982; calculated for $[C_{17}H_{28}NaO_2]^+$ 287.1982.

(3,3-Diisopropoxypropyl)benzene (4d) [2]

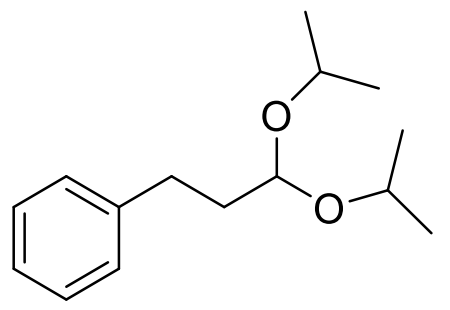

Elution solvent system: petroleum ether:EtOAc 95:5; Yield 15% (18 mg) in MeCN; Yield 58% (69 mg) in isopropyl alcohol; Colorless oil; ^{1}H-NMR (400 MHz, $CDCl_3$): δ = 7.33–7.28 (m, 2H, 2 × ArH), 7.25–7.20 (m, 3H, 3 × ArH), 4.59 (t, *J* = 5.8 Hz, 1H, CH), 3.88 (hept, *J* = 5.9 Hz, 2H, 2 × OCH), 2.72 (t, *J* = 7.7 Hz, 2H, $PhCH_2$), 1.98–1.91 (m, 2H, CH_2), 1.23 (d, *J* = 5.9 Hz, 6H, 2 × CH_3), 1.17 (d, *J* = 5.9 Hz, 6H, 2 × CH_3); ^{13}C-NMR (100 MHz, $CDCl_3$): δ = 142.0, 128.4, 128.3, 125.7, 99.6, 67.7, 36.9, 31.0, 23.4, 22.6; HRMS (ESI) $[M + Na]^+$ *m/z*: 259.1669; calculated for $[C_{15}H_{24}NaO_2]^+$ 259.1669.

(((3-Phenylpropane-1,1-diyl)bis(oxy))bis(methylene))dibenzene (4e) [3]

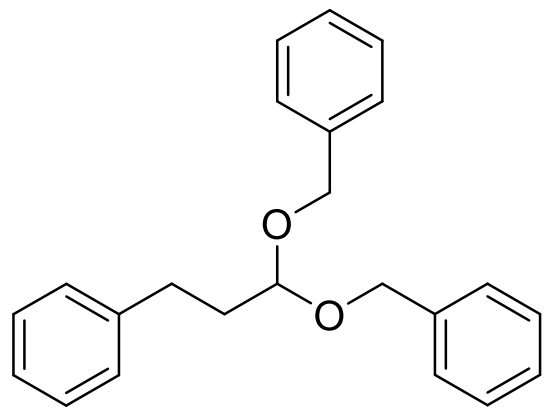

Elution solvent system: petroleum ether:EtOAc 95:5; Yield 63% (104 mg) in MeCN; Yield 40% (66 mg) in benzyl alcohol; Colorless oil; ^{1}H-NMR (400 MHz, $CDCl_3$): δ = 7.53–7.29 (m, 12H, 12 × ArH), 7.28–7.16 (m, 3H, 3 × ArH), 4.81 (t, J = 6.0 Hz, 1H, CH), 4.73 (d, J = 11.5 Hz, 2H, 2 × OC*H*H), 4.63 (d, J = 11.5 Hz, 2H, 2 × OCH*H*), 2.78 (t, J = 7.7 Hz, 2H, $PhCH_2$), 2.20–2.09 (m, 2H, CH_2); ^{13}C-NMR (100 MHz, $CDCl_3$): δ = 141.5, 138.1, 128.4, 128.4, 127.8, 127.6, 125.8, 101.4, 67.2, 34.9, 31.0; HRMS (ESI) $[M + Na]^+$ *m/z*: 355.1670; calculated for $[C_{23}H_{24}NaO_2]^+$ 355.1669.

(((3-Phenylpropane-1,1-diyl)bis(oxy))bis(ethane-2,1-diyl))dibenzene (4f) [4]

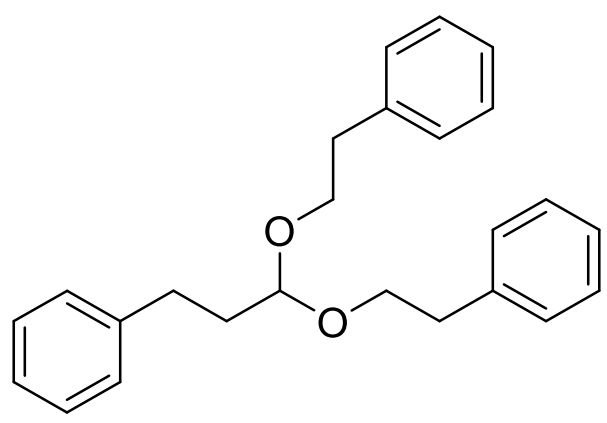

Elution solvent system: petroleum ether:EtOAc 95:5; Yield 78% (140 mg) in MeCN; Yield 21% (38 mg) in phenethyl alcohol; Colorless oil; ^{1}H-NMR (400 MHz, $CDCl_3$): δ = 7.36–7.21 (m, 13H, 13 × ArH), 7.15 (d, J = 7.2 Hz, 2H, 2 × ArH), 4.50 (t, J = 5.7 Hz, 1H, CH), 3.82–3.73 (m, 2H, 2 × OC*H*H), 3.67–3.58 (m, 2H, 2 × OCH*H*), 2.89 (t, J = 6.9 Hz, 4H, 2 × $PhCH_2$), 2.63 (t, J = 7.7 Hz, 2H, $PhCH_2$), 2.01–1.91 (m, 2H, CH_2); ^{13}C-NMR (100 MHz, $CDCl_3$): δ = 141.6, 139.0, 129.0, 128.4, 128.3, 128.3, 126.2, 125.8, 102.4, 66.4, 36.4, 34.8, 30.8; HRMS (ESI) $[M + Na]^+$ *m/z*: 383.1982; calculated for $[C_{25}H_{28}NaO_2]^+$ 383.1982.

(3,3-Bis(allyloxy)propyl)benzene (4g) [2]

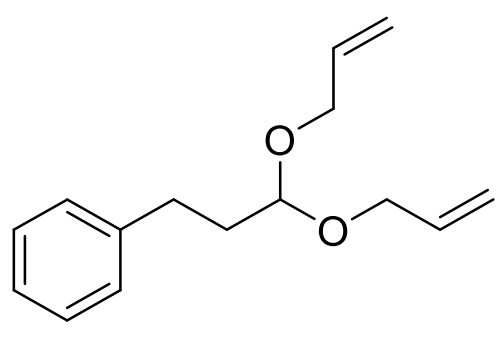

Elution solvent system: petroleum ether:EtOAc 95:5; Yield 65% (75mg) in MeCN; Yield 70% (81 mg) in allyl alcohol; Colorless oil; ^{1}H-NMR (400 MHz, $CDCl_3$): δ = 7.35–7.29 (m, 2H, 2 × ArH), 7.27–7.19 (m, 3H, 3 × ArH), 6.06–5.89 (m, 2H, 2 × =CH), 5.34 (d, J = 17.2 Hz, 2H, 2 × =C*H*H), 5.22 (d, J = 10.4 Hz, 2H, 2 × =CH*H*), 4.65 (t, J = 6.2 Hz, 1H, CH), 4.20–4.16 (dd, J_1 = 12.7 Hz, J_2 = 4.6 Hz, 2H, 2 × OC*H*H), 4.06 (dd,

J_1 = 12.7 Hz, J_2 = 4.6 Hz, 2H, 2 × OCH*H*), 2.75 (t, *J* = 7.7 Hz, 2H, $PhCH_2$), 2.07–1.99 (m, 2H, CH_2); ^{13}C-NMR (100 MHz, $CDCl_3$): δ = 141.6, 134.6, 128.4, 125.8, 116.7, 101.5, 66.3, 34.9, 30.9; HRMS (ESI) $[M + Na]^+$ *m/z*: 255.1357; calculated for $[C_{15}H_{20}NaO_2]^+$ 255.1356.

(3,3-Bis(prop-2-yn-1-yloxy)propyl)benzene (4h) [2]

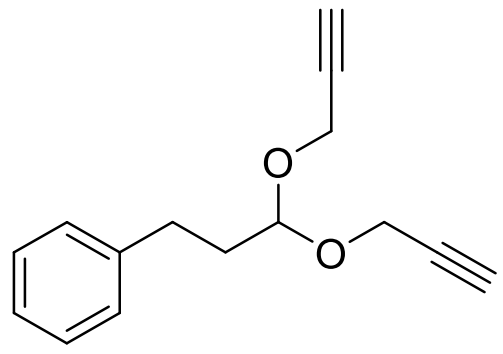

Elution solvent system: petroleum ether:EtOAc 95:5; Yield 21% (24 mg) in MeCN; Yield 57% (65 mg) in propargyl alcohol; Colorless oil; ^{1}H-NMR (400 MHz, $CDCl_3$): δ = 7.34–7.28 (m, 2H, 2 × ArH), 7.27–7.19 (m, 3H, 3 × ArH), 4.85 (t, *J* = 5.9 Hz, 1H, CH), 4.28 (d, *J* = 2.5 Hz, 4H, 2 × $OCH_2C\equiv$), 2.76 (t, *J* = 7.3 Hz, 2H, $PhCH_2$), 2.46 (t, *J* = 2.5 Hz, 2H, 2 × ≡CH), 2.07–2.00 (m, 2H, CH_2); ^{13}C-NMR (100 MHz, $CDCl_3$): δ = 141.2, 128.4, 128.4, 125.9, 100.9, 79.7, 74.3, 53.1, 34.7, 30.6; HRMS (ESI) $[M + Na]^+$ *m/z*: 251.1045; calculated for $[C_{15}H_{16}NaO_2]^+$ 251.1043.

((((3-Phenylpropane-1,1-diyl)bis(oxy))bis(ethane-2,1-diyl))bis(oxy))dibenzene (4i) [4]

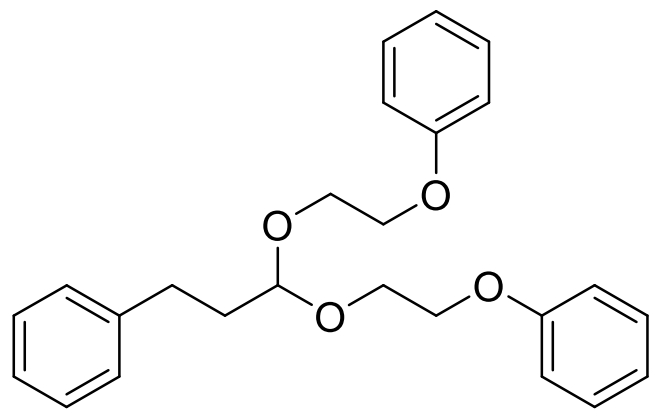

Elution solvent system: petroleum ether:EtOAc 95:5 gradient to 85:15; Yield 23% (46 mg) in MeCN; Yield 19% (37 mg) in 2-phenoxyethanol; Colorless oil; ^{1}H-NMR (400 MHz, $CDCl_3$): δ = 7.36–7.28 (m, 6H, 6 × ArH), 7.26–7.20 (m, 3H, 3 × ArH), 7.04–6.91 (m, 6H, 6 × ArH), 4.77 (t, *J* = 5.9 Hz, 1H, CH), 4.22–4.13 (m, 4H, 2 × OCH_2), 4.06–3.98 (m, 2H, 2 × OC*H*H), 3.96–3.87 (m, 2H, 2 × OCH*H*), 2.78 (t, *J* = 7.6 Hz, 2H, $PhCH_2$), 2.12–2.03 (m, 2H, CH_2); ^{13}C-NMR (100 MHz, $CDCl_3$): δ = 158.7, 141.4, 129.4, 128.5, 128.4, 125.9, 120.8, 114.5, 102.5, 67.2, 63.6, 34.6, 30.9; HRMS (ESI) $[M + Na]^+$ *m/z*: 415.1885; calculated for $[C_{25}H_{28}NaO_4]^+$ 415.1880.

(3,3-Bis(2-chloroethoxy)propyl)benzene (4j) [5]

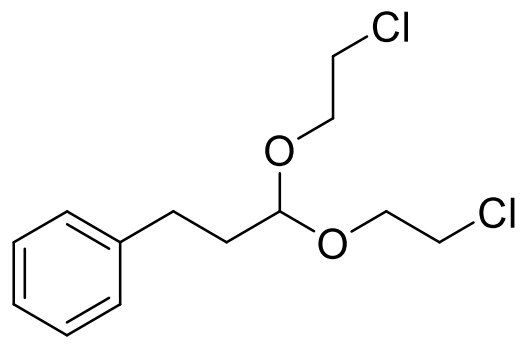

Elution solvent system: petroleum ether:EtOAc 95:5; Yield 62% (86 mg) in MeCN; Yield 79% (109 mg) in 2-chloroethanol; Colorless oil; ^{1}H-NMR (400 MHz, $CDCl_3$): δ = 7.36–7.29 (m, 2H, 2 × ArH), 7.28–7.20 (m, 3H, 3 × ArH), 4.67 (t, J = 5.8 Hz, 1H, CH), 3.93–3.86 m, 2H, 2 × OC*H*H), 3.83–3.75 (m, 2H, 2 × CH*H*), 3.68 (t, J = 5.6 Hz, 4H, 2 × $ClCH_2$), 2.76 (t, J = 7.8 Hz, 2H, $PhCH_2$), 2.07–1.99 (m, 2H, CH_2); ^{13}C-NMR (100 MHz, $CDCl_3$): δ = 141.1, 128.4, 128.4, 126.0, 102.3, 65.3, 43.3, 34.4, 30.8; HRMS (ESI) $[M + Na]^+$ *m/z*: 299.0576 / 301.0548 / 300.0610 / 303.0519; calculated for $[C_{13}H_{18}Cl_2NaO_2]^+$ 299.0577 / 301.0547 / 300.0610 / 303.0518.

1,1-Dimethoxyheptane (4k) [6]

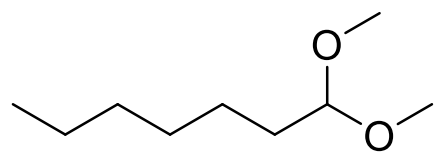

Elution solvent system: petroleum ether:EtOAc 95:5; Yield 84% (67 mg) in MeOH; Colorless oil; ^{1}H-NMR (400 MHz, $CDCl_3$): δ = 4.34 (t, J = 5.8 Hz, 1H, CH), 3.30 (s, 6H, 2 × OCH_3), 1.61–1.54 (m, 2H, CH_2), 1.36–1.23 (m, 8H, 4 × CH_2), 0.87 (t, J = 6.3 Hz, 3H, CH_3); ^{13}C-NMR (100 MHz, $CDCl_3$): δ = 104.6, 52.5, 32.5, 31.7, 29.1, 24.5, 22.5, 14.0; HRMS (ESI) $[M + Na]^+$ *m/z*: 183.1356; calculated for $[C_9H_{20}NaO_2]^+$ 183.1356.

2-(3,3-Dimethoxypropyl)-6-methoxynaphthalene (4l)

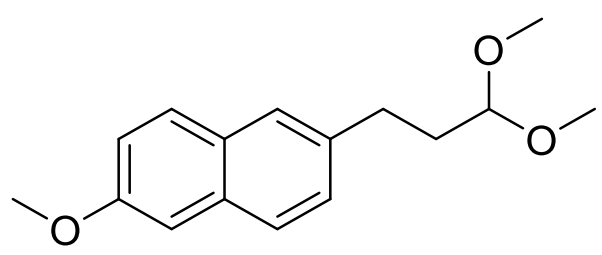

Elution solvent system: petroleum ether:EtOAc 80:20; Yield 91% (118 mg) in MeOH; White solid; Melting point: 58–59 °C; ^{1}H-NMR (400 MHz, $CDCl_3$): δ = 7.68 (d, J = 8.5 Hz, 2H, 2 × ArH), 7.57 (s, 1H, ArH), 7.31 (d, J = 8.4 Hz, 1H, ArH), 7.17–7.09 (m, 2H, 2 × ArH), 4.41 (t, J = 5.8 Hz, 1H, CH), 3.92 (s, 3H, OCH_3), 3.36 (s, 6H, 2 × OCH_3), 2.82 (t, J = 7.9 Hz, 2H, $PhCH_2$), 2.05–1.97 (m, 2H, CH_2); ^{13}C-NMR (100 MHz, $CDCl_3$): δ = 157.2, 136.8, 133.0, 129.1, 128.9, 127.7, 126.8, 126.2, 118.7, 105.7, 103. 8, 55.3, 52.7, 34.0, 30.8; HRMS (ESI) $[M + Na]^+$ *m/z*: 283.1305; calculated for $[C_{16}H_{20}NaO_3]^+$ 283.1305.

7,7-Dimethoxy-2,6-dimethylhept-2-ene (4m)

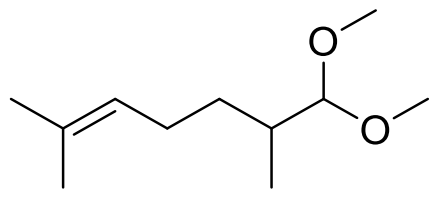

Elution solvent system: petroleum ether:CH_2Cl_2 70:30 gradient to 50:50; Yield 84% (78 mg) in MeOH; Colorless oil; ^{1}H-NMR (400 MHz, $CDCl_3$): δ = 5.09 (t, J = 7.2 Hz, 1H, =CH), 4.02 (d, J = 6.7 Hz, 1H, CH), 3.34 (s, 6H, 2 × OCH_3), 2.09–1.99 (m, 1H, C*H*H), 1.98–1.88 (m, 1H, CH*H*), 1.78–1.70 (m, 1H, CH), 1.67 (s, 3H, CH_3), 1.60 (s, 3H, CH_3), 1.56–1.48 (m, 1H, C*H*H), 1.17–1.07 (m, 1H, CH*H*), 0.90 (d, J = 6.5 Hz, 3H, CH_3); ^{13}C-NMR (100 MHz, $CDCl_3$): δ = 131.4, 124.6, 108.9, 54.0, 53.9, 35.2, 31.8, 25.7, 25.3, 17.6, 14.2; HRMS (ESI) [M + Na]$^+$ *m/z*: 209.1512; calculated for $[C_{11}H_{22}NaO_2]^+$ 209.1512.

11,11-Dimethoxyundec-1-yne (4n) [7]

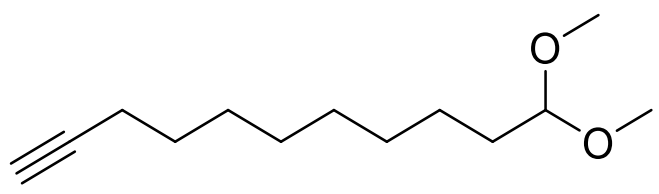

Elution solvent system: petroleum ether:EtOAc 95:5; Yield 90% (96 mg) in MeOH; Colorless oil; ^{1}H-NMR (400 MHz, $CDCl_3$): δ = 4.34 (t, J = 5.7 Hz, 1H, CH), 3.29 (s, 6H, 2 × OCH_3), 2.16 (t, J = 7.1 Hz, 2H, CH_2C≡), 1.92 (s, 1H, ≡CH), 1.60–1.54 (m, 2H, CH_2), 1.53–1.46 (m, 2H, CH_2), 1.41–1.26 (m, 10H, 5 × CH_2); ^{13}C-NMR (100 MHz, $CDCl_3$): δ = 104.5, 84.7, 68.0, 52.5, 32.4, 29.4, 28.9, 28.7, 28.4, 24.5, 18.3; HRMS (ESI) [M + Na]$^+$ *m/z*: 235.1669; calculated for $[C_{13}H_{24}NaO_2]^+$ 235.1669.

(((10,10-Dimethoxydecyl)oxy)methyl)benzene (4o)

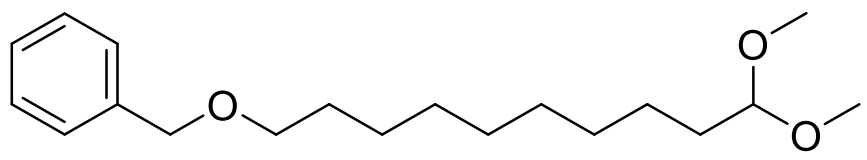

Elution solvent system: petroleum ether:EtOAc 90:10; Yield 92% (142 mg) in MeOH; Colorless oil; ^{1}H-NMR (400 MHz, $CDCl_3$): δ = 7.36–7.25 (m, 5H, 5 × ArH), 4.50 (s, 2H, $PhCH_2$), 4.35 (t, J = 5.7 Hz, 1H, CH), 3.46 (t, J = 6.6 Hz, 2H, OCH_2), 3.31 (s, 6H, 2 × OCH_3), 1.65–1.56 (m, 4H, 2 × CH_2), 1.39–1.26 (m, 12H, 6 × CH_2); ^{13}C-NMR (100 MHz, $CDCl_3$): δ = 138.7, 128.3, 127.6, 127.4, 104.6, 72.8, 70.5, 52.6, 32.5, 29.8, 29.5, 29.4, 26.2, 24.6; HRMS (ESI) [M + Na]$^+$ *m/z*: 331.2244; calculated for $[C_{19}H_{32}NaO_3]^+$ 331.2244.

(6*S*)-2,8,8-Trimethoxy-2,6-dimethyloctan-3-ol (4p) [8]

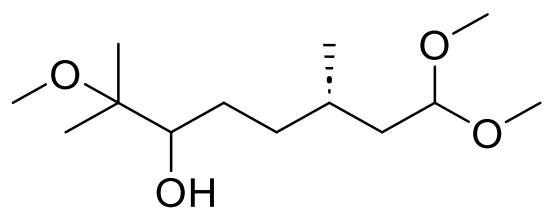

Elution solvent system: petroleum ether:EtOAc 90:10 gradient to 70:30; Yield 76% (94 mg) in MeOH; Colorless oil; ^{1}H-NMR (400 MHz, $CDCl_3$): δ = 4.47–4.39 (m, 1H, CH), 3.38–3.33 (m, 1H, C*H*OH), 3.30–3.22 (m, 6H, 2 × OCH_3), 3.18 (s, 3H, OCH_3), 2.51 (br, 1H, OH), 1.67–1.13 (m, 7H, 3 × CH_2, CH), 1.08 (s, 3H, CH_3), 1.04 (s, 3H, CH_3), 0.91–0.86 (m, 3H, CH_3); ^{13}C-NMR (100 MHz, $CDCl_3$): δ = 103.1, 77.4, 76.8, 52.7, 52.5, 52.3, 52.1, 48.9, 39.6, 39.2, 34.5, 34.3, 29.1, 29.0, 28.5, 28.4, 20.6, 19.8, 19.6, 18.7; HRMS (ESI) $[M + Na]^+$ *m/z*: 271.1881; calculated for $[C_{13}H_{28}NaO_4]^+$ 271.1880.

1-Chloro-4-(dimethoxymethyl)benzene (4q) [5]

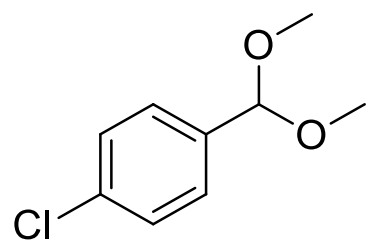

Elution solvent system: petroleum ether:EtOAc 90:10; Yield 81% (76 mg) in MeOH; Colorless oil; ^{1}H-NMR (400 MHz, $CDCl_3$): δ = 7.39 (d, *J* = 8.4 Hz, 2H, 2 × ArH), 7.34 (d, *J* = 8.4 Hz, 2H, 2 × ArH), 5.37 (s, 1H, CH), 3.31 (s, 6H, 2 × OCH_3); ^{13}C NMR (100 MHz, $CDCl_3$): δ = 136.6, 134.2, 128.3, 128.1, 102.2, 52.5; HRMS (ESI) $[M + Na]^+$ *m/z*: 209.0339 / 211.0305; calculated for $[C_9H_{11}ClNaO_2]^+$ 209.0340 / 211.0311.

1-(Dimethoxymethyl)-4-fluorobenzene (4r) [5]

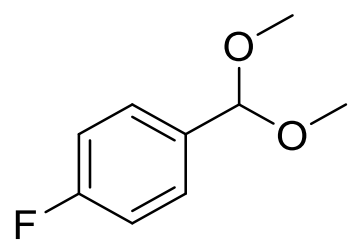

Elution solvent system: petroleum ether:EtOAc 90:10; Yield 66% (56 mg) in MeOH; Colorless oil; ^{1}H-NMR (400 MHz, $CDCl_3$): δ = 7.46–7.39 (m, 2H, 2 × ArH), 7.08–7.01 (m, 2H, 2 × ArH), 5.37 (s, 1H, CH), 3.31 (s, 6H, 2 × OCH_3); ^{13}C NMR (100 MHz, $CDCl_3$): δ = 162.8 (d, *J* = 244.8 Hz), 134.0 (d, *J* = 3.6 Hz), 128.5 (d, *J* = 7.9 Hz), 115.0 (d, *J* = 20.9 Hz), 102.5, 52.5; ^{19}F-NMR (377 MHz, $CDCl_3$): δ = −113.84; HRMS (ESI) $[M + Na]^+$ *m/z*: 193.0634; calculated for $[C_9H_{11}FNaO_2]^+$ 193.0635.

1-Bromo-2-(dimethoxymethyl)benzene (4s) [9]

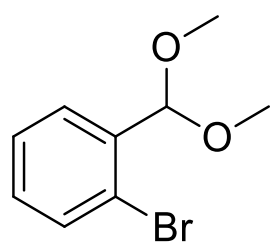

Elution solvent system: petroleum ether:CH_2Cl_2 70:30; Yield 75% (87 mg) in MeOH; Colorless oil; ^{1}H-NMR (400 MHz, $CDCl_3$): δ = 7.61 (d, J = 7.6 Hz, 1H, ArH), 7.56 (d, J = 8.0 Hz, 1H, ArH), 7.36–7.31 (m, 1H, ArH), 7.22–7.17 (m, 1H, ArH), 5.57 (s, 1H, CH), 3.39 (s, 6H, 2 × OCH_3); ^{13}C-NMR (100 MHz, $CDCl_3$): δ = 136.8, 132.9, 130.0, 128.3, 127.1, 122.9, 102.9, 53.9; HRMS (ESI) $[M + Na]^+$ *m/z*: 252.9833 / 254.9812; calculated for $[C_9H_{11}BrNaO_2]^+$ 252.9835 / 254.9815.

1-(Dimethoxymethyl)-4-(trifluoromethyl)benzene (4t) [5]

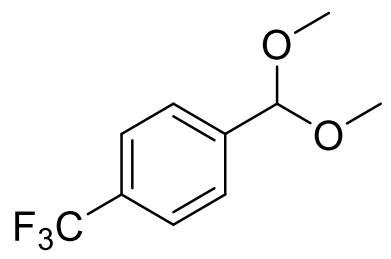

Elution solvent system: petroleum ether:EtOAc 95:5; Yield 58% (64 mg) in MeOH; Colorless oil; ^{1}H-NMR (400 MHz, $CDCl_3$): δ = 7.63 (d, J = 8.1 Hz, 2H, 2 × ArH), 7.58 (d, J = 8.1 Hz, 2H, 2 × ArH), 5.44 (s, 1H, CH), 3.33 (s, 6H, 2 × OCH_3); ^{13}C NMR (100 MHz, $CDCl_3$): δ = 142.0, 130.6 (q, J = 32.3 Hz), 127.2, 125.2 (q, J = 3.6 Hz), 124.1 (q, J = 270.4 Hz), 102.1, 52.6; ^{19}F-NMR (377 MHz, $CDCl_3$): δ = −62.64; HRMS (ESI) $[M + Na]^+$ *m/z*: 243.0602; calculated for $[C_{10}H_{11}F_3NaO_2]^+$ 243.0603.

1-(Dimethoxymethyl)-3,5-dimethoxybenzene (4u) [10]

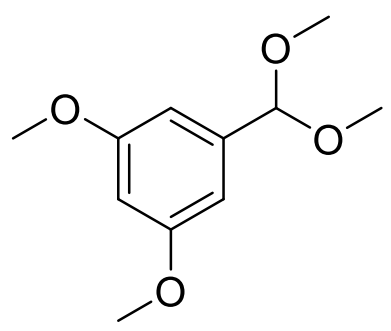

Elution solvent system: petroleum ether:EtOAc 90:10; Yield 69% (73 mg) in MeOH; Colorless oil; ^{1}H-NMR (400 MHz, $CDCl_3$): δ = 6.62 (s, 2H, 2 × ArH), 6.43 (s, 1H, ArH), 5.30 (s, 1H, CH), 3.79 (s, 6H, 2 × OCH_3), 3.33 (s, 6H, 2 × OCH_3); ^{13}C NMR (100 MHz, $CDCl_3$): δ = 160.7, 140.5, 104.5, 103.1, 100.7, 55.3, 52.8; HRMS (ESI) $[M + Na]^+$ *m/z*: 235.0941; calculated for $[C_{11}H_{16}NaO_4]^+$ 235.0941.

2-(Dimethoxymethyl)naphthalene (4v) [8]

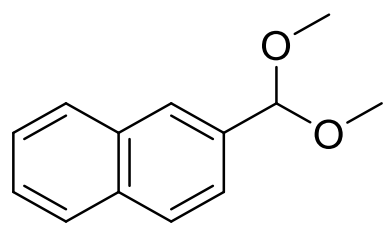

Elution solvent system: petroleum ether:EtOAc 90:10; Yield 60% (61 mg) in MeOH; Colorless oil; ^{1}H-NMR (400 MHz, $CDCl_3$): δ = 7.97 (s, 1H, ArH), 7.92–7.84 (m, 3H, 3 × ArH), 7.58 (d, J = 8.6 Hz, 1H, ArH), 7.54–7.47 (m, 2H, 2 × ArH), 5.58 (s, 1H, CH), 3.40 (s, 6H, 2 × OCH_3); ^{13}C NMR (100 MHz, $CDCl_3$): δ = 135.4, 133.4, 132.9, 128.3, 128.0, 127.6, 126.2, 126.1, 126.0, 124.3, 103.1, 52.7; HRMS (ESI) $[M + Na]^+$ *m/z*: 225.0887; calculated for $[C_{13}H_{14}NaO_2]^+$ 225.0886.

2-(Dimethoxymethyl)furan (4w) [5]

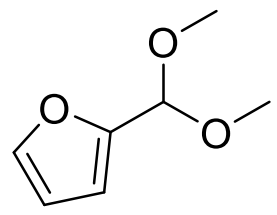

Elution solvent system: petroleum ether:EtOAc 95:5; Yield 56% (40 mg) in MeOH; Colorless liquid; ^{1}H-NMR (400 MHz, $CDCl_3$): δ = 7.40 (dd, J_1 = 1.7 Hz, J_2 = 0.9 Hz, 1H, ArH), 6.41 (dt, J_1 = 3.2 Hz, J_2 = 0.9 Hz, 1H, ArH), 6.36 (dd, J_1 = 3.2 Hz, J_2 = 1.7 Hz, 1H, ArH), 5.43 (s, 1H, CH), 3.35 (s, 6H, 2 × OCH_3); ^{13}C-NMR (100 MHz, $CDCl_3$): δ = 150.8, 142.4, 110.0, 108.4, 97.9, 52.8; MS (ESI) $[M+ H]^+$ *m/z*: 143.

2-(Dimethoxymethyl)thiophene (4x) [5]

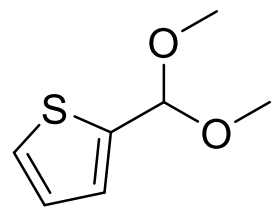

Elution solvent system: petroleum ether:EtOAc 95:5; Yield 48% (38 mg) in MeOH; Pale yellow liquid; ^{1}H-NMR (400 MHz, $CDCl_3$): δ = 7.30 (dd, J_1 = 5.0 Hz, J_2 = 1.1 Hz, 1H, ArH), 7.08 (dt, J_1 = 3.5 Hz, J_2 = 1.1 Hz, 1H, ArH), 7.01 (dd, J_1 = 5.0 Hz, J_2 = 3.5 Hz, 1H, ArH), 5.64 (s, 1H, CH), 3.36 (s, 6H, 2 × OCH_3); ^{13}C-NMR (100 MHz, $CDCl_3$): δ = 141.5, 126.6, 125.7, 125.4, 100.1, 52.5; MS (ESI) $[M+ H]^+$ *m/z*: 159.

Benzyl 3-(dimethoxymethyl)-5-methoxy-1*H*-indole-1-carboxylate (4y)

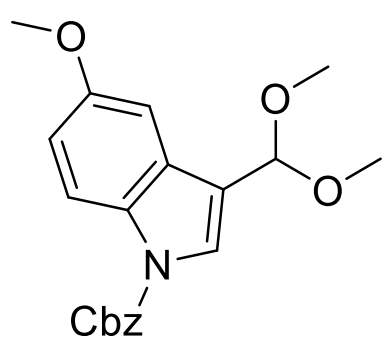

Elution solvent system: petroleum ether:EtOAc 90:10; Yield 68% (122 mg) in MeOH; Colorless liquid; ^{1}H-NMR (400 MHz, Acetone-d_6): δ = 8.07 (d, J = 9.1 Hz, 1H, ArH), 7.64 (s, 1H, ArH), 7.62–7.56 (m, 2H, 2 × ArH), 7.49–7.39 (m, 3H, 3 × ArH), 7.21 (d, J = 2.6 Hz, 1H, ArH), 6.97 (dd, J_1 = 9.1, J_2 = 2.6 Hz, 1H, ArH), 5.67 (d, J = 1.1 Hz, 1H, CH), 5.50 (s, 2H, OCH_2), 3.84 (s, 3H, OCH_3), 3.31 (s, 6H, 2 × OCH_3); ^{13}C-NMR (100 MHz, Acetone-d_6): δ = 156.4, 150.4, 135.7, 130.3, 129.2, 128.6, 128.6, 128.6, 124.8, 119.2, 115.6, 113.3, 103.0, 98.8, 68.5, 55.0, 51.5; HRMS (ESI) $[M + Na]^+$ *m/z*: 378.1315; calculated for $[C_{20}H_{21}NNaO_5]^+$ 378.1312.

2-Phenethyl-1,3-dioxolane (6a) [2]

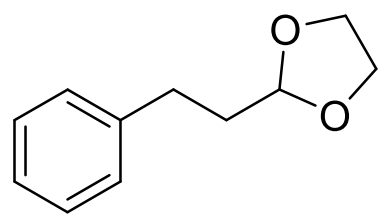

Elution solvent system: petroleum ether:EtOAc 95:5; Yield 91% (81 mg) in MeCN; Colorless oil; ^{1}H-NMR (400 MHz, $CDCl_3$): δ = 7.35–7.29 (m, 2H, 2 × ArH), 7.28–7.19 (m, 3H, 3 × ArH), 4.93 (t, J = 5.7 Hz, 1H, CH), 4.09–3.98 (m, 2H, 2 × OC*H*H), 3.96–3.86 (m, 2H, 2 × OCH*H*), 2.79 (t, J = 7.8 Hz, 2H, $PhCH_2$), 2.07–1.98 (m, 2H, CH_2); ^{13}C-NMR (100 MHz, $CDCl_3$): δ = 141.5, 128.3, 125.8, 103.8, 64.9, 35.5, 30.1; HRMS (ESI) $[M + Na]^+$ *m/z*: 201.0886; calculated for $[C_{11}H_{14}NaO_2]^+$ 201.0886.

4-Methyl-2-phenethyl-1,3-dioxolane (6b) [11]

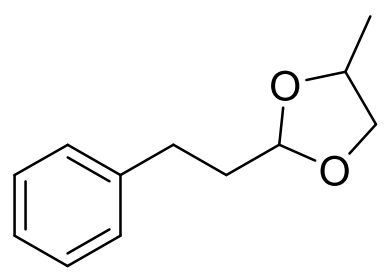

Elution solvent system: petroleum ether:EtOAc 95:5; Yield 97% (93 mg) in MeCN; Two diastereomers; Colorless oil; ^{1}H-NMR (400 MHz, $CDCl_3$): δ = 7.38–7.19 (m, 5H, 5 × ArH), 5.10 (t, J = 5.9 Hz, 0.2H, 0.2 × CH), 4.99 (t, J = 5.9 Hz, 0.8H, 0.8 × CH), 4.33–4.26 (m, 0.2H, 0.2 × OC*H*(CH_3)) 4.25–4.17 (m, 1H, 0.8 × OC*H*(CH_3), 0.2 × OC*H*H), 4.00 (t, J = 7.0 Hz, 0.8H, 0.8 × OC*H*H), 3.49 (t, J = 7.0 Hz, 0.8H, 0.8 × OCH*H*), 3.44 (t, J = 7.1 Hz, 0.2H, 0.2 × OCH*H*), 2.85–2.76 (m, 2H, $PhCH_2$), 2.08–1.97 (m, 2H, CH_2), 1.36 (d, J = 5.8 Hz, 2.4H, 0.8 × CH_3), 1.31 (d, J = 5.8 Hz, 0.6H, 0.2 × CH_3); ^{13}C-NMR (100 MHz, $CDCl_3$): δ = 141.6, 128.3, 128.3, 125.8, 103.8, 103.0, 72.8, 71.9, 71.8, 70.9, 35.9, 35.7, 30.0, 30.0, 18.6, 18.3; HRMS (ESI) $[M + Na]^+$ *m/z*: 215.1043; calculated for $[C_{12}H_{16}NaO_2]^+$ 215.1043.

4,4,5,5-Tetramethyl-2-phenethyl-1,3-dioxolane (6c) [12]

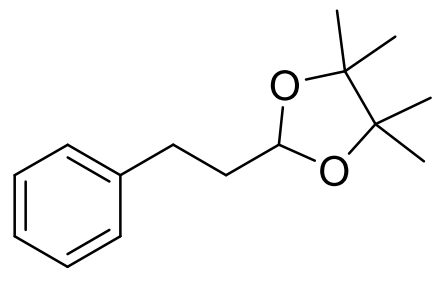

Elution solvent system: petroleum ether:EtOAc 95:5; Yield 94% (110 mg) in MeCN; Colorless oil; ^{1}H-NMR (400 MHz, $CDCl_3$): δ = 7.36–7.29 (m, 2H, 2 × ArH), 7.28–7.18 (m, 3H, 3 × ArH), 5.14 (t, J = 5.9 Hz, 1H, CH), 2.78 (t, J = 8.3 Hz, 2H, $PhCH_2$), 2.01–1.93 (m, 2H, CH_2), 1.27 (s, 6H, 2 × CH_3), 1.26 (s, 6H, 2 × CH_3); ^{13}C-NMR (100 MHz, $CDCl_3$): δ = 141.8, 128.3, 125.7, 100.1, 81.7, 37.8, 30.5, 24.2, 22.1; HRMS (ESI) $[M + Na]^+$ *m/z*: 257.1512; calculated for $[C_{15}H_{22}NaO_2]^+$ 257.1512.

2-Phenethyl-1,3-dioxane (6d) [13]

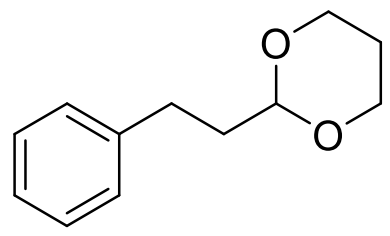

Elution solvent system: petroleum ether:EtOAc 95:5; Yield >99% (96 mg) in MeCN; Colorless oil; ^{1}H-NMR (400 MHz, $CDCl_3$): δ = 7.39–7.30 (m, 2H, 2 × ArH), 7.29–7.18 (m, 3H, 3 × ArH), 4.56 (t, J = 6.0 Hz, 1H, CH), 4.16 (dd, J_1 = 11.3 Hz, J_2 = 3.0 Hz, 2H, 2 × OC*H*H), 3.79 (td, J_1 = 12.0 Hz, J_2 = 2.4 Hz, 2H, 2×OCH*H*), 2.77 (t, J = 7.9 Hz, 2H, $PhCH_2$), 2.21–2.07 (m, 1H, C*H*H), 2.02–1.92 (m, 2H, CH_2), 1.37 (d, J = 13.4 Hz, 1H, CH*H*); ^{13}C-NMR (100 MHz, $CDCl_3$): δ = 141.6, 128.4, 128.3, 125.7, 101.4, 66.8, 36.6, 30.0, 25.8; HRMS (ESI) $[M + Na]^+$ m/z: 215.1043; calculated for $[C_{12}H_{16}NaO_2]^+$ 215.1043.

5,5-Dimethyl-2-phenethyl-1,3-dioxane (6e) [14]

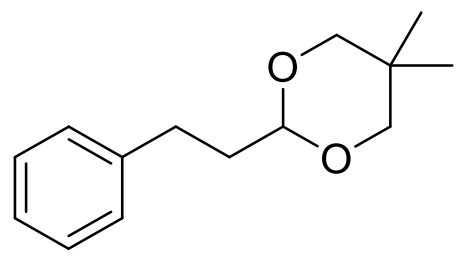

Elution solvent system: petroleum ether:EtOAc 95:5; Yield 96% (106 mg) in MeCN; Colorless oil; ^{1}H-NMR (400 MHz, $CDCl_3$): δ = 7.35–7.28 (m, 2H, 2 × ArH), 7.27–7.18 (m, 3H, 3 × ArH), 4.45 (t, J = 5.5 Hz, 1H, CH), 3.66 (d, J = 11.0 Hz, 2H, 2 × OC*H*H), 3.45 (d, J = 11.0 Hz, 2H, 2 × OCH*H*), 2.79 (t, J = 7.8 Hz, 2H, $PhCH_2$), 2.04–1.96 (m, 2H, CH_2), 1.25 (s, 3H, CH_3), 0.75 (s, 3H, CH_3); ^{13}C-NMR (100 MHz, $CDCl_3$): δ = 141.7, 128.4, 128.3, 125.8, 101.3, 77.2, 36.2, 30.2, 30.0, 23.0, 21.8; HRMS (ESI) $[M + Na]^+$ m/z: 243.1358; calculated for $[C_{14}H_{20}NaO_2]^+$ 243.1356.

2-Phenethylhexahydrobenzo[*d*][1,3]dioxole (6f)

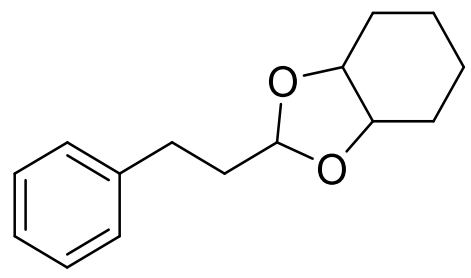

Elution solvent system: petroleum ether:EtOAc 95:5; Yield 67% (78 mg) in MeCN; Four diastereomers; Colorless oil; ^{1}H-NMR (400 MHz, $CDCl_3$): δ = 7.38–7.17 (m, 5H, 5 × ArH), 5.34 (t, J = 5.9 Hz, 0.04H, 0.04 × CH), 5.23 (t, J = 5.9 Hz, 0.07H, 0.07 × CH), 5.03 (t, J = 5.9 Hz, 0.88H, 0.88 × CH), 4.82 (t, J = 5.9 Hz, 0.01H, 0.01 × CH), 4.17–4.11 (m, 0.1H, 2 × 0.05 × OCH), 4.10–3.99 (m, 1.76H, 2 × 0.88 × OCH), 3.33–3.22 (m, 0.14H, 2 × 0.07 × OCH), 2.87–2.75 (m, 2H, $PhCH_2$), 2.24–2.17 (m, 0.14H, 0.07 × CH_2), 2.10–2.02 (m, 1.86H, 0.93 × CH_2), 2.00–1.91 (m, 0.16H, 2 × 0.04 × CH_2), 1.88–1.74 (m, 3.84H, 4 × 0.96 × CH_2), 1.68–1.50 (m, 2H, CH_2), 1.40–1.29 (m, 2H,

CH_2); ^{13}C-NMR (100 MHz, $CDCl_3$): δ = 141.6, 128.3, 125.8, 104.1, 103.3, 102.2, 81.9, 79.8, 74.3, 74.0, 36.8, 30.4, 30.2, 30.1, 29.0, 28.6, 28.4, 27.0, 23.7, 23.7, 21.1, 20.7; HRMS (ESI) $[M + Na]^+$ *m/z*: 255.1356; calculated for $[C_{15}H_{20}NaO_2]^+$ 255.1356.

2-(Heptan-3-yl)-4,4,5,5-tetramethyl-1,3-dioxolane (6g)

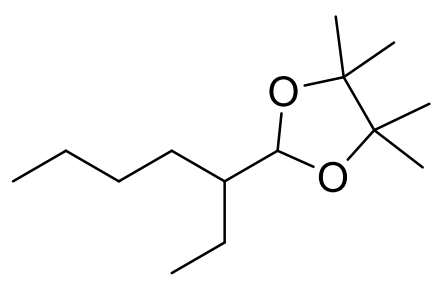

Elution solvent system: petroleum ether:Et_2O 99:1; Yield 75% (86 mg) in MeCN; Colorless oil; ^{1}H-NMR (400 MHz, $CDCl_3$): δ = 4.88 (d, J = 5.2 Hz, 1H, CH), 1.55–1.42 (m, 2H, CH, C*H*H), 1.39–1.25 (m, 7H, CH*H*, 3 × CH_2), 1.18 (s, 6H, 2 × CH_3), 1.17 (s, 6H, 2 × CH_3), 0.93–0.86 (m, 6H, 2 × CH_3); ^{13}C-NMR (100 MHz, $CDCl_3$): δ = 102.8, 81.2, 44.3, 29.2, 28.2, 24.3, 24.3, 23.1, 22.2, 21.6, 14.1, 11.2; HRMS (ESI) $[M + Na]^+$ *m/z*: 251.1981; calculated for $[C_{14}H_{28}NaO_2]^+$ 251.1982.

5,5-Dimethyl-2-(pentan-3-yl)-1,3-dioxane (6h)

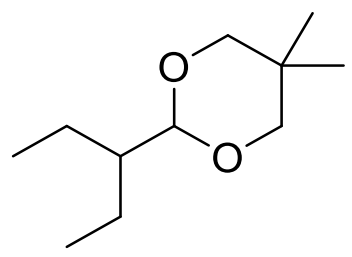

Elution solvent system: petroleum ether:Et_2O 98:2; Yield 77% (72 mg) in MeCN; Colorless oil; ^{1}H-NMR (400 MHz, $CDCl_3$): δ = 4.35 (d, J = 3.5 Hz, 1H, CH), 3.60 (d, J = 10.7 Hz, 2H, 2 × OC*H*H), 3.39 (d, J = 10.7 Hz, 2H, 2 × OCH*H*), 1.59–1.48 (m, 2H, CH, C*H*H), 1.43–1.31 (m, 3H, CH*H*, CH_2) 1.17 (s, 3H, CH_3), 0.89 (t, J = 7.2 Hz, 6H, 2 × CH_3), 0.69 (s, 3H, CH_3); ^{13}C-NMR (100 MHz, $CDCl_3$): δ = 103.8, 77.4, 45.5, 30.2, 23.0, 21.8, 21.0, 11.6; HRMS (ESI) $[M + Na]^+$ *m/z*: 209.1512; calculated for $[C_{11}H_{22}NaO_2]^+$ 209.1512.

5,5-Dimethyl-2-(pentan-2-yl)-1,3-dioxane (6i)

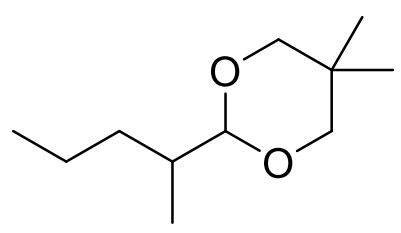

Elution solvent system: petroleum ether:Et_2O 98:2; Yield 76% (71 mg) in MeCN; Colorless oil; ^{1}H-NMR (400 MHz, $CDCl_3$): δ = 4.22 (d, J = 3.5 Hz, 1H, CH), 3.59 (d, J = 10.7 Hz, 2H, 2 × OC*H*H), 3.39 (d, J = 10.7 Hz, 2H, 2 × OCH*H*), 1.71–1.63 (m, 1H, CH), 1.55–1.47 (m, 1H, C*H*H), 1.44–1.34 (m, 1H, CH*H*), 1.30–1.08 (m, 5H, CH_2, CH_3), 0.93 (d, J = 6.8 Hz, 3H. CH_3), 0.89 (t, J = 7.3 Hz, 3H, CH_3), 0.70 (s, 3H, CH_3); ^{13}C-NMR (100 MHz, $CDCl_3$): δ = 105.1, 77.3, 37.4, 33.6, 30.2, 22.9, 21.8, 20.2, 14.3, 13.8; HRMS (ESI) $[M + Na]^+$ *m/z*: 209.1509; calculated for $[C_{11}H_{22}NaO_2]^+$ 209.1512.

Synthesis of ketals

For ketals **8a**–**c**, **8e**, **8f** and **8i**, ketone (0.50 mmol) and esculetin (4.5 mg, 0.025 mmol) were dissolved in the appropriate alcohol (1.0 mL) (method A). Alternatively, for ketals **8d**, **8e**, **8g**, **8h**, **8j** and **8k**, diol (0.50 mmol) and esculetin (4.5 mg, 0.025 mmol) were dissolved in the appropriate ketone (1.0 mL) (method B). The reaction mixture was left stirring at 427 nm (40 W Kessil lamp) at a distance of approximately 4.5 cm for 16 hours. The solvent was then evaporated under reduced pressure. The resulting residue was purified by column chromatography.

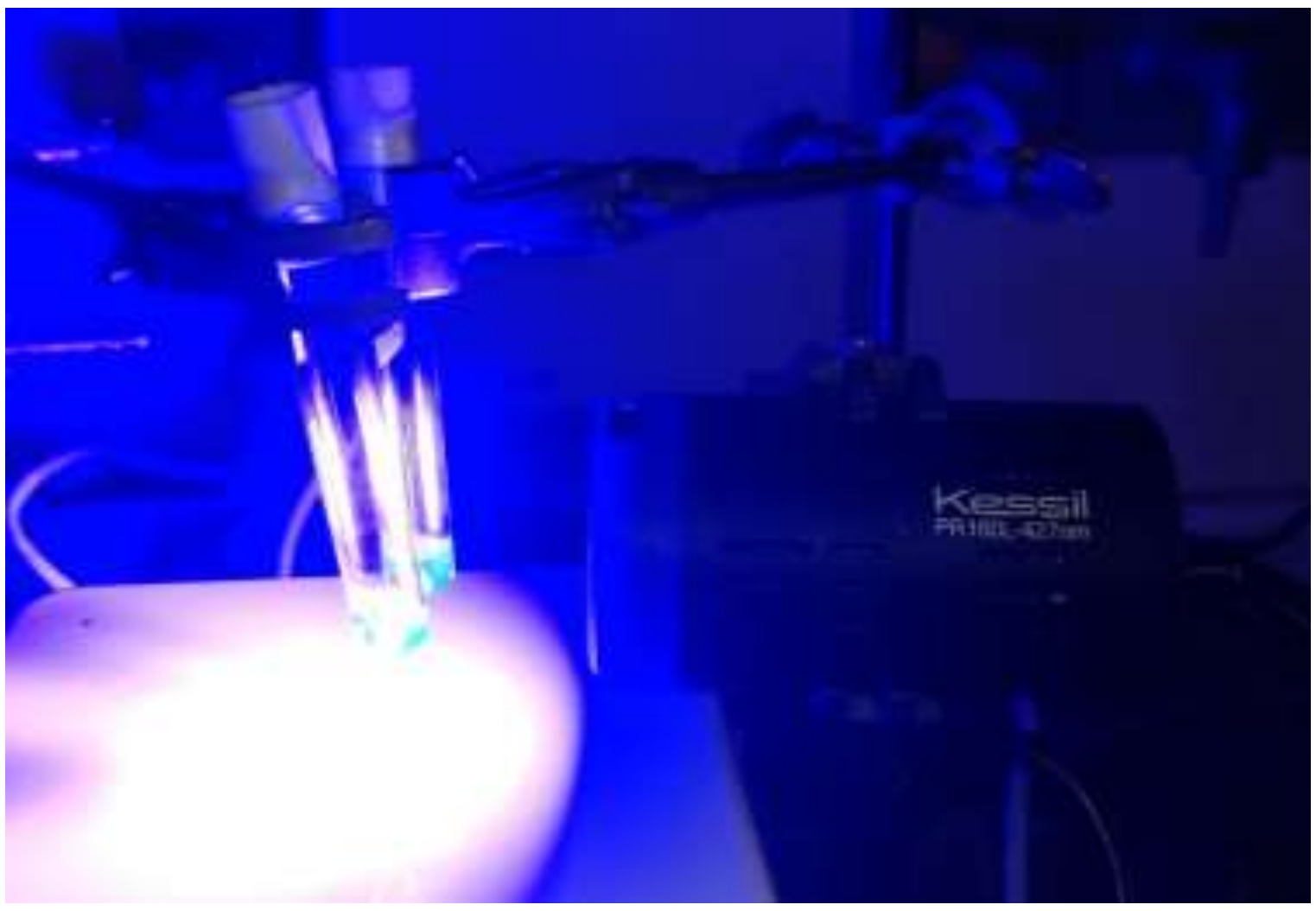

Supplementary Figure S3. Representative experimental setup for the synthesis of ketals.

1,1-Dimethoxycyclohexane (8a) [15]

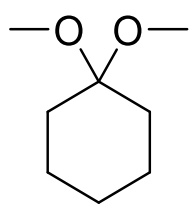

Elution solvent system: petroleum ether:EtOAc 95:5; Method A, yield 74% (53 mg); Colorless oil; ^{1}H-NMR (400 MHz, $CDCl_3$): δ = 3.17 (s, 6H, 2 × OCH_3), 1.65–1.60 (m, 4H, 2 × CH_2), 1.53–1.46 (m, 4H, 2 × CH_2), 1.43–1.36 (m, 2H, CH_2); ^{13}C-NMR (100 MHz, $CDCl_3$): δ = 100.0, 47.3, 32.7, 25.6, 22.8; HRMS (ESI) $[M + Na]^+$ *m/z*: 167.1045; calculated for $[C_8H_{16}NaO_2]^+$ 167.1043.

1,1-Diethoxycyclohexane (8b) [16]

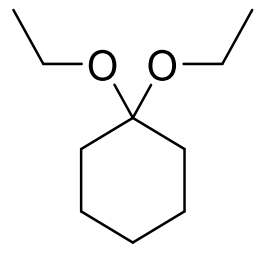

Elution solvent system: petroleum ether:EtOAc 95:5; Method A, yield 36% (31 mg); Colorless oil; ^{1}H-NMR (400 MHz, $CDCl_3$): δ = 3.45 (q, J = 7.1 Hz, 4H, 2 × OCH_2), 1.64 (t, J = 5.8 Hz, 4H, 2 × CH_2), 1.50 (quint, J = 5.8 Hz, 4H, 2 × CH_2), 1.39 (q, J = 5.8 Hz, 2H, CH_2), 1.17 (t, J = 7.1 Hz, 6H, 2 × CH_3); ^{13}C-NMR (100 MHz, $CDCl_3$): δ = 99.9, 54.8, 33.8, 25.7, 23.0, 15.6; HRMS (ESI) $[M + Na]^+$ m/z: 195.1362; calculated for $[C_{10}H_{20}NaO_2]^+$ 195.1356.

1,4-Dioxaspiro[4.5]decane (8c) [17]

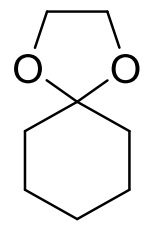

Elution solvent system: petroleum ether:EtOAc 95:5; Method A, yield 69% (49 mg); Colorless oil; ^{1}H-NMR (400 MHz, $CDCl_3$): δ = 3.93 (s, 4H, 2 × OCH_2), 1.62–1.56 (m, 8H, 4 × CH_2), 1.43–1.37 (m, 2H, CH_2); ^{13}C-NMR (100 MHz, $CDCl_3$): δ = 109.0, 64.1, 35.2, 25.1, 24.0; HRMS (ESI) $[M + H]^+$ m/z: 143.1067; calculated for $[C_8H_{15}O_2]^+$ 165.0886.

(1,4-Dioxaspiro[4.5]decan-2-yl)methanol (8d) [18]

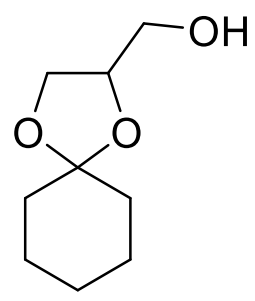

Elution solvent system: petroleum ether:EtOAc 65:35; Method B, yield 99% (85 mg); Colorless oil; ^{1}H-NMR (400 MHz, $CDCl_3$): δ = 4.23–4.16 (m, 1H, OCH), 3.99 (dd, J_1 = 8.3 Hz, J_2 = 6.5 Hz, 1H, OC*H*H), 3.73 (dd, J_1 = 8.3 Hz, J_2 = 6.4 Hz, 1H, OCH*H*), 3.67 (dd, J_1 = 11.6 Hz, J_2 = 4.0 Hz, 1H, OC*H*H), 3.55 (dd, J_1 = 11.6 Hz, J_2 = 5.3 Hz, 1H, OCH*H*), 2.68 (br, 1H, OH), 1.64–1.49 (m, 8H, 4 × CH_2), 1.42–1.29 (m, 2H, CH_2); ^{13}C-NMR (100 MHz, $CDCl_3$): δ = 109.9, 75.7, 65.3, 63.0, 36.3, 34.6, 25.0, 23.9, 23.6; HRMS (ESI) $[M + Na]^+$ m/z: 195.0996; calculated for $[C_6H_{12}NaO_3]^+$ 195.0992.

(2,2-Dimethyl-1,3-dioxolan-4-yl)methanol / Solketal (8e) [19]

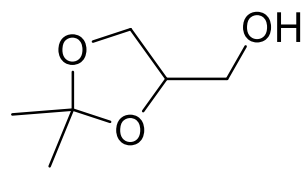

Elution solvent system: petroleum ether:EtOAc 55:45; Method A, yield 33% (22 mg); Method B, yield 91% (60 mg); Colorless oil; ^{1}H-NMR (400 MHz, $CDCl_3$): δ = 4.28–

4.19 (m, 1H, OCH), 4.03 (dd, J_1 = 8.1 Hz, J_2 = 6.6 Hz, 1H, OC*H*H), 3.79 (dd, J_1 = 8.1 Hz, J_2 = 6.6 Hz, 1H, OCH*H*), 3.73 (dd, J_1 = 11.6 Hz, J_2 = 3.6 Hz, 1H, OC*H*H), 3.59 (dd, J_1 = 11.6 Hz, J_2 = 4.9 Hz, 1H, OCH*H*), 1.97 (br, 1H, OH), 1.44 (s, 3H, CH_3), 1.37 (s, 3H, CH_3); ^{13}C-NMR (100 MHz, $CDCl_3$): δ = 109.4, 76.1, 65.6, 63.0, 26.7, 25.2; HRMS (ESI) $[M + Na]^+$ *m/z*: 155.0685; calculated for $[C_6H_{12}NaO_3]^+$ 155.0679.

Ethyl 2-(2-methyl-1,3-dioxolan-2-yl)acetate / Fructone / Applinal (8f) [20]

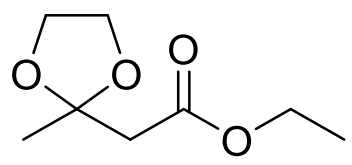

Elution solvent system: petroleum ether:EtOAc 90:10; Method A, yield 73% (64 mg); Colorless oil; ^{1}H-NMR (400 MHz, $CDCl_3$): δ = 4.13 (q, J = 7.1 Hz, 2H, OCH_2), 3.95 (s, 4H, 2 × OCH_2), 2.64 (s, 2H, CH_2CO), 1.48 (s, 3H, CH_3), 1.24 (t, J = 7.1 Hz, 3H, CH_3); ^{13}C-NMR (100 MHz, $CDCl_3$): δ = 169.4, 107.6, 64.7, 60.5, 44.2, 24.4, 14.1; HRMS (ESI) $[M + Na]^+$ *m/z*: 197.0786; calculated for $[C_8H_{14}NaO_4]^+$ 197.0784.

Ethyl 2-(4-(hydroxymethyl)-2-methyl-1,3-dioxolan-2-yl)acetate (8g)

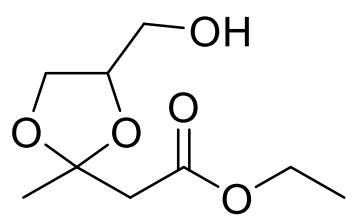

Elution solvent system: petroleum ether:EtOAc 65:35; Method B, yield 96% (98 mg); Two diastereomers (d.r. 55:45); Colorless oil; ^{1}H-NMR (400 MHz, $CDCl_3$): δ = 4.25–4.17 (m, 1H, OCH), 4.14–4.06 (m, 2H, OCH_2), 4.04–3.98 (m, 1H, OC*H*H), 3.89 (dd, J_1 = 8.2 Hz, J_2 = 7.5 Hz, 0.55H, 0.55 × OCH*H*), 3.81–3.74 (m, 1H, 0.45 × CH*H*, 0.55 × OC*H*H), 3.65 (dd, J_1 = 11.8 Hz, J_2 = 7.5 Hz, 0.45H, 0.45 × OC*H*H), 3.55 (dd, J_1 = 11.8 Hz, J_2 = 5.0 Hz, 0.45H, 0.45 × OCH*H*), 3.47 (dd, J_1 = 12.3 Hz, J_2 = 3.3 Hz, 0.55H, 0.55 × OCH*H*), 2.96 (br, 1H, OH), 2.72–2.63 (m, 1.10H, 0.55 × CH_2CO), 2.60 (s, 0.90H, 0.45 × CH_2CO), 1.49 (s, 1.35H, 0.45 × CH_3), 1.40 (s, 1.65H, 0.55 × CH_3), 1.24–1.17 (m, 3H, CH_3); ^{13}C-NMR (100 MHz, $CDCl_3$): δ = 170.0, 169.4, 108.2, 107.8, 76.6, 76.6, 65.9, 65.2, 62.5, 61.4, 60.8, 60.5, 44.3, 43.7, 25.1, 24.6, 14.0, 13.9; HRMS (ESI) $[M + Na]^+$ *m/z*: 227.0892; calculated for $[C_8H_{14}NaO_4]^+$ 227.0890.

Ethyl 2-(2,5,5-trimethyl-1,3-dioxan-2-yl)acetate (8h) [21]

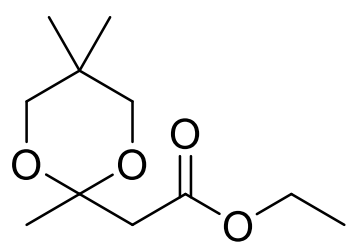

Elution solvent system: petroleum ether:EtOAc 90:10; Method B, yield 48% (52 mg); Colorless oil; ^{1}H-NMR (400 MHz, $CDCl_3$): δ = 4.15 (q, J = 7.1 Hz, 2H, OCH_2), 3.58–3.47 (m, 4H, 2 × OCH_2), 2.77 (s, 2H, CH_2CO), 1.53 (s, 3H, CH_3), 1.25 (t, J = 7.1 Hz, 3H, CH_3), 0.97 (s, 3H, CH_3), 0.94 (s, 3H, CH_3); ^{13}C-NMR (100 MHz, $CDCl_3$): δ =

169.5, 97.3, 70.6, 60.5, 41.6, 29.8, 22.8, 22.6, 22. 5, 14.1; HRMS (ESI) $[M + Na]^+$ *m/z*: 239.1256; calculated for $[C_{11}H_{20}NaO_4]^+$ 239.1254.

Methyl 3-(2-methyl-1,3-dioxolan-2-yl)propanoate (8i) [22]

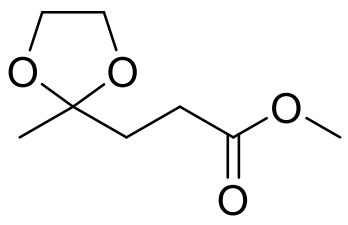

Elution solvent system: petroleum ether:EtOAc 80:20; Method A, yield 56% (49 mg); Colorless oil; ^{1}H-NMR (400 MHz, $CDCl_3$): δ = 3.96–3.87 (m, 4H, 2 × OCH_2), 3.65 (s, 3H, OCH_3), 2.38 (t, J = 7.6 Hz, 2H, CH_2), 2.00 (t, J = 7.6 Hz, 2H, CH_2), 1.30 (s, 3H, CH_3); ^{13}C-NMR (100 MHz, $CDCl_3$): δ = 174.0, 109.1, 64.7, 51.5, 33.9, 28.8, 23.9; HRMS (ESI) $[M + Na]^+$ *m/z*: 197.0784; calculated for $[C_8H_{14}NaO_4]^+$ 197.0784.

Methyl 3-(4-(hydroxymethyl)-2-methyl-1,3-dioxolan-2-yl)propanoate (8j)

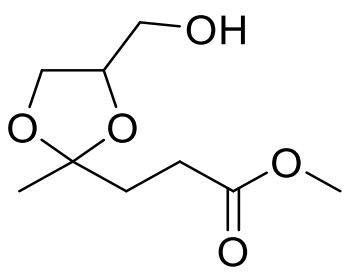

Elution solvent system: petroleum ether:EtOAc 50:50; Method B, yield 86% (88 mg); Two diastereomers (d.r. 55:45); Colorless oil; ^{1}H-NMR (400 MHz, $CDCl_3$): δ = 4.27–4.13 (m, 1H, OCH), 4.04–3.97 (m, 1H, OC*H*H), 3.83–3.68 (m, 2H, OCH*H*, OC*H*H), 3.66 (s, 1.65H, 0.55 × OCH_3), 3.65 (s, 1.35H, 0.45 × OCH_3), 3.59–3.51 (m, 1H, OCH*H*), 2.50–2.28 (m, 3H, CH_2, OH), 2.06 (t, J = 7.0 Hz, 1.10H, 0.55 × CH_2), 1.99 (t, J = 7.6 Hz, 0.90H, 0.45 × CH_2), 1.35 (s, 3H, 0.45 × CH_3), 1.30 (s, 3H, 0.55 × CH_3); ^{13}C-NMR (100 MHz, $CDCl_3$): δ = 174.9, 173.9, 110.0, 109.7, 76.9, 76.3, 65.8, 65.3, 62.7, 62.3, 51.8, 51.6, 34.1, 33.7, 29.0, 28.7, 24.7, 23.6; HRMS (ESI) $[M + Na]^+$ *m/z*: 227.0894; calculated for $[C_9H_{16}NaO_5]^+$ 227.0890.

Methyl 3-(2,5,5-trimethyl-1,3-dioxan-2-yl)propanoate (8k)

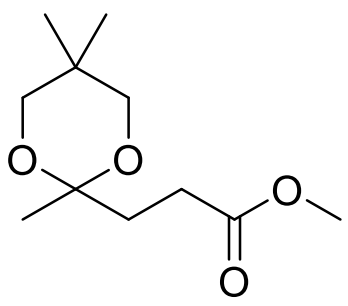

Elution solvent system: petroleum ether:EtOAc 90:10; Method B, yield 71% (77 mg); Colorless oil; ^{1}H-NMR (400 MHz, $CDCl_3$): δ = 3.66 (s, 3H, OCH_3), 3.55 (d, J = 11.5 Hz, 2H, 2 × OC*H*H), 3.40 (d, J = 11.5 Hz, 2H, 2 × OCH*H*), 2.49 (dd, J_1 = 8.6 Hz, J_2 = 7.1 Hz, 2H, 2 × C*H*H), 2.02 (dd, J_1 = 8.6 Hz, J_2 = 7.1 Hz, 2H, 2 × CH*H*), 1.36 (s, 3H, CH_3), 1.02 (s, 3H, CH_3), 0.84 (s, 3H, CH_3); ^{13}C-NMR (100 MHz, $CDCl_3$): δ = 174.3, 98.0, 70.4, 51.5, 33.7, 29.8, 28.2, 22.8, 22.4, 20.0; HRMS (ESI) $[M + Na]^+$ *m/z*: 239.1261; calculated for $[C_8H_{14}NaO_4]^+$ 239.1254.

4,4-Dimethoxytetrahydro-2*H*-pyran (8l) [23]

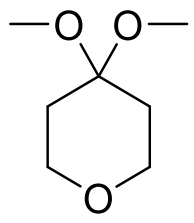

Elution solvent system: petroleum ether:EtOAc 95:5; Method A, yield 56% (41 mg); Colorless oil; ^{1}H-NMR (400 MHz, $CDCl_3$): δ = 3.69-3.67 (m, 4H, 2 × CH_2), 3.20 (s, 6H, 2 × OCH_3), 1.77 (t, J = 5.7 Hz, 4H, 2 × CH_2); ^{13}C-NMR (100 MHz, $CDCl_3$): δ = 97.7, 64.9, 47.4, 33.6; MS (ESI) $[M+ H]^+$ *m/z*: 147.

4,4-Dimethoxytetrahydro-2*H*-thiopyran (8m) [23]

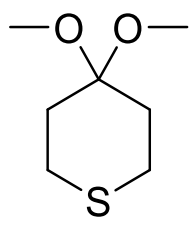

Elution solvent system: petroleum ether:EtOAc 95:5; Method A, yield 63% (50 mg); Colorless oil; ^{1}H-NMR (400 MHz, $CDCl_3$): δ = 3.15 (s, 6H, 2 × OCH_3), 2.65–2.60 (m, 4H, 2 × SCH_2), 1.96–1.92 (m, 4H, 2 × CH_2); ^{13}C-NMR (100 MHz, $CDCl_3$): δ = 98.4, 47.1, 34.3, 25.6; MS (ESI) $[M+ H]^+$ *m/z*: 163.

Large scale synthesis of solketal

Glycerol (**5g**, 1.00 g, 10.86 mmol) and esculetin (**1**, 97 mg, 0.54 mmol) were dissolved in acetone (22 mL). The reaction mixture was left stirring for 22 hours, irradiated by two 427 nm lamps (40 W Kessil lamps). The solvent was then evaporated, and methanol (1–2 mL) was added to the residue. Precipitation of esculetin was achieved after addition of Et_2O (45 mL). The precipitate was filtered and the filtrate was evaporated under reduced pressure to afford pure solketal in 92% yield (1.32 g).

Precipitated esculetin was then recycled and reused in the same reaction. Using the recycled esculetin in consecutive runs, solketal was obtained in the following yields: second run, 89% yield (1.28 g); third run, 84% yield (1.21 g); fourth run, 76% yield (1.09 g); fifth run, 61% yield (0.88 g).

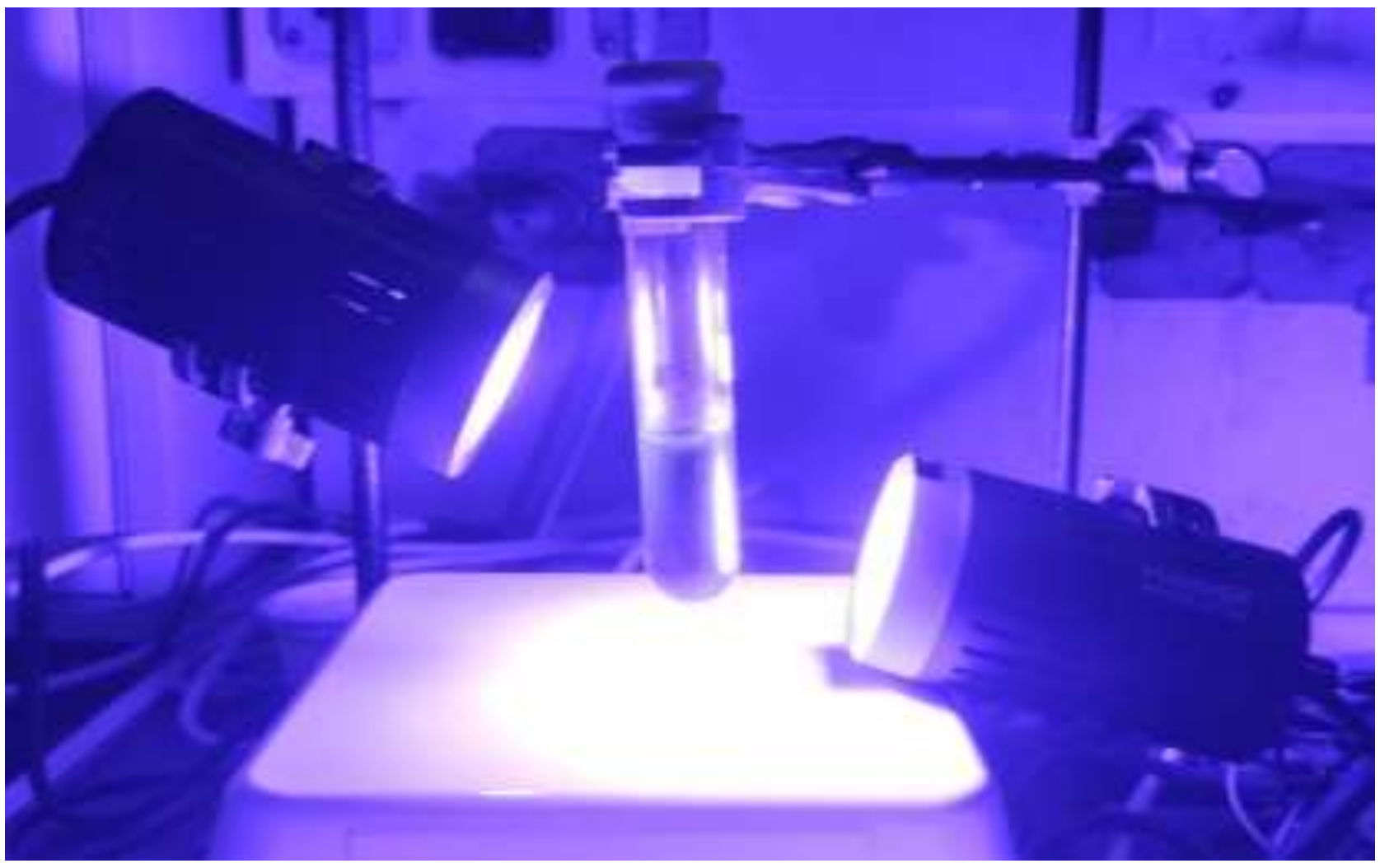

Supplementary Figure S4. Experimental setup for the large scale synthesis of solketal.

Computational Studies

Density functional theory (DFT) calculations were performed to investigate the geometries and energetics of all structures involved in the esculetin-photocatalyzed acetalization of 3-phenylpropanal with methanol. Specifically, all mechanistic steps were computed employing the B3LYP [24]/6-31G(d,p) [25] level of theory, in MeOH solvent, employing the SMD continuum solvation model [26], which is appropriate for evaluating Gibbs free energies ΔG. The UltraFine integration grid was used. The geometries of the S_0, S_1 and T_1 states of all corresponding minima structures were energetically optimized. Initially, conformational analysis was carried out for esculetin in the S_0, S_1 and T_1 states, as well as for the esculetin–3-phenylpropanal hydrogen-bonded complex, at the same level of theory, by setting different initial geometries (Supplementary Figures S5–S9), in order to determine the lowest in energy structures. The chosen B3LYP-6-31G(d,p) methodology is well-established for describing hydrogen-bonding interactions. The frequencies of all optimized structures were computed, to calculate Gibbs free energies (Standard conditions, P = 1atm, T = 298.15K) and verify that the optimized structures correspond to minima (no imaginary frequency) or transition states (Berny Optimization Algorithm, 1 imaginary frequency for **TS** structure). The ΔE_{bind} and ΔG_{bind} energies for the esculetin-3-phenylpropanal complex were calculated via equation (1) and (2)

$$\Delta E_{bind} = E_{complex} - E_{esculetin}{}^{*3} - E_{3\text{-phenylpropanal}} \quad (1)$$

$$\Delta G_{bind} = G_{complex} - G_{esculetin}{}^{*3} - G_{3\text{-phenylpropanal}} + RT\ln(1/22.4) \quad (2)$$

where, the last expression is a correction with regard to the reference state changing from ideal gas to solution. It converts a 1 atm standard state to a more relevant 1 M solution standard state.

The UV-Vis spectrum of esculetin, as well as its S_1 state, were calculated employing the Time-Dependent DFT (TD-DFT) [27], TD-B3LYP/6-31G(d,p). Specifically, the lowest 20 singlet and 20 triplet states were calculated.

All calculations were carried out using the Gaussian16 code, and the geometries were visualized using the GaussView6.0 code [28].

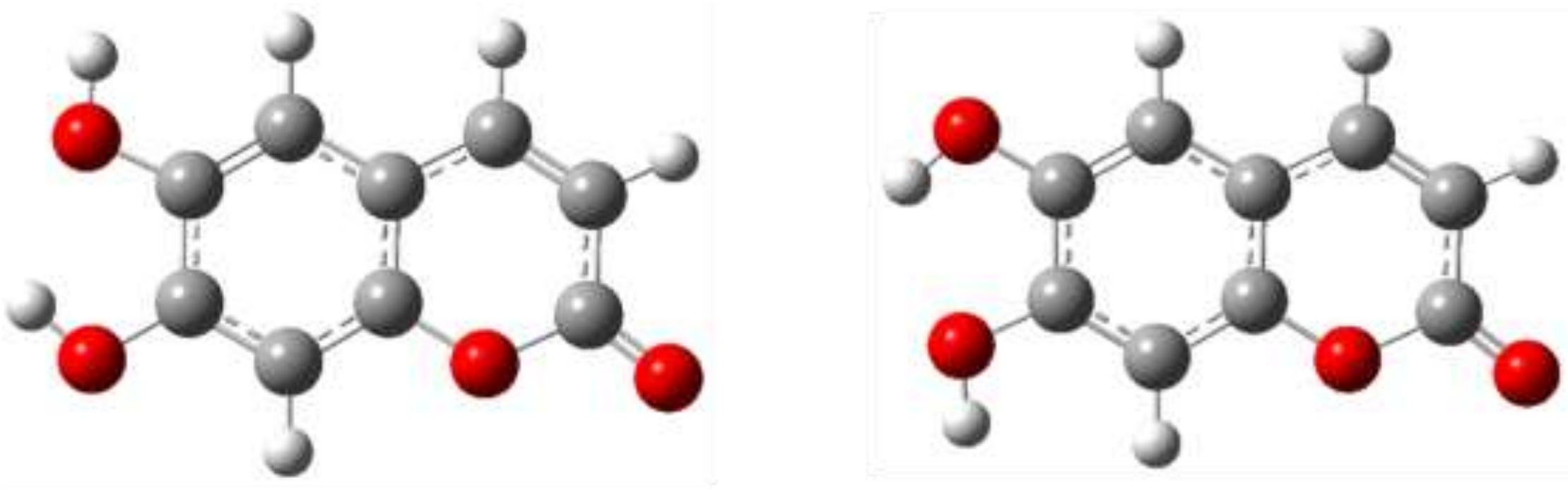

0.0 0.2

Supplementary Figure S5. Relative energy ordering of the two possible conformers (kcal/mol) of esculetin in the S_0 state (B3LYP/6-31G(d,p) methodology, in MeOH, SMD Model).

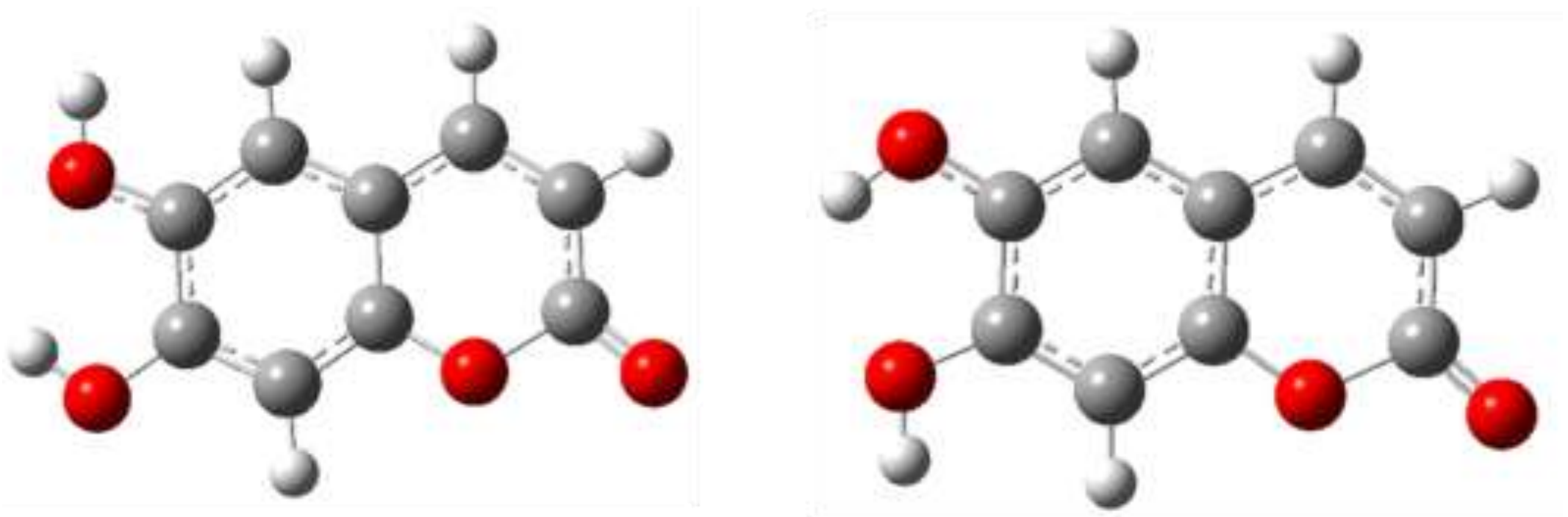

0.6 0.0

Supplementary Figure S6. Relative energy ordering of the two possible conformers (kcal/mol) of esculetin in the S_1 state (B3LYP/6-31G(d,p) methodology, in MeOH, SMD Model).

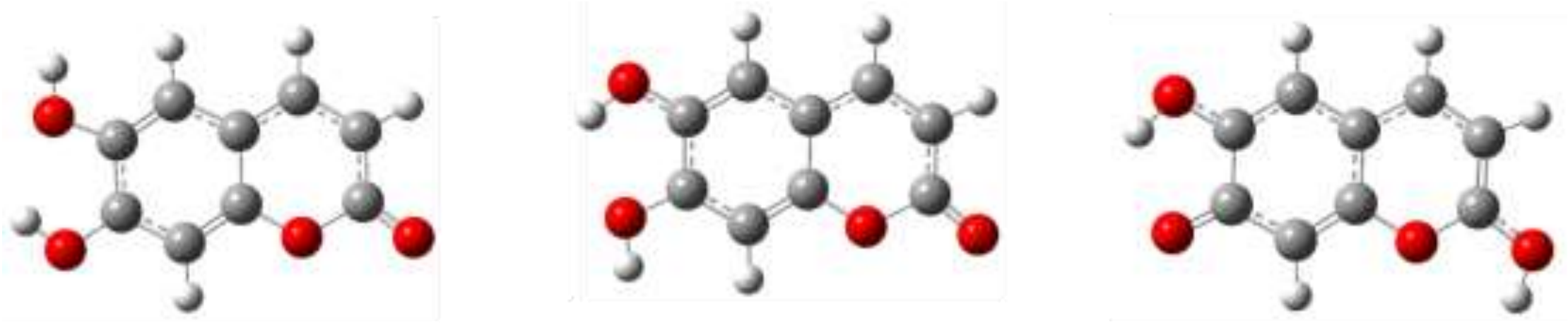

0.2 0.1 0.0

Supplementary Figure S7. Relative energy ordering of the two possible conformers and tautomer (kcal/mol) of esculetin in the T_1 state (B3LYP/6-31G(d,p) methodology, in MeOH, SMD Model).

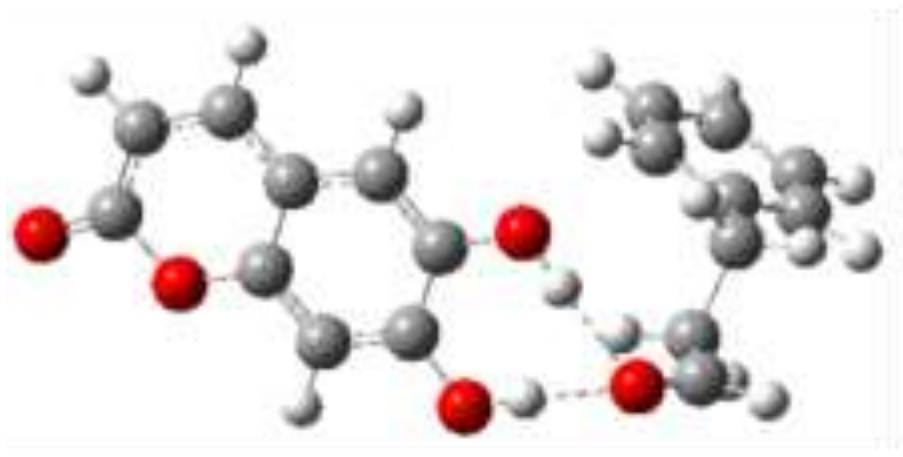

5.9

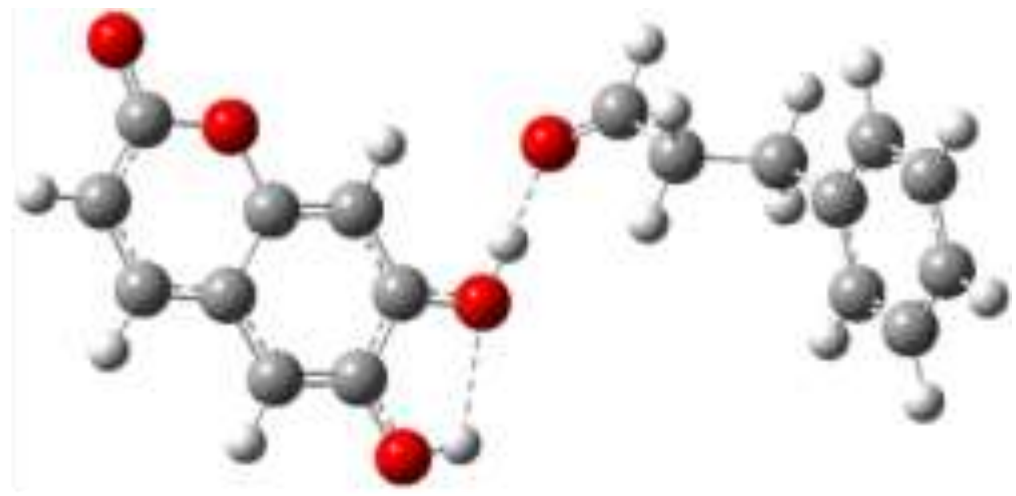

3.5

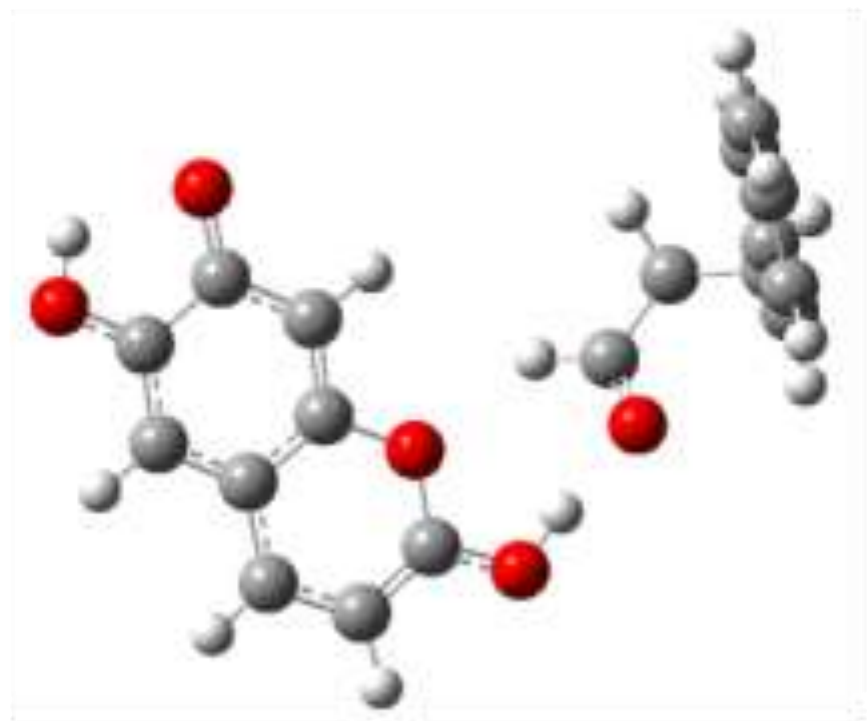

0.0

Supplementary Figure S8. Relative energy ordering of the three possible conformers (kcal/mol) of esculetin(T_1)-3-phenylpropanal complex (B3LYP/6-31G(d,p) methodology, in MeOH, SMD Model).

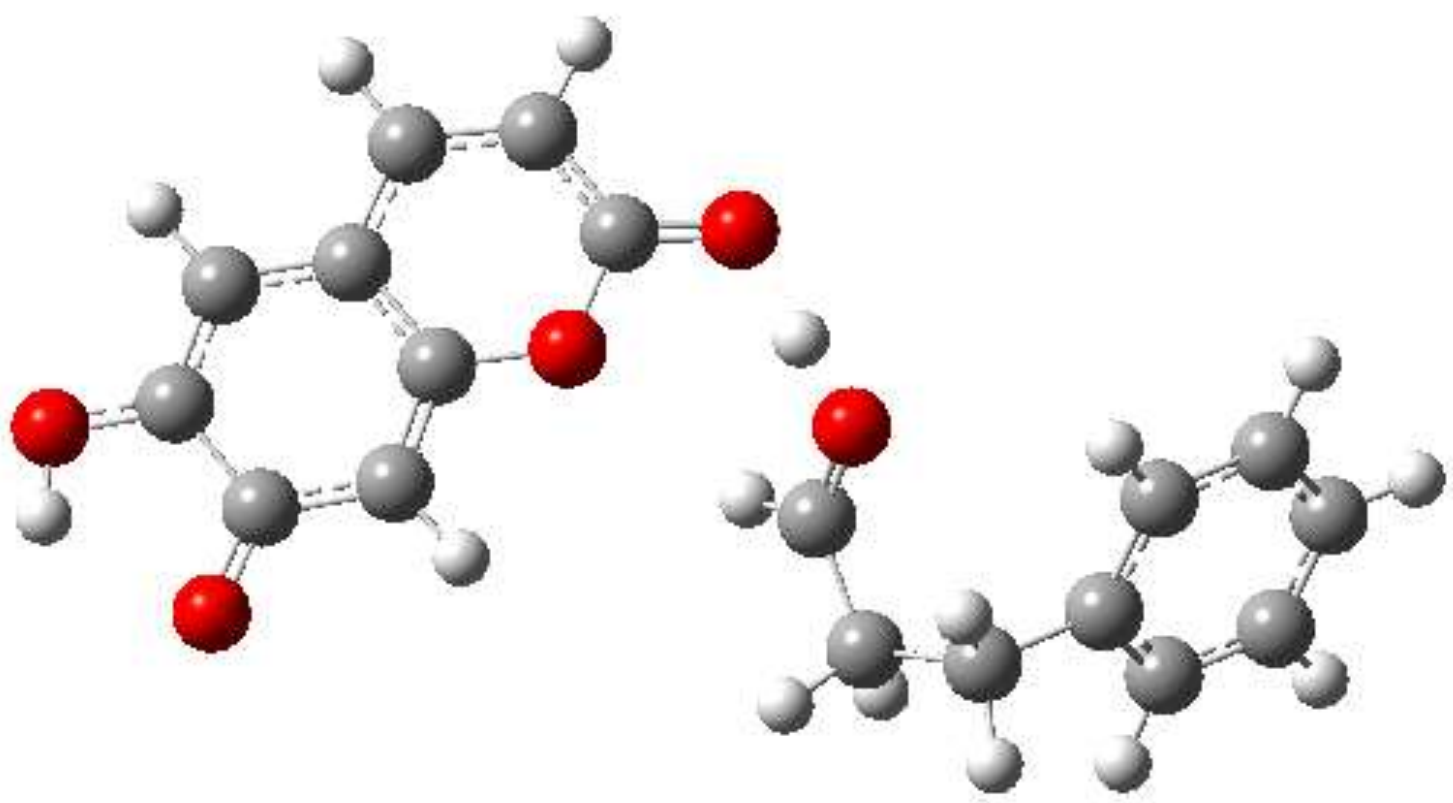

Supplementary Figure S9. Calculated structure of TS1 (**VI**) at the B3LYP/6-31G(d,p) methodology, in MeOH, SMD Model, using the Berny Optimization Algorithm.

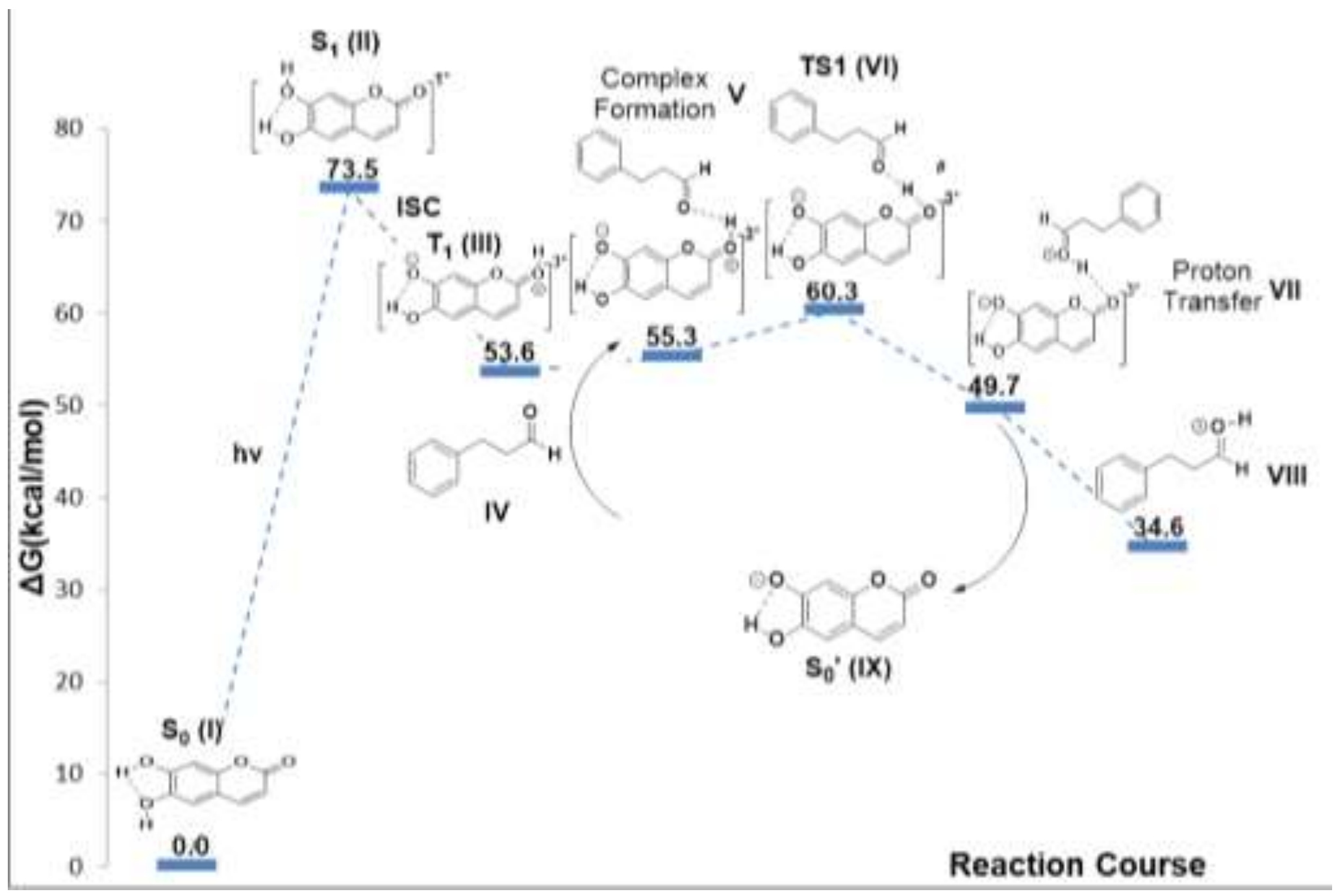

Supplementary Figure S10. Energy profile of the activation of 3-phenylpropanal, using esculetin as the photoacid catalyst. Calculations were carried out at B3LYP/6-31G(d,p) level of theory in MeOH, employing the SMD solvent model.

References

[1] D. Steuernagel, H.-A. Wagenknecht, Photocatalytic synthesis of acetals and ketals from aldehydes and silylenolethers without the use of acids, Chem. Eur. J. 29 (2023) e202203767. https://doi.org/10.1002/chem.202203767.

[2] N. Spiliopoulou, N.F. Nikitas, C.G. Kokotos, Photochemical synthesis of acetals utilizing Schreiner's thiourea as the catalyst, Green Chem. 22 (2020) 3539–3545. https://doi.org/10.1039/D0GC01135E.

[3] Y. Mei, P.A. Bentley, J. Du, NCS with thiourea as highly efficient catalysts for acetalization of aldehydes, Tetrahedron Lett. 50 (2009) 4199–4200. https://doi.org/10.1016/j.tetlet.2009.01.006.

[4] L. Di Terlizzi, E.M. Galathri, S. Protti, C.G. Kokotos, M. Fagnoni, A general metal-free protocol for the visible-light-driven protection of carbonyls, ChemSusChem 16 (2023) e202201998. https://doi.org/10.1002/cssc.202201998.

[5] J. Saway, A.F. Pierre, J.J. Badillo, Photoacid-catalyzed acetalization of carbonyls with alcohols, Org. Biomol. Chem. 20 (2022) 6188–6192. https://doi.org/10.1039/D2OB00435F.

[6] D. Wang, F.-R. Cao, G. Lu, J. Ren, B.-B. Zeng, Practical acetalization and transacetalization of carbonyl compounds catalyzed by recyclable PVP-I, Tetrahedron 92 (2021) 132250. https://doi.org/10.1016/j.tet.2021.132250.

[7] S. Liang, J. Jasinski, G.B. Hammond, B. Xu, Supported gold nanoparticle-catalyzed hydration of alkynes under basic conditions, Org. Lett. 17 (2015) 162–165. https://doi.org/10.1021/ol5033859.

[8] N.F. Nikitas, I. Triandafillidi, C.G. Kokotos, Photo-organocatalytic synthesis of acetals from aldehydes, Green Chem. 21 (2019) 669–674. https://doi.org/10.1039/C8GC03605E.

[9] Q. Zhou, T. Jia, X.-X. Li, L. Zhou, C.-J. Li, Y.-S. Feng, House bulb light-induced photochemical acetalization of carbonyl compounds catalyzed by Eosin Y, Synth. Commun. 48 (2018) 1068–1075. https://doi.org/10.1080/00397911.2018.1434545.

[10] X. Ding, D.A. Devalankar, P. Wang, Structurally simple benzylidene-type photolabile diol protecting groups, Org. Lett. 18 (2016) 5396–5399. https://doi.org/10.1021/acs.orglett.6b02777.

[11] M.R. Cramarossa, L. Forti, F. Ghelfi, Acetals by AlFe-pillared montmorillonite catalysis, Tetrahedron 53 (1997) 15889–15894. https://doi.org/10.1016/S0040-4020(97)10049-7.

[12] S. Hashizume, K. Oisaki, M. Kanai, Functional-group-tolerant catalytic migratory oxidative coupling of nitrones, Chem. Asian J. 7 (2012) 2600–2606. https://doi.org/10.1002/asia.201200359.

[13] R.Yu. Peshkov, C. Wang, E.V. Panteleeva, E.V. Tretyakov, V.D. Shteingarts, Synthesis of 4-(ω-X-alkyl)benzonitriles (X = 1,3-dioxan-2-yl, CN, CO2Et) by the reaction of terephthalonitrile dianion with ω-X-alkyl bromides in liquid ammonia, Russ. Chem. Bull. 65 (2016) 2430–2436. https://doi.org/10.1007/s11172-016-1602-x.

[14] D. Arnodo, C. Meazzo, S. Baldino, M. Blangetti, C. Prandi, Efficient and low-impact acetalization reactions in deep eutectic solvents, Chem. Eur. J. 29 (2023) e202300820. https://doi.org/10.1002/chem.202300820.

[15] M. Baxter, Y. Bolshan, A general access to propargylic ethers through Brønsted acid catalyzed alkynylation of acetals and ketals with trifluoroborates, Chem. Eur. J. 21 (2015) 13535–13538. https://doi.org/10.1002/chem.201502797.

[16] P.J. Tortajada, T. Kärnman, P. Martínez-Pardo, C. Nilsson, H. Holmquist, M.J. Johansson, B. Martín-Matute, Electrochemical hydrogenation of alkenes over a nickel foam guided by life cycle, safety and toxicological assessments, Green Chem. 27 (2024) 227–239. https://doi.org/10.1039/D4GC02924K.
[17] T.A. To, T.V. Nguyen, Olefination of aromatic carbonyls via site-specific activation of cycloalkanone ketals, Angew. Chem. Int. Ed. 63 (2024) e202317003. https://doi.org/10.1002/anie.202317003.
[18] M.J. da Silva, A.A. Julio, F.C.S. Dorigetto, Solvent-free heteropolyacid-catalyzed glycerol ketalization at room temperature, RSC Adv. 5 (2015) 44499–44506. https://doi.org/10.1039/C4RA17090C.
[19] J.J. Chang, M. Kumar, M. Okamoto, E.S. Vergara-Pimentel, D.K. Kim, Unified hydrogen atom transfer approach to construct vicinal functionality, Org. Lett. 27 (2025) 4417–4422. https://doi.org/10.1021/acs.orglett.4c04767.
[20] W. Wang, S. Zhou, W. Jiang, G. Chen, Design, synthesis and anti-rheumatoid arthritis activity of target TLR4 inhibitors, Bioorg. Med. Chem. 97 (2024) 117539. https://doi.org/10.1016/j.bmc.2023.117539.
[21] A. Hou, L. Lauterbach, J.S. Dickschat, Enzymatic synthesis of methylated terpene analogues using the plasticity of bacterial terpene synthases, Chem. Eur. J. 26 (2020) 2178–2182. https://doi.org/10.1002/chem.201905827.
[22] S. Radomkit, J.A.H. White, E. Chong, Y. Zhang, MSTFA as an effective TMS source for the TMSOTf-catalzyed synthesis of cyclic acetals, J. Org. Chem. 89 (2024) 5555–5559. https://doi.org/10.1021/acs.joc.4c00020.
[23] T. Sarkar, S. Keerthana, R.S. Reyno, G. Rajendar, Dimethyl(alkyl) sulfates as Lewis acid type catalysts for acetalization and related reactions of carbonyl compounds, RSC Adv. 15 (2025) 34746–34753. https://doi.org/10.1039/D5RA06610G.
[24] A.D. Becke, Density-functional thermochemistry. III. The role of exact exchange, J. Chem. Phys. 98 (1993) 5648–5652. https://doi.org/10.1063/1.464913.
[25] R. Ditchfield, W.J. Hehre, J.A. Pople, Self-consistent molecular-orbital methods. IX. An extended Gaussian-type basis for molecular-orbital studies of organic molecules, J. Chem. Phys. 54 (1971) 724–728. https://doi.org/10.1063/1.1674902.
[26] A.V. Marenich, C.J. Cramer, D.G. Truhlar, Universal solvation model based on solute electron density and on a continuum model of the solvent defined by the bulk dielectric constant and atomic surface tensions, J. Phys. Chem. B 113 (2009) 6378–6396. https://doi.org/10.1021/jp810292n.
[27] J. Schirmer, Review of the foundations of time-dependent density-functional theory (TDDFT), Phys. Chem. Chem. Phys. 27 (2025) 4992–5005. https://doi.org/10.1039/D4CP04551C.
[28] M.J. Frisch, G.W. Trucks, H.B. Schlegel, G.E. Scuseria, M.A. Robb, J.R. Cheeseman, G. Scalmani, V. Barone, G.A. Petersson, H. Nakatsuji, X. Li, M. Caricato, A.V. Marenich, J. Bloino, B.G. Janesko, R. Gomperts, B. Mennucci, H.P. Hratchian, J.V. Ortiz, A.F. Izmaylov, J.L. Sonnenberg, D. Williams-Young, F. Ding, F. Lipparini, F. Egidi, J. Goings, B. Peng, A. Petrone, T. Henderson, D. Ranasinghe, V.G. Zakrzewski, J. Gao, N. Rega, G. Zheng, W. Liang, M. Hada, M. Ehara, K. Toyota, R. Fukuda, J. Hasegawa, M. Ishida, T. Nakajima, Y. Honda, O. Kitao, H. Nakai, T. Vreven, K. Throssell, J.A. Montgomery, J.E. Peralta, F. Ogliaro, M.J. Bearpark, J.J. Heyd, E.N. Brothers, K.N. Kudin, V.N. Staroverov, T.A. Keith, R. Kobayashi, J. Normand, K. Raghavachari, A.P. Rendell, J.C. Burant, S.S. Iyengar, J. Tomasi, M. Cossi, J.M. Millam, M. Klene, C. Adamo, R.

Cammi, J.W. Ochterski, R.L. Martin, K. Morokuma, O. Farkas, J.B. Foresman, D.J. Fox, Gaussian 16, Revision A.03, (2016).

NMR Spectra

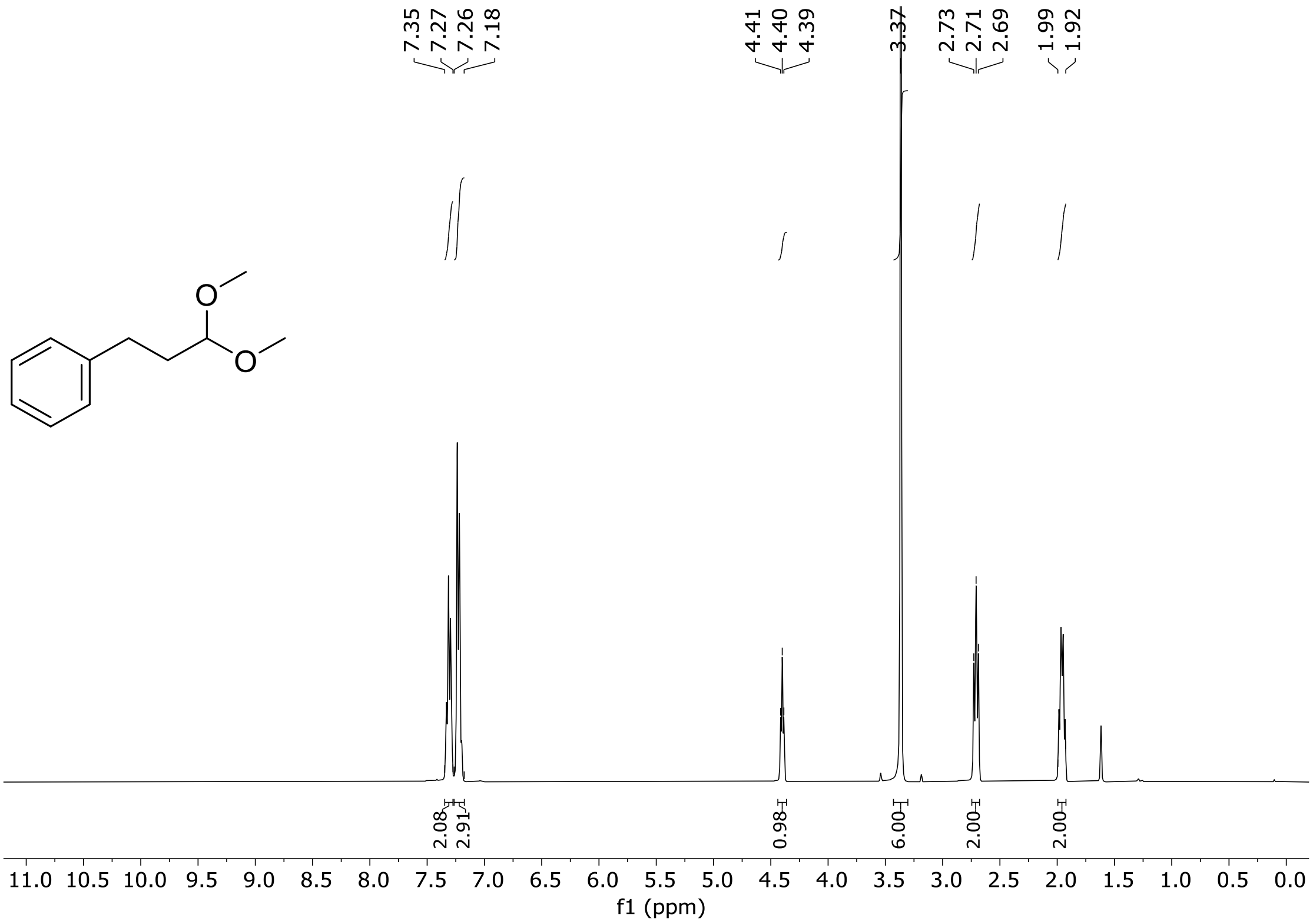

^{1}H-NMR of compound **4a** ($CDCl_3$, 400 MHz).

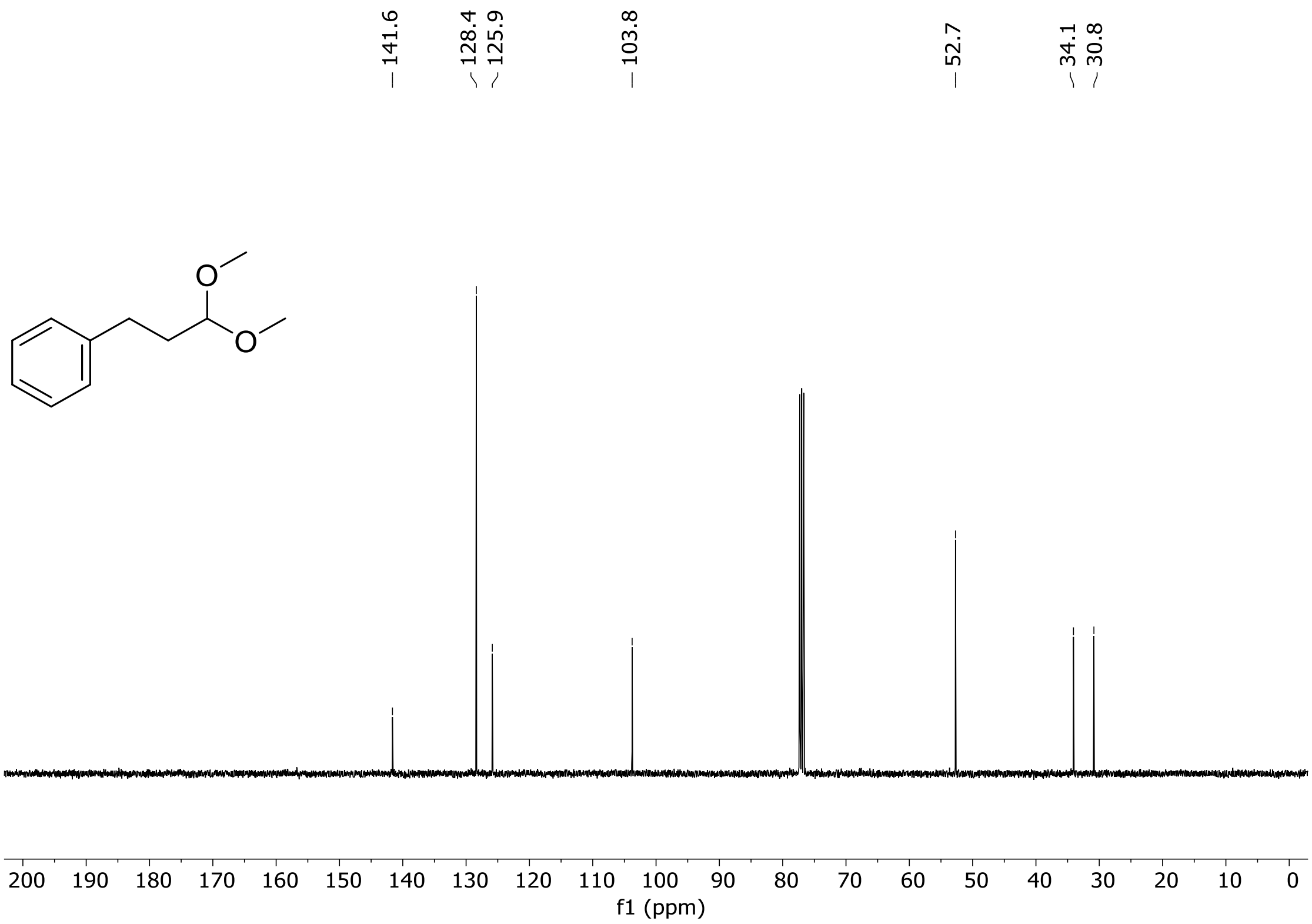

^{13}C-NMR of compound **4a** ($CDCl_3$, 100 MHz).

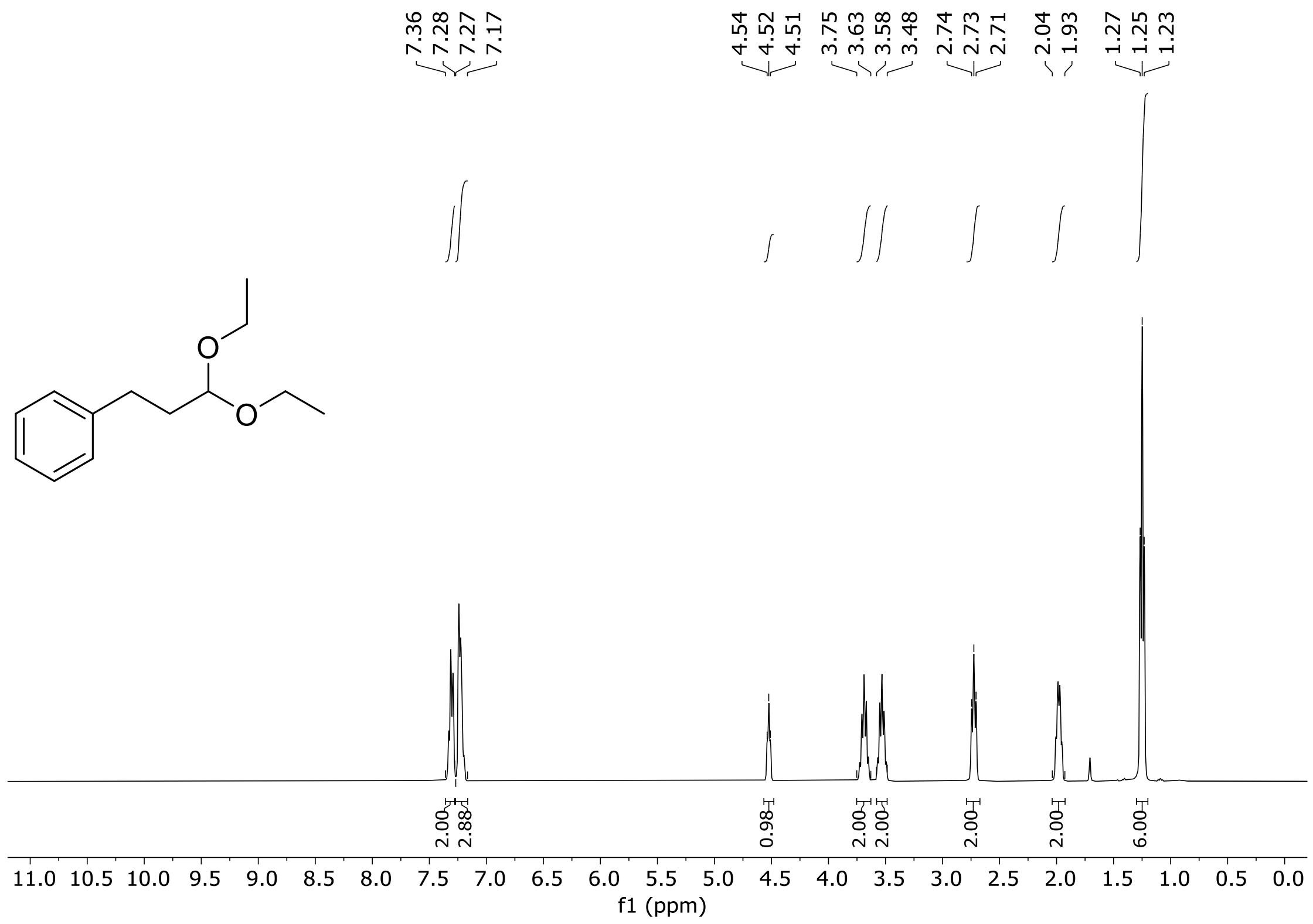

^{1}H-NMR of compound **4b** ($CDCl_3$, 400 MHz).

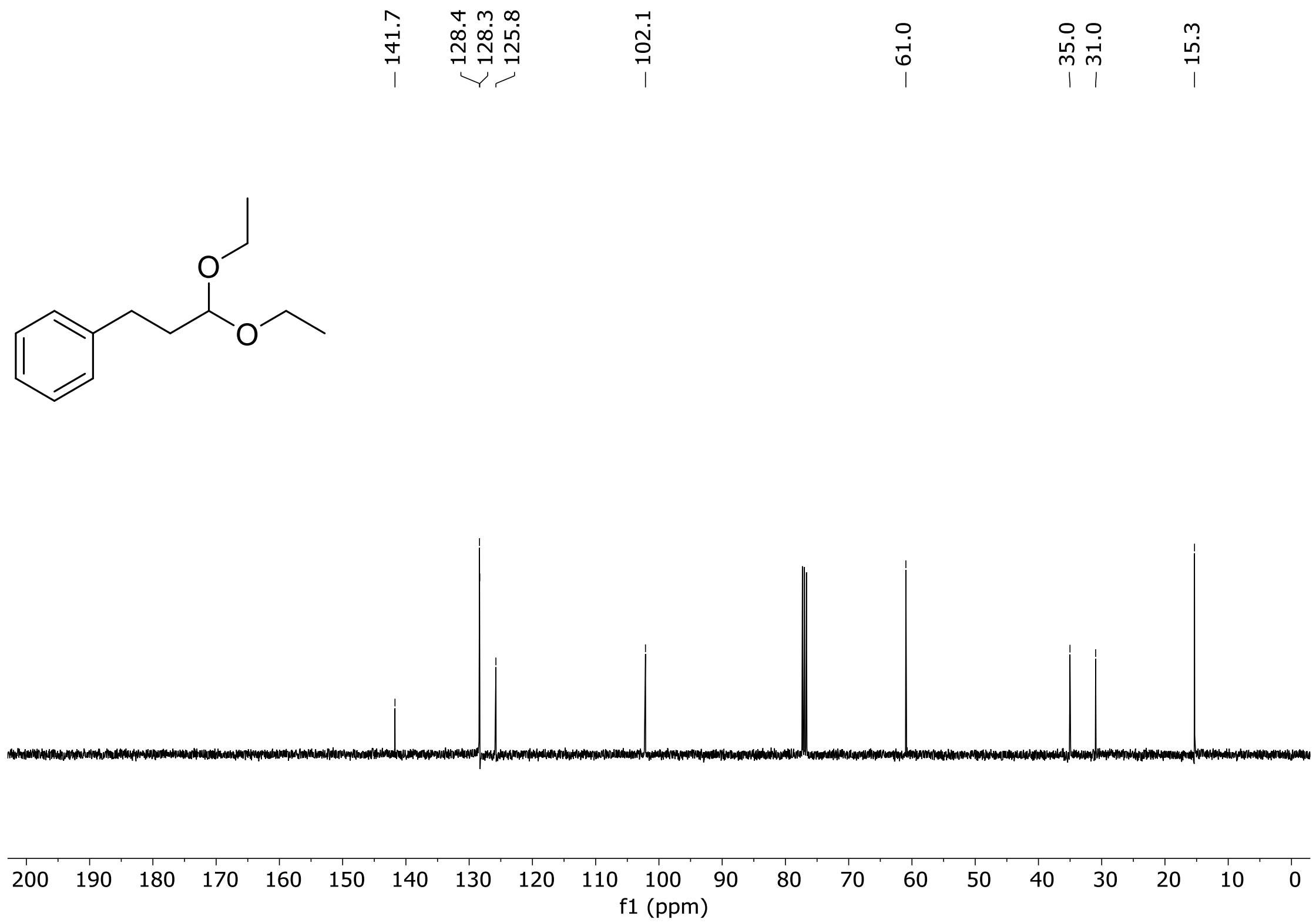

^{13}C-NMR of compound **4b** ($CDCl_3$, 100 MHz).

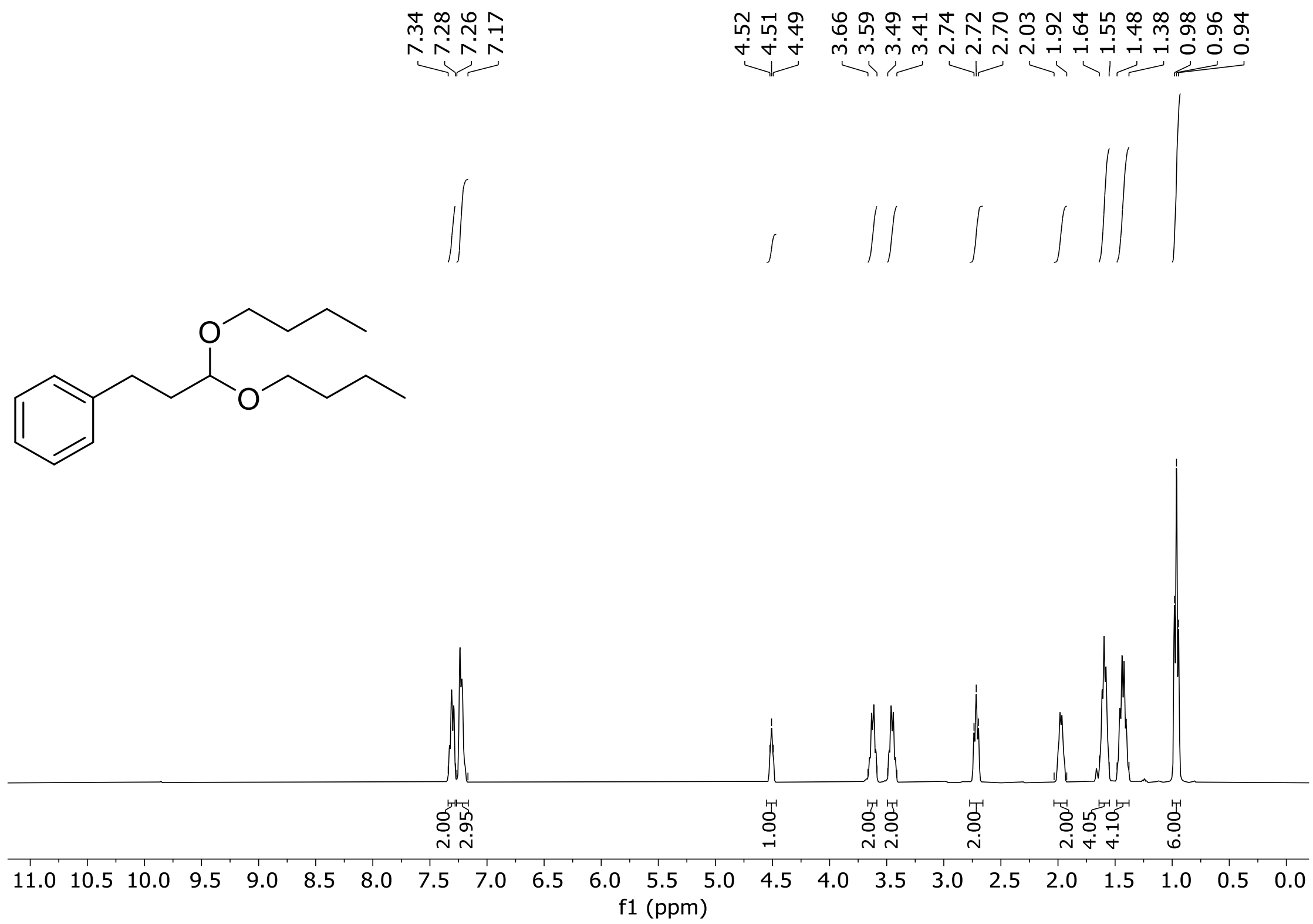

^{1}H-NMR of compound **4c** ($CDCl_3$, 400 MHz).

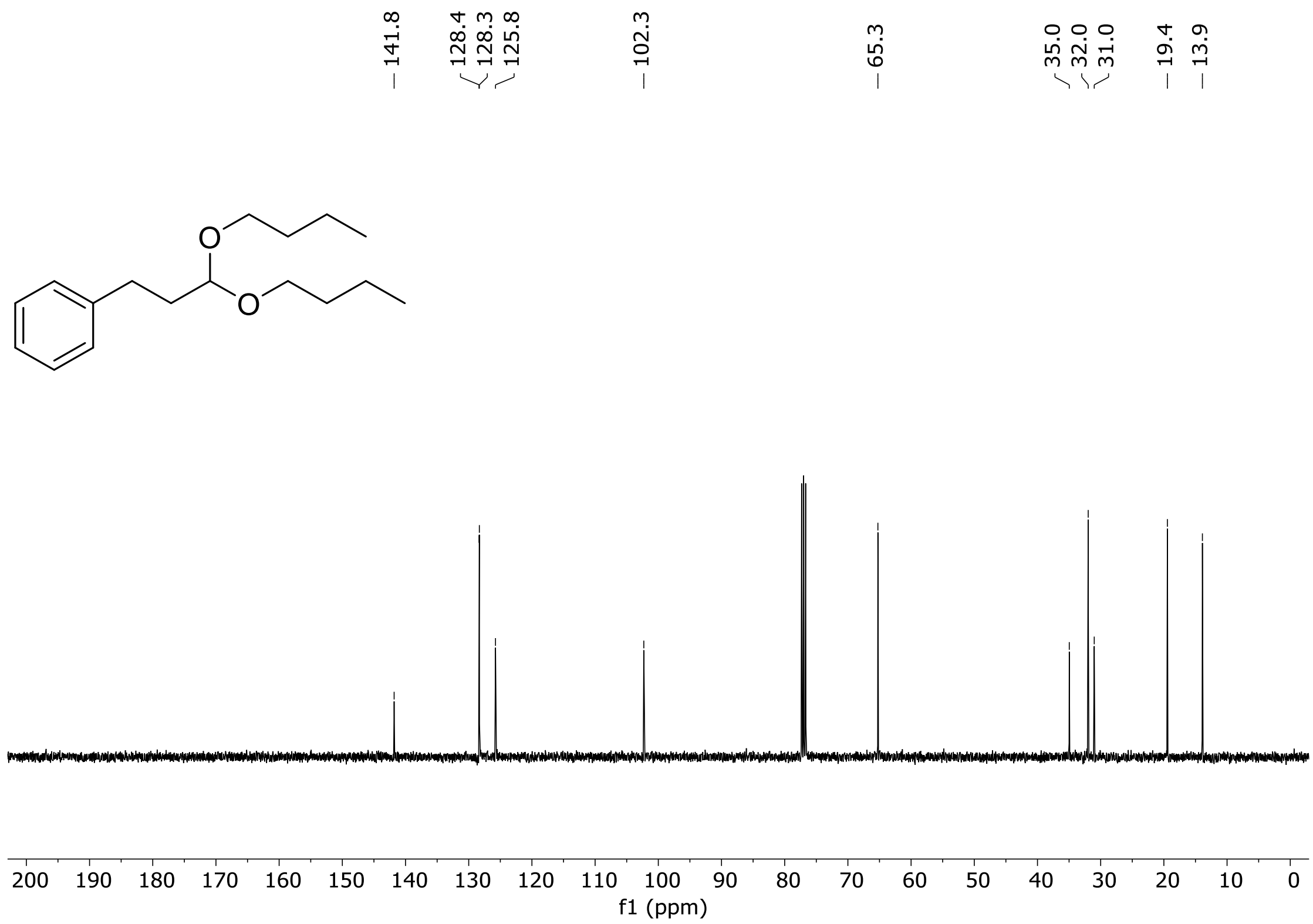

^{13}C-NMR of compound **4c** ($CDCl_3$, 100 MHz).

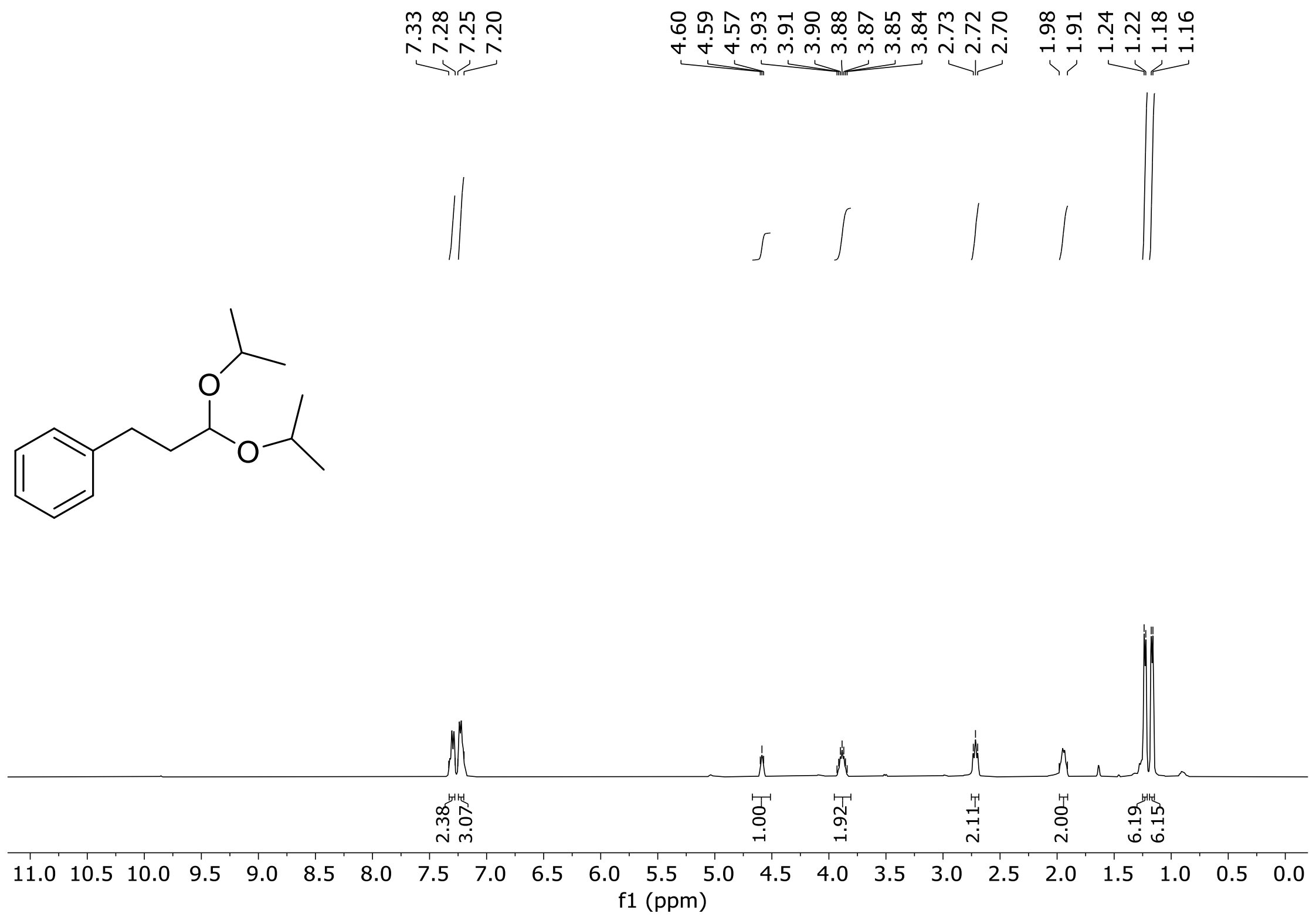

^{1}H-NMR of compound **4d** ($CDCl_3$, 400 MHz).

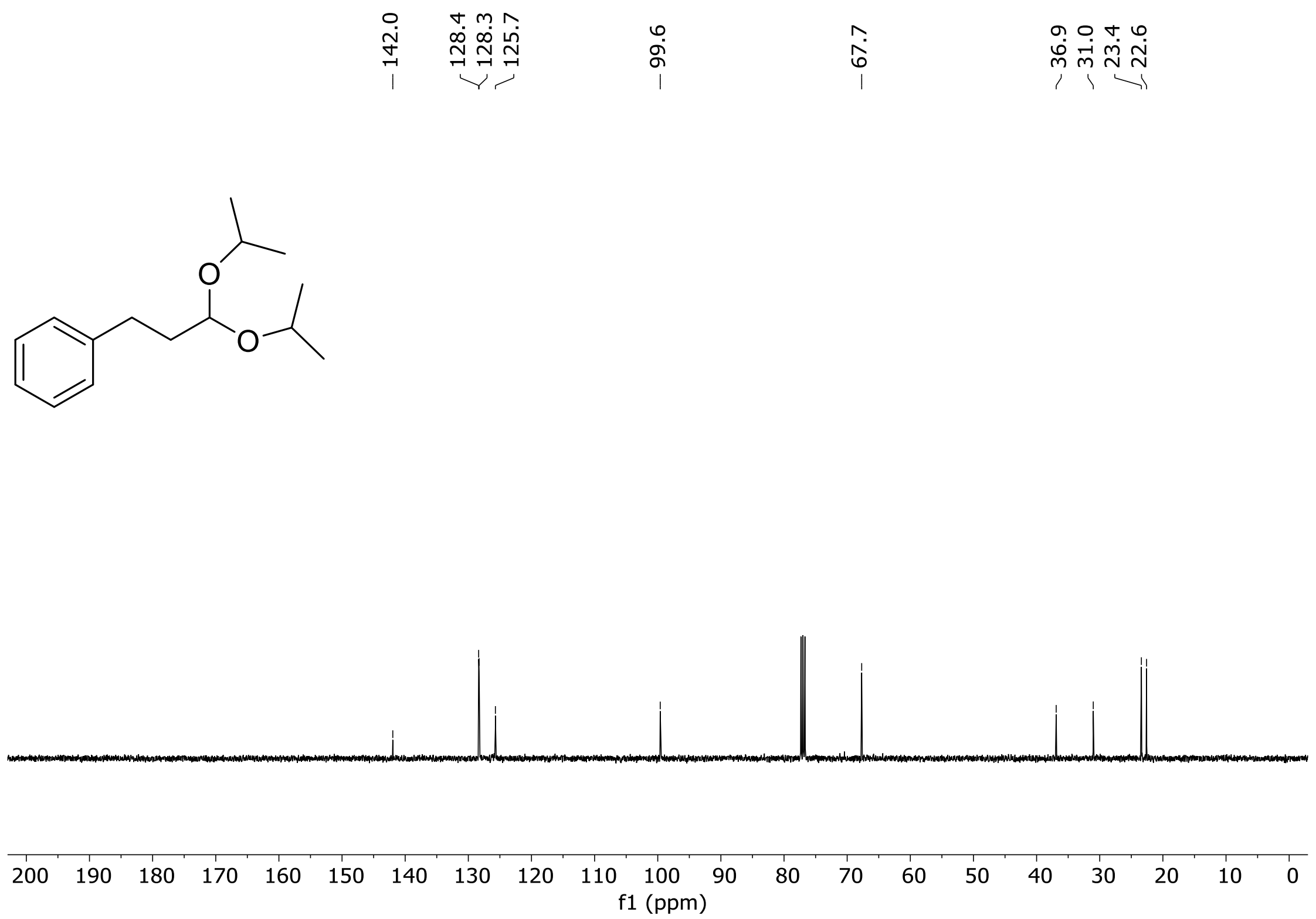

^{13}C-NMR of compound **4d** ($CDCl_3$, 100 MHz).

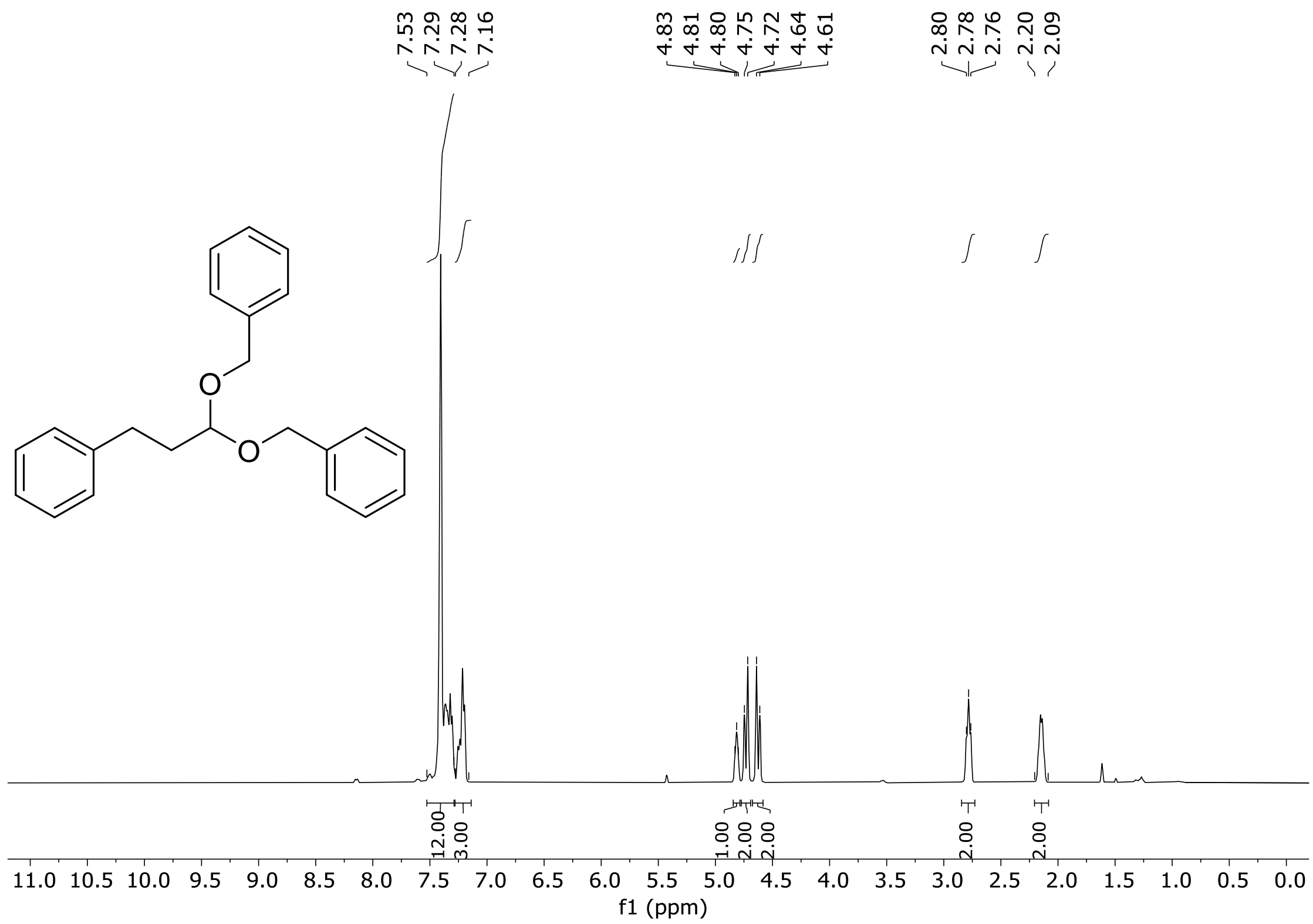

^{1}H-NMR of compound **4e** ($CDCl_3$, 400 MHz).

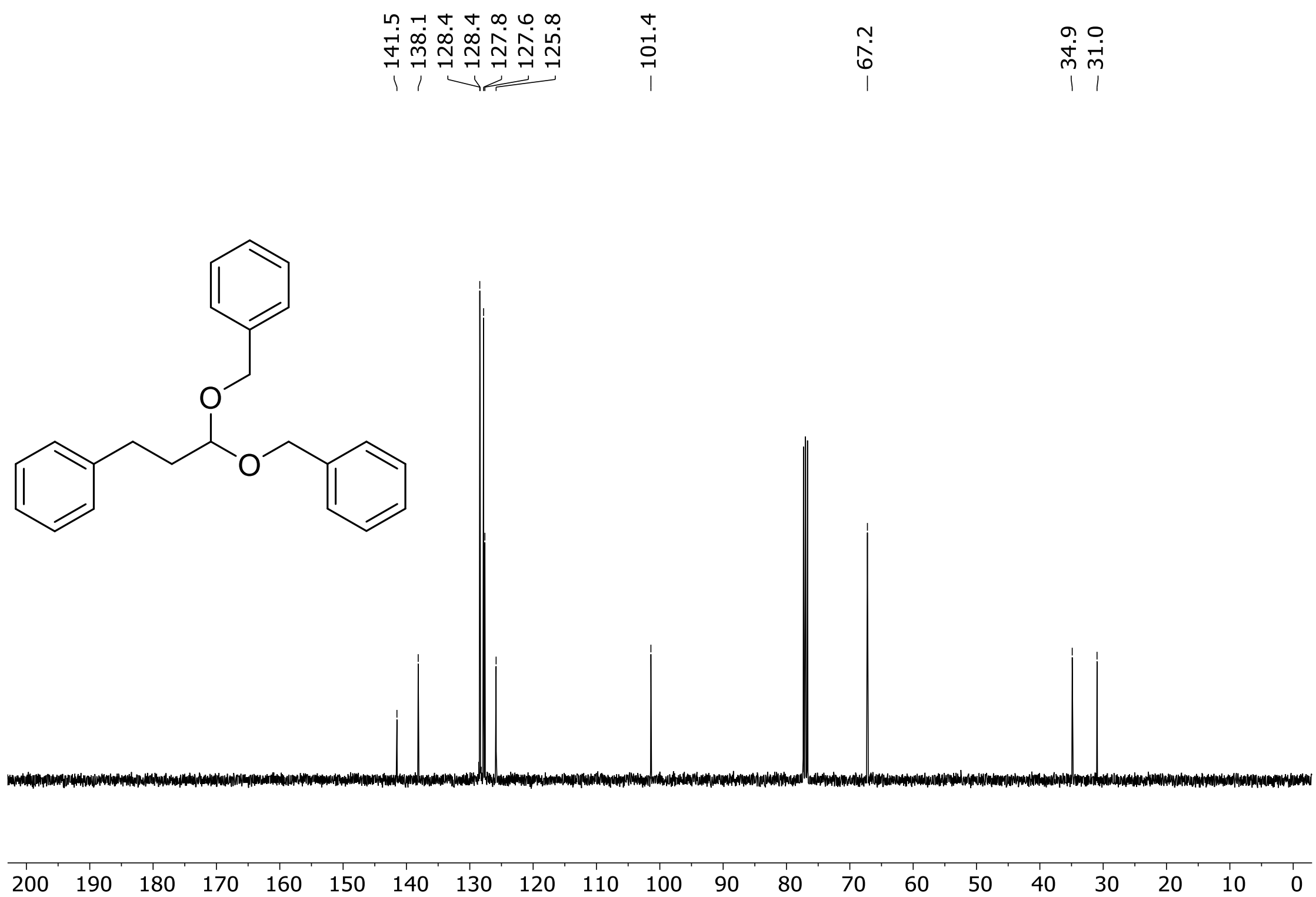

^{13}C-NMR of compound **4e** ($CDCl_3$, 100 MHz).

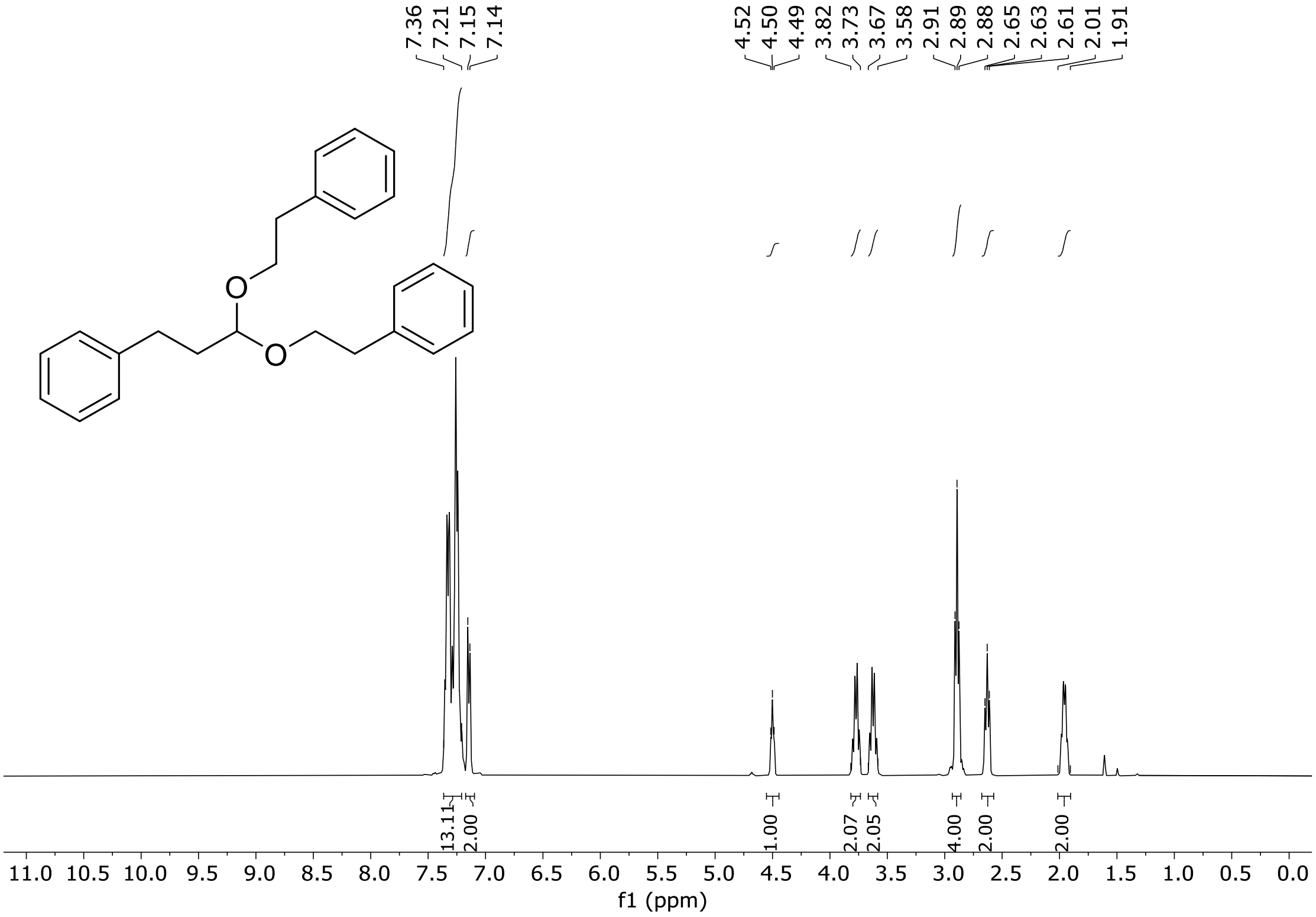

^{1}H-NMR of compound **4f** ($CDCl_3$, 400 MHz).

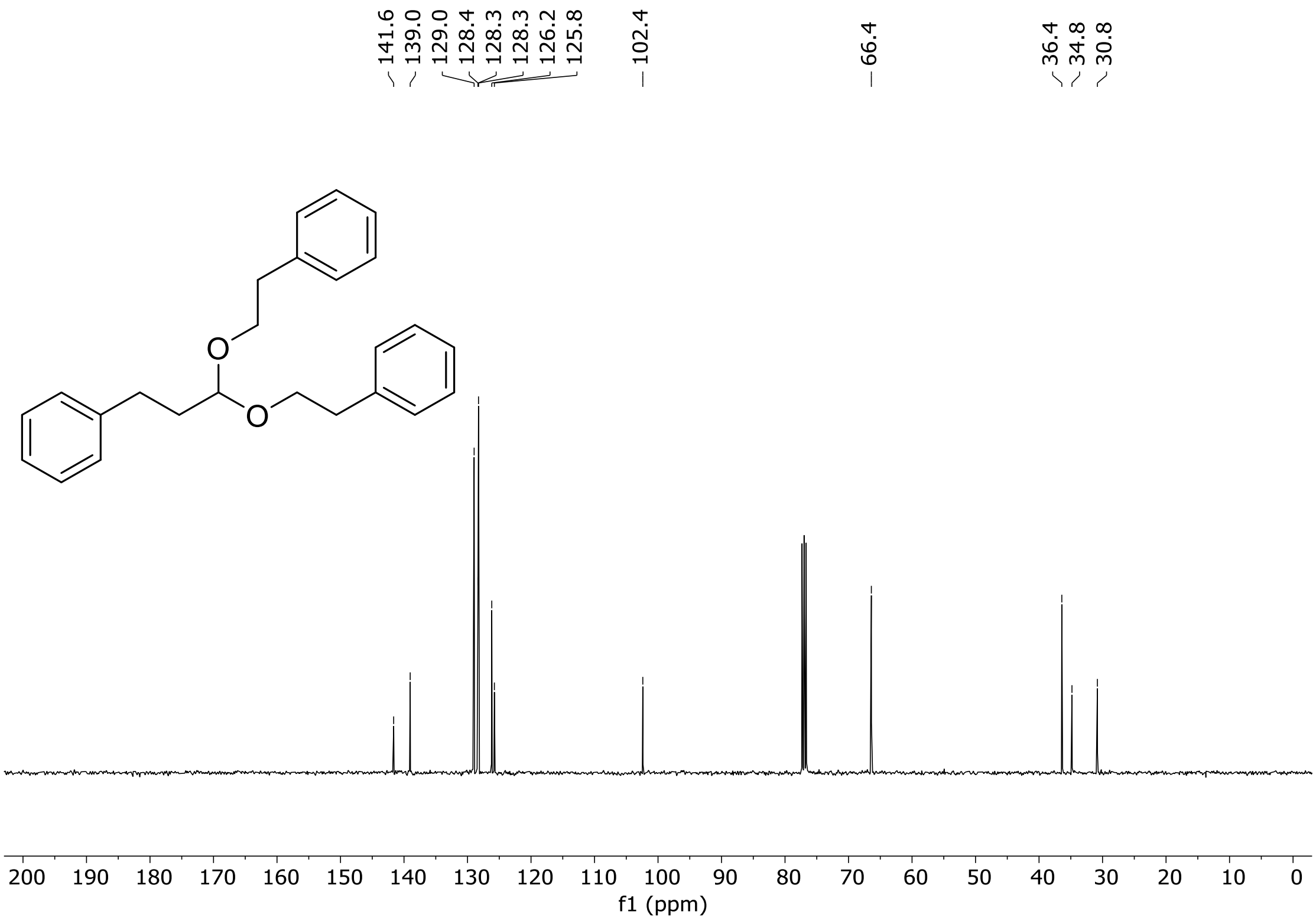

^{13}C-NMR of compound **4f** ($CDCl_3$, 100 MHz).

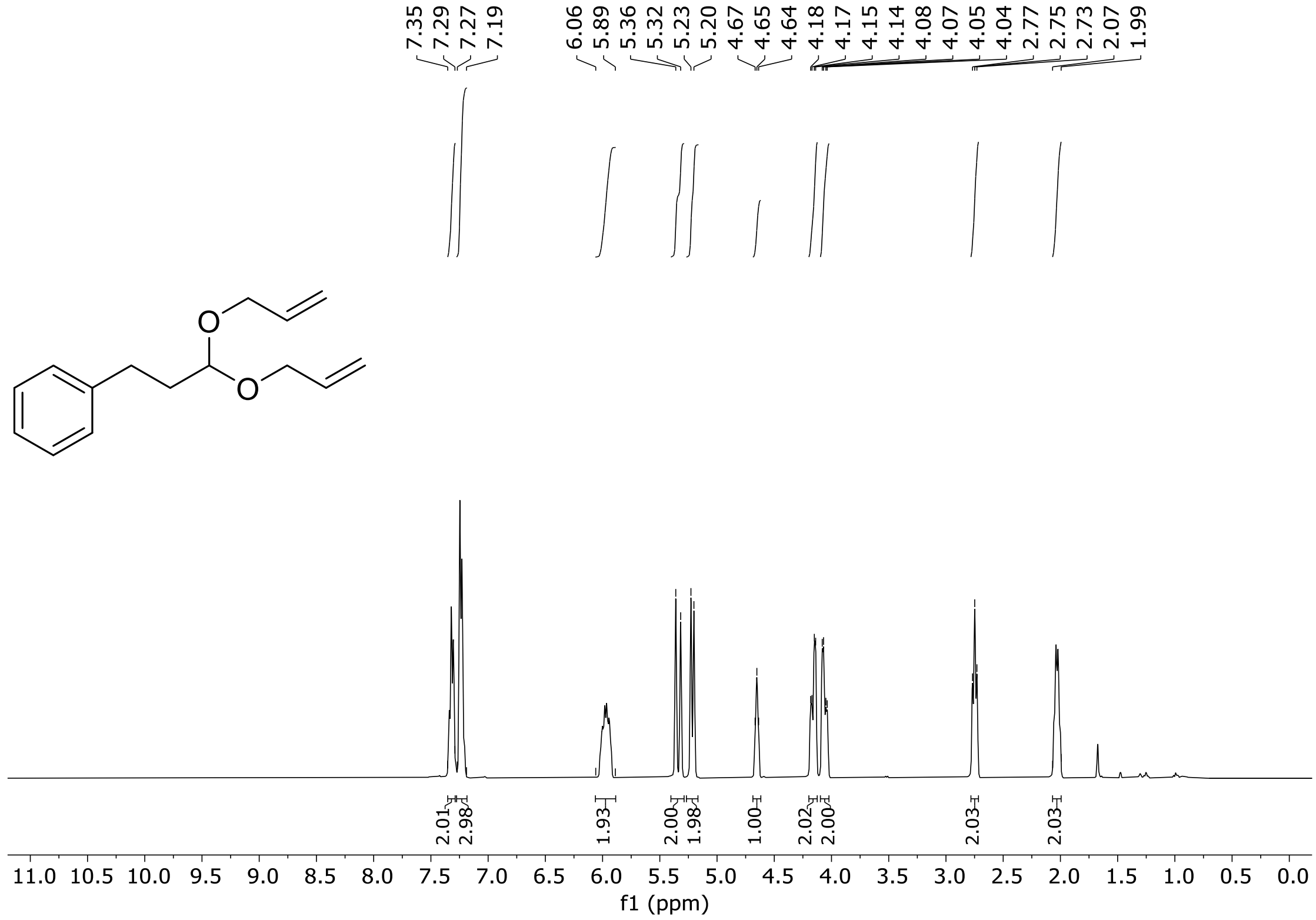

^{1}H-NMR of compound **4g** ($CDCl_3$, 400 MHz).

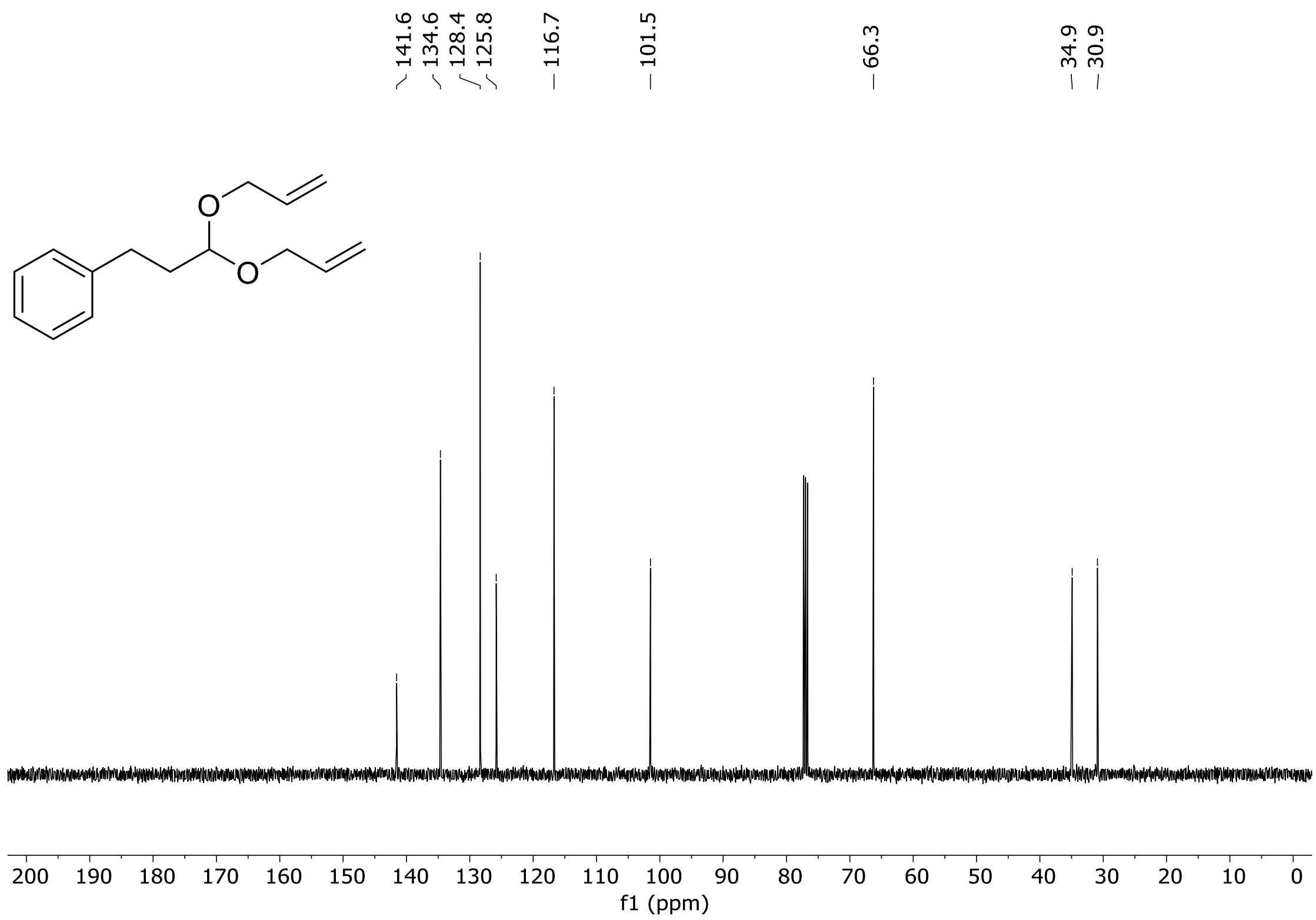

^{13}C-NMR of compound **4g** ($CDCl_3$, 100 MHz).

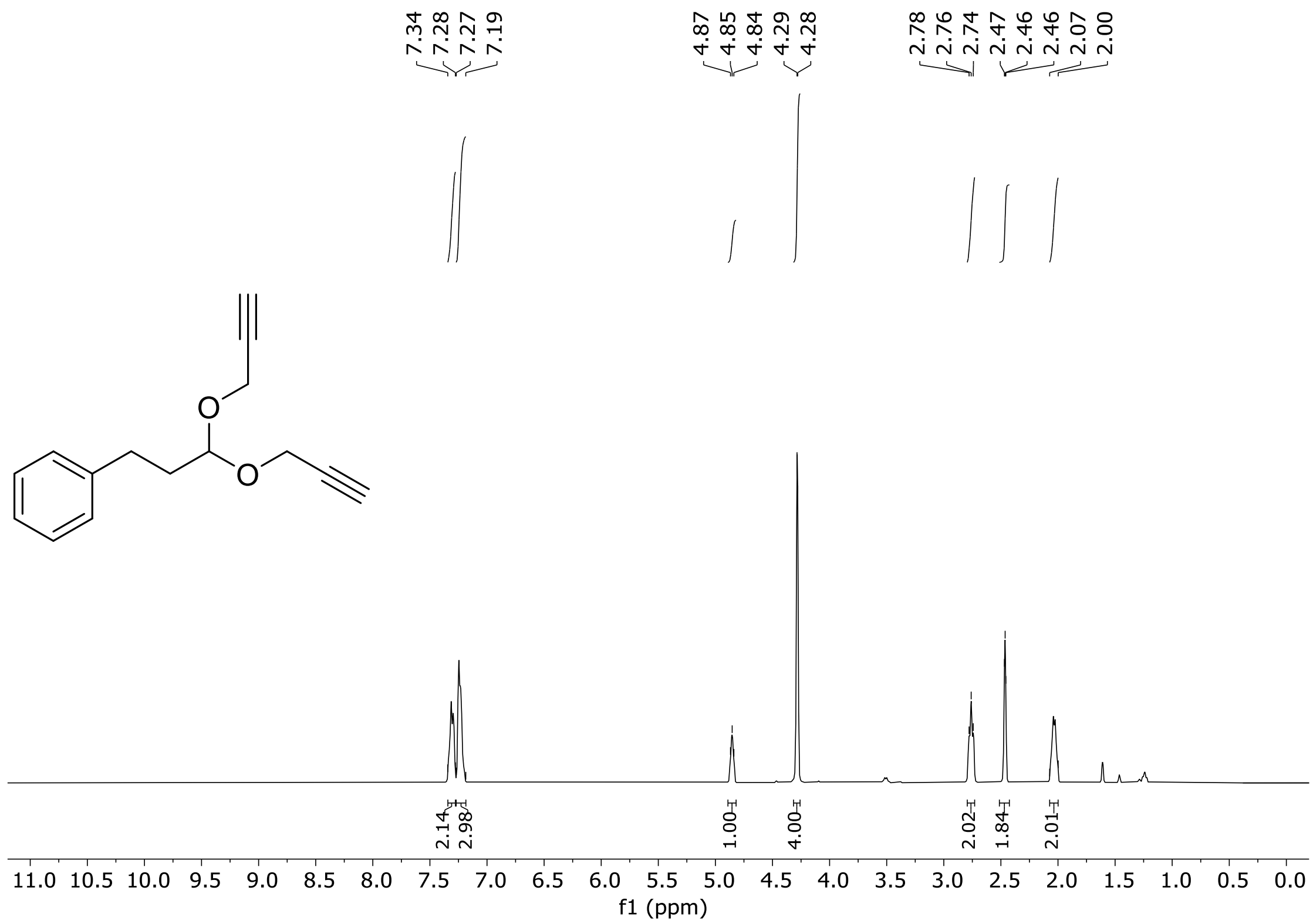

^{1}H-NMR of compound **4h** ($CDCl_3$, 400 MHz).

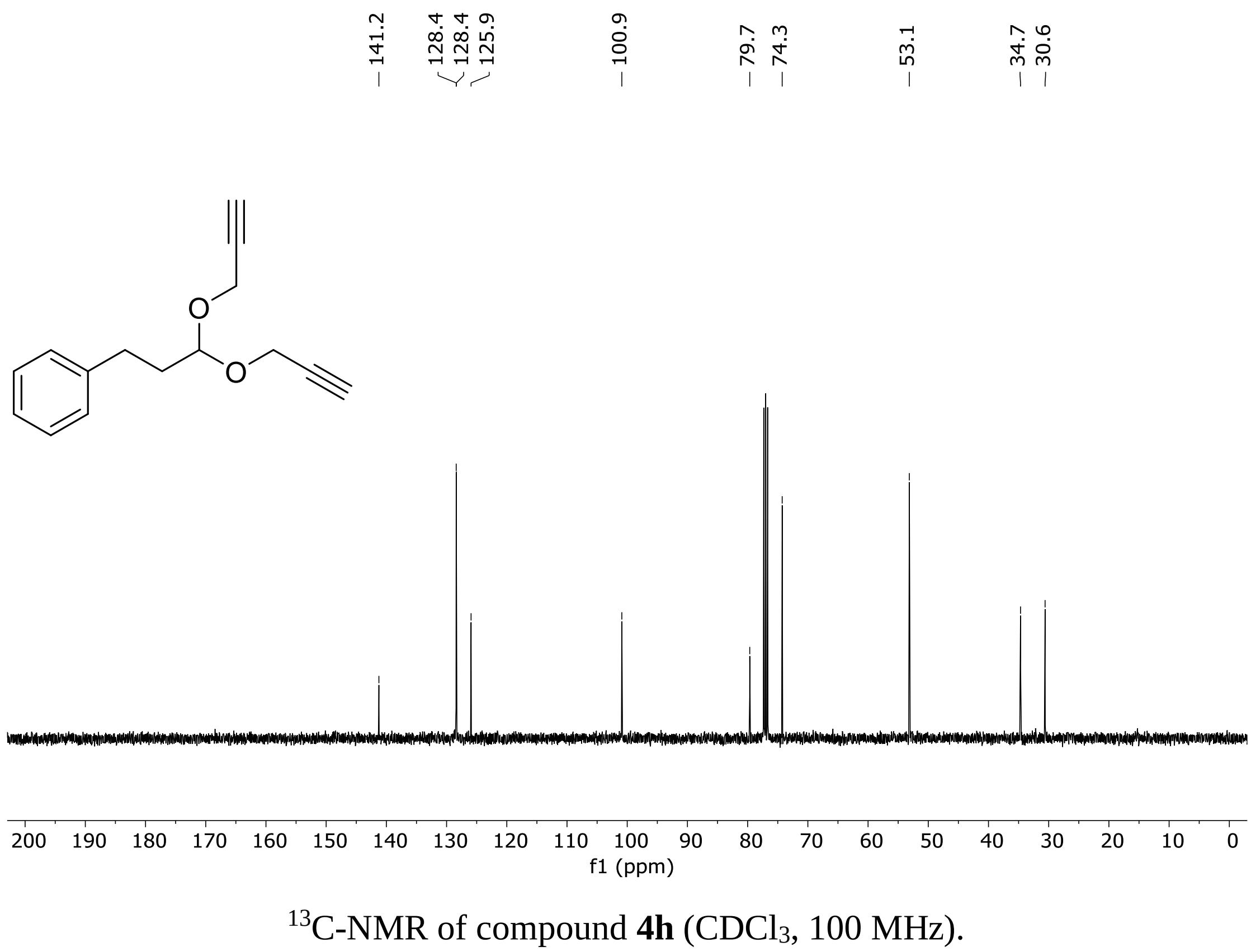

^{13}C-NMR of compound **4h** ($CDCl_3$, 100 MHz).

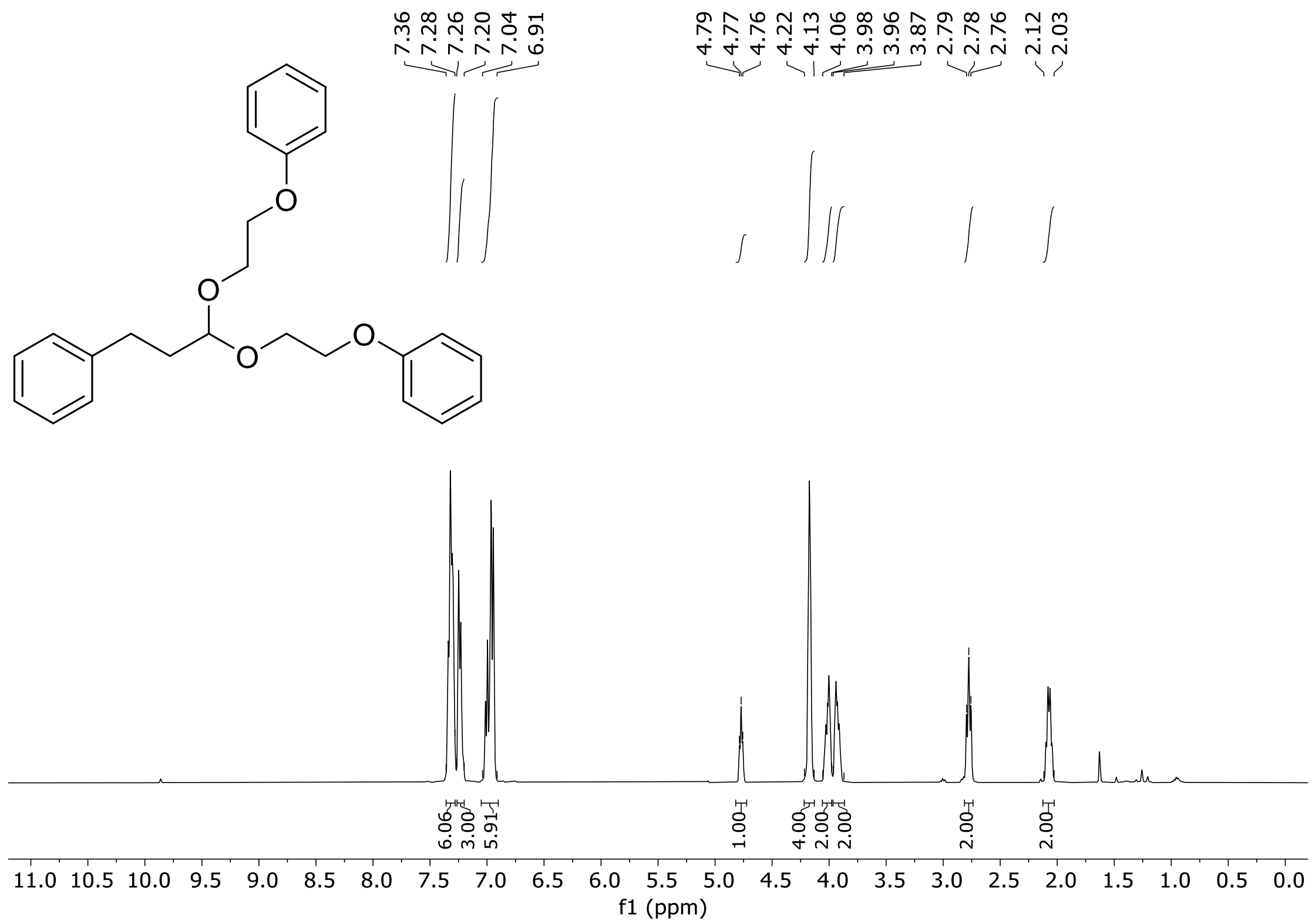

^{1}H-NMR of compound **4i** ($CDCl_3$, 400 MHz).

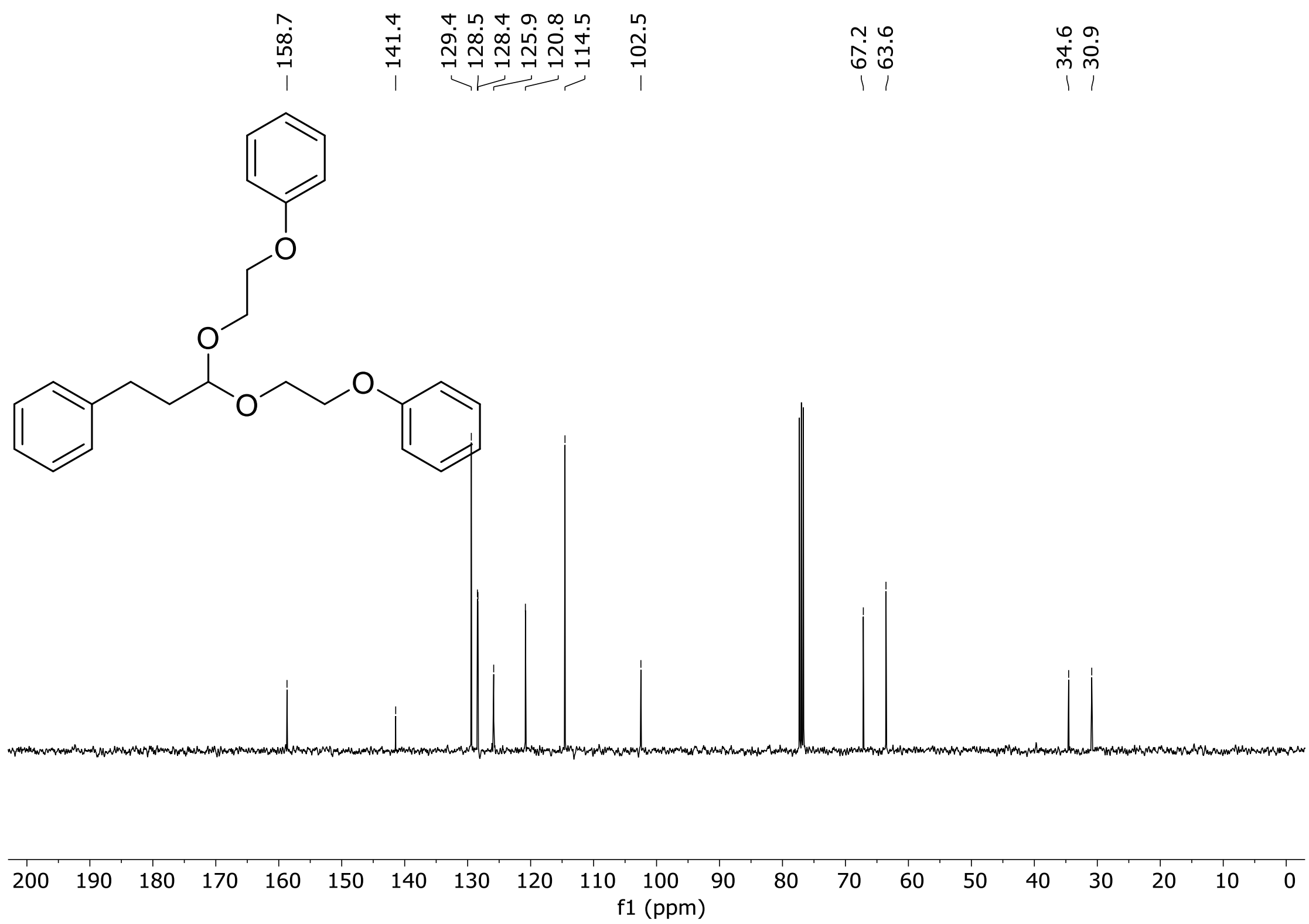

^{13}C-NMR of compound **4i** ($CDCl_3$, 100 MHz).

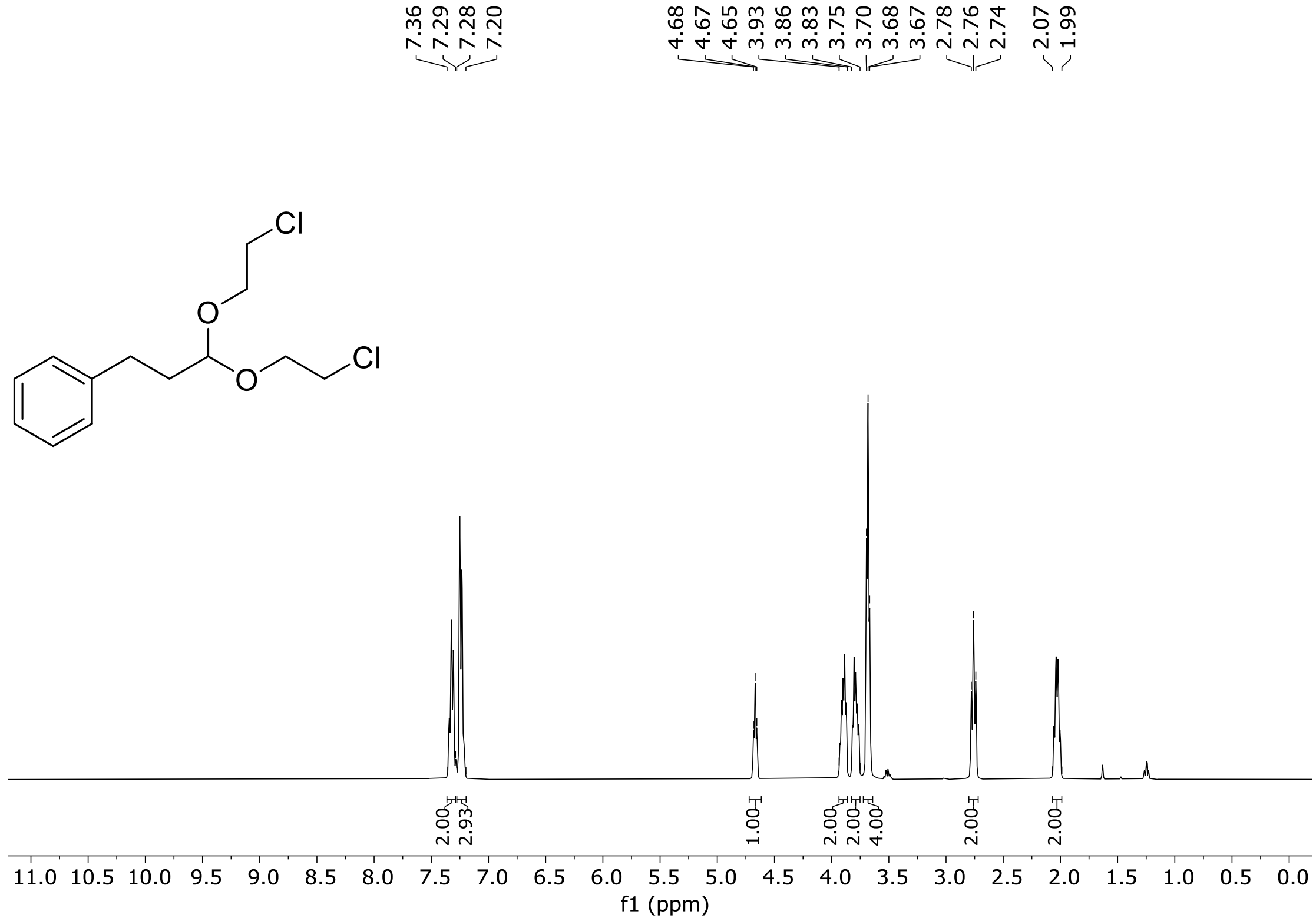

^{1}H-NMR of compound **4j** ($CDCl_3$, 400 MHz).

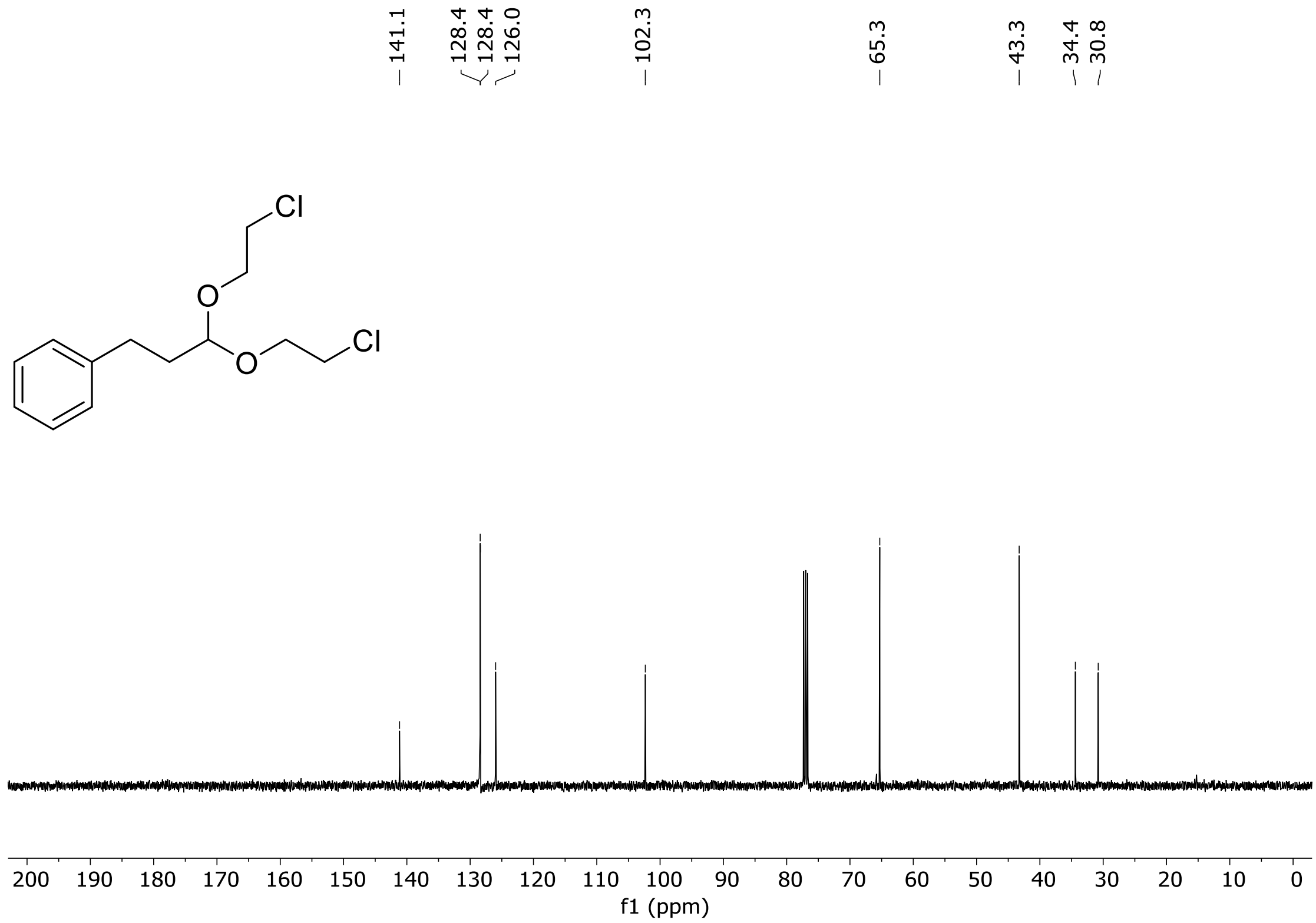

^{1}H-NMR of compound **4j** ($CDCl_3$, 100 MHz).

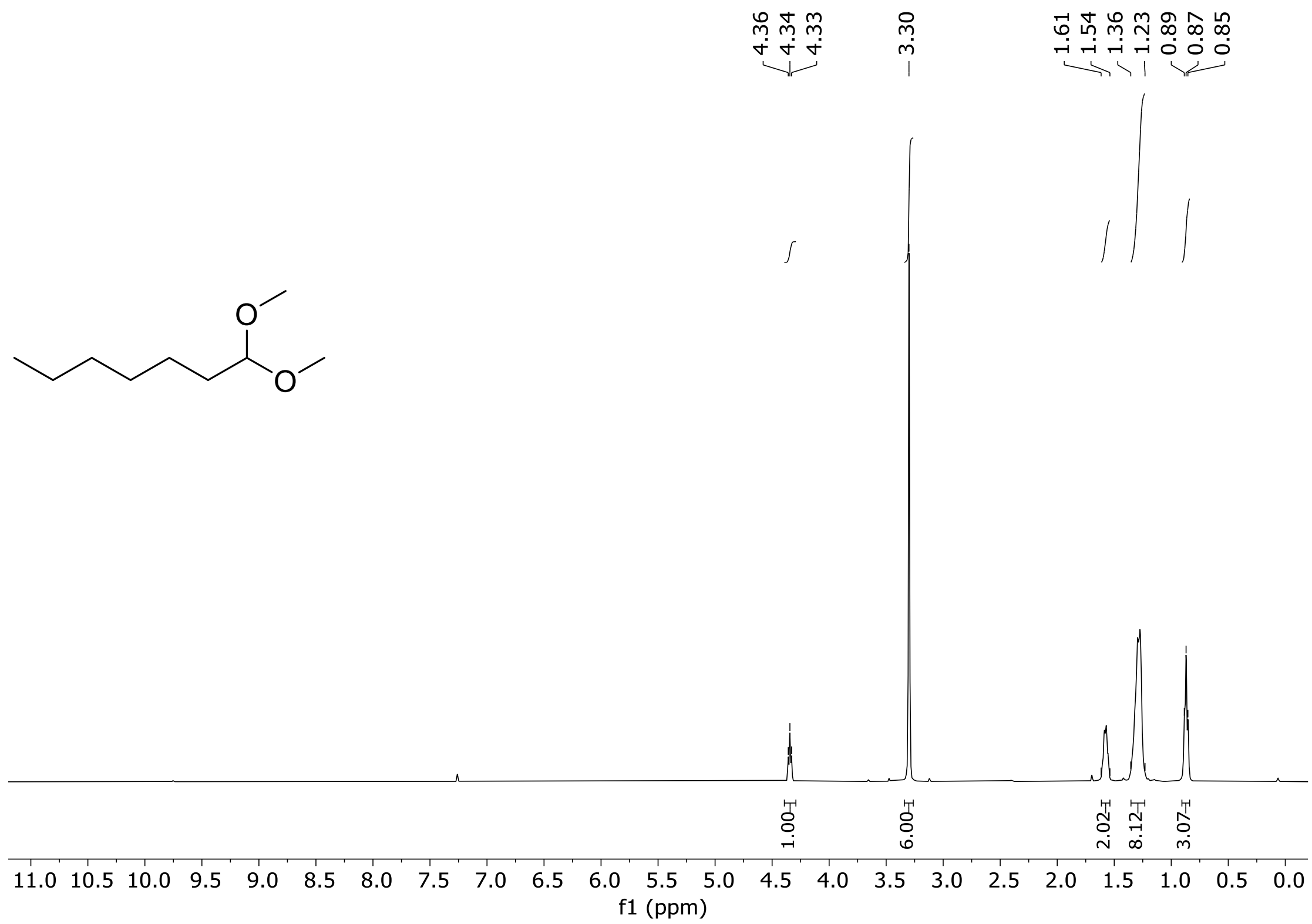

^{1}H-NMR of compound **4k** ($CDCl_3$, 400 MHz).

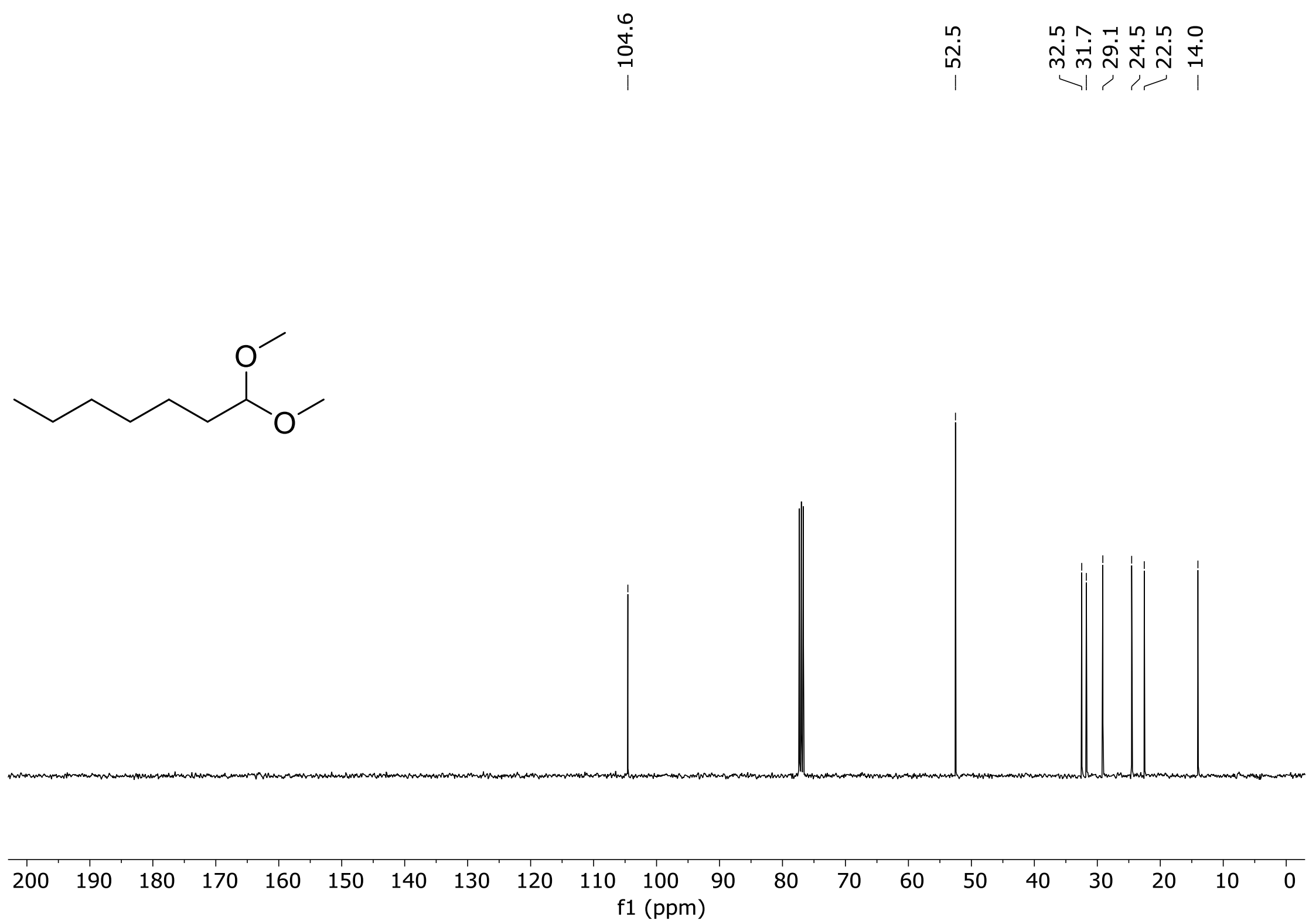

^{13}C-NMR of compound **4k** ($CDCl_3$, 100 MHz).

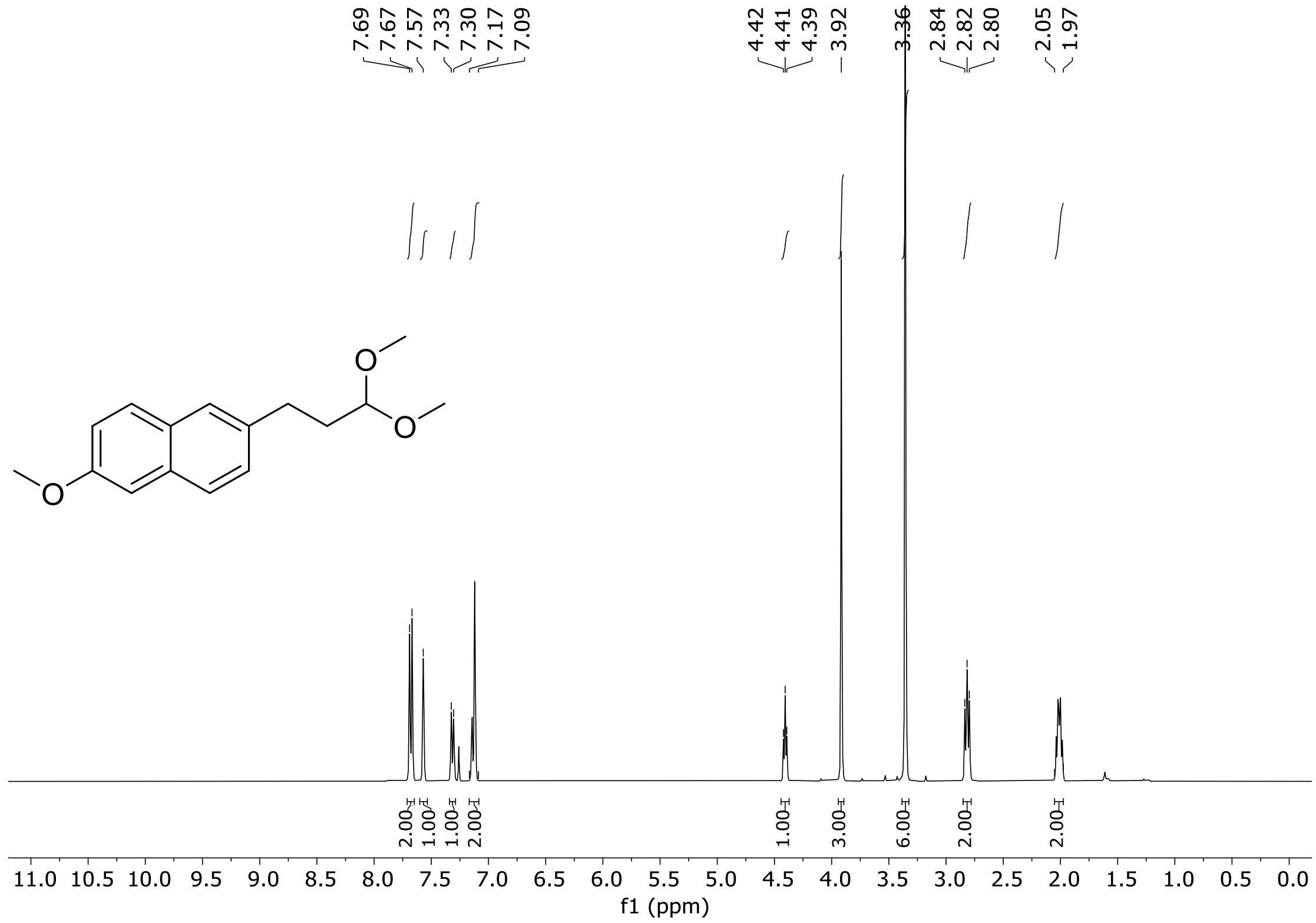

^{1}H-NMR of compound **4l** ($CDCl_3$, 400 MHz).

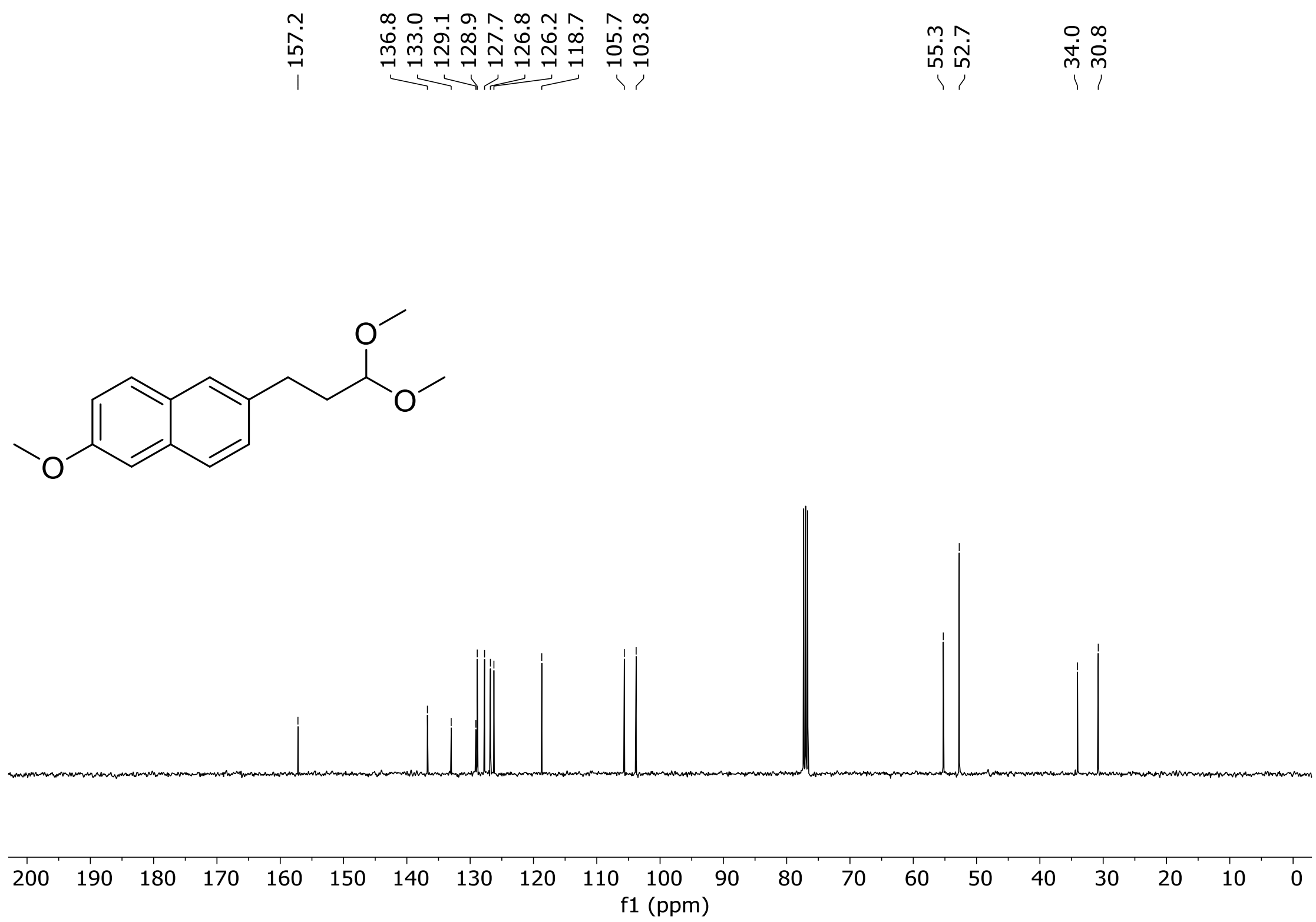

^{13}C-NMR of compound **4l** ($CDCl_3$, 100 MHz).

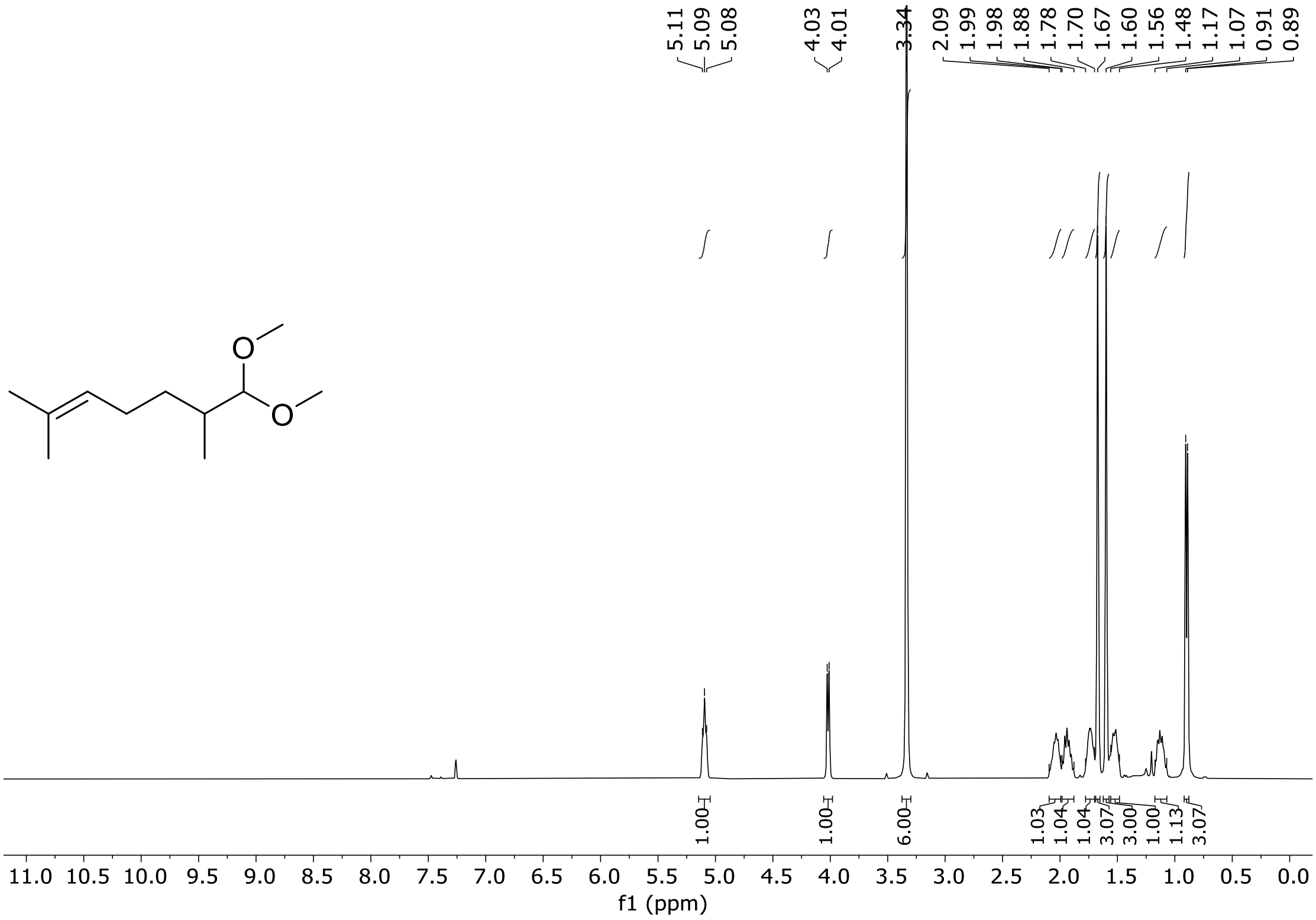

^{1}H-NMR of compound **4m** ($CDCl_3$, 400 MHz).

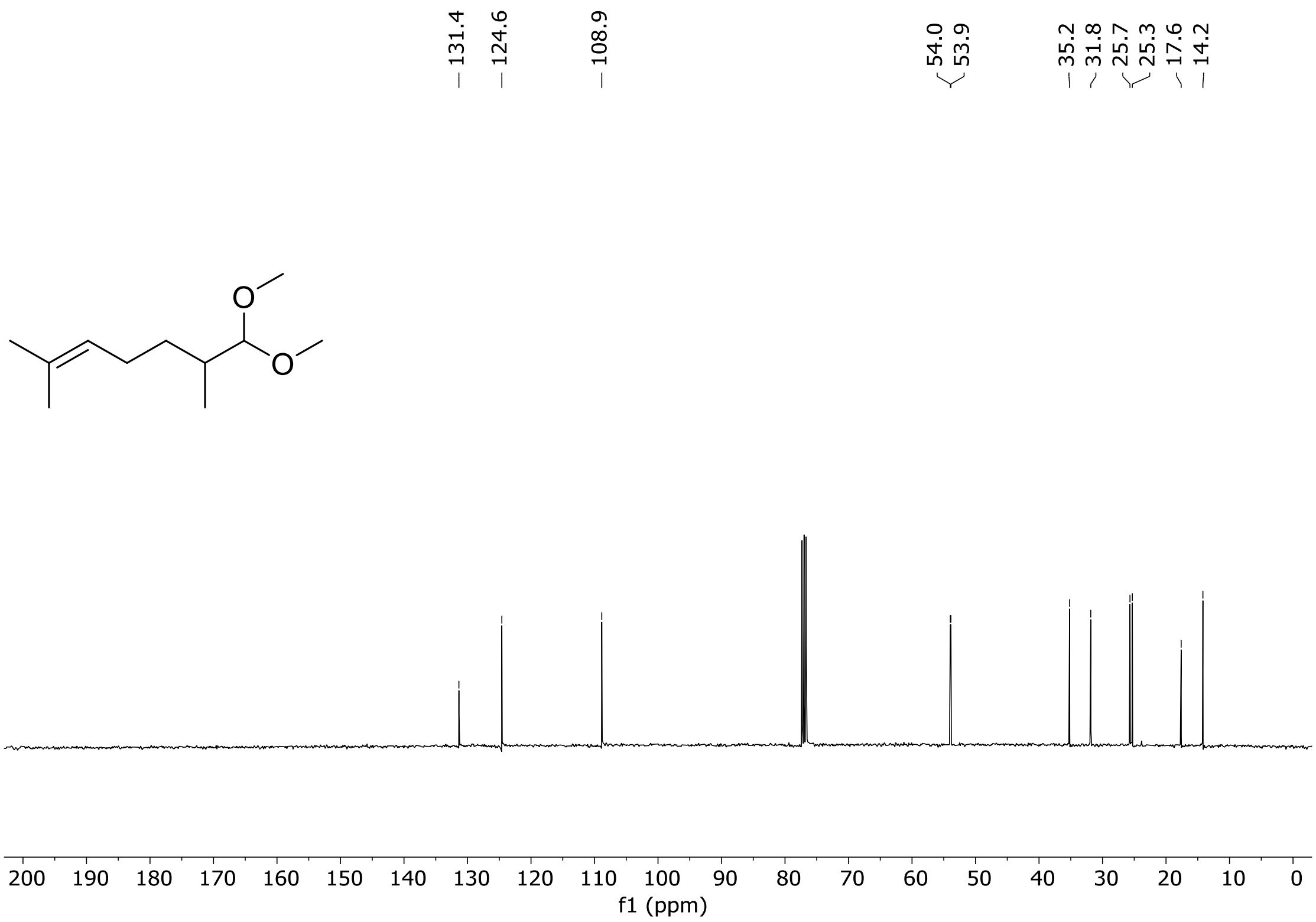

^{13}C-NMR of compound **4m** ($CDCl_3$, 100 MHz).

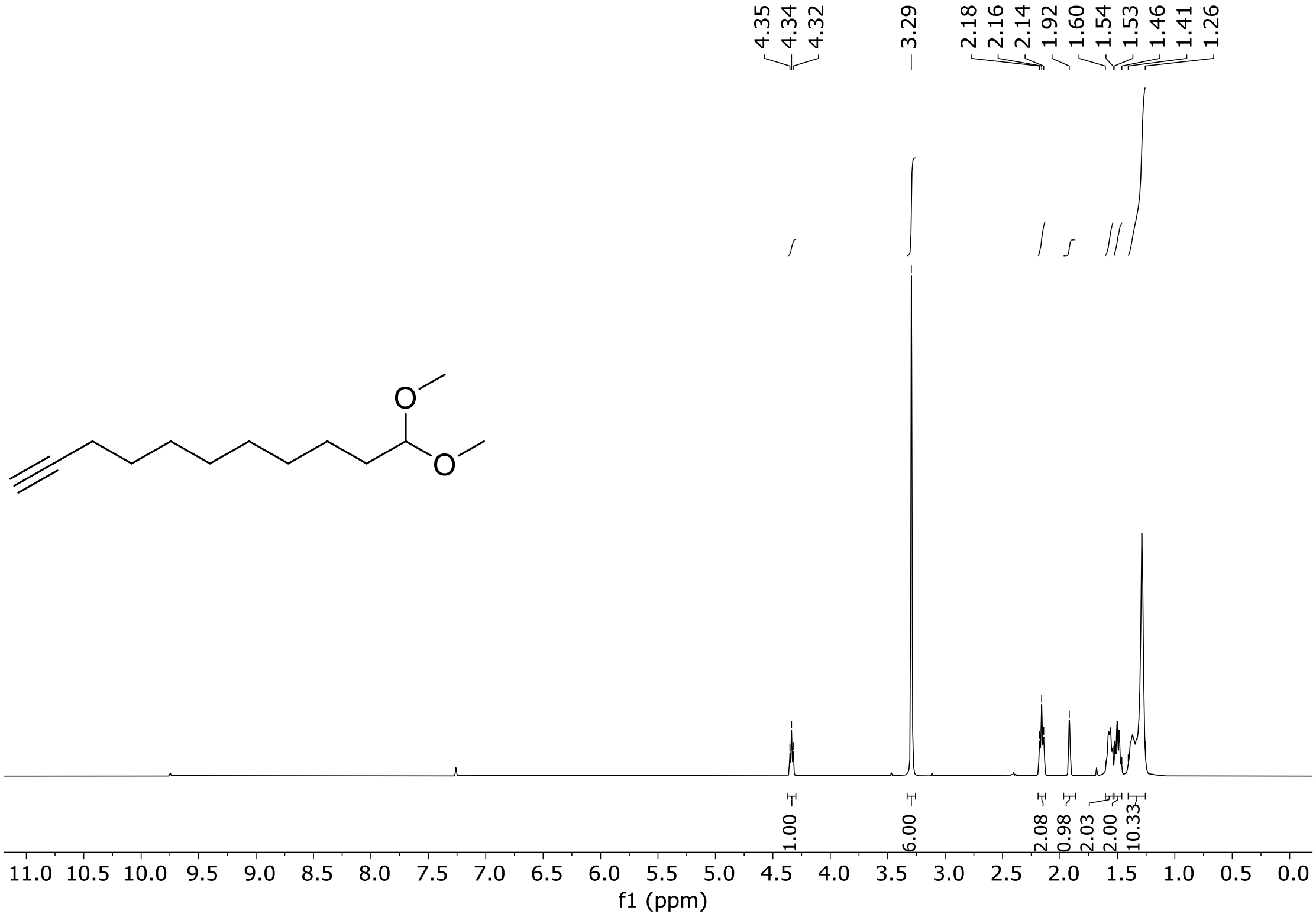

^{1}H-NMR of compound **4n** ($CDCl_3$, 400 MHz).

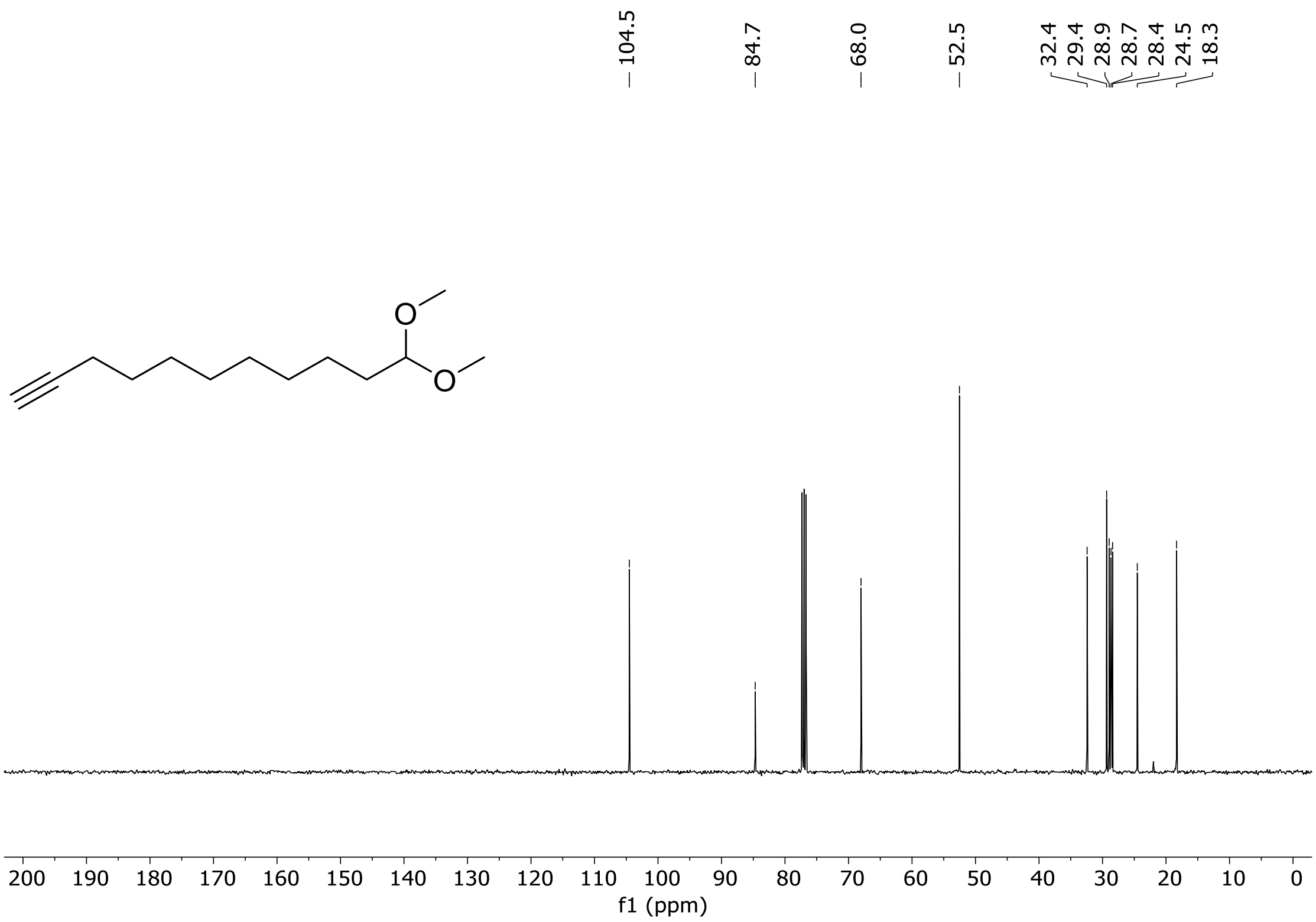

^{13}C-NMR of compound **4n** ($CDCl_3$, 100 MHz).

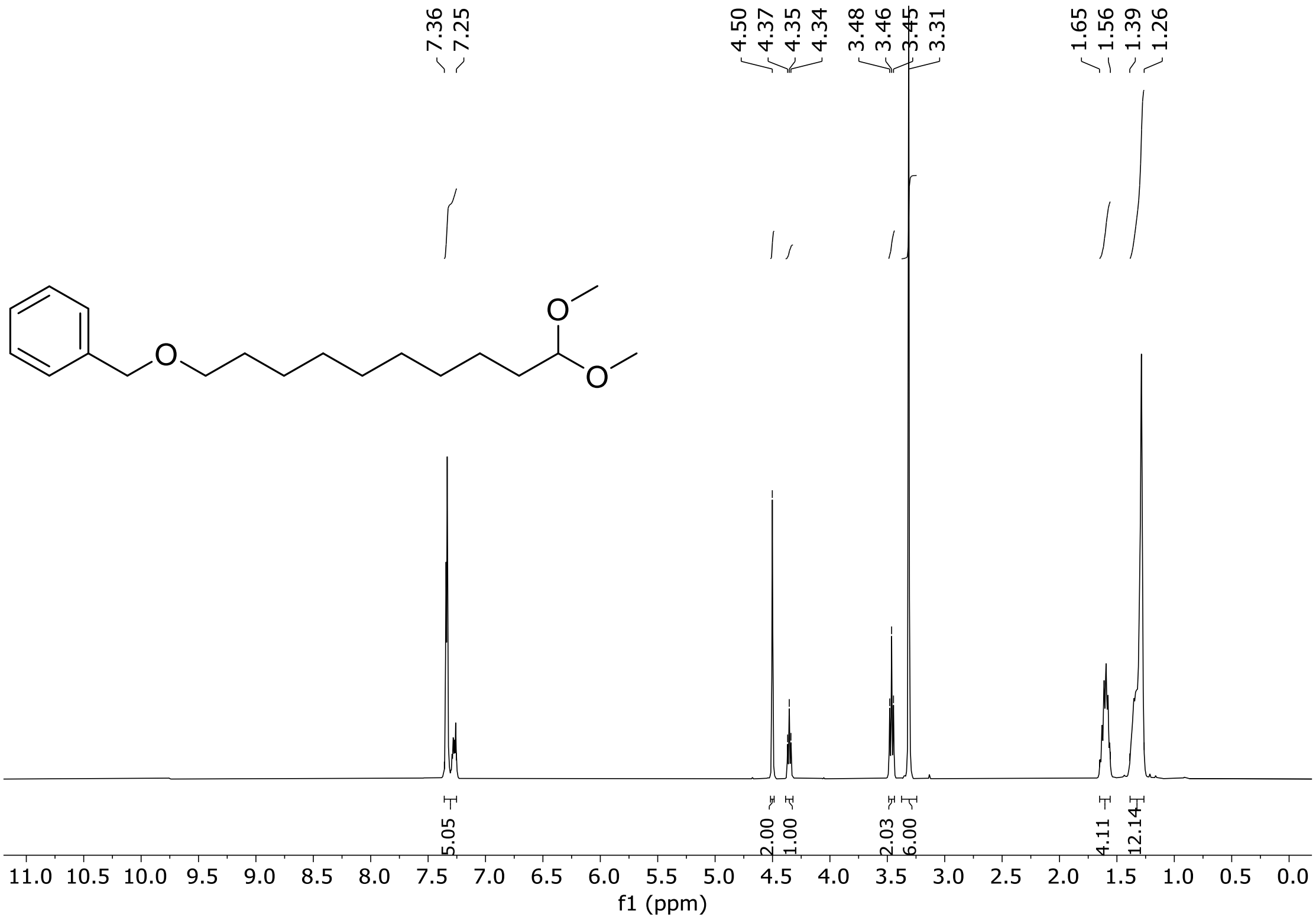

^{1}H-NMR of compound **4o** ($CDCl_3$, 400 MHz).

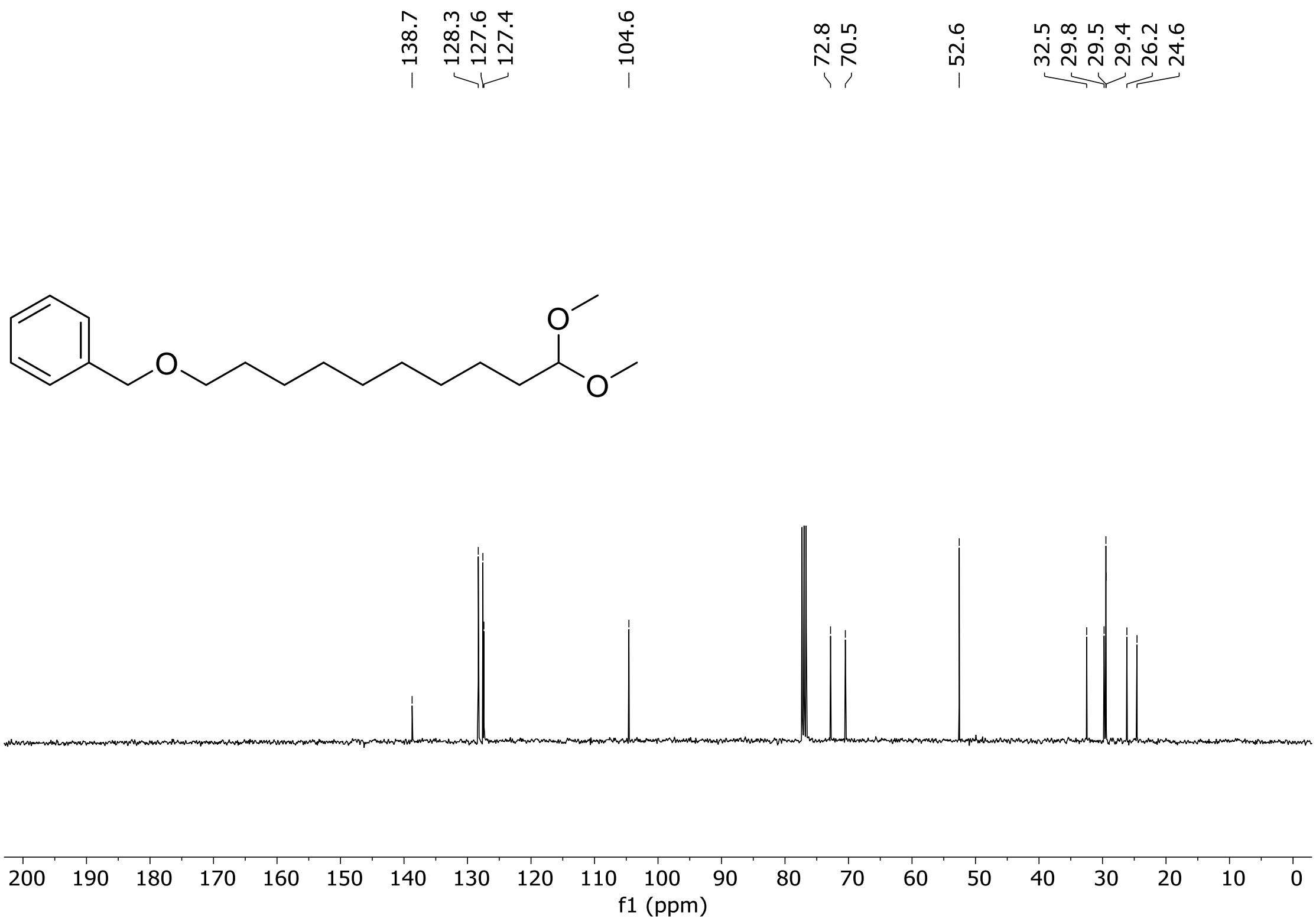

^{13}C-NMR of compound **4o** ($CDCl_3$, 100 MHz).

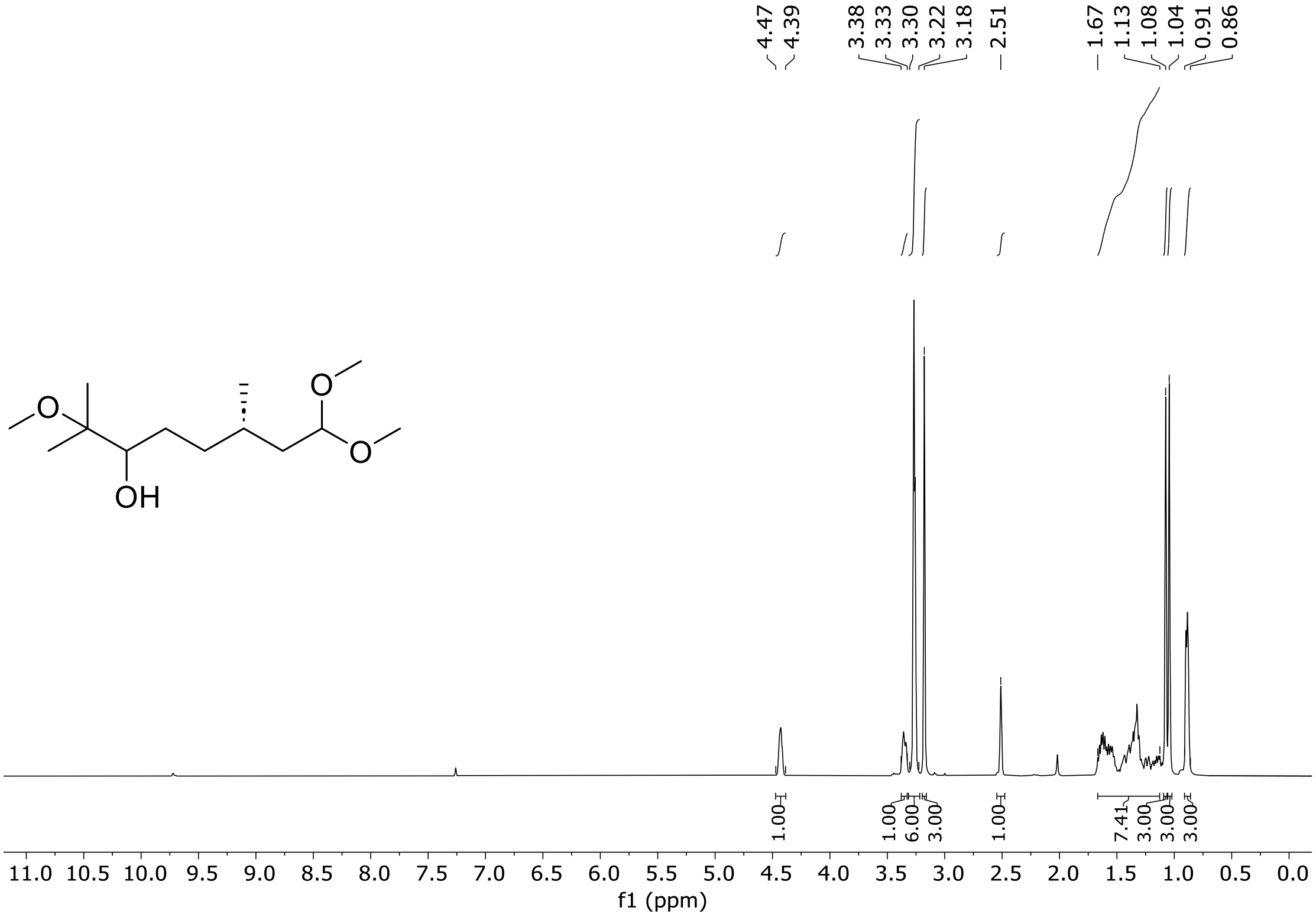

^{1}H-NMR of compound **4p** ($CDCl_3$, 400 MHz).

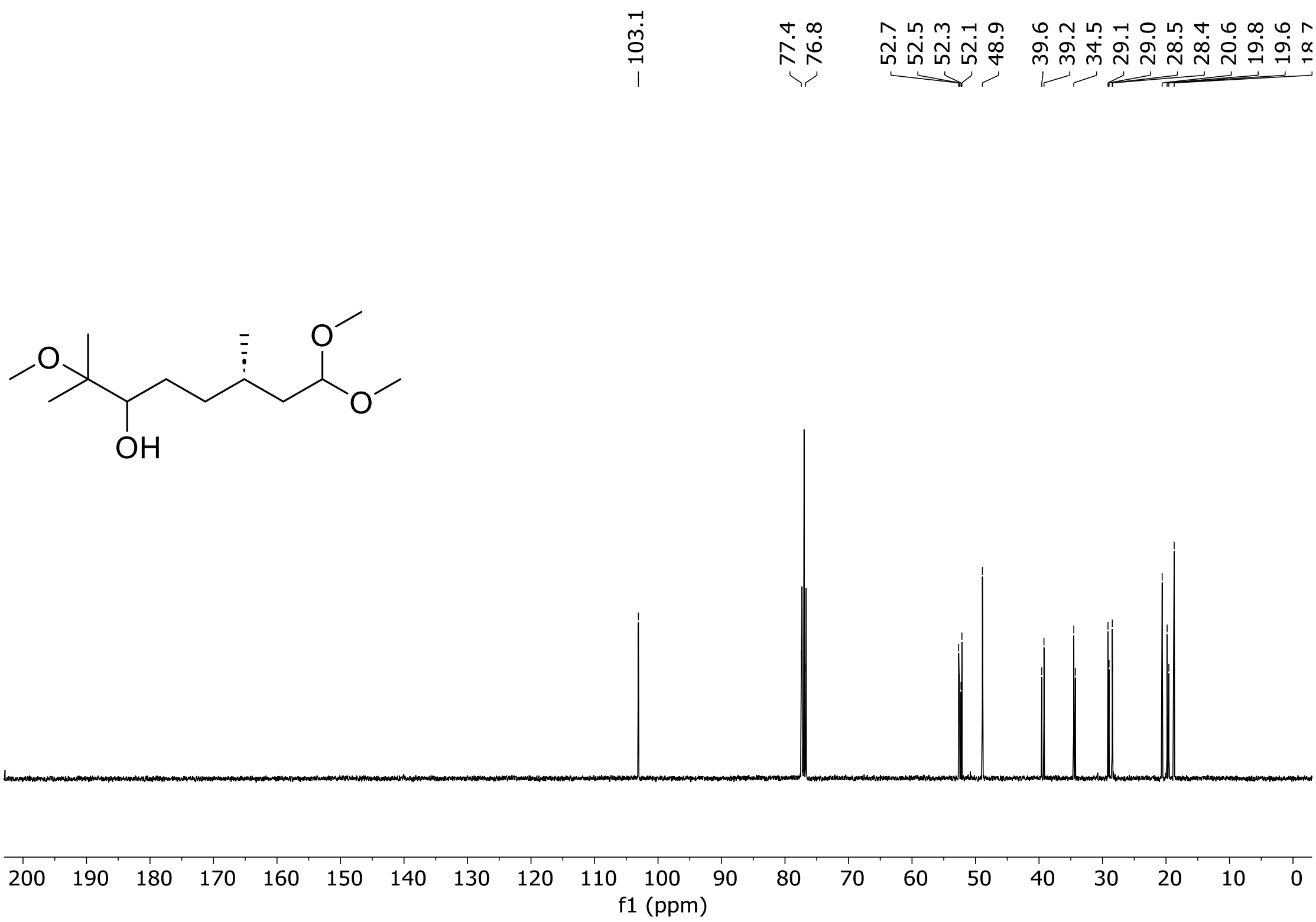

^{13}C-NMR of compound **4p** ($CDCl_3$, 100 MHz).

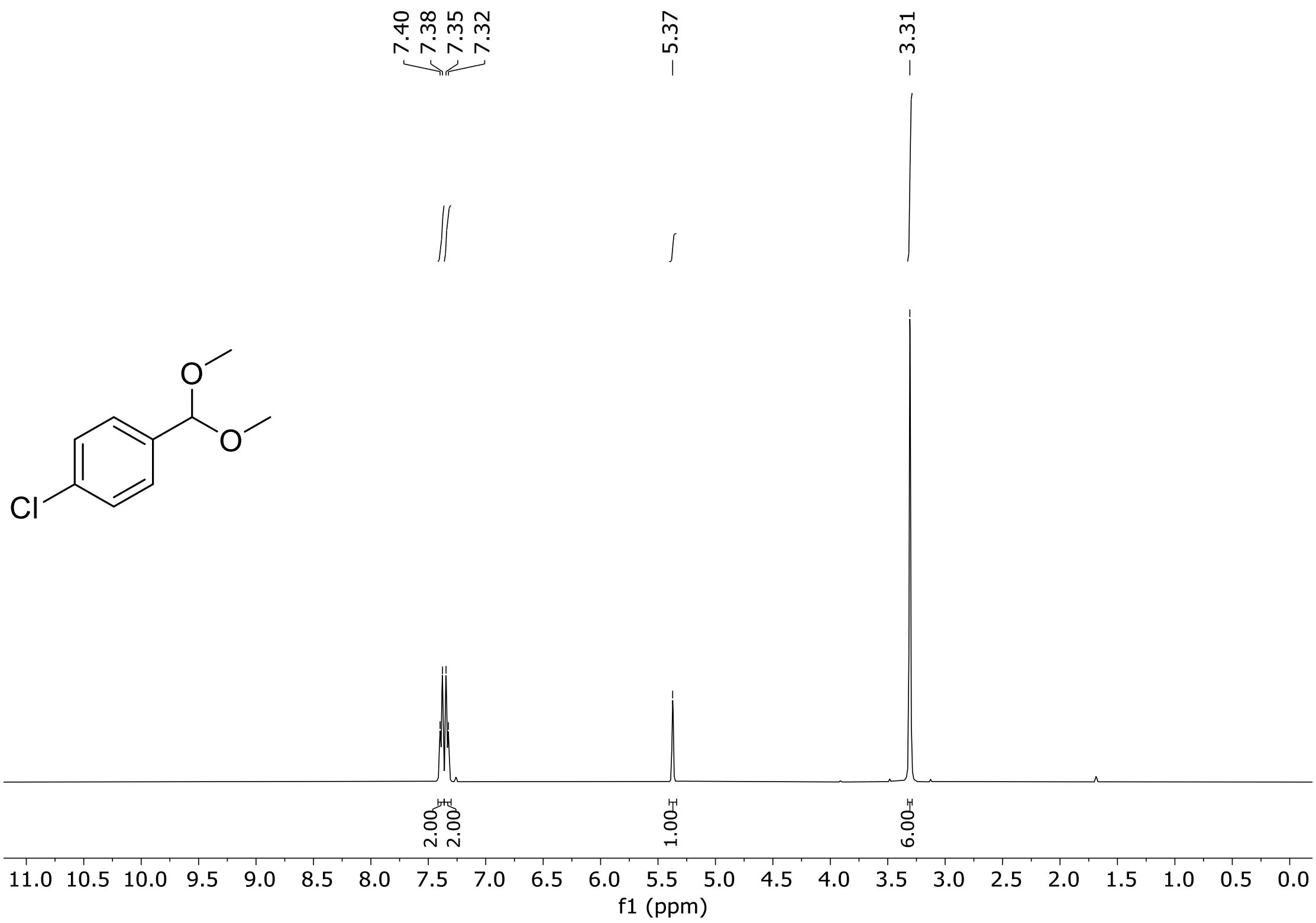

^{1}H-NMR of compound **4q** ($CDCl_3$, 400 MHz).

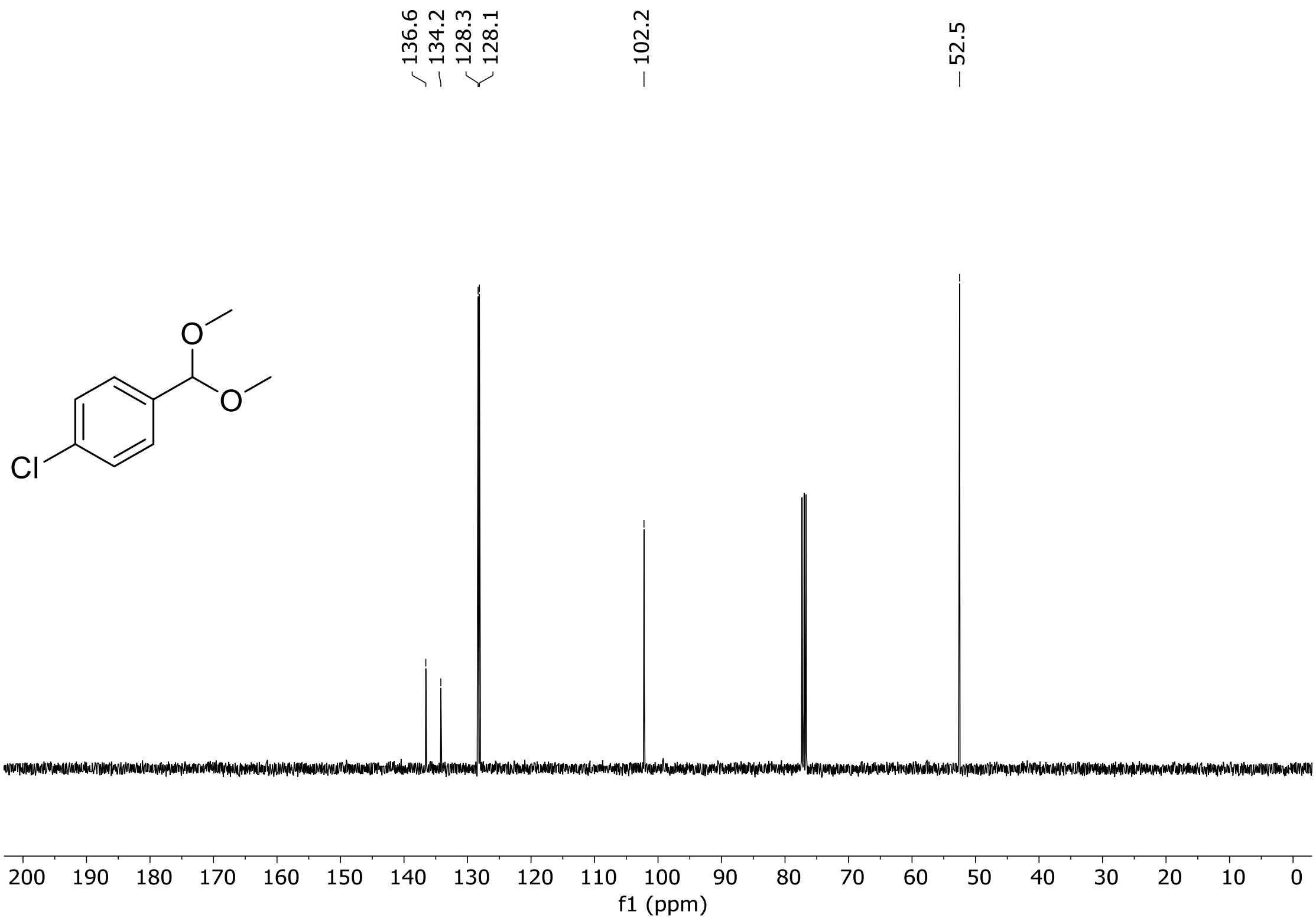

^{13}C-NMR of compound **4q** ($CDCl_3$, 100 MHz).

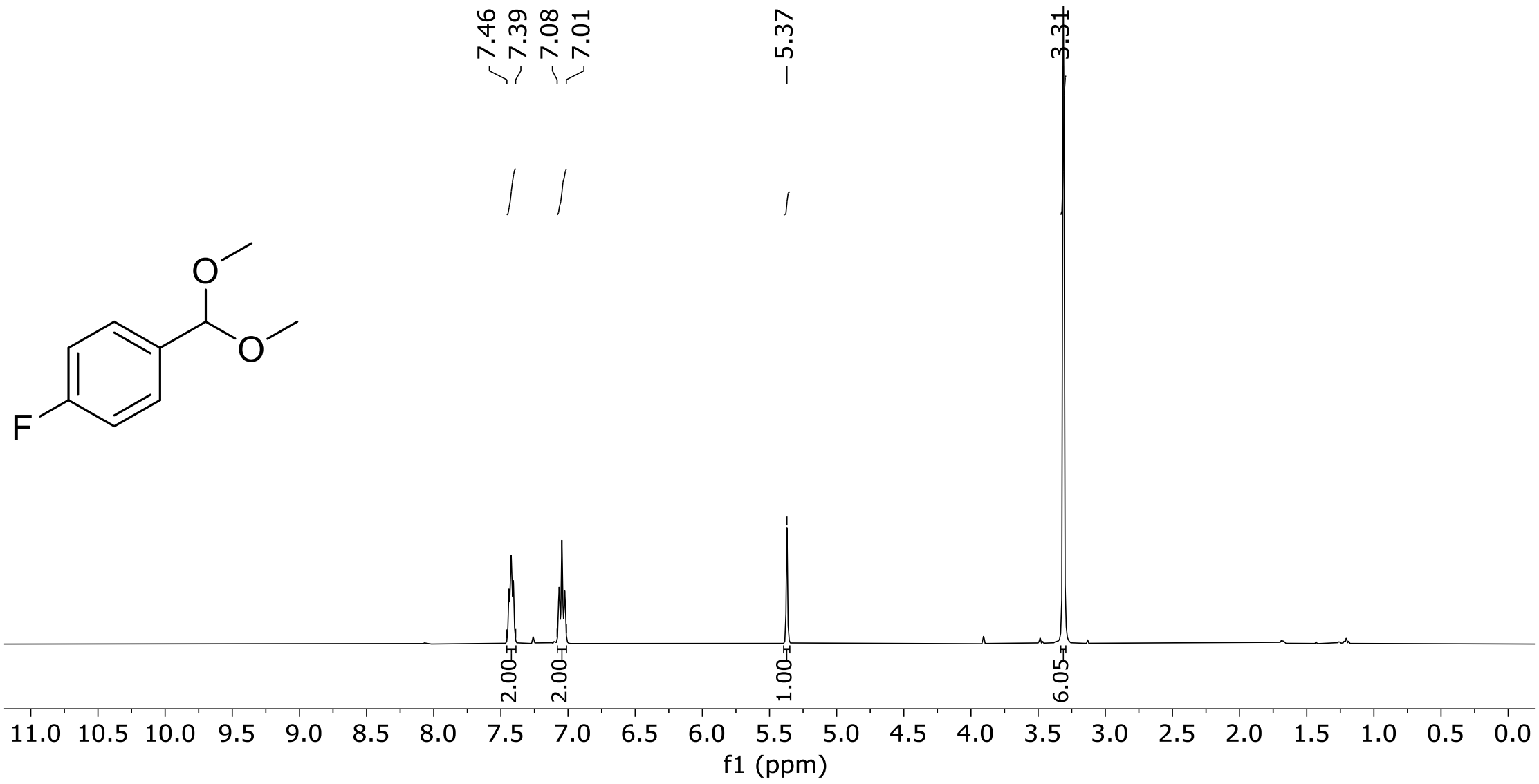

^{1}H-NMR of compound **4r** ($CDCl_3$, 400 MHz).

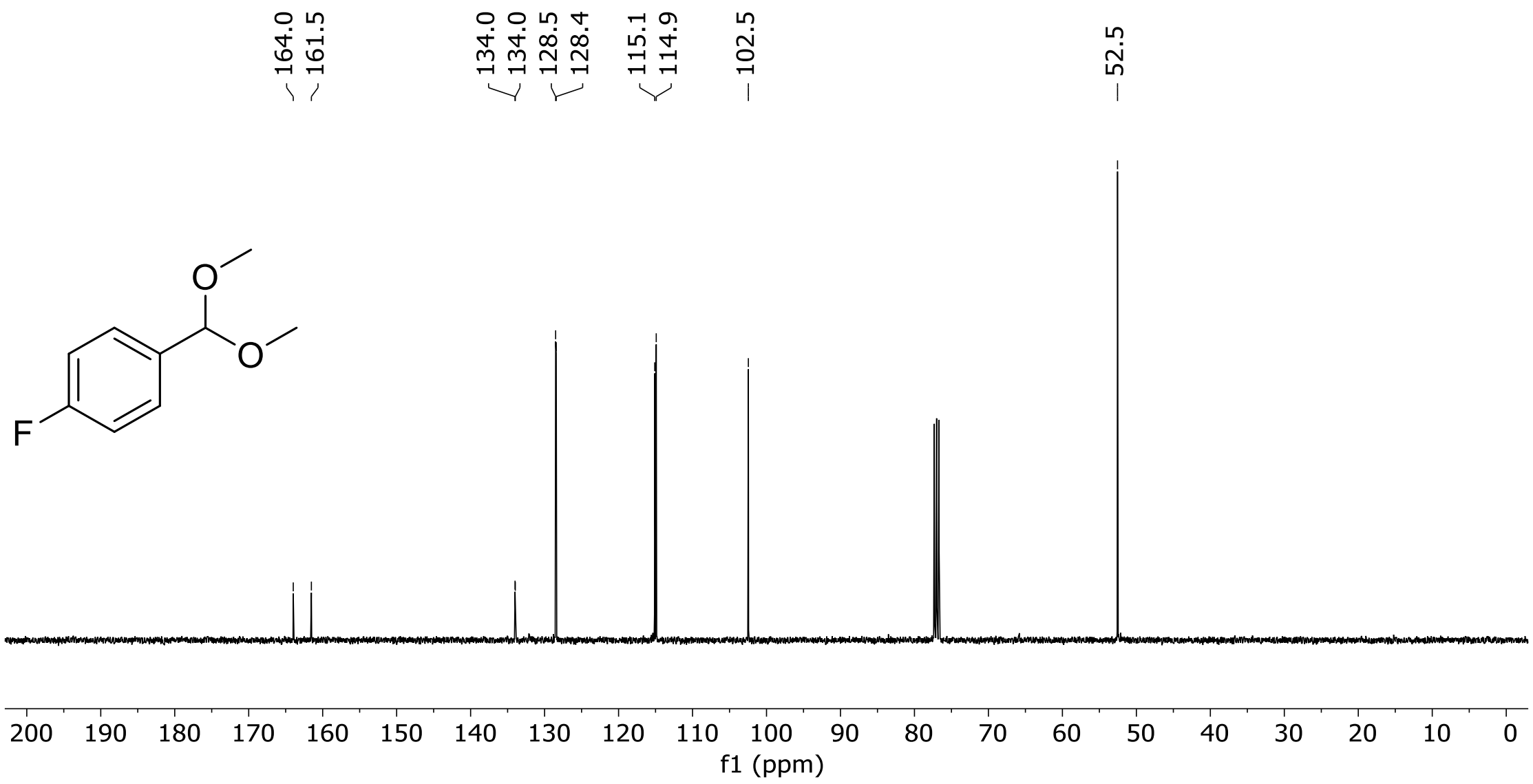

^{13}C-NMR of compound **4r** ($CDCl_3$, 100 MHz).

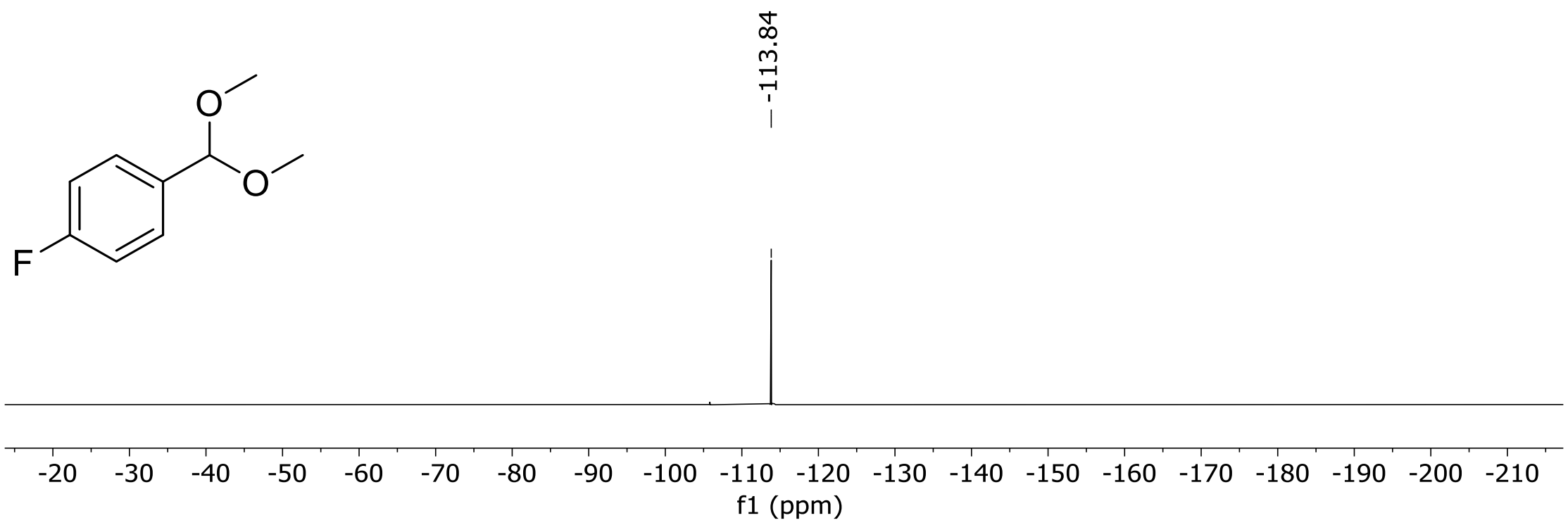

^{19}F-NMR of compound **4r** ($CDCl_3$, 377 MHz).

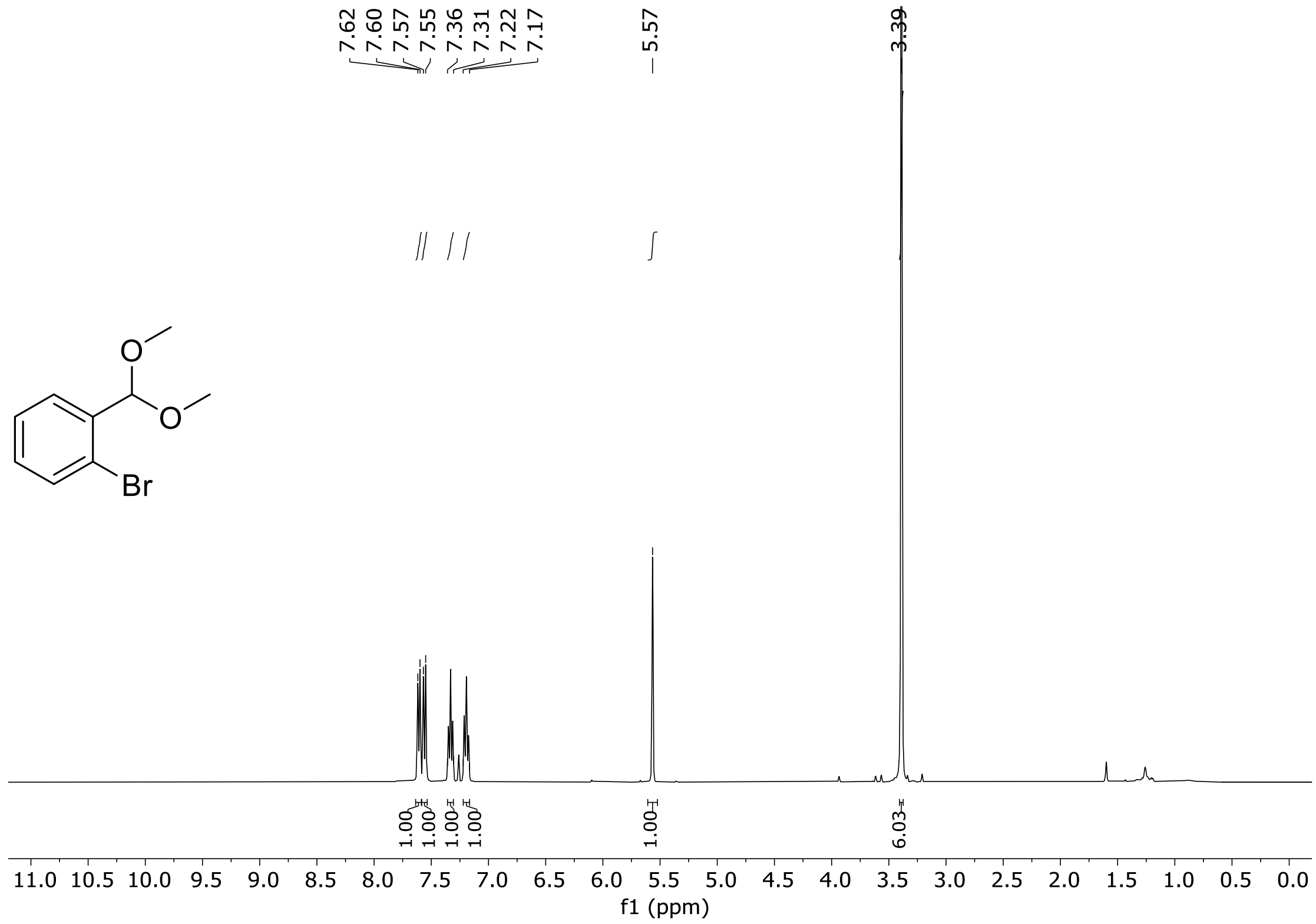

^{1}H-NMR of compound **4s** ($CDCl_3$, 400 MHz).

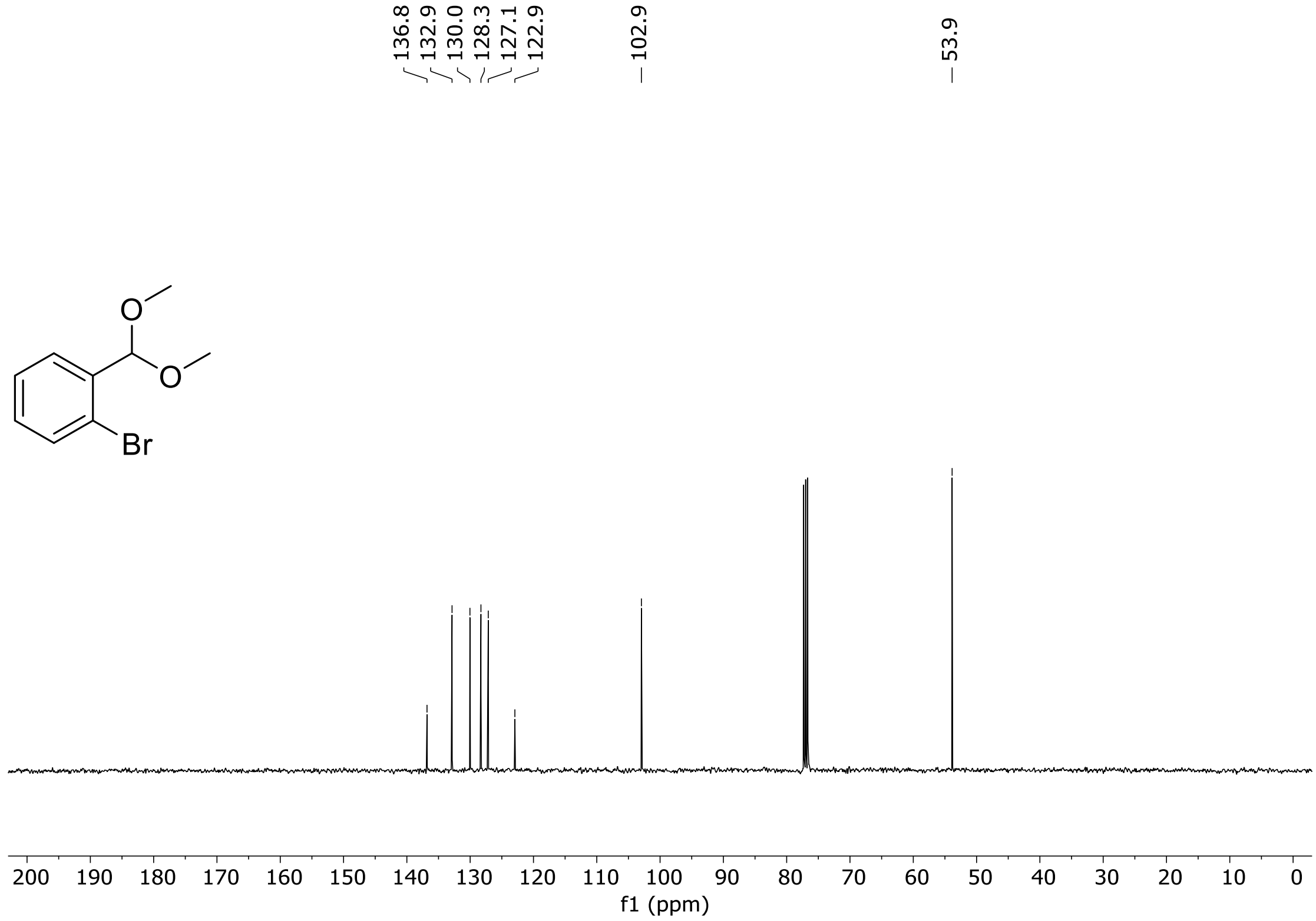

^{13}C-NMR of compound **4s** ($CDCl_3$, 100 MHz).

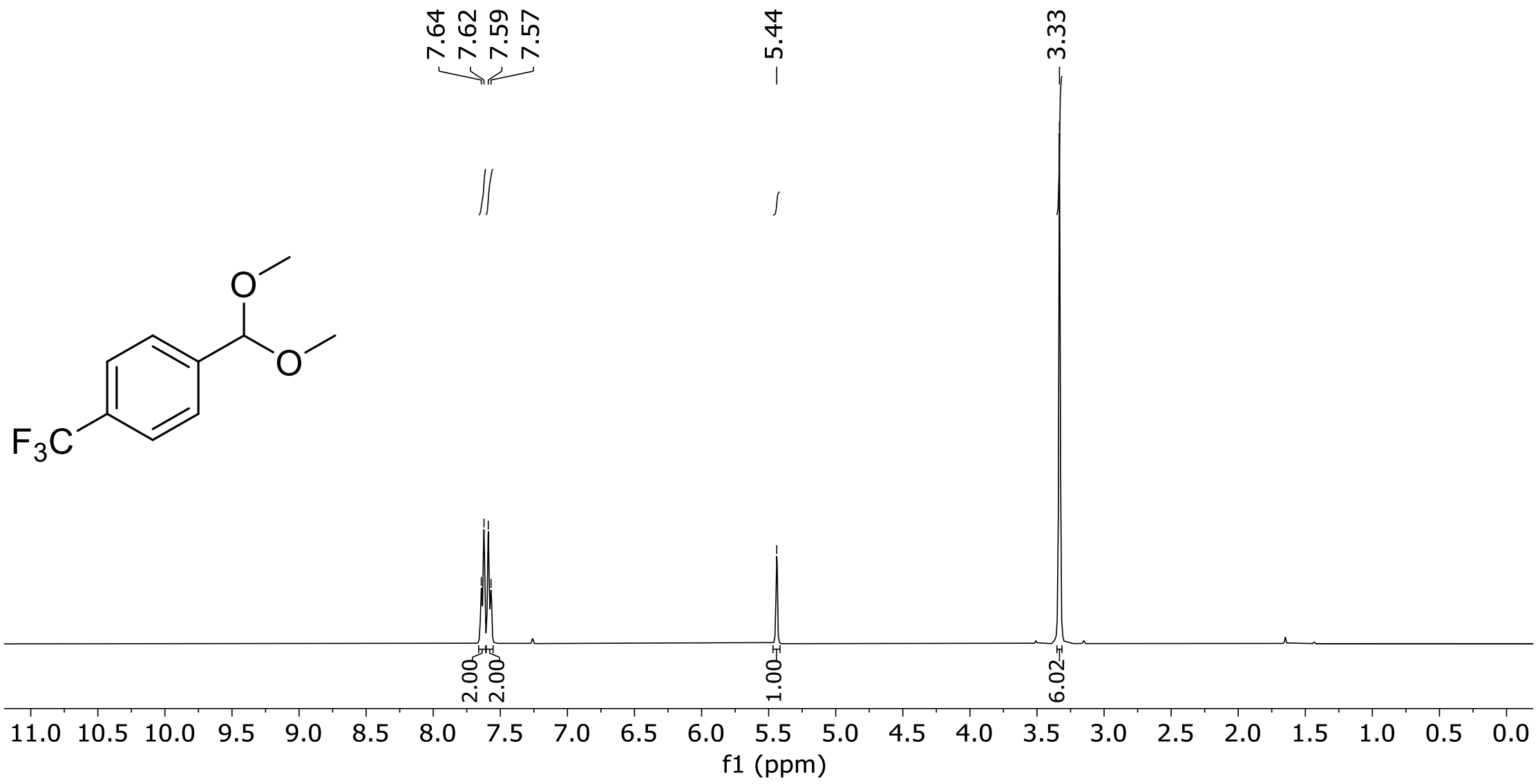

^{1}H-NMR of compound **4t** ($CDCl_3$, 400 MHz).

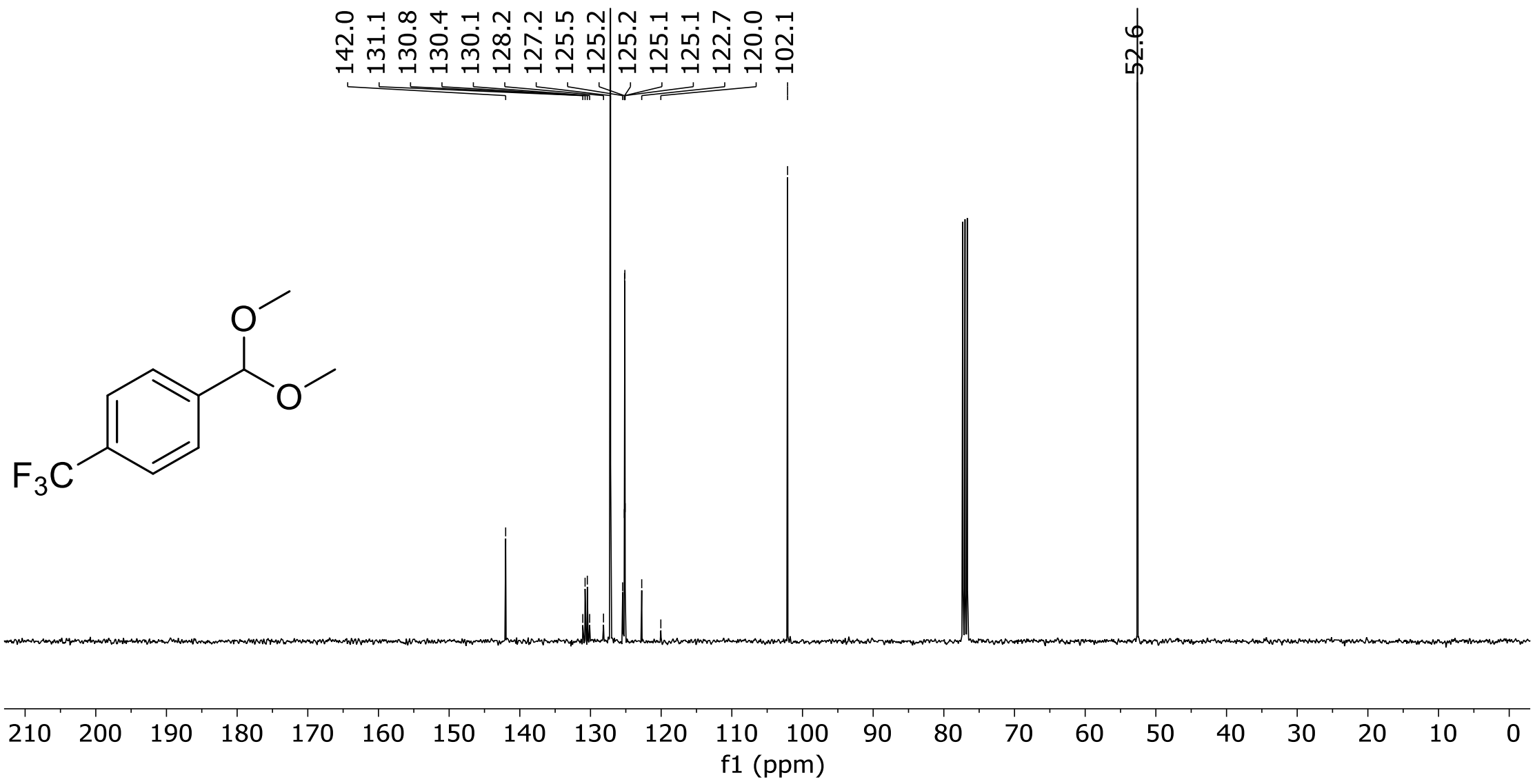

^{13}C-NMR of compound **4t** ($CDCl_3$, 100 MHz).

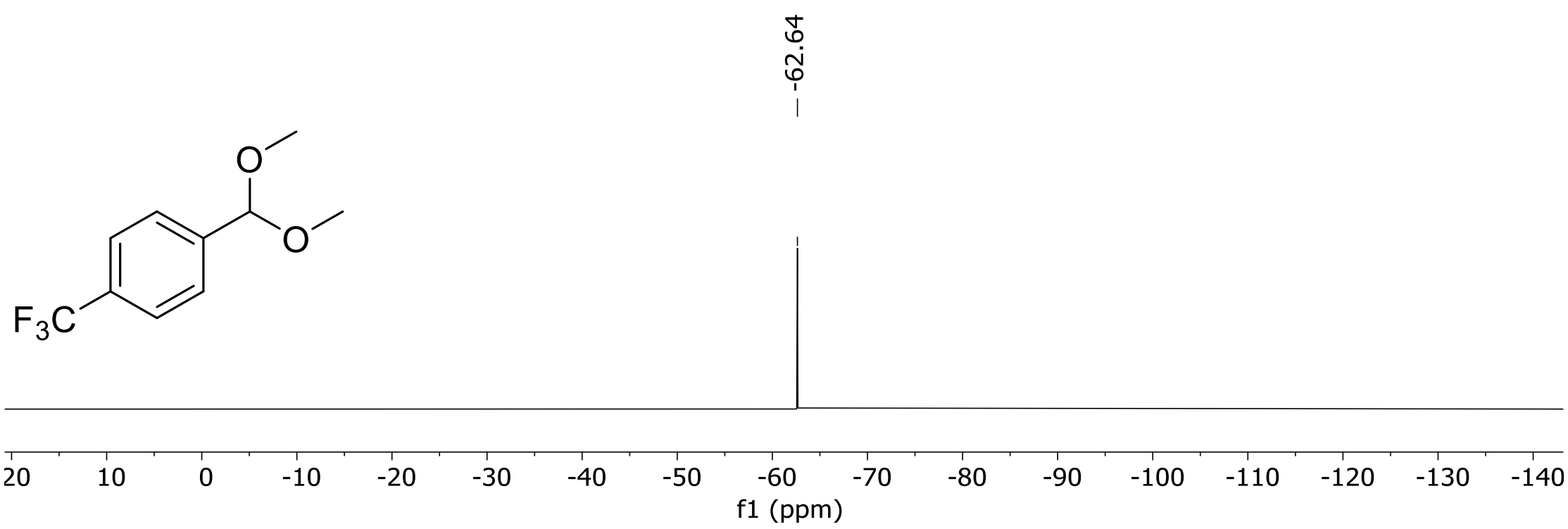

^{19}F-NMR of compound **4t** ($CDCl_3$, 377 MHz).

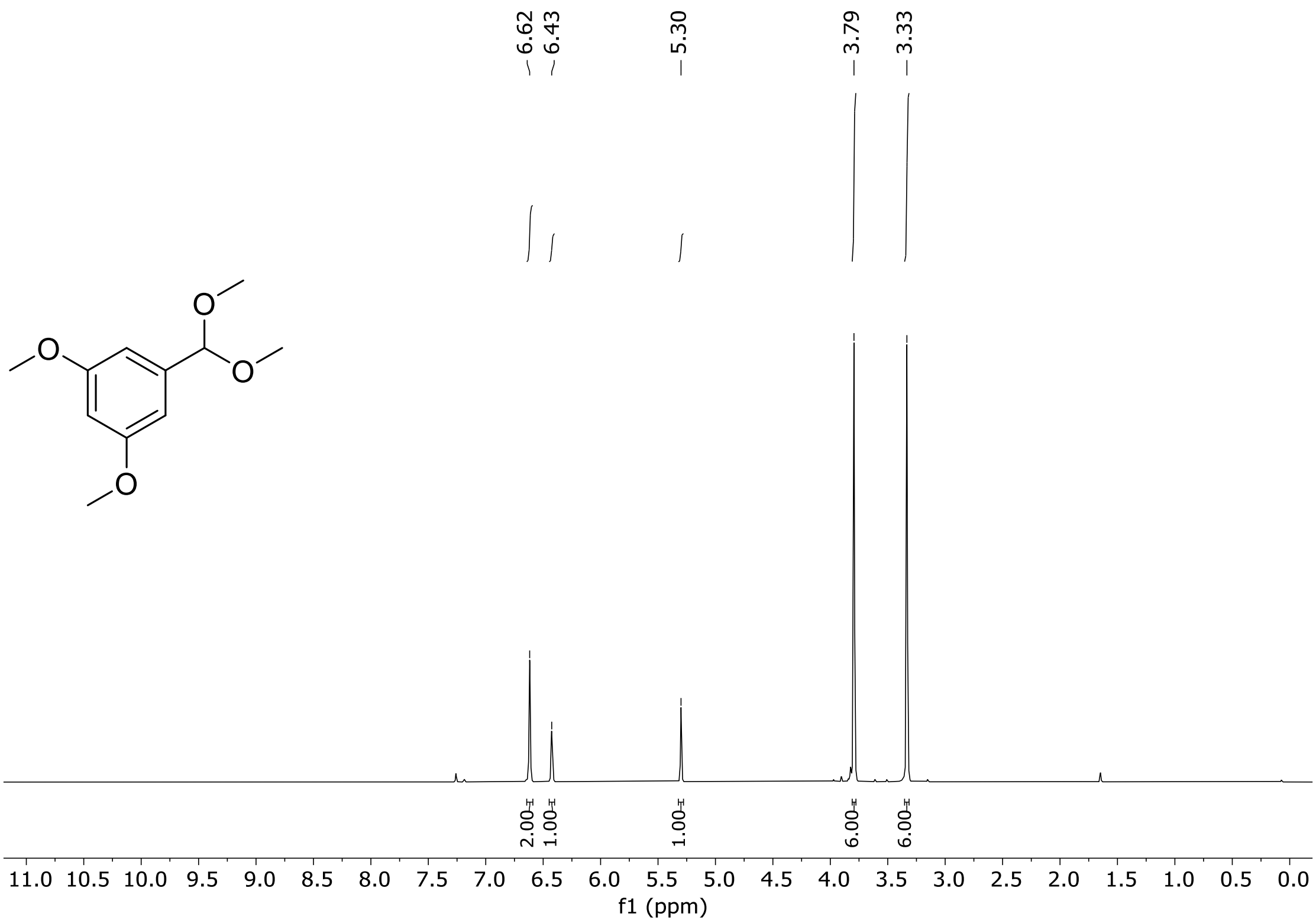

^{1}H-NMR of compound **4u** ($CDCl_3$, 400 MHz).

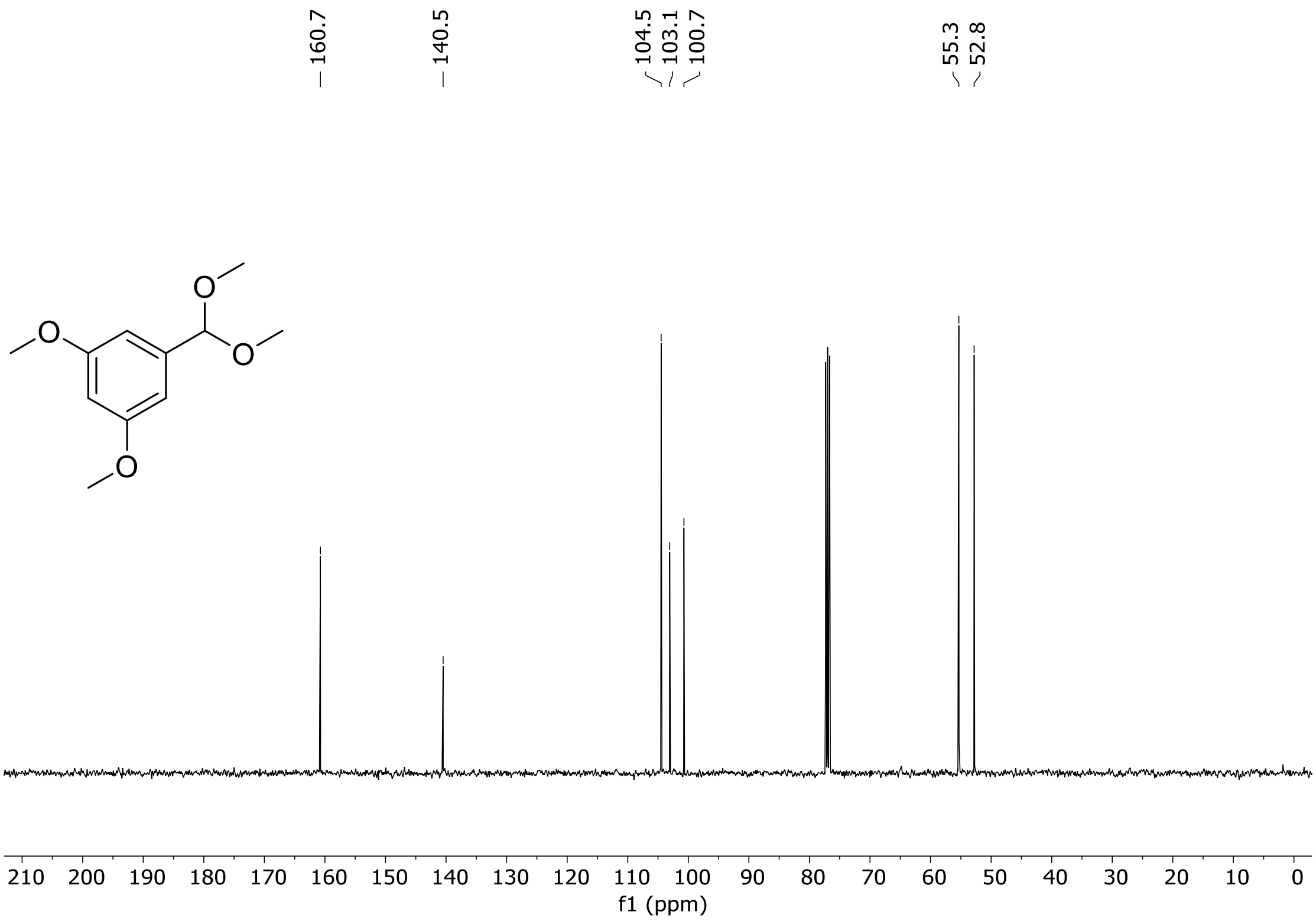

^{13}C-NMR of compound **4u** ($CDCl_3$, 100 MHz).

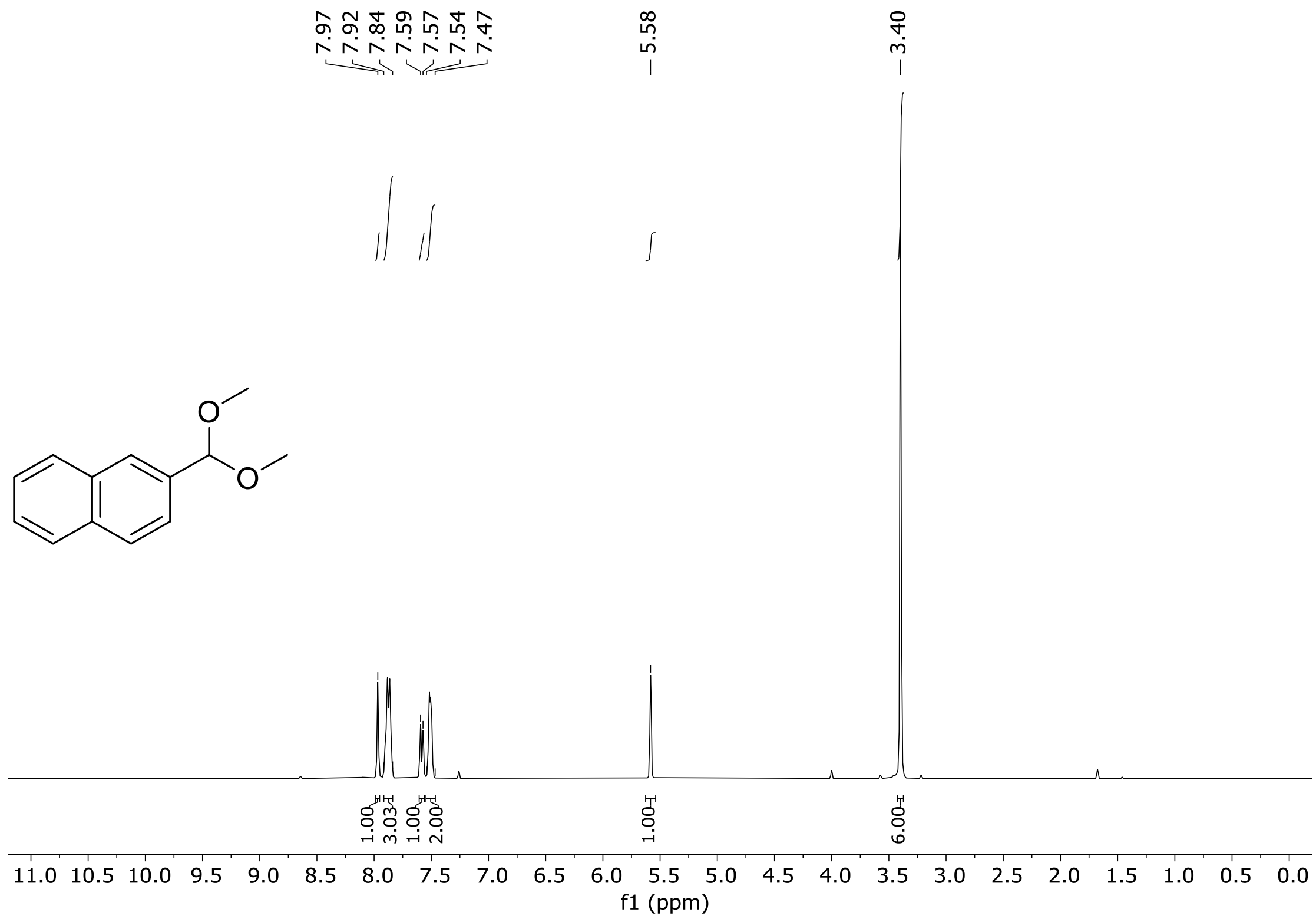

^{1}H-NMR of compound **4v** ($CDCl_3$, 400 MHz).

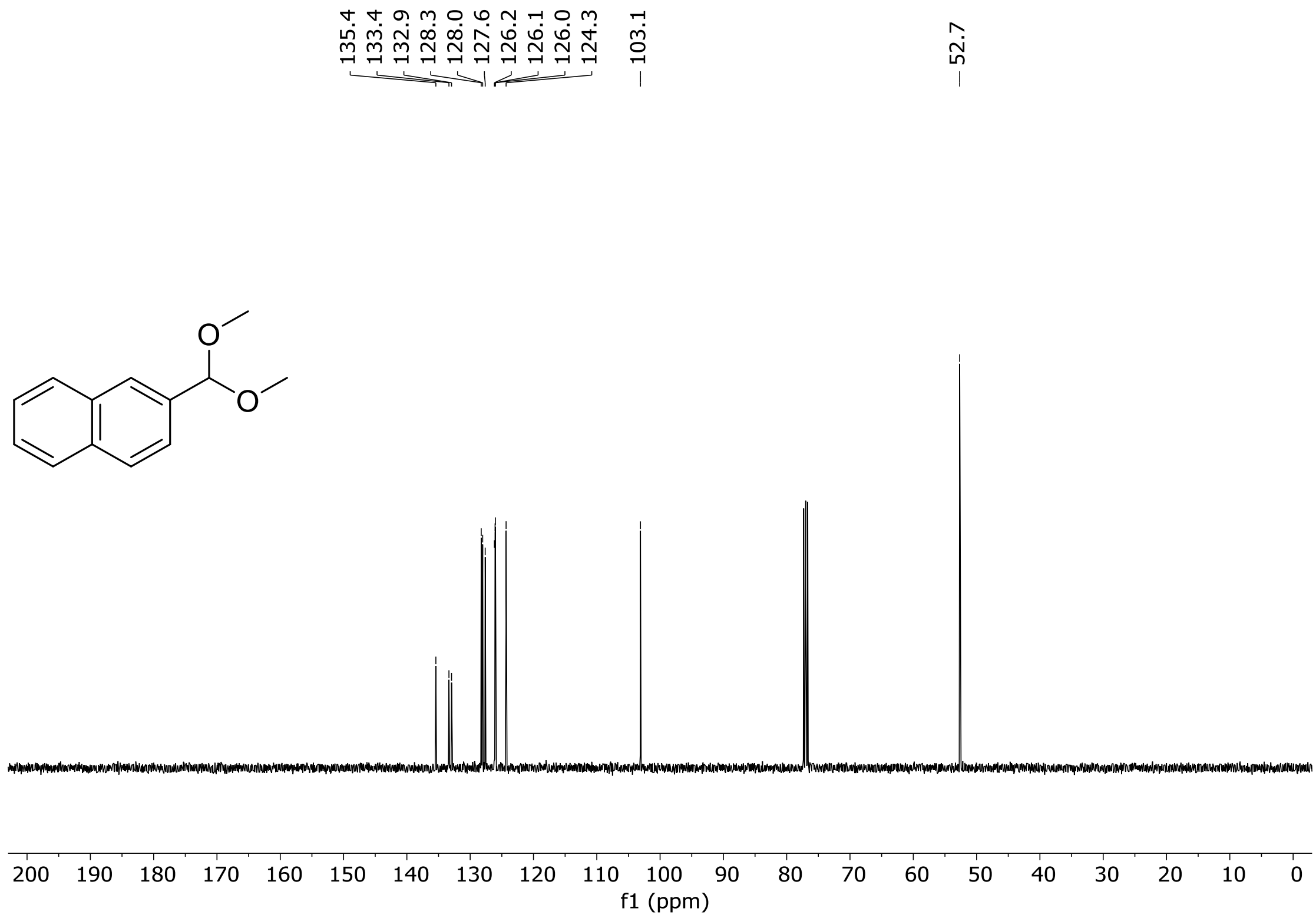

^{13}C-NMR of compound **4v** ($CDCl_3$, 100 MHz).

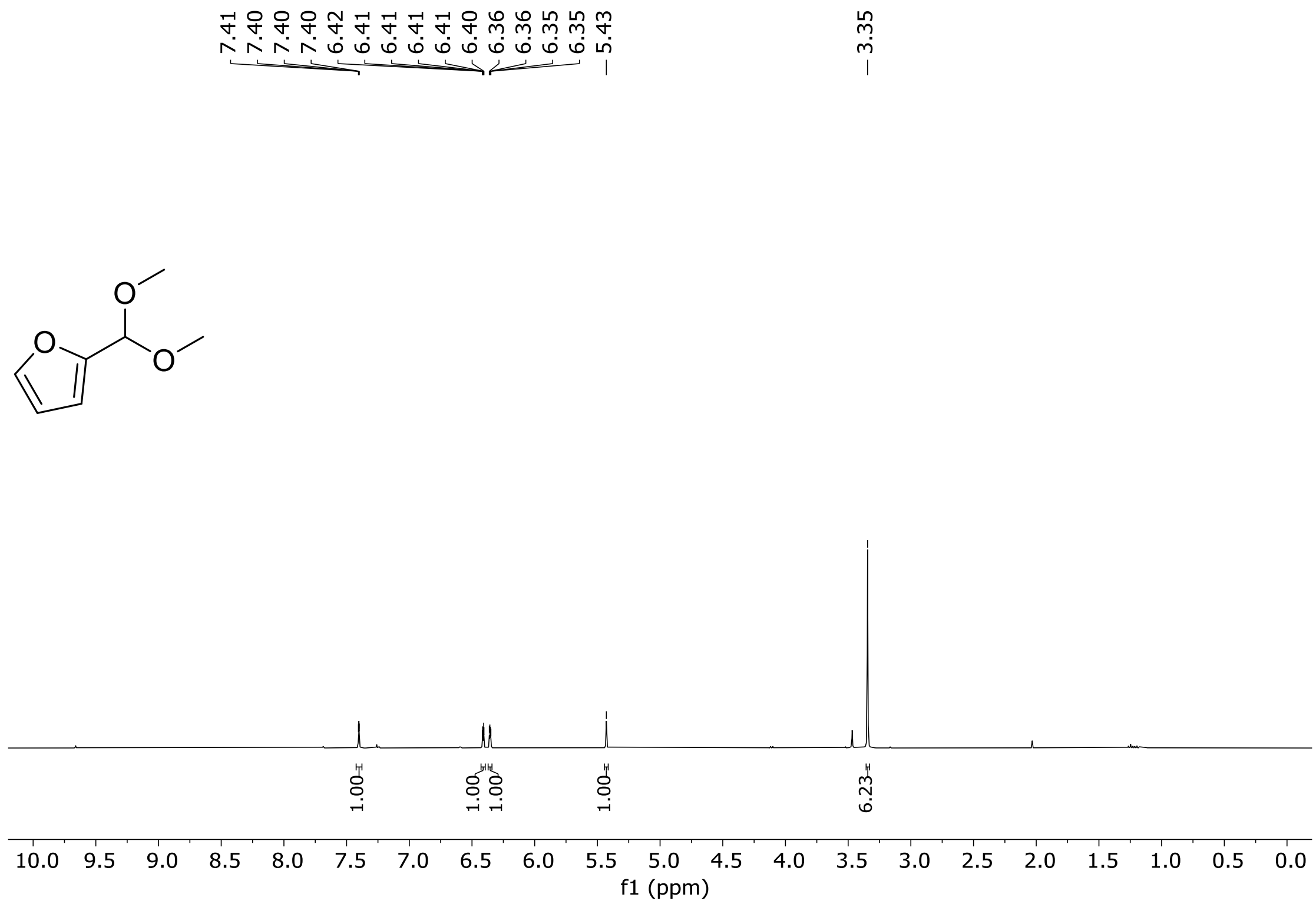

^{1}H-NMR of compound **4w** ($CDCl_3$, 400 MHz).

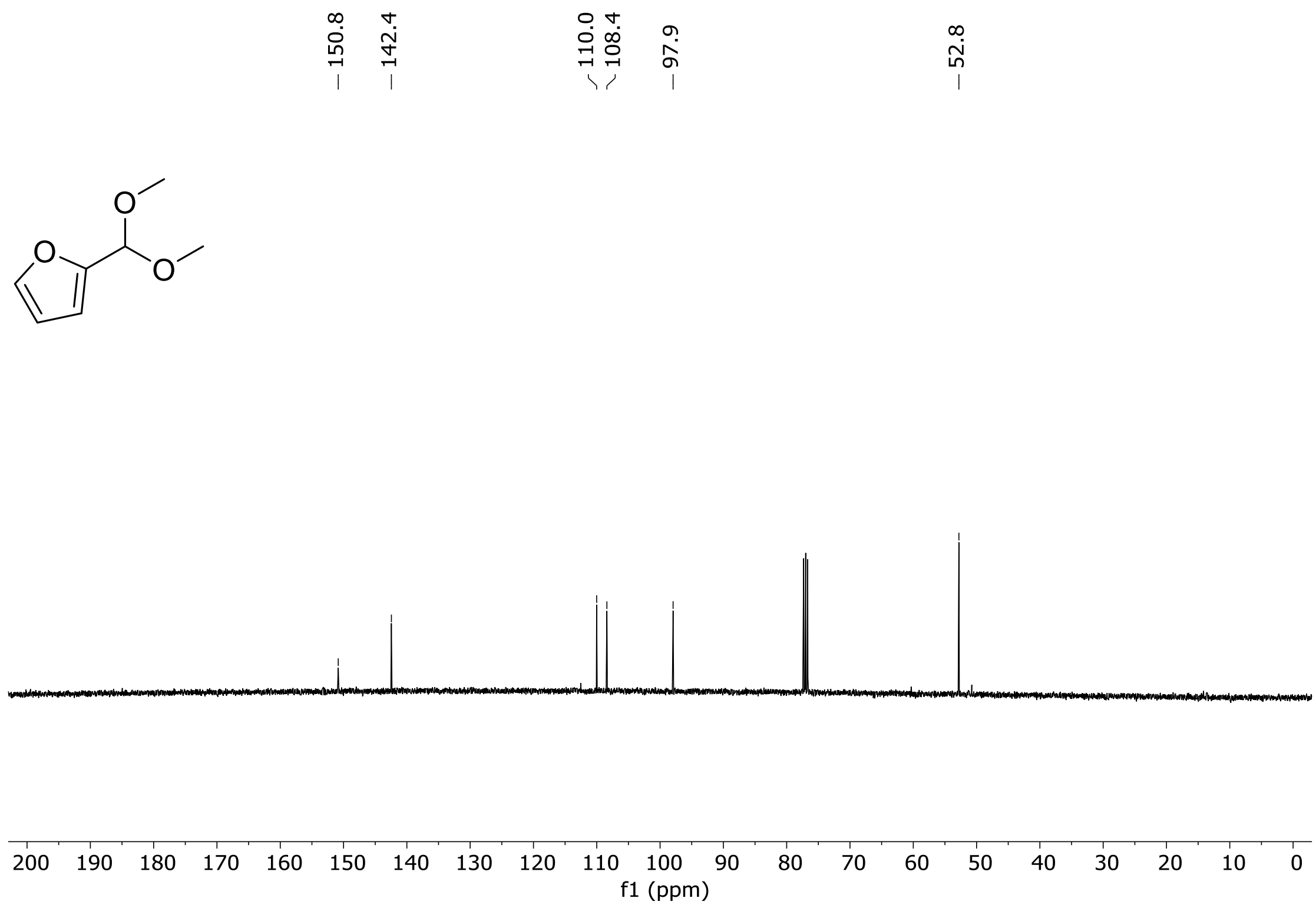

^{13}C-NMR of compound **4w** ($CDCl_3$, 100 MHz).

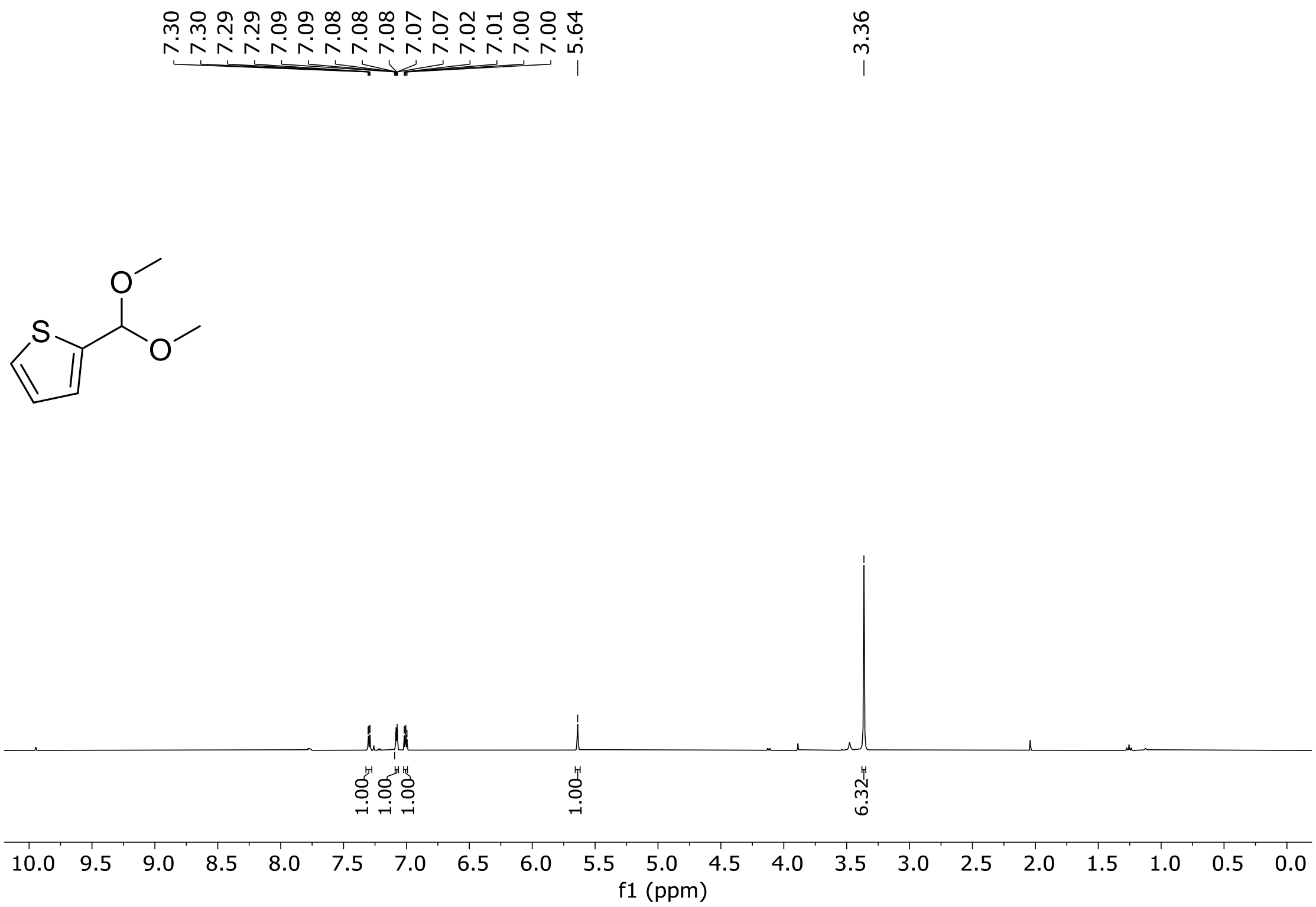

^{1}H-NMR of compound **4x** ($CDCl_3$, 400 MHz).

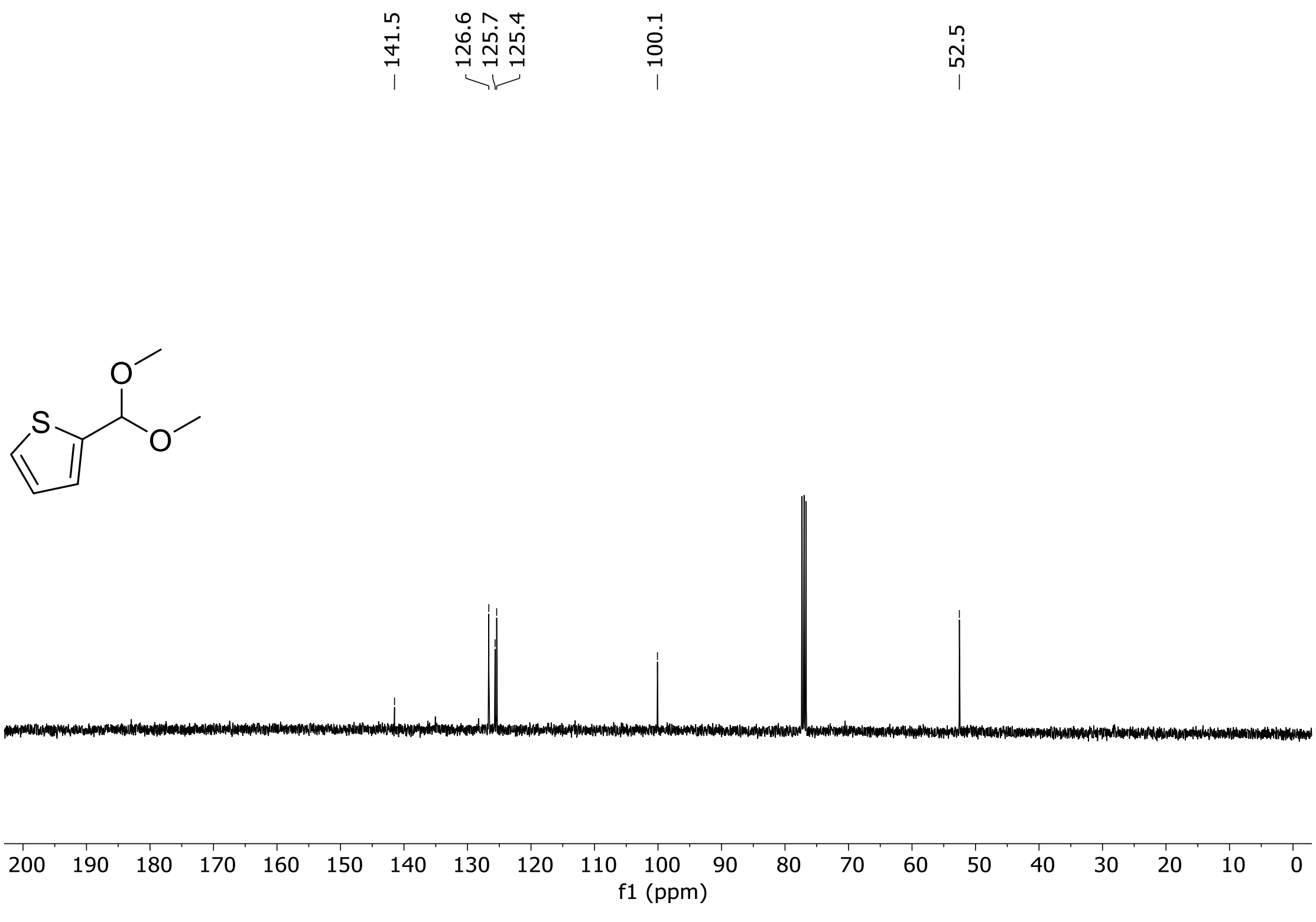

^{13}C-NMR of compound **4x** ($CDCl_3$, 100 MHz).

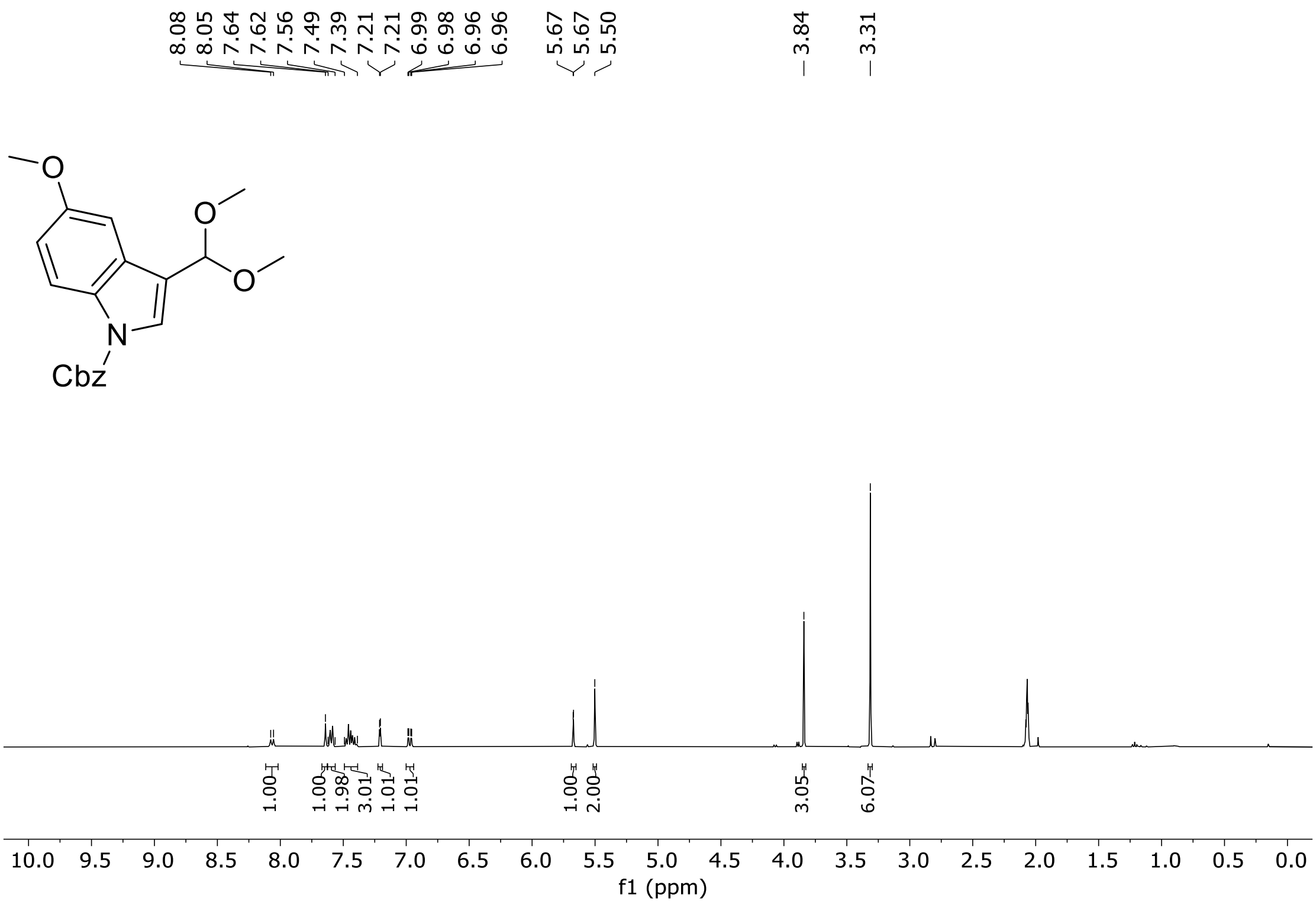

^{1}H-NMR of compound **4y** (Acetone-d_6, 400 MHz).

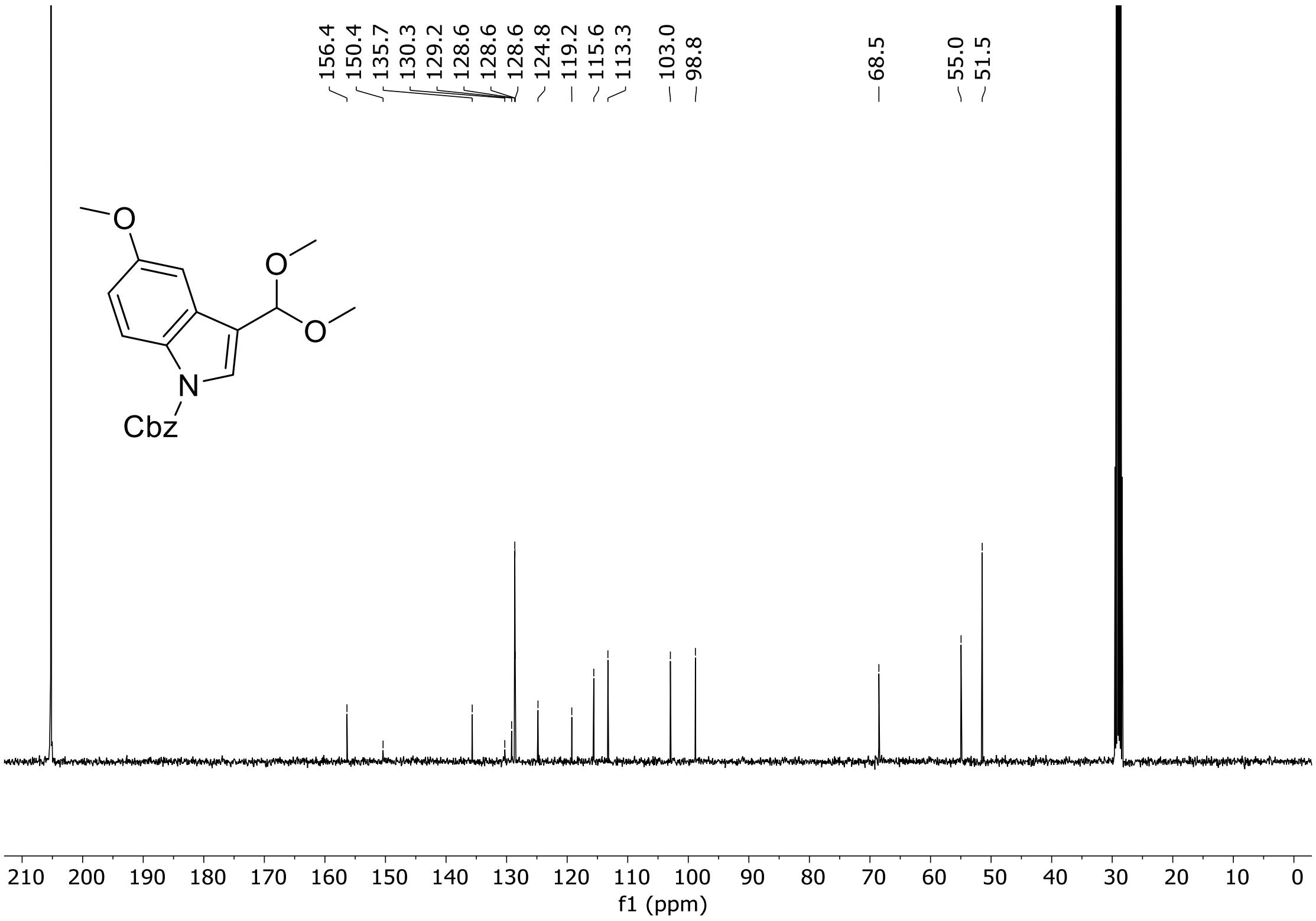

^{13}C-NMR of compound **4y** (Acetone-d_6, 100 MHz).

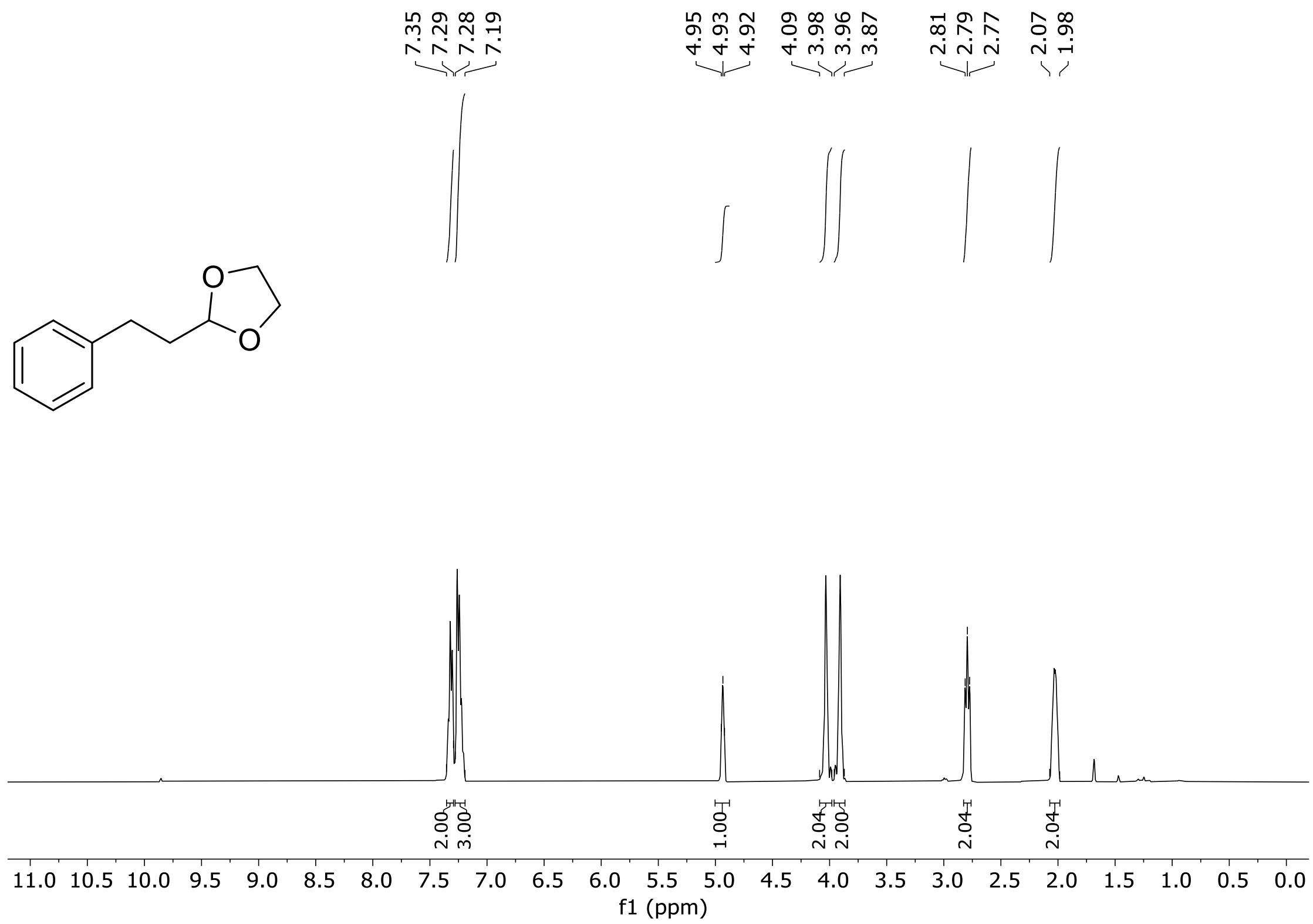

^{1}H-NMR of compound **6a** ($CDCl_3$, 400 MHz).

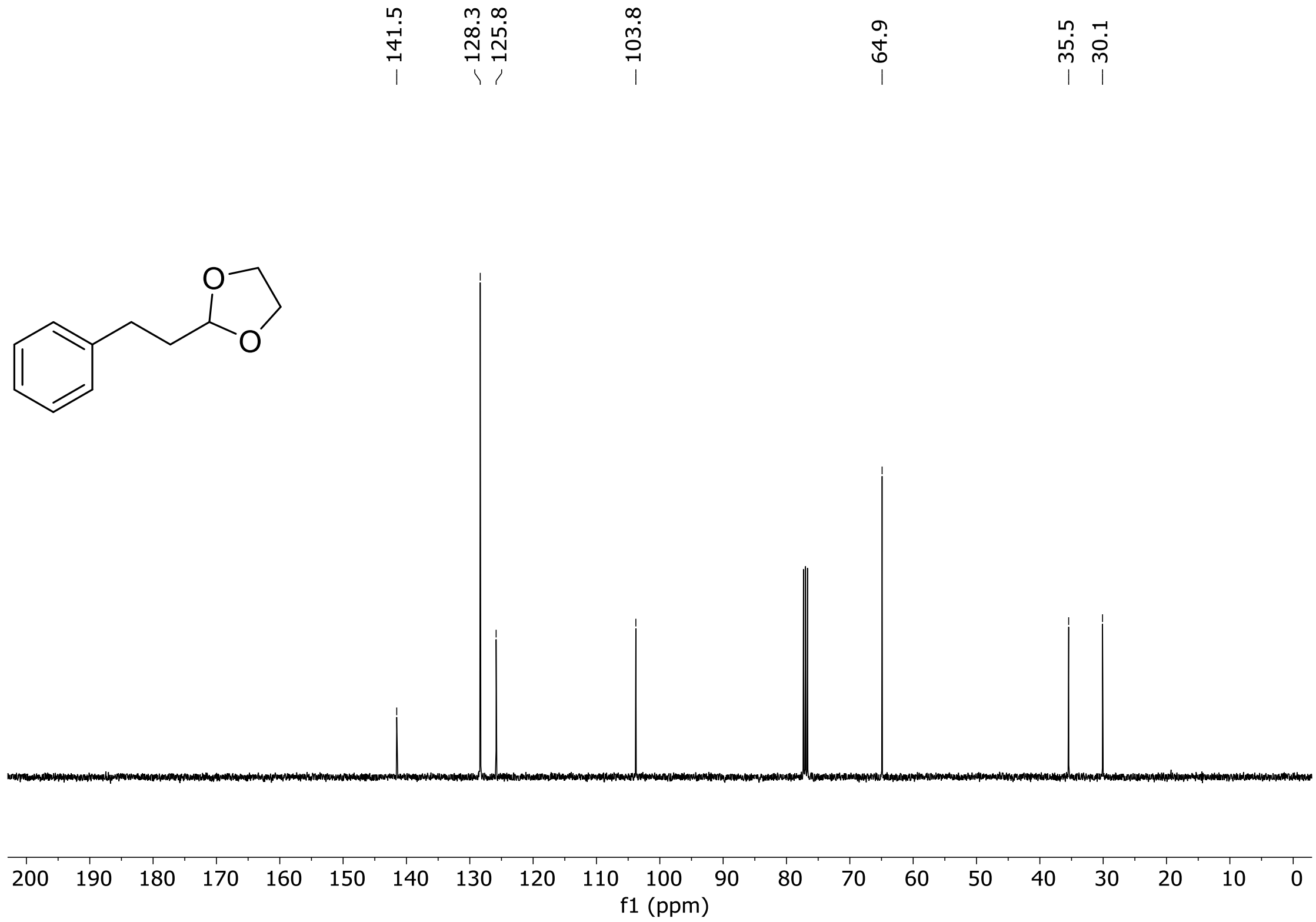

^{13}C-NMR of compound **6a** ($CDCl_3$, 100 MHz).

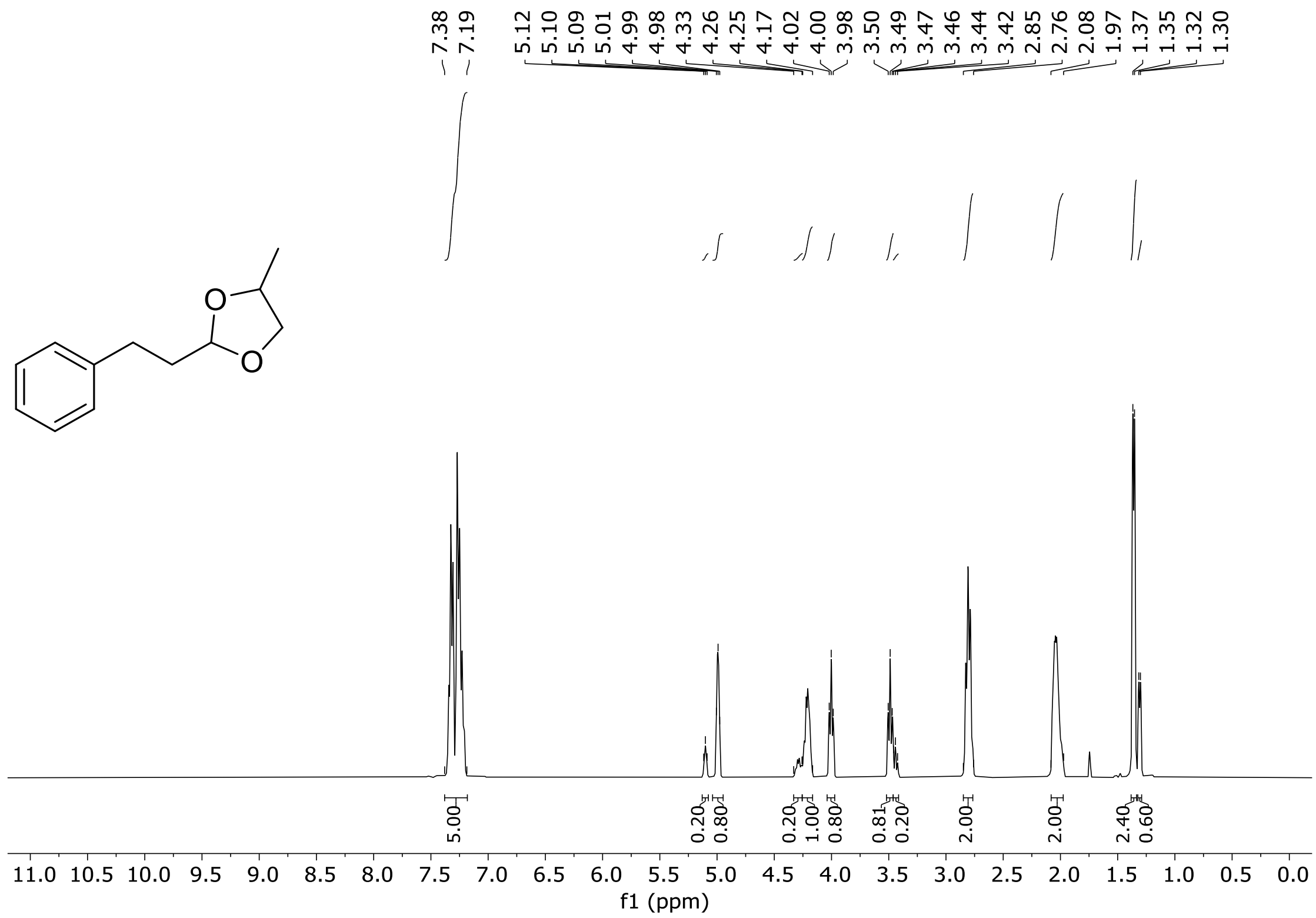

^{1}H-NMR of compound **6b** ($CDCl_3$, 400 MHz).

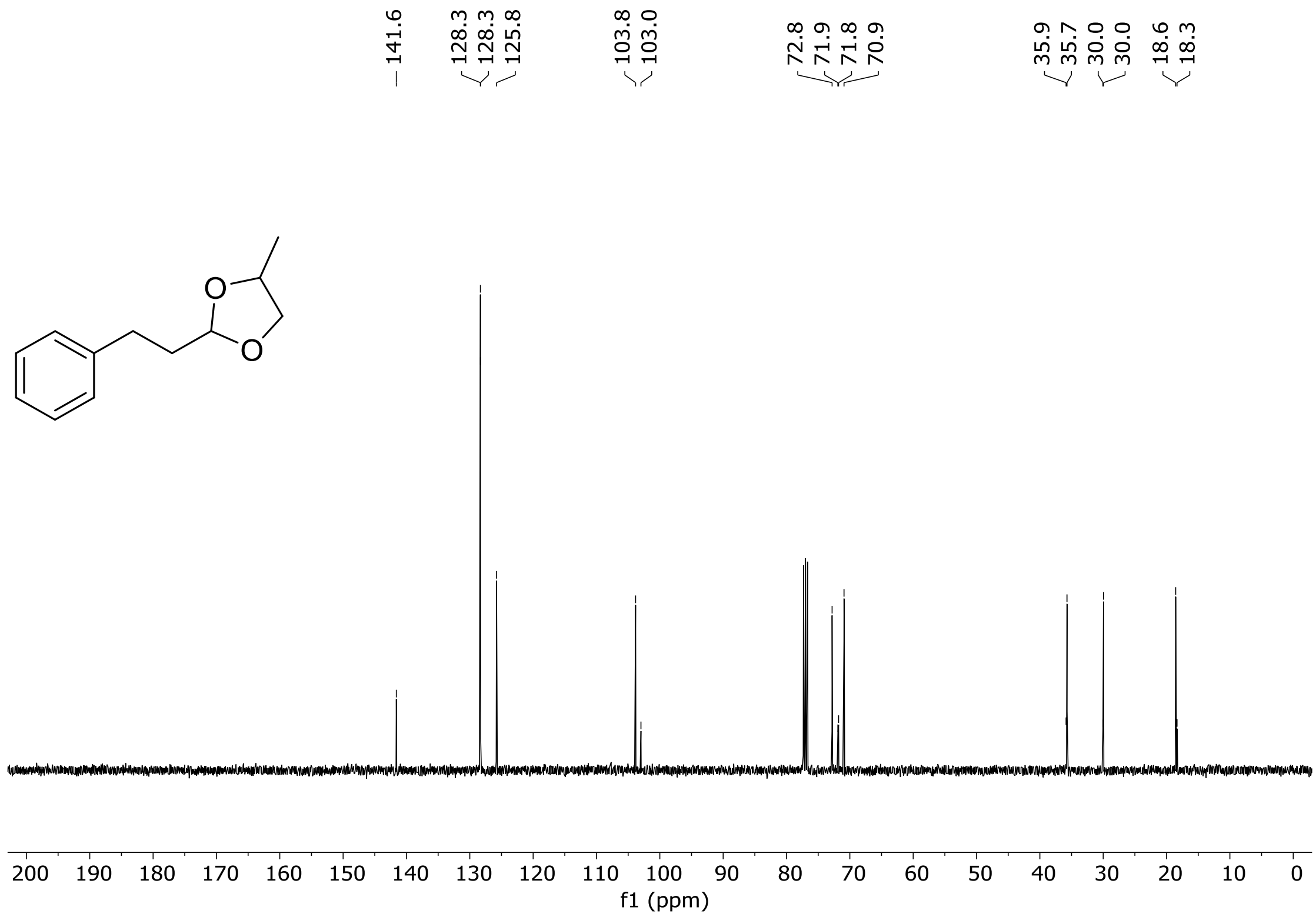

^{13}C-NMR of compound **6b** ($CDCl_3$, 100 MHz).

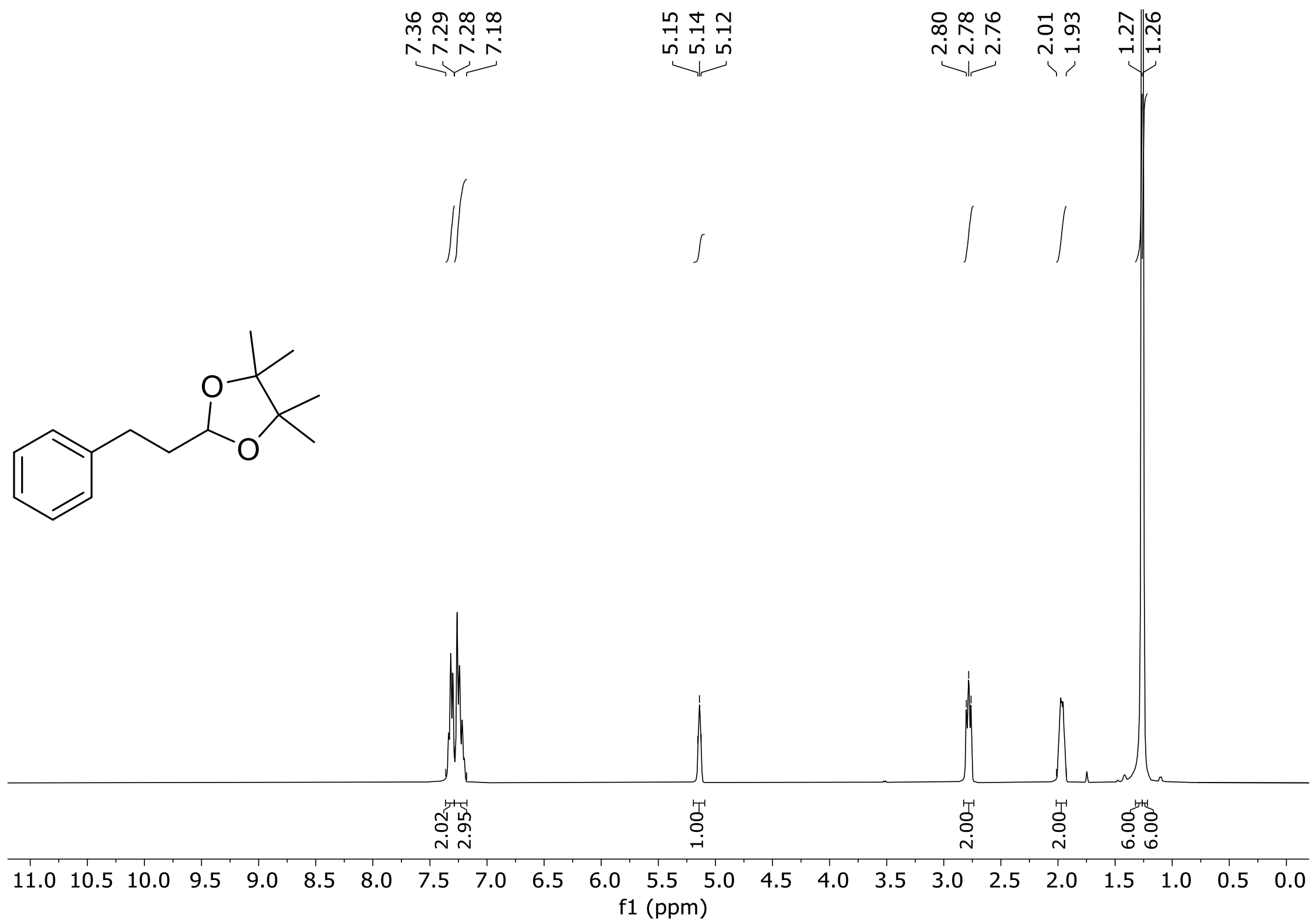

^{1}H-NMR of compound **6c** ($CDCl_3$, 400 MHz).

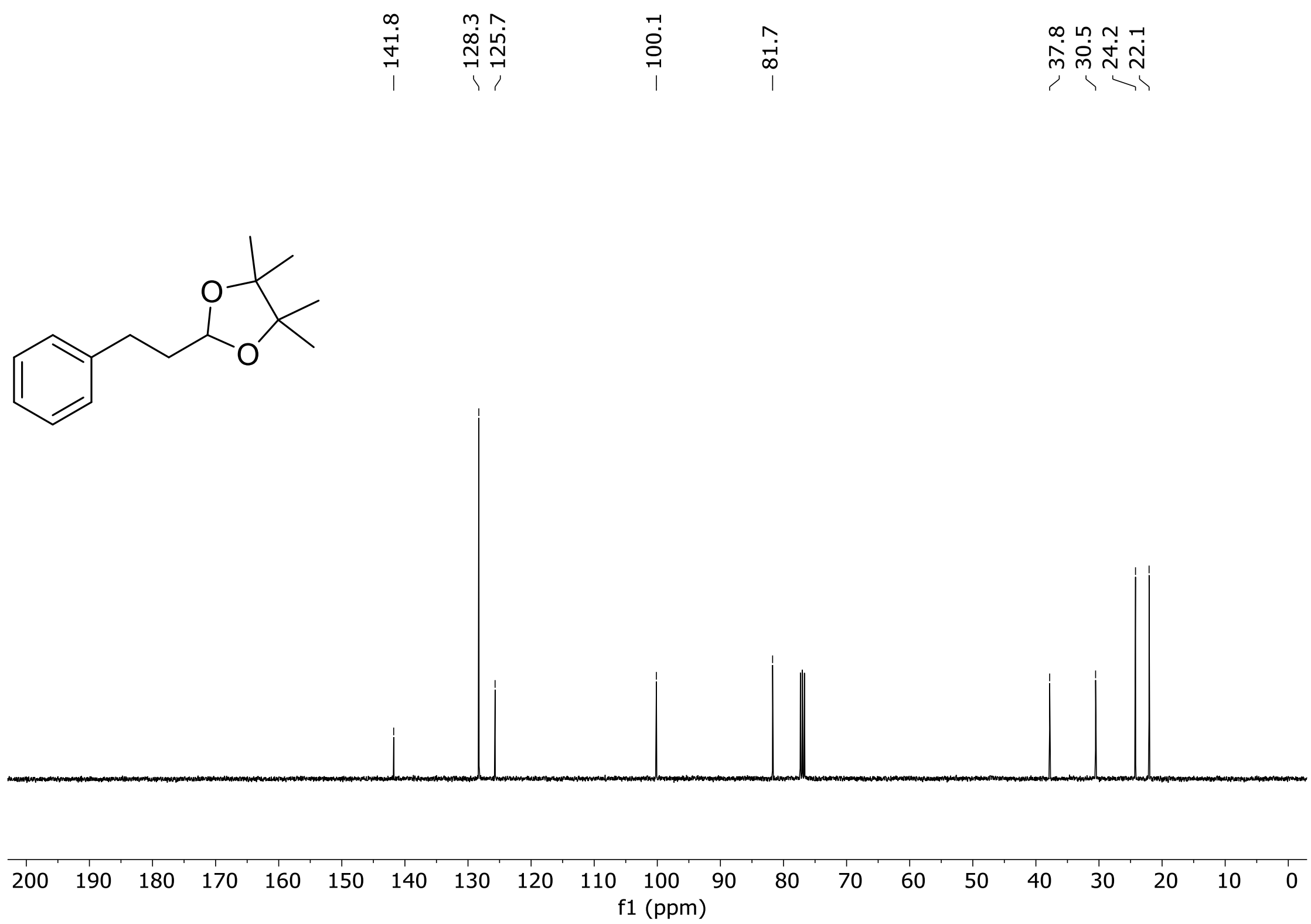

^{13}C-NMR of compound **6c** ($CDCl_3$, 100 MHz).

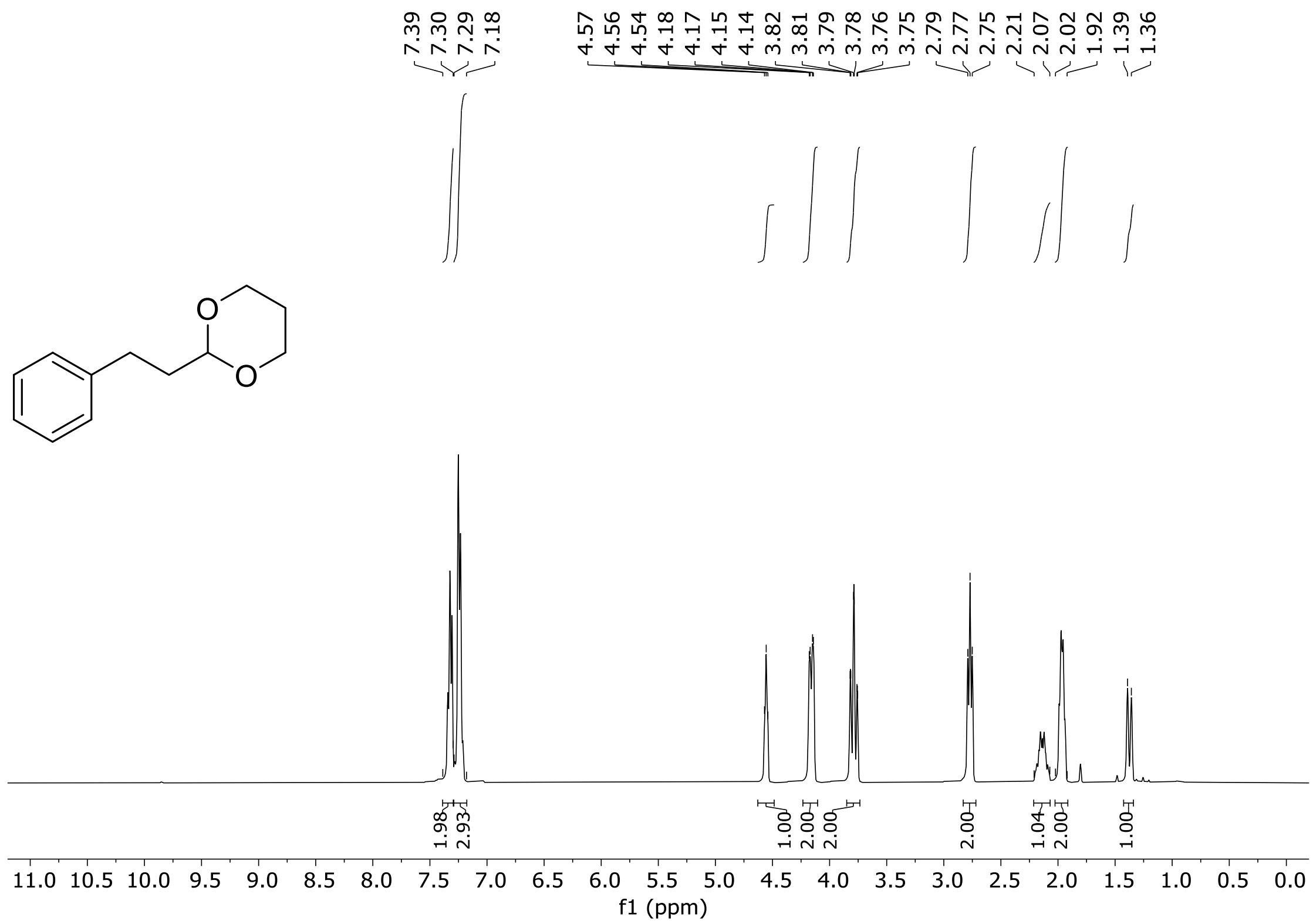

^{1}H-NMR of compound **6d** ($CDCl_3$, 400 MHz).

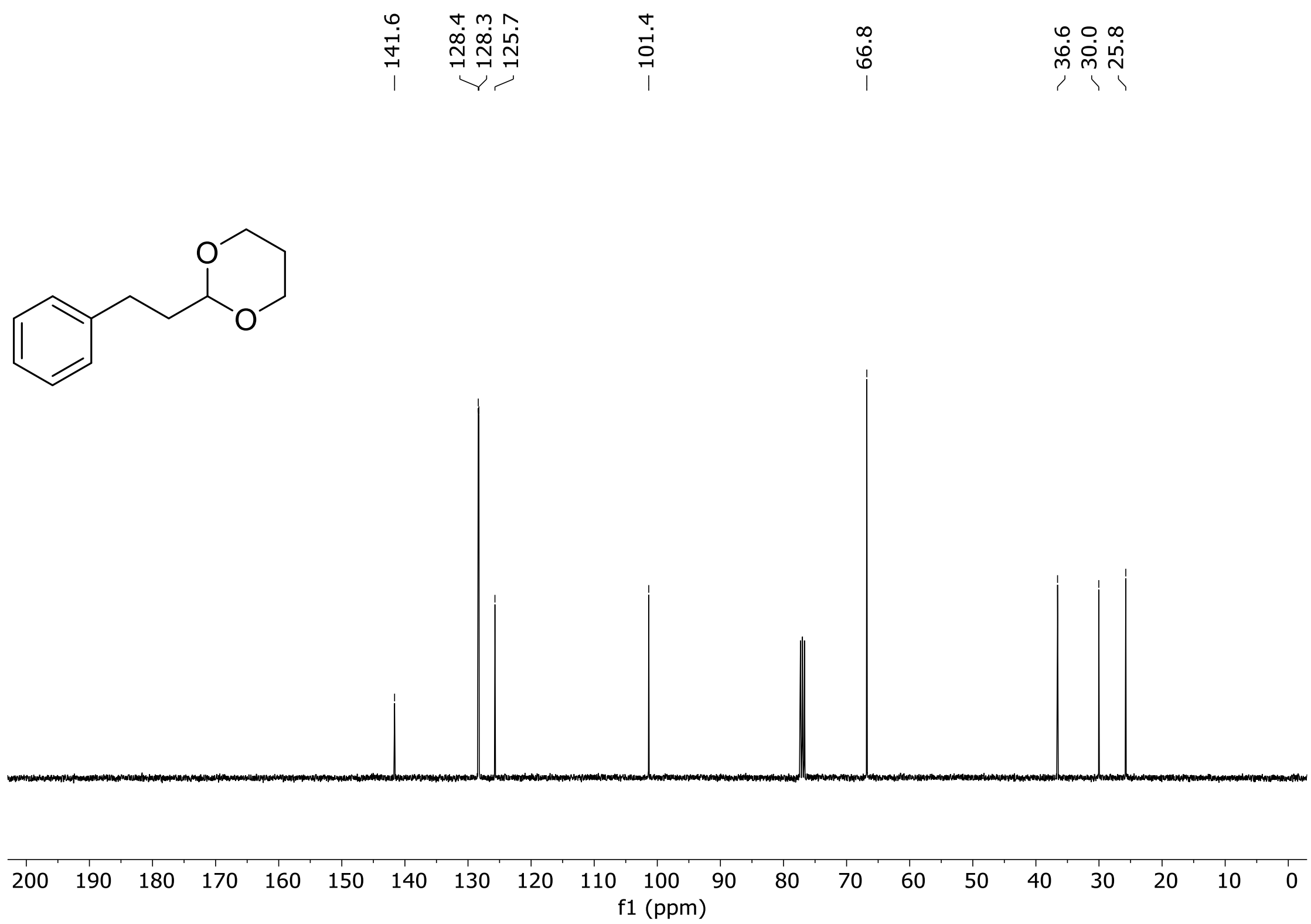

^{13}C-NMR of compound **6d** ($CDCl_3$, 100 MHz).

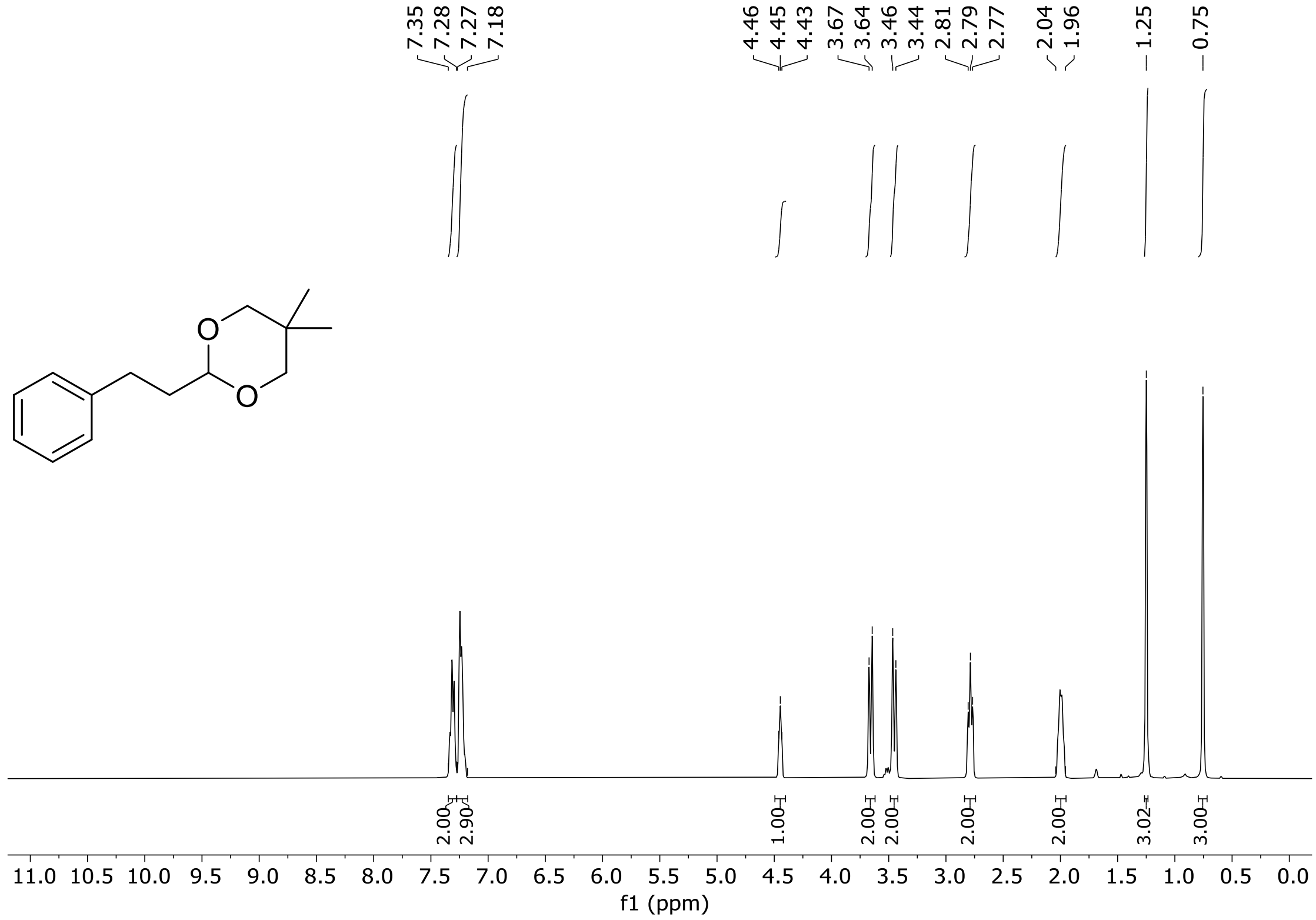

^{1}H-NMR of compound **6e** ($CDCl_3$, 400 MHz).

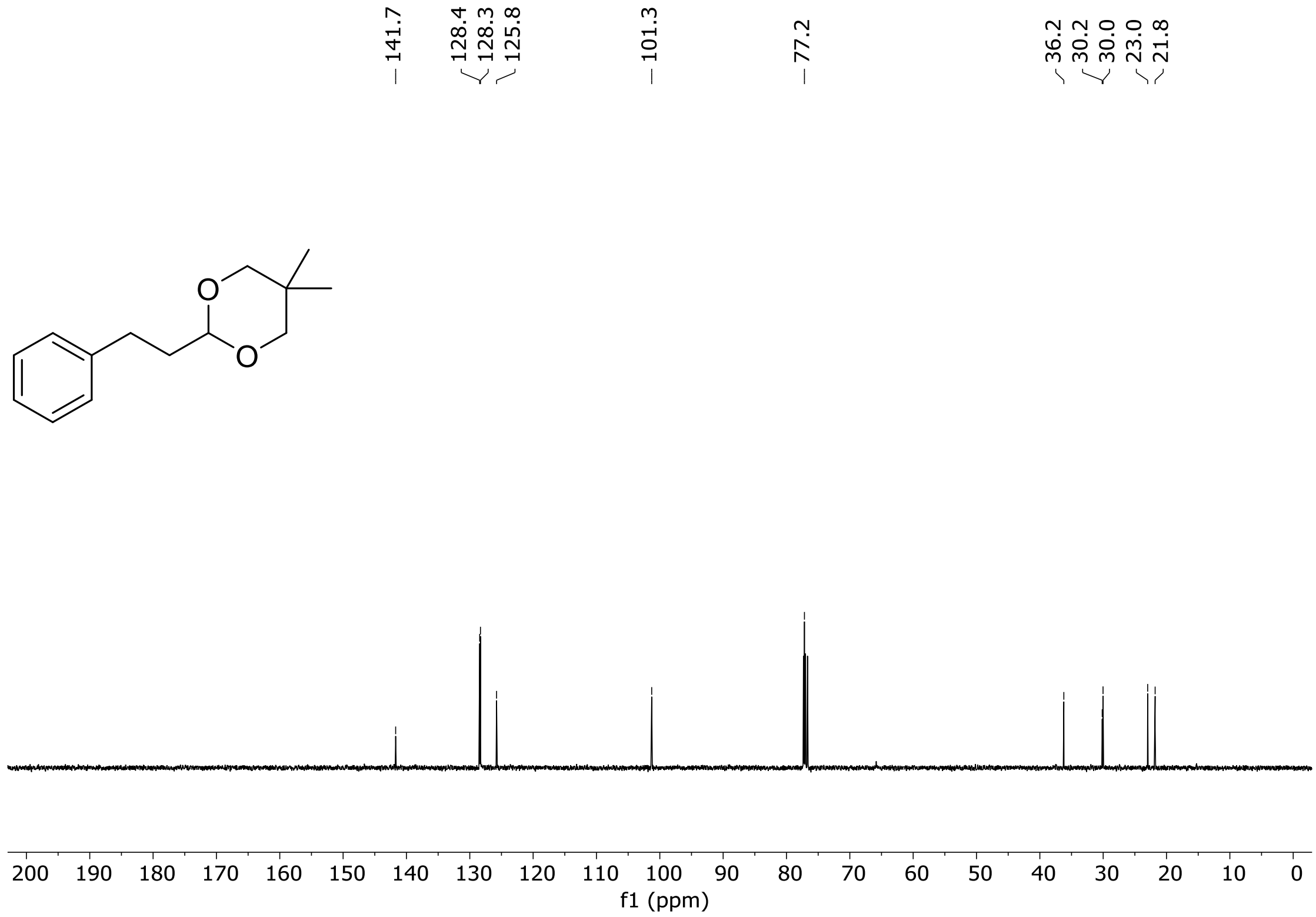

^{13}C-NMR of compound **6e** ($CDCl_3$, 100 MHz).

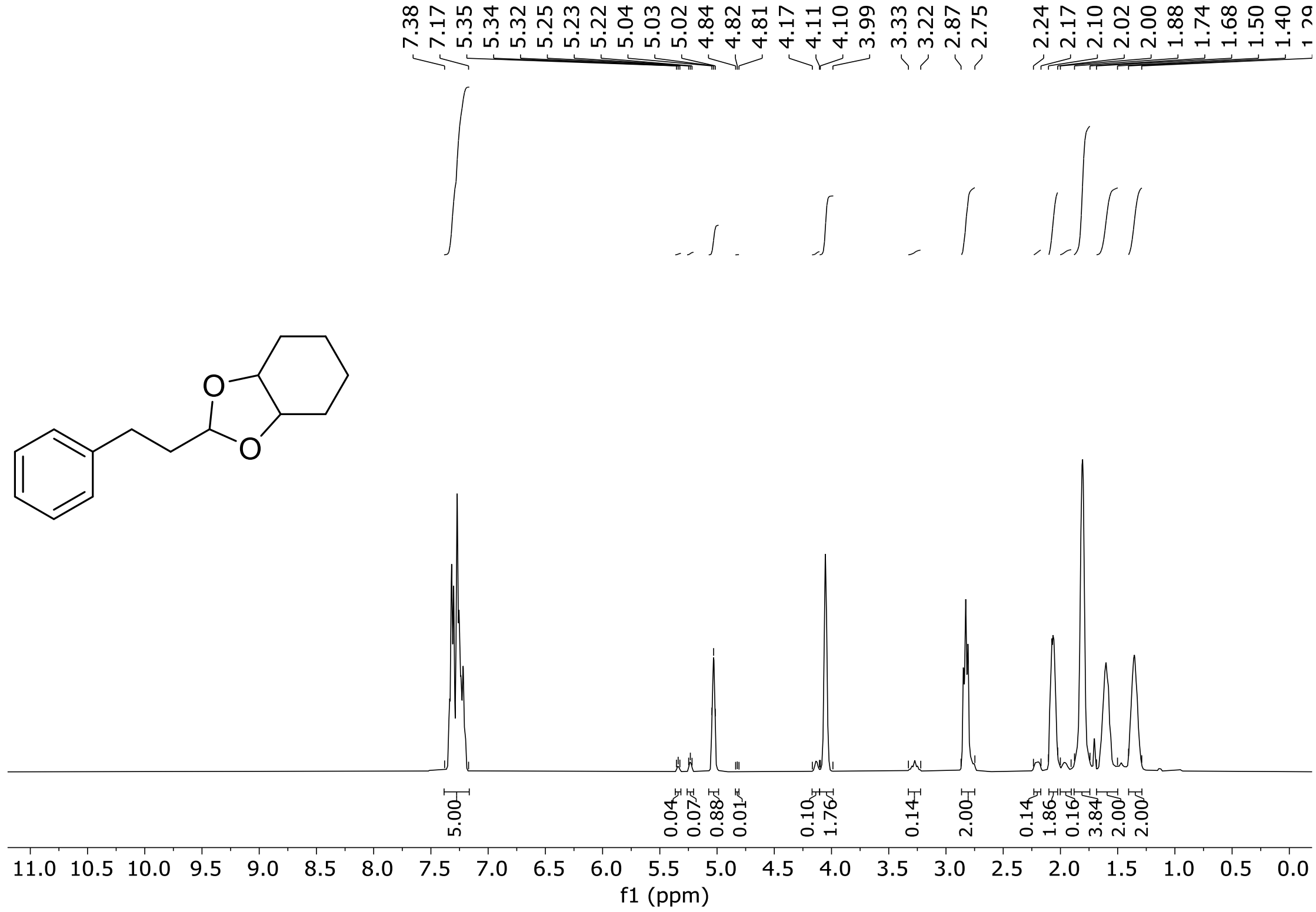

^{1}H-NMR of compound **6f** ($CDCl_3$, 400 MHz).

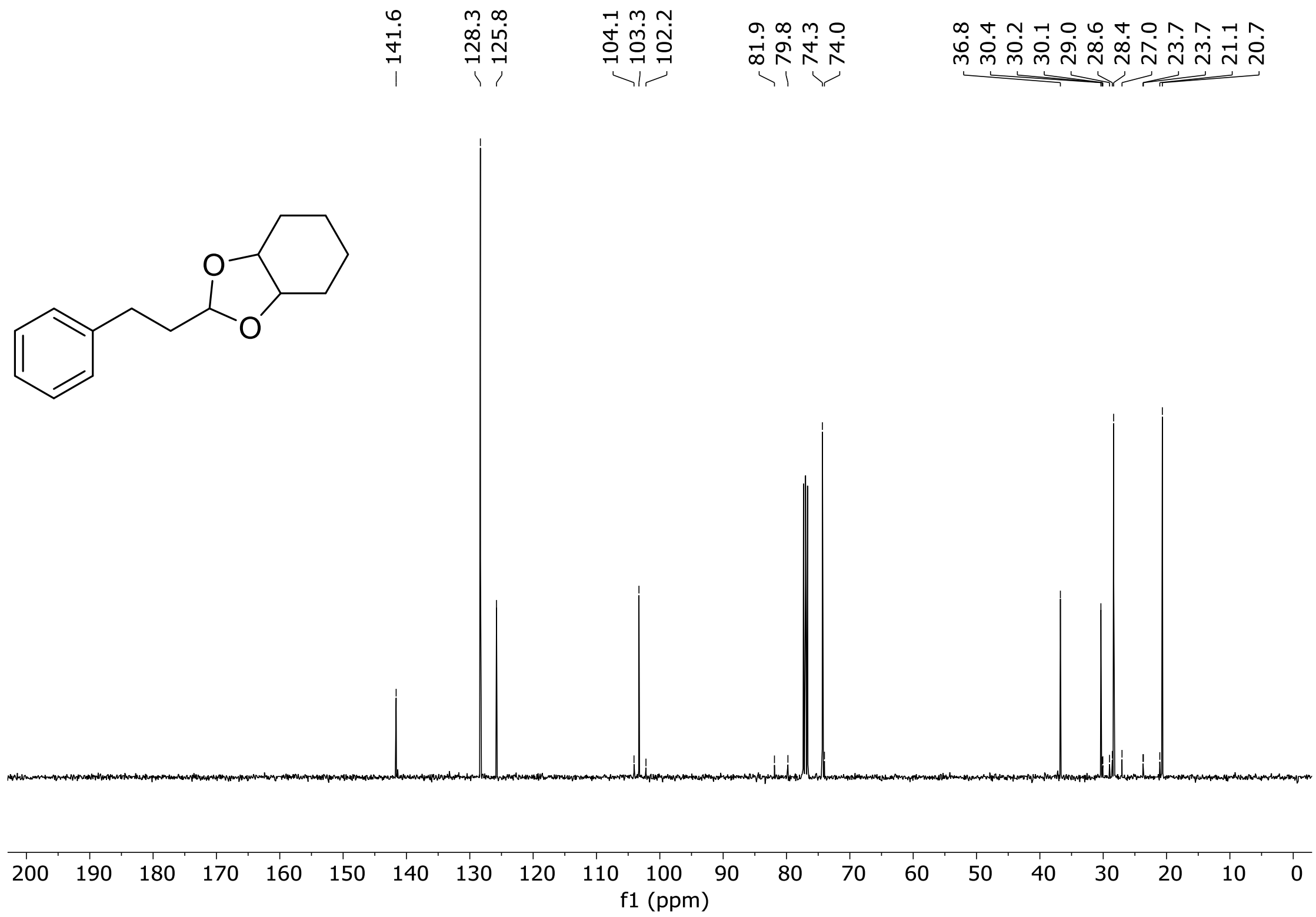

^{13}C-NMR of compound **6f** ($CDCl_3$, 100 MHz).

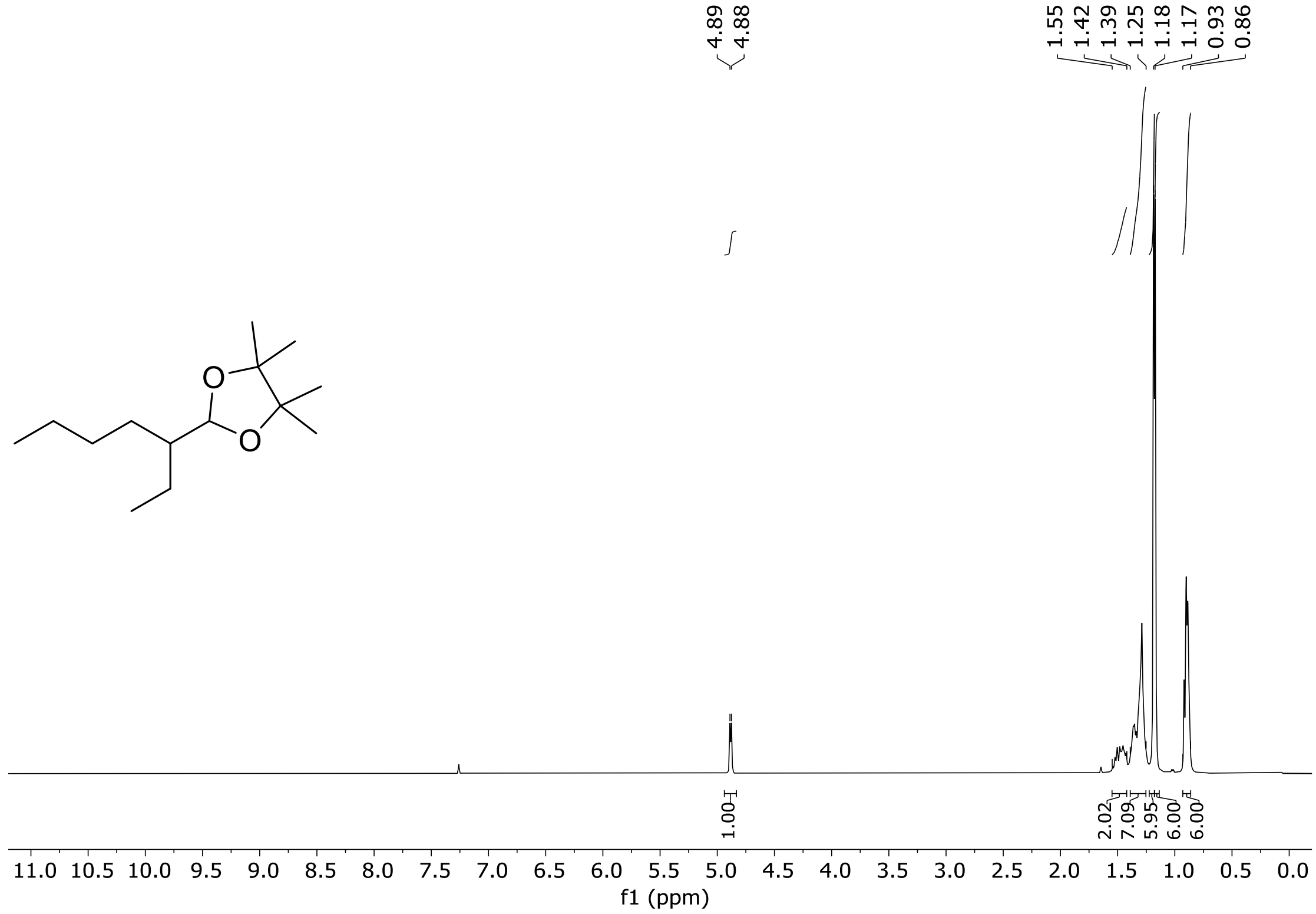

^{1}H-NMR of compound **6g** ($CDCl_3$, 400 MHz).

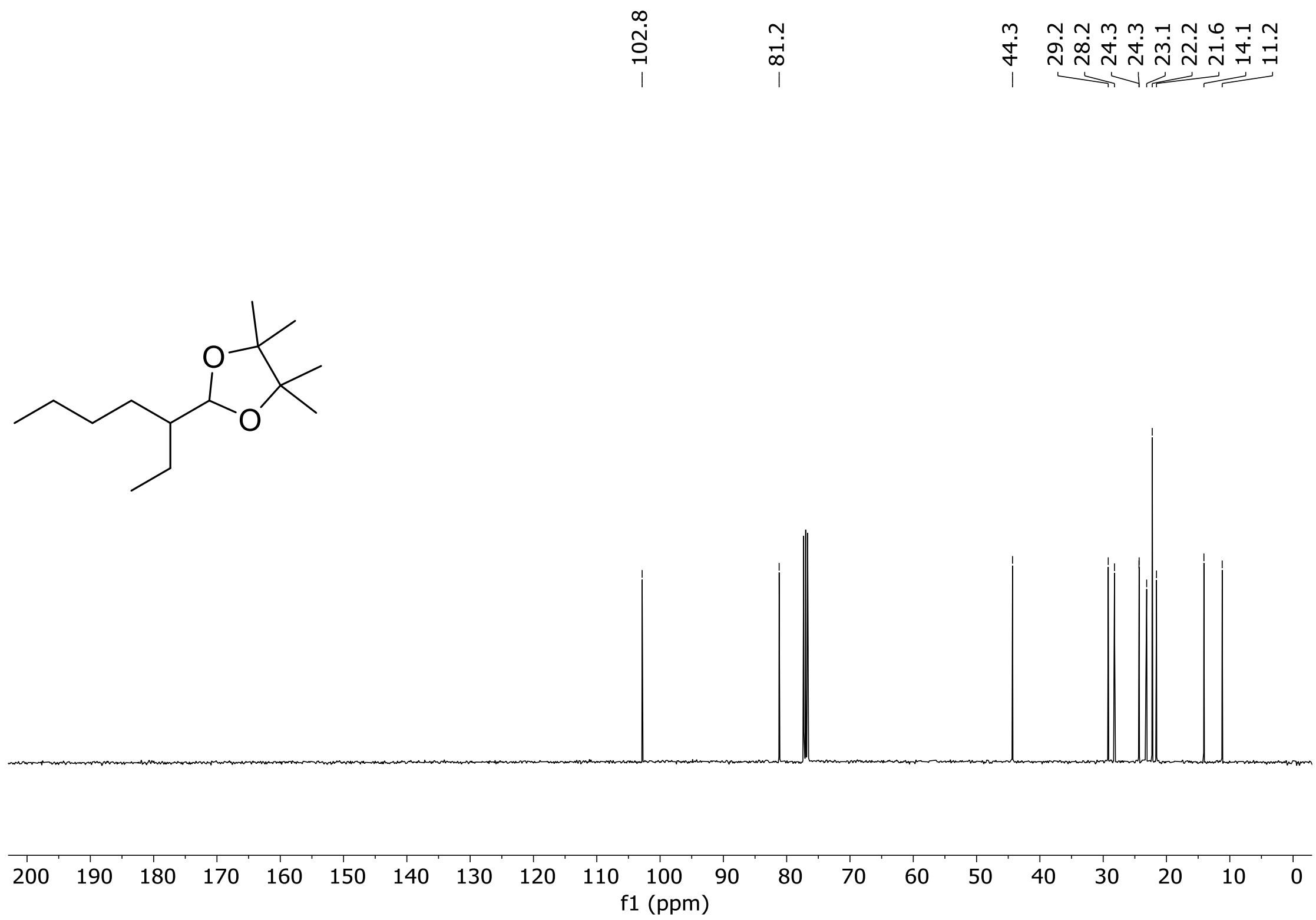

^{13}C-NMR of compound **6g** ($CDCl_3$, 100 MHz).

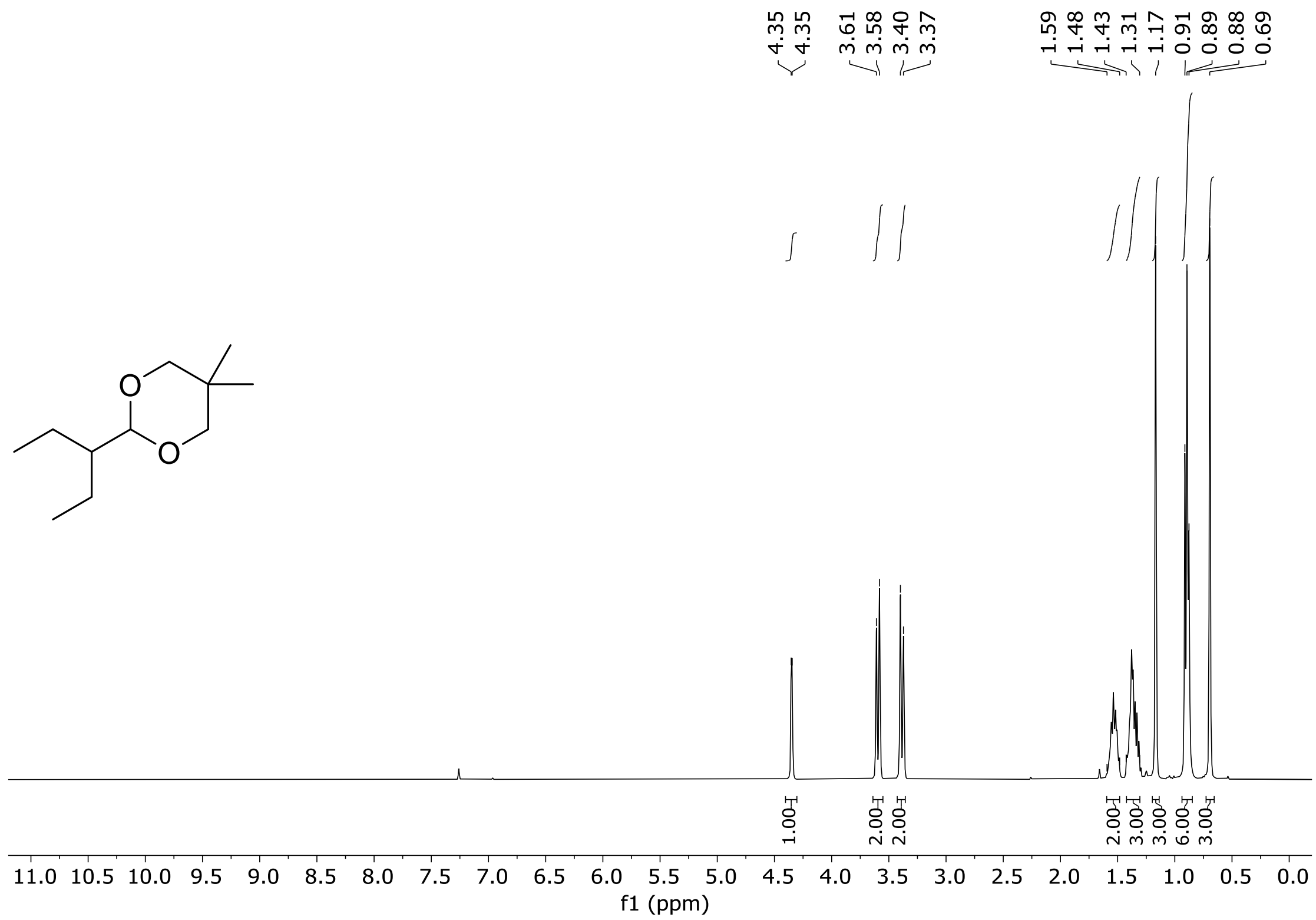

^{1}H-NMR of compound **6h** ($CDCl_3$, 400 MHz).

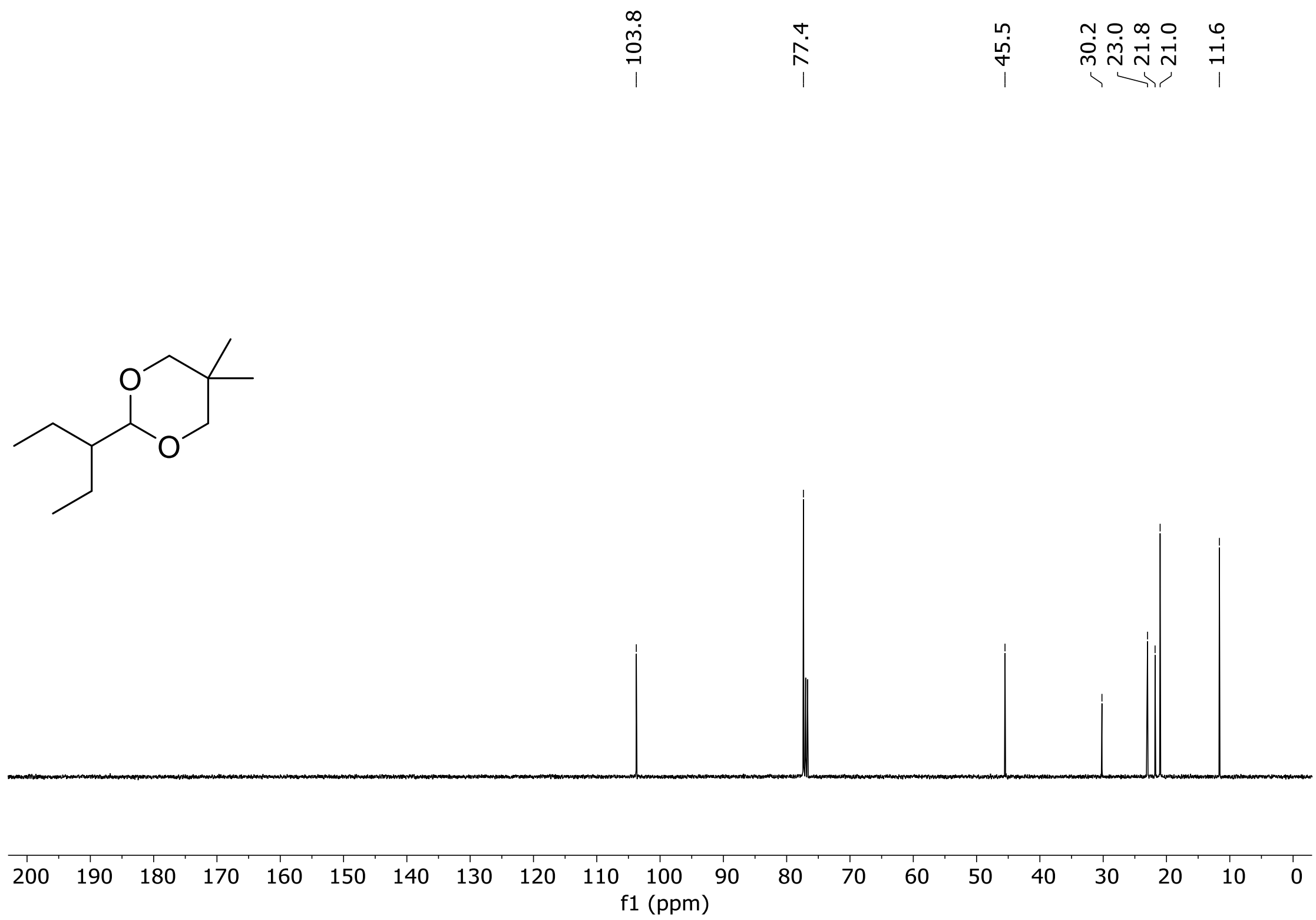

^{13}C-NMR of compound **6h** ($CDCl_3$, 100 MHz).

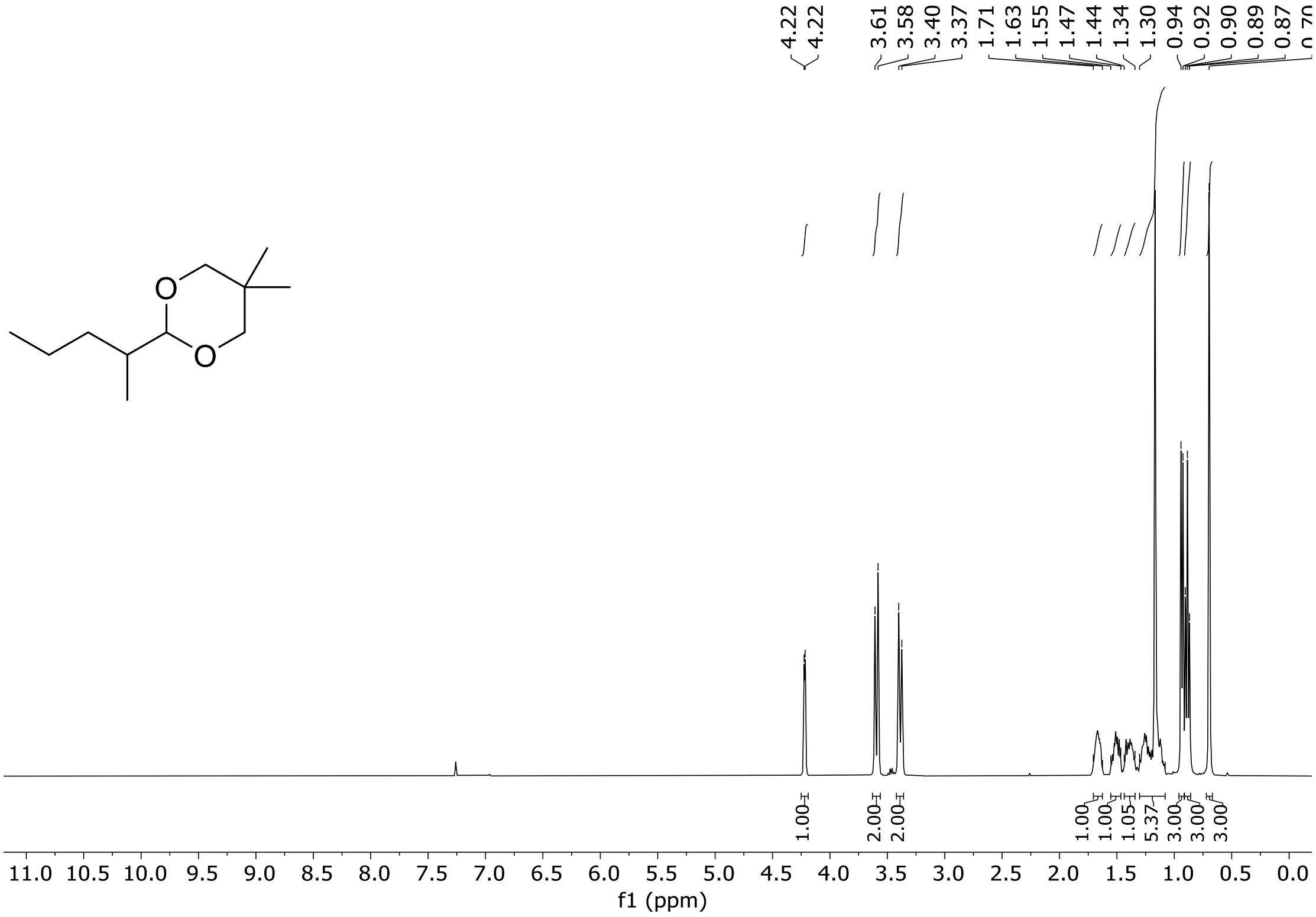

^{1}H-NMR of compound **6i** ($CDCl_3$, 400 MHz).

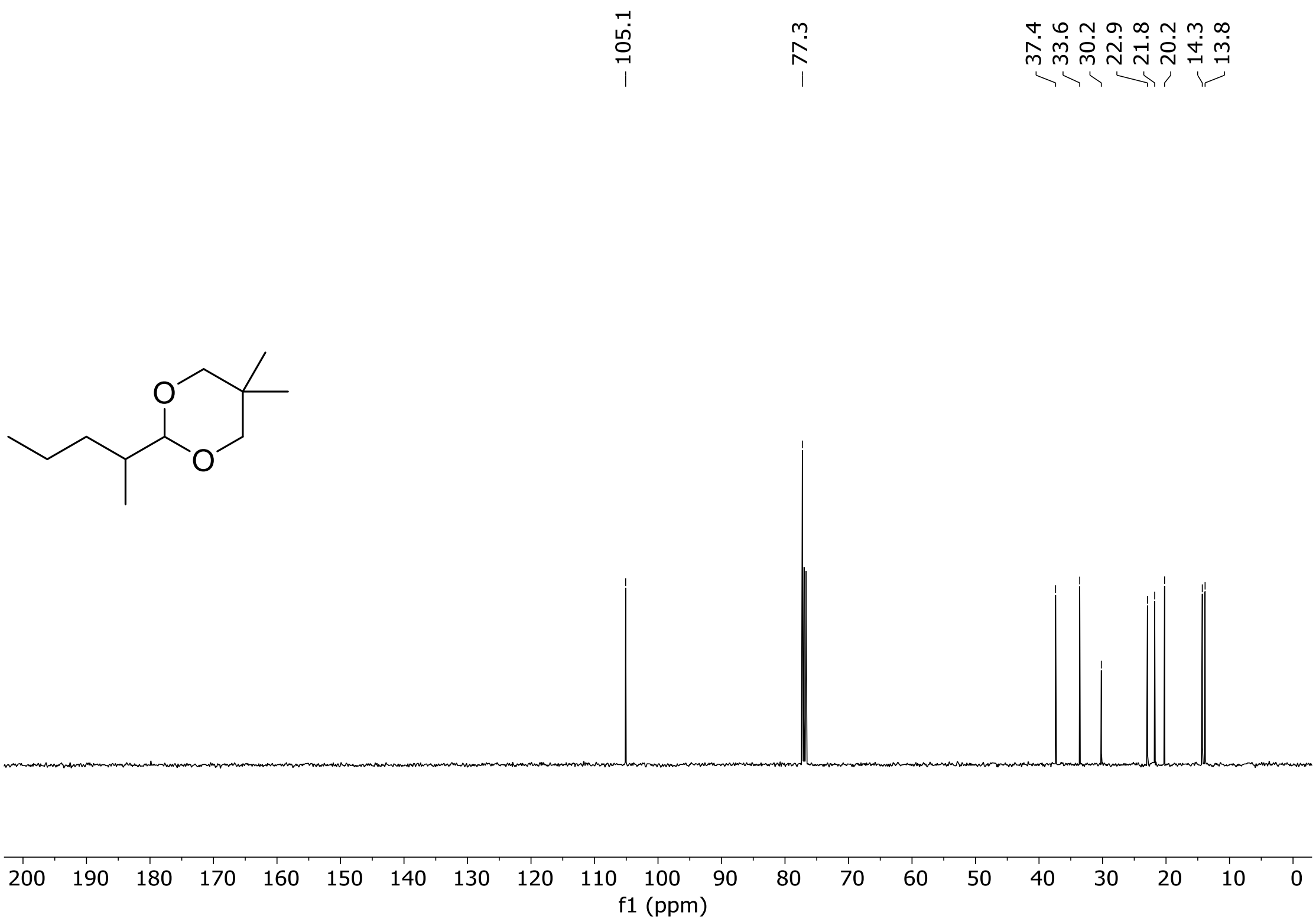

^{13}C-NMR of compound **6i** ($CDCl_3$, 100 MHz).

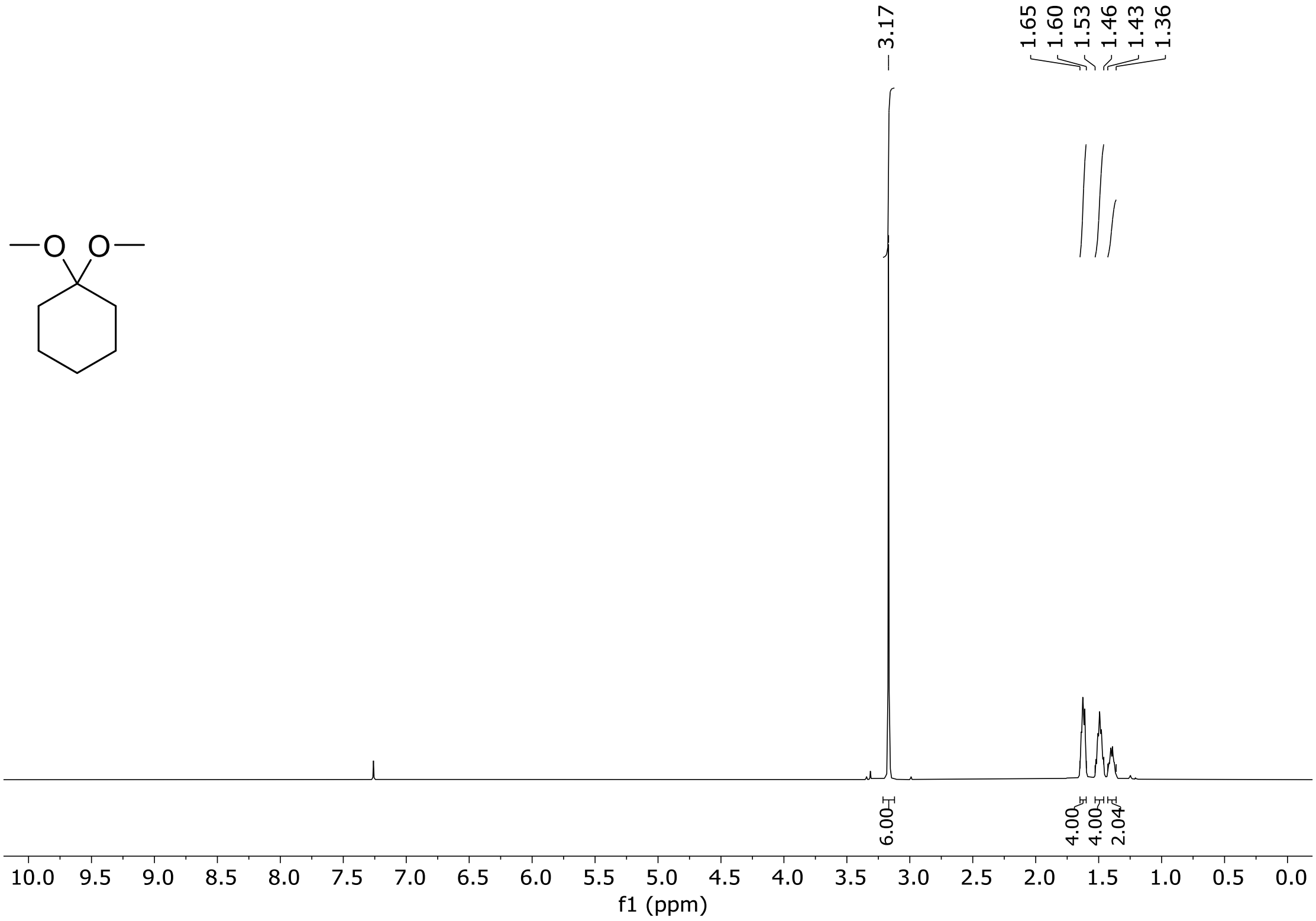

^{1}H-NMR of compound **8a** ($CDCl_3$, 400 MHz).

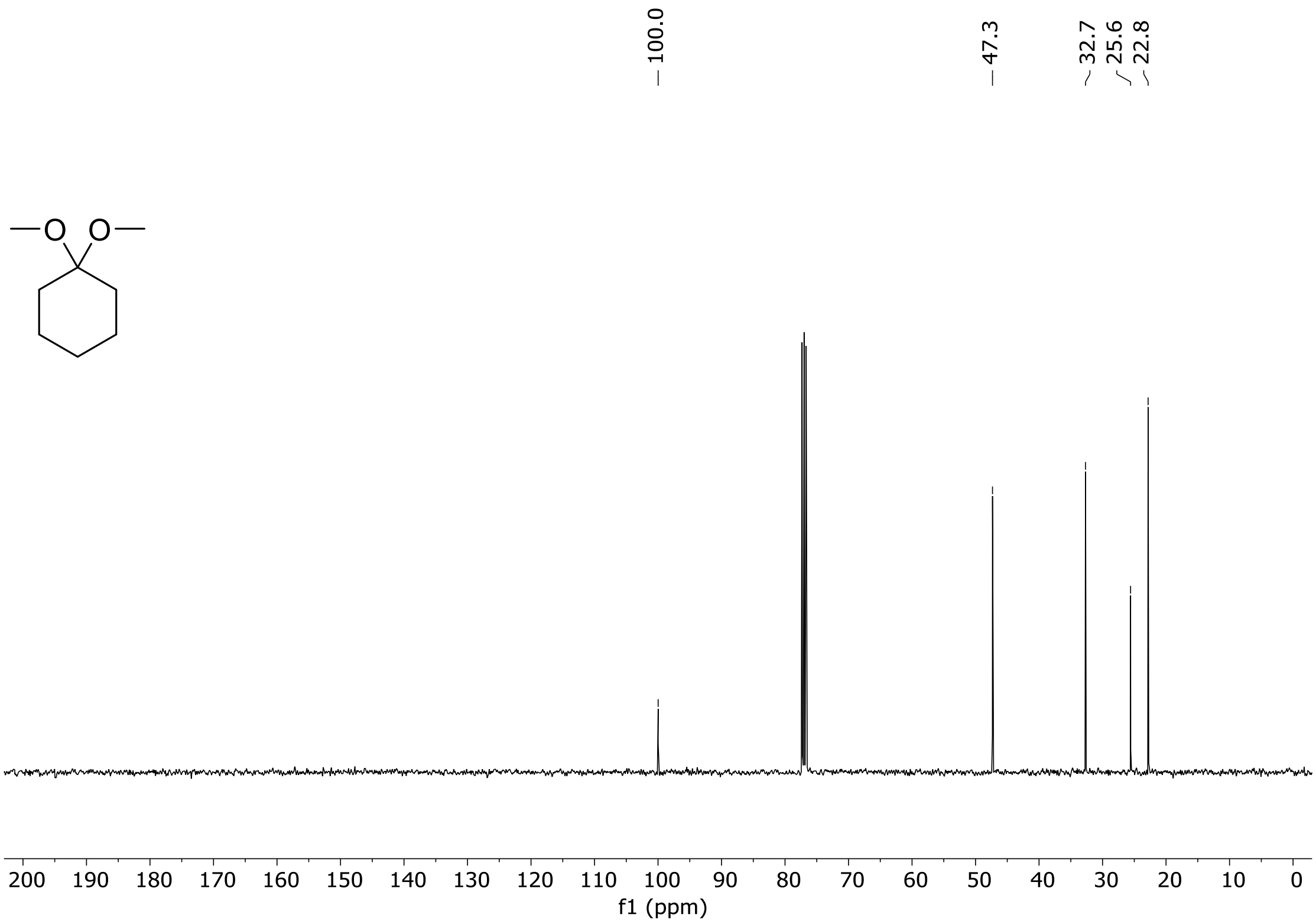

^{13}C-NMR of compound **8a** ($CDCl_3$, 100 MHz).

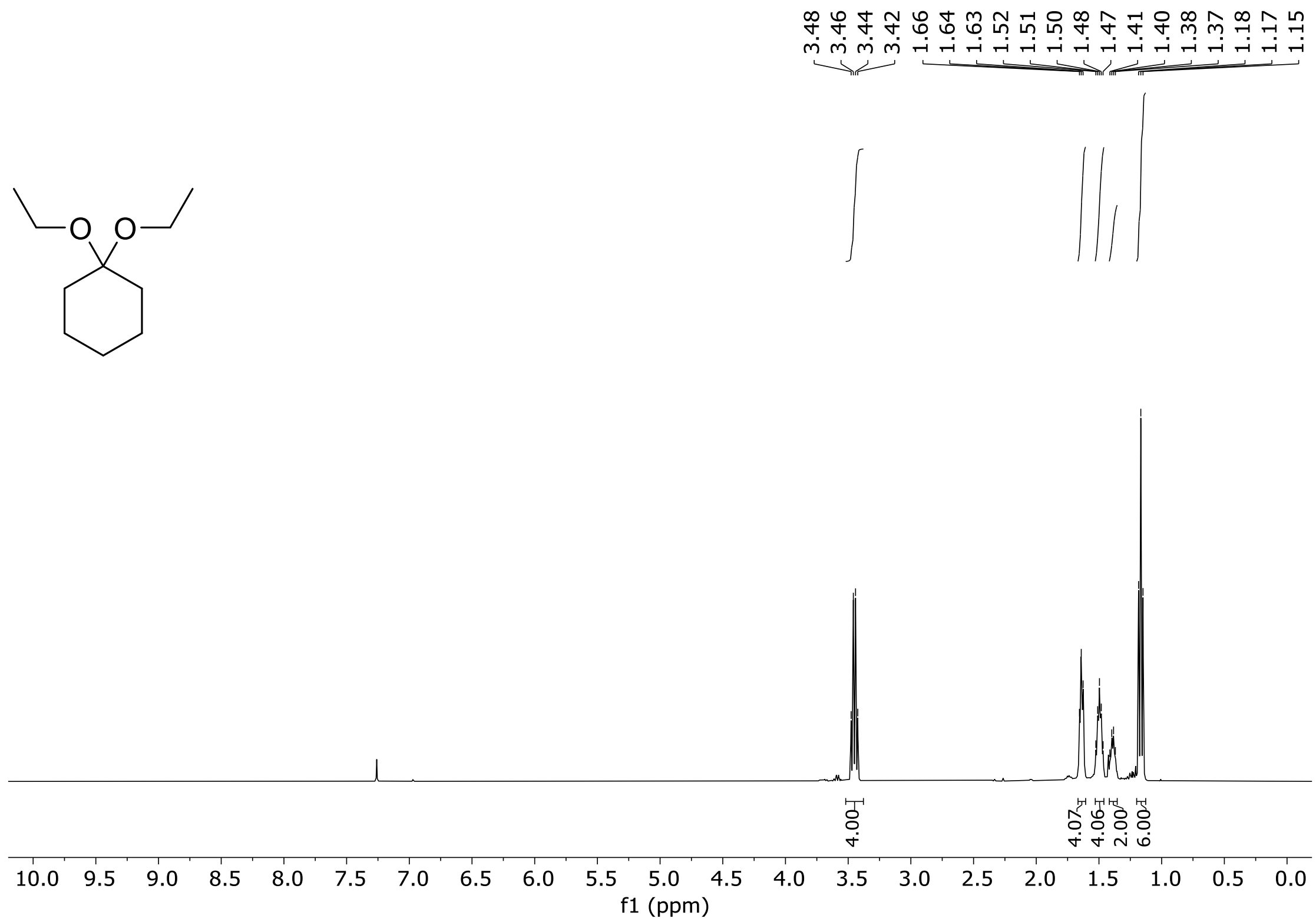

^{1}H-NMR of compound **8b** ($CDCl_3$, 400 MHz).

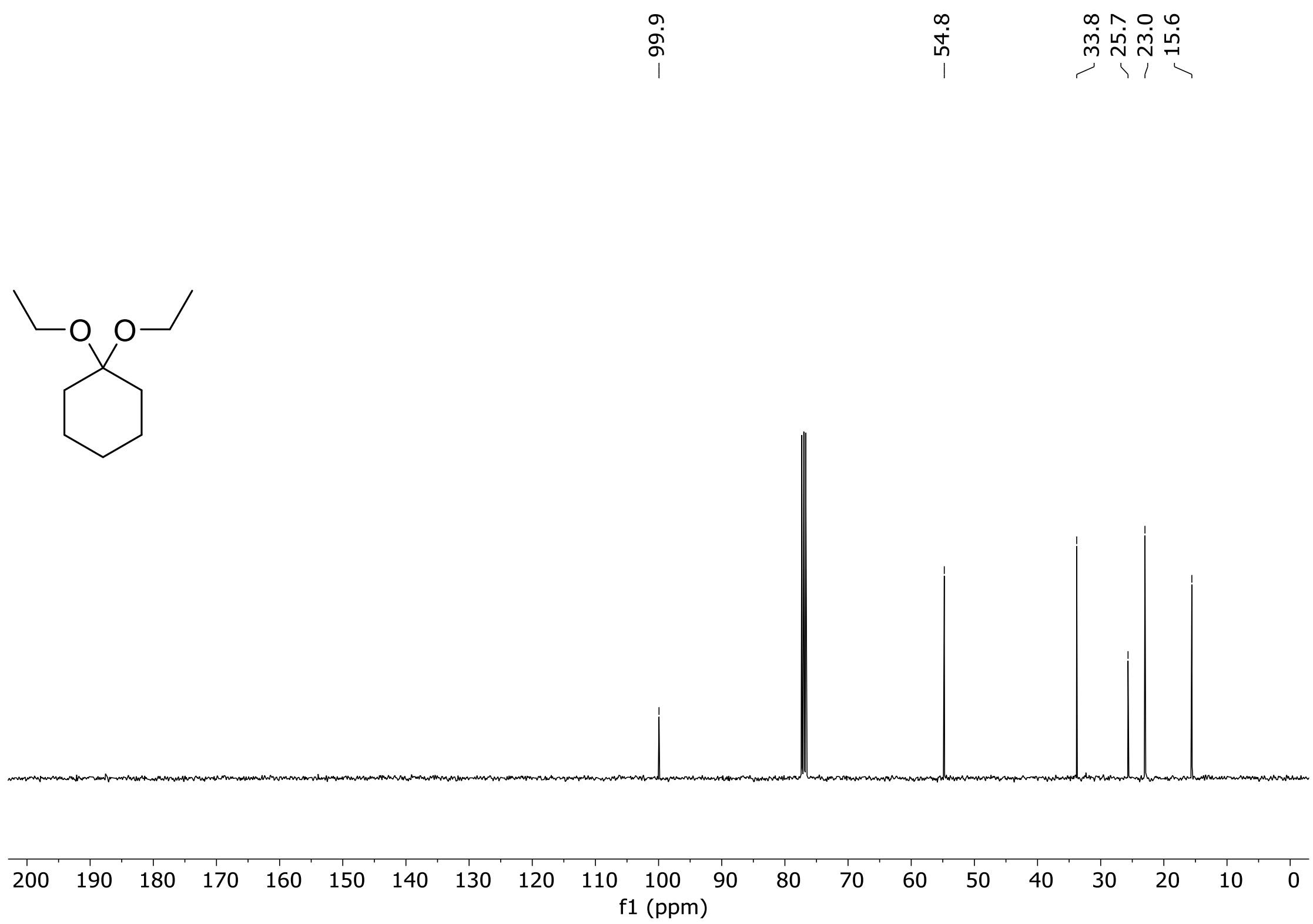

^{13}C-NMR of compound **8b** ($CDCl_3$, 100 MHz).

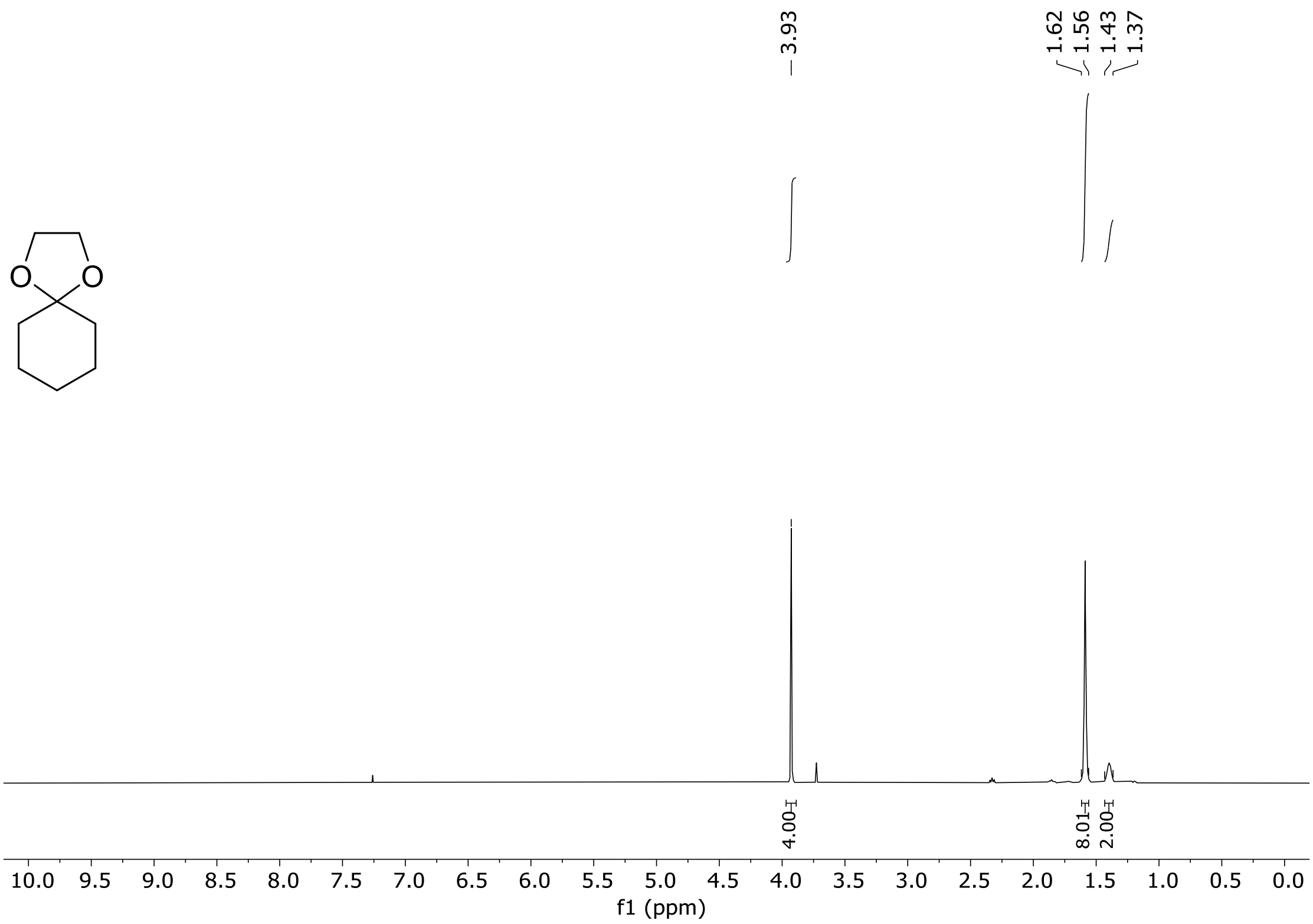

^{1}H-NMR of compound **8c** ($CDCl_3$, 400 MHz).

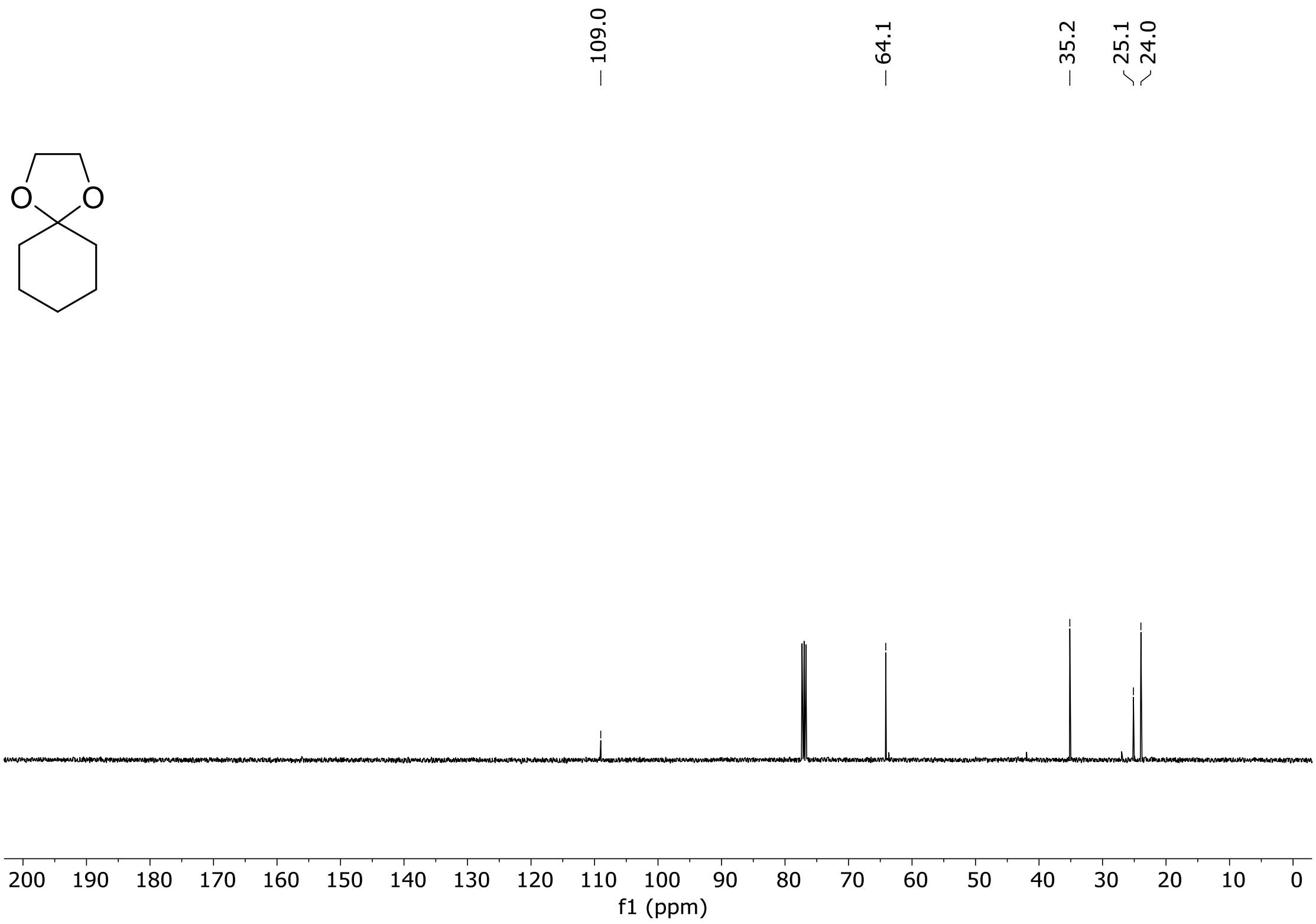

^{13}C-NMR of compound **8c** ($CDCl_3$, 100 MHz).

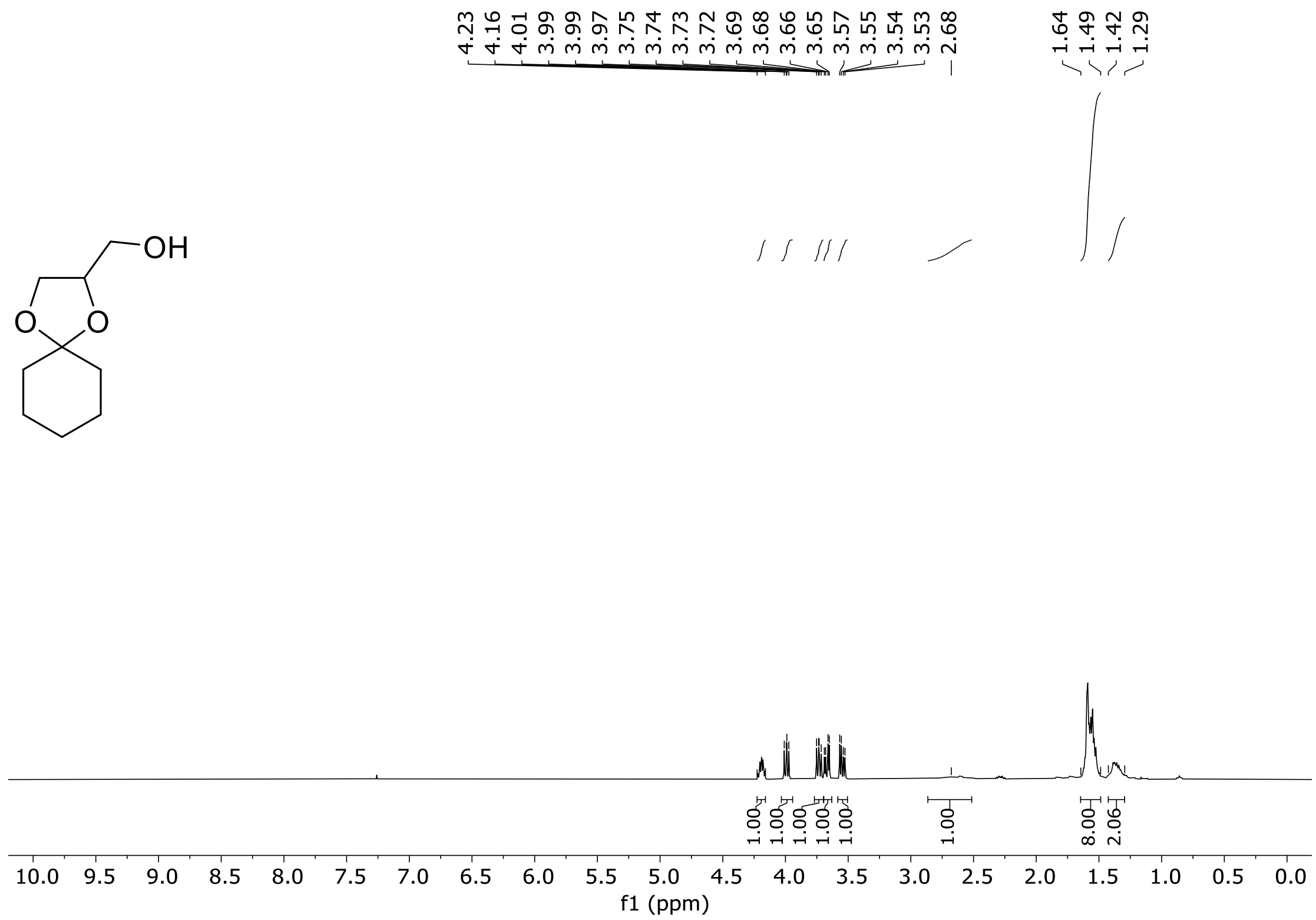

^{1}H-NMR of compound **8d** ($CDCl_3$, 400 MHz).

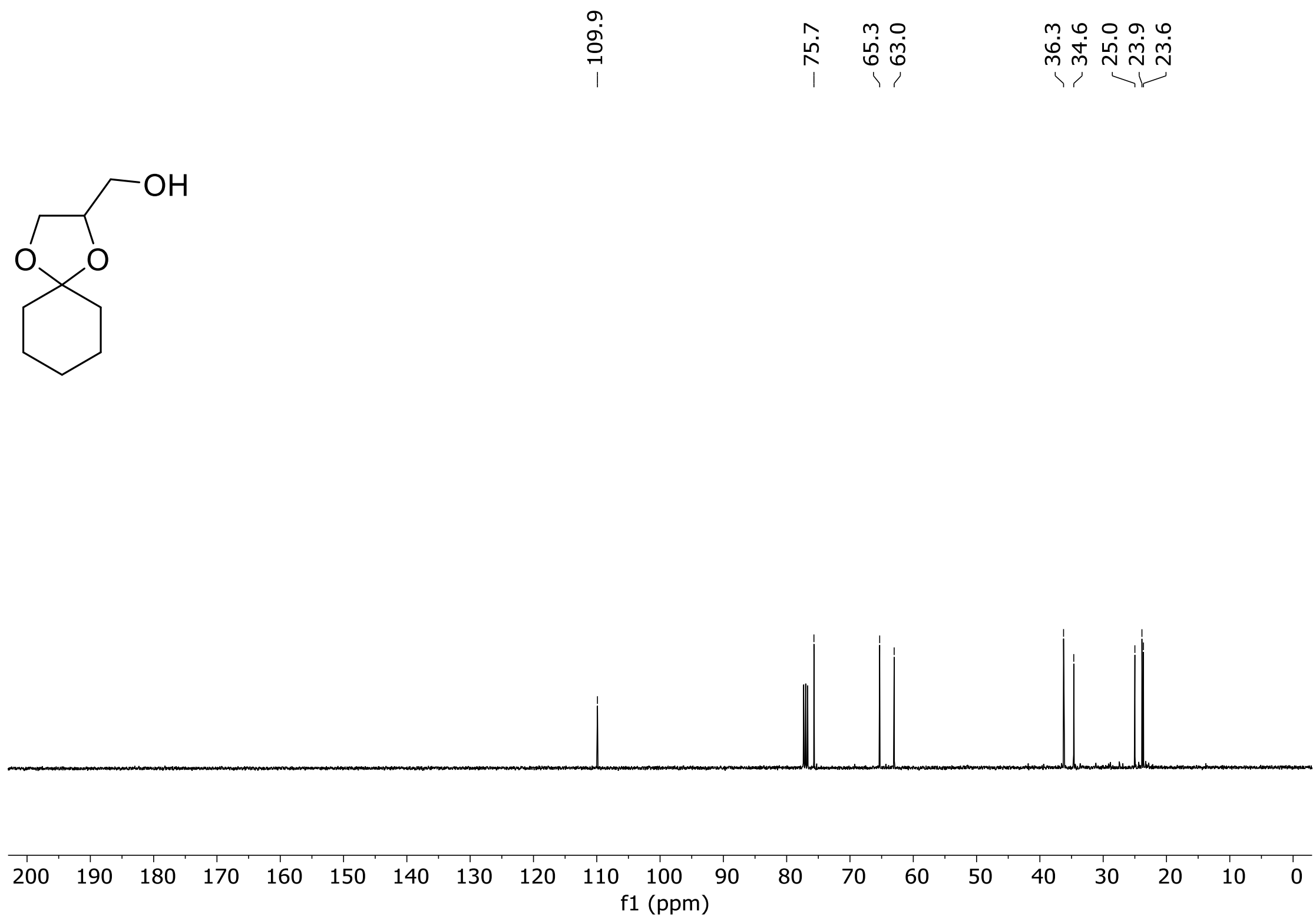

^{13}C-NMR of compound **8d** ($CDCl_3$, 100 MHz).

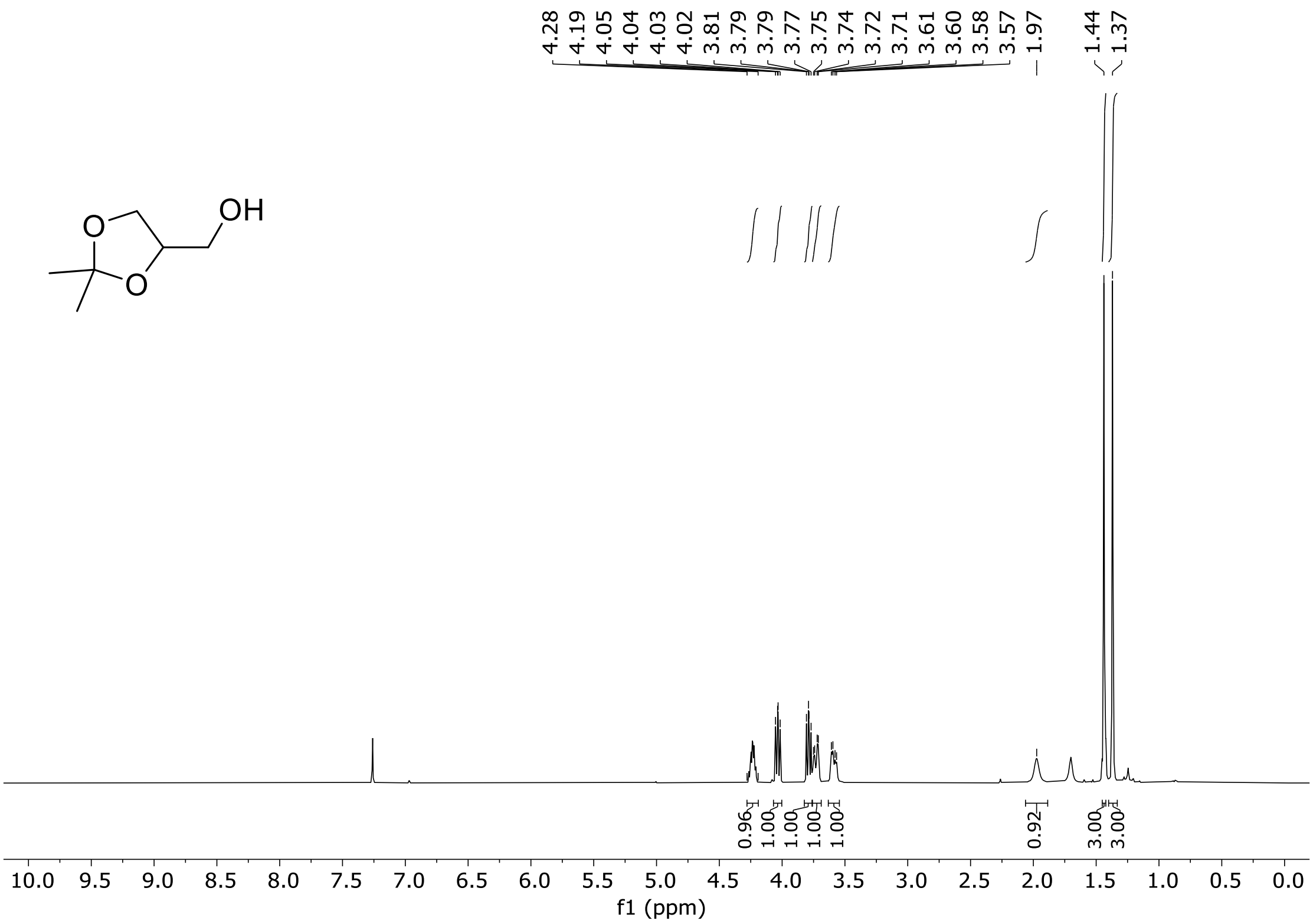

^{1}H-NMR of compound **8e** ($CDCl_3$, 400 MHz).

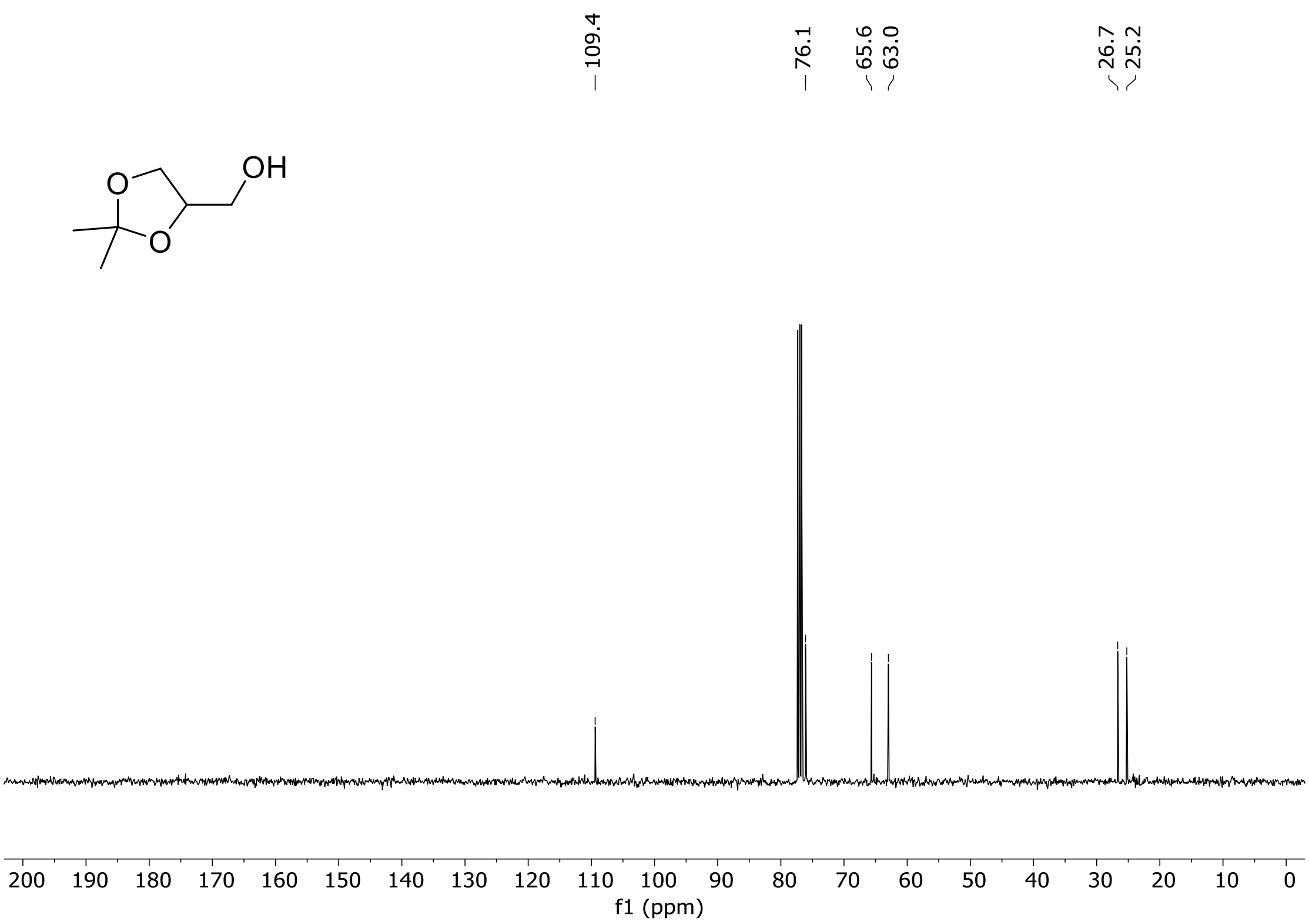

^{13}C-NMR of compound **8e** ($CDCl_3$, 100 MHz).

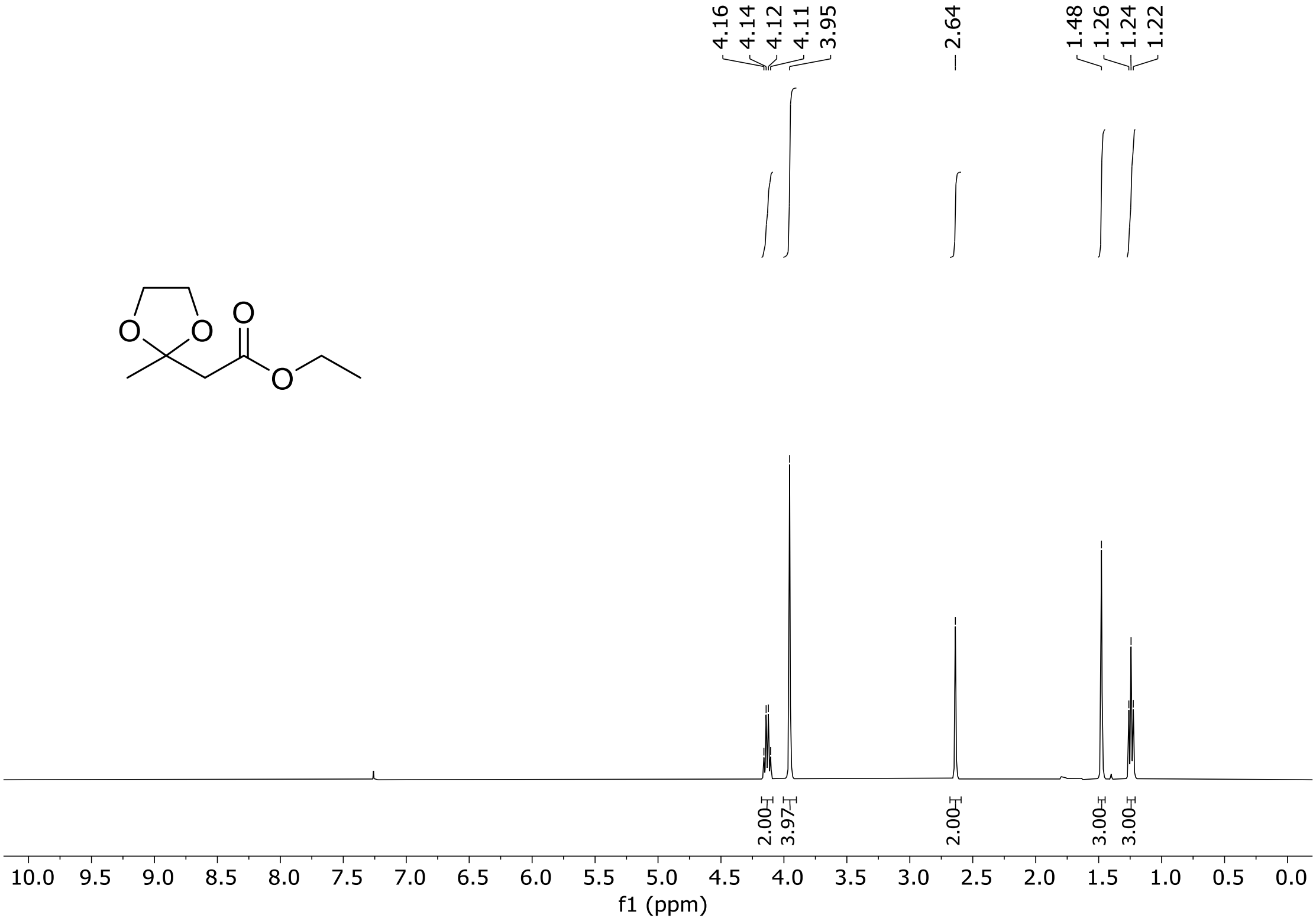

^{1}H-NMR of compound **8f** ($CDCl_3$, 400 MHz).

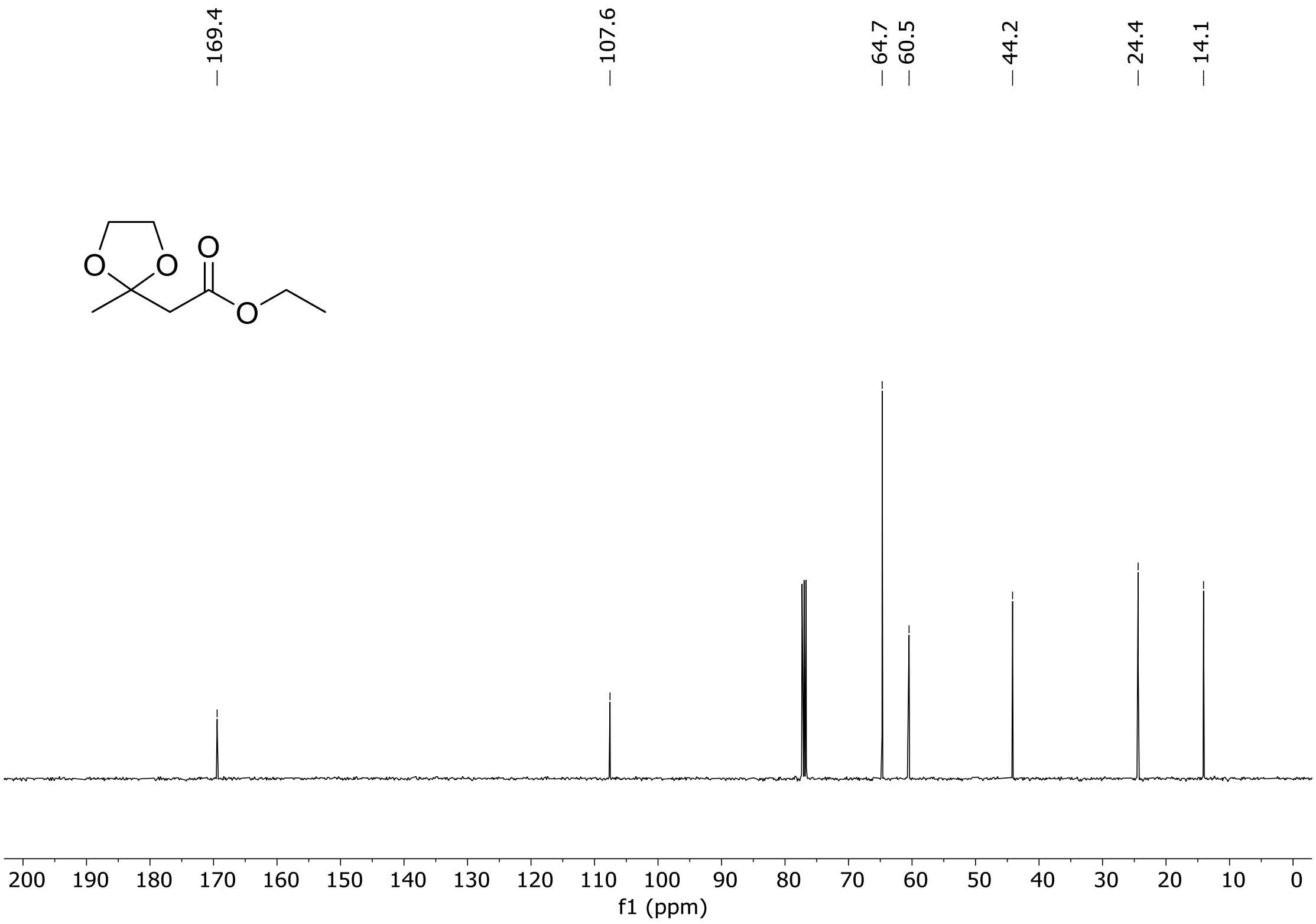

^{13}C-NMR of compound **8f** ($CDCl_3$, 100 MHz).

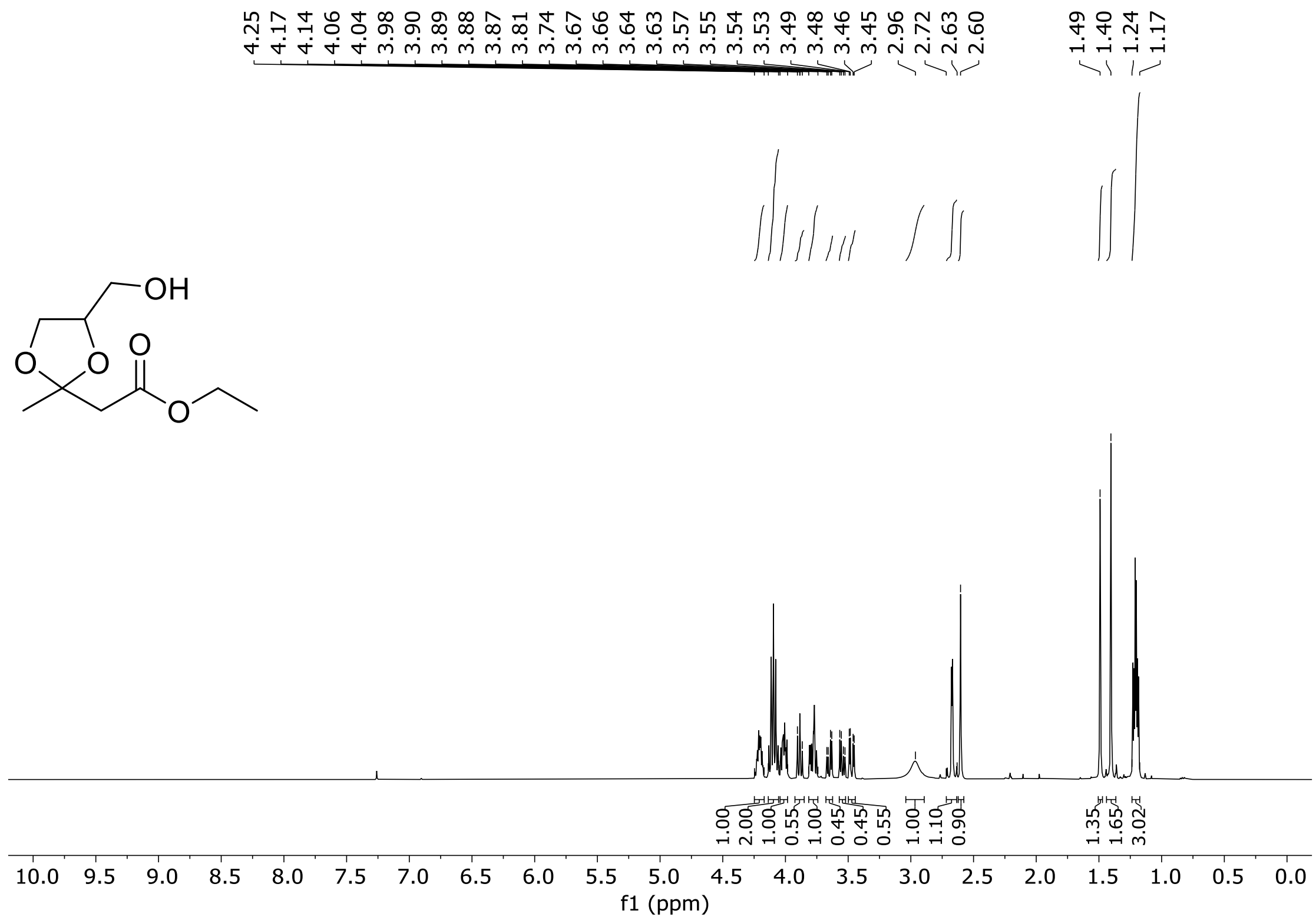

^{1}H-NMR of compound **8g** ($CDCl_3$, 400 MHz).

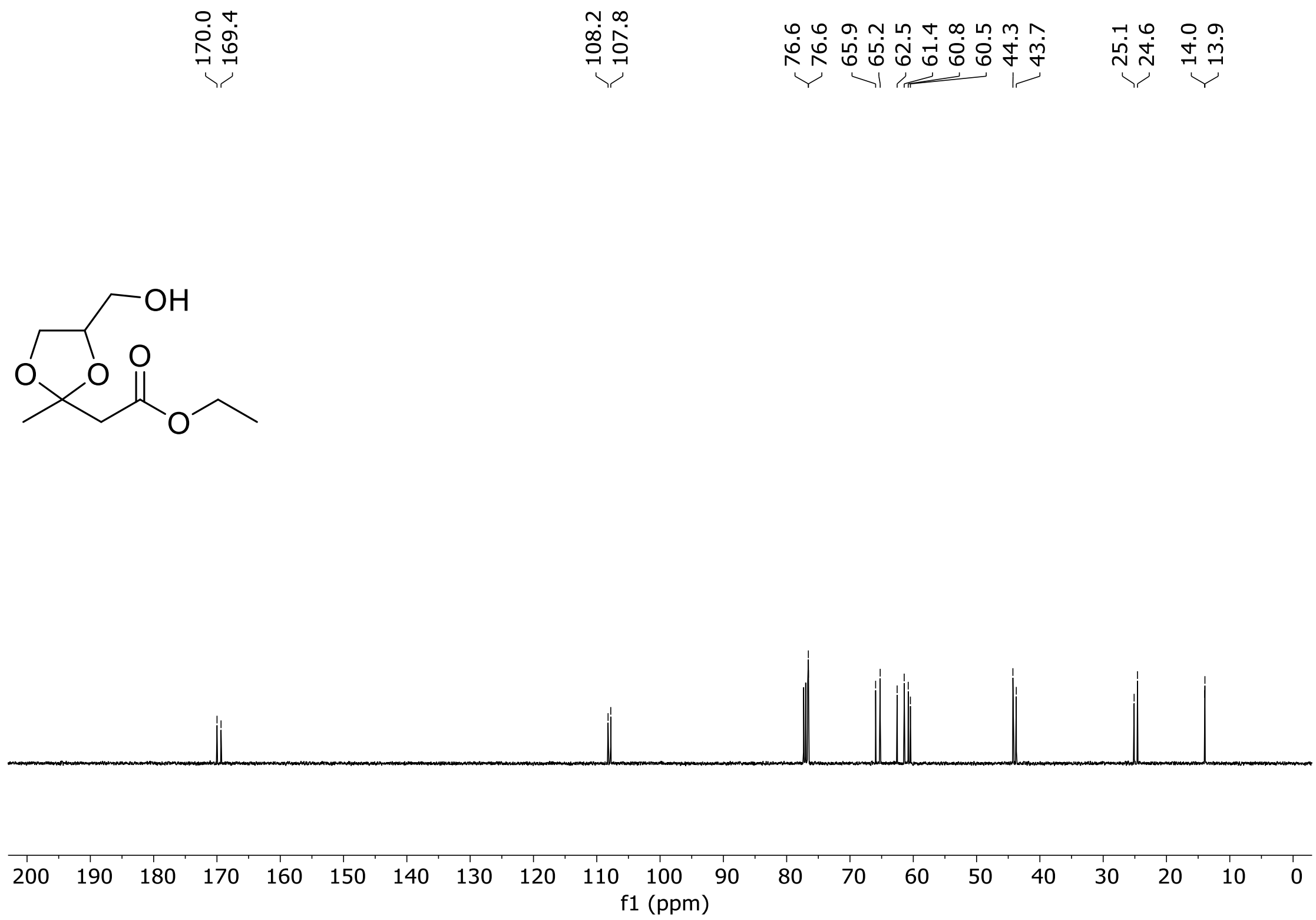

^{13}C-NMR of compound **8g** ($CDCl_3$, 100 MHz).

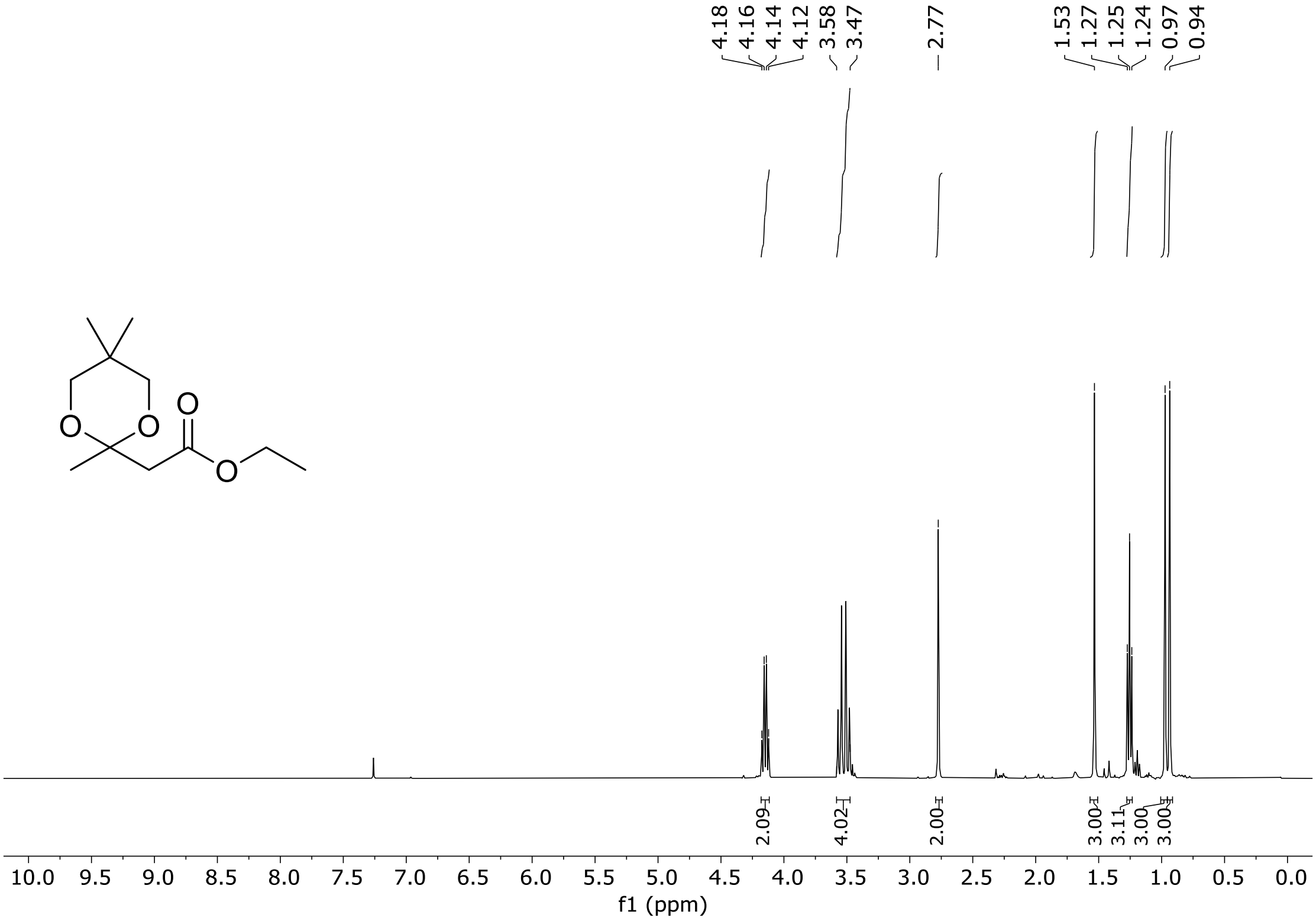

^{1}H-NMR of compound **8h** ($CDCl_3$, 400 MHz).

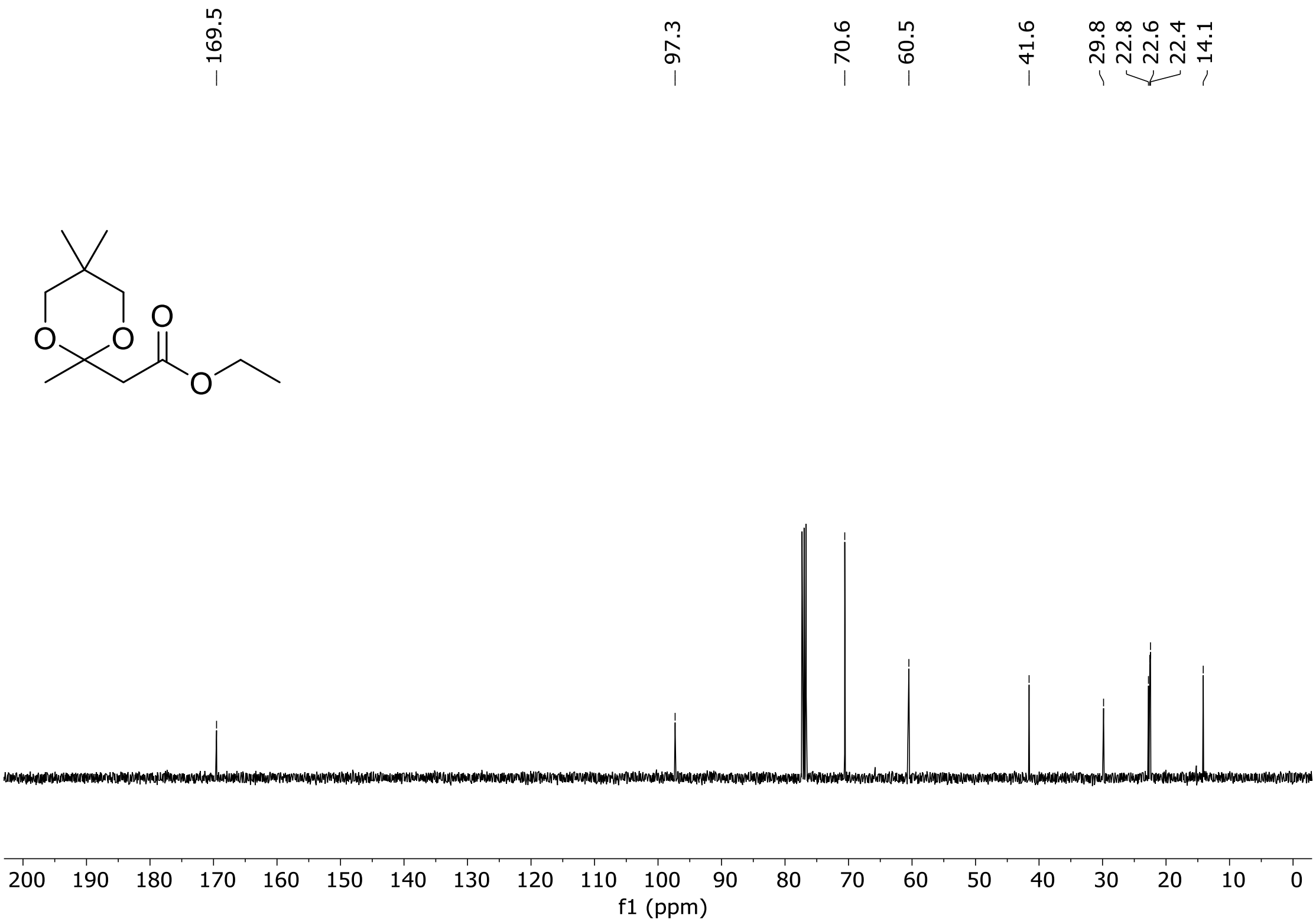

^{13}C-NMR of compound **8h** ($CDCl_3$, 100 MHz).

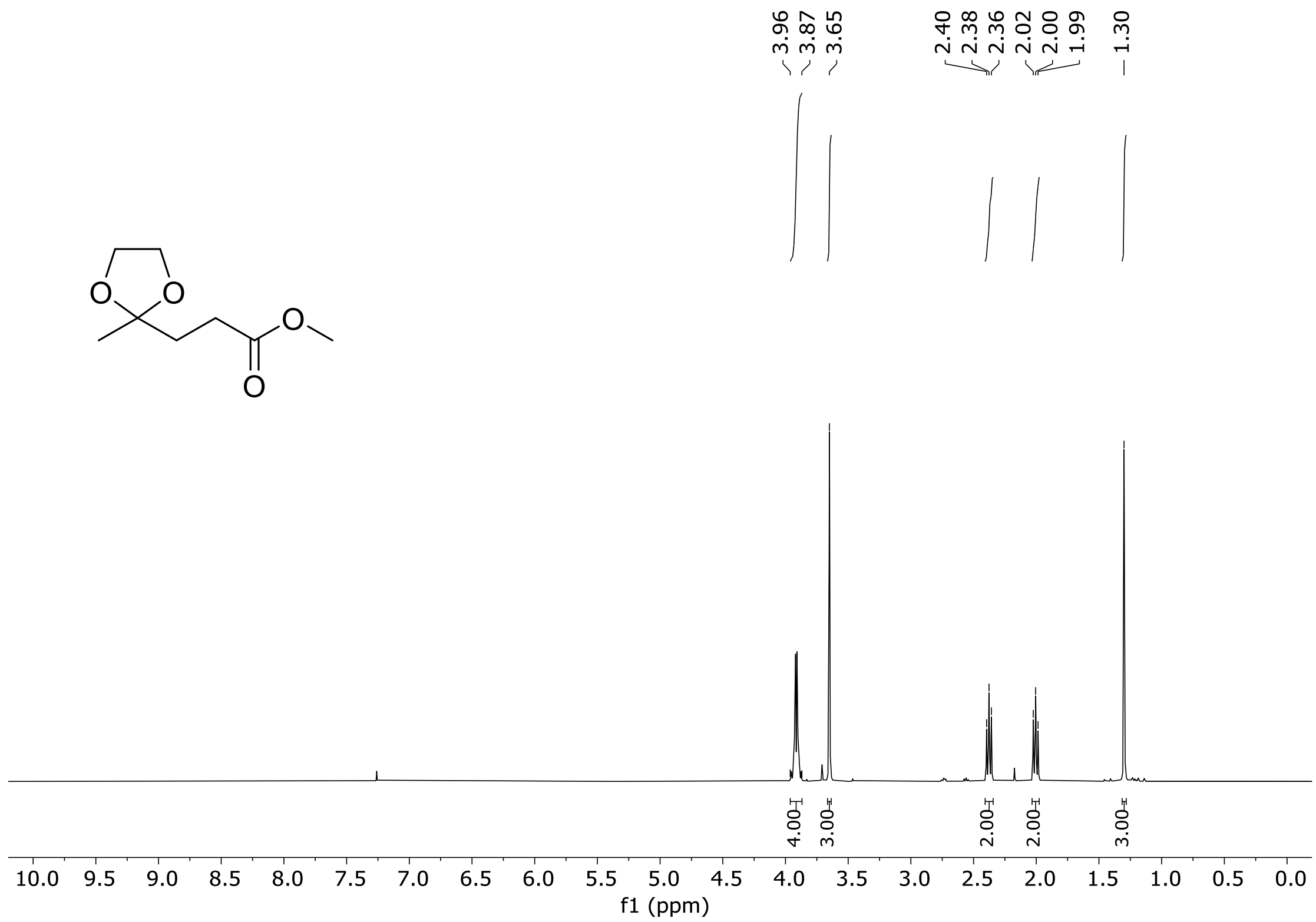

^{1}H-NMR of compound **8i** ($CDCl_3$, 400 MHz).

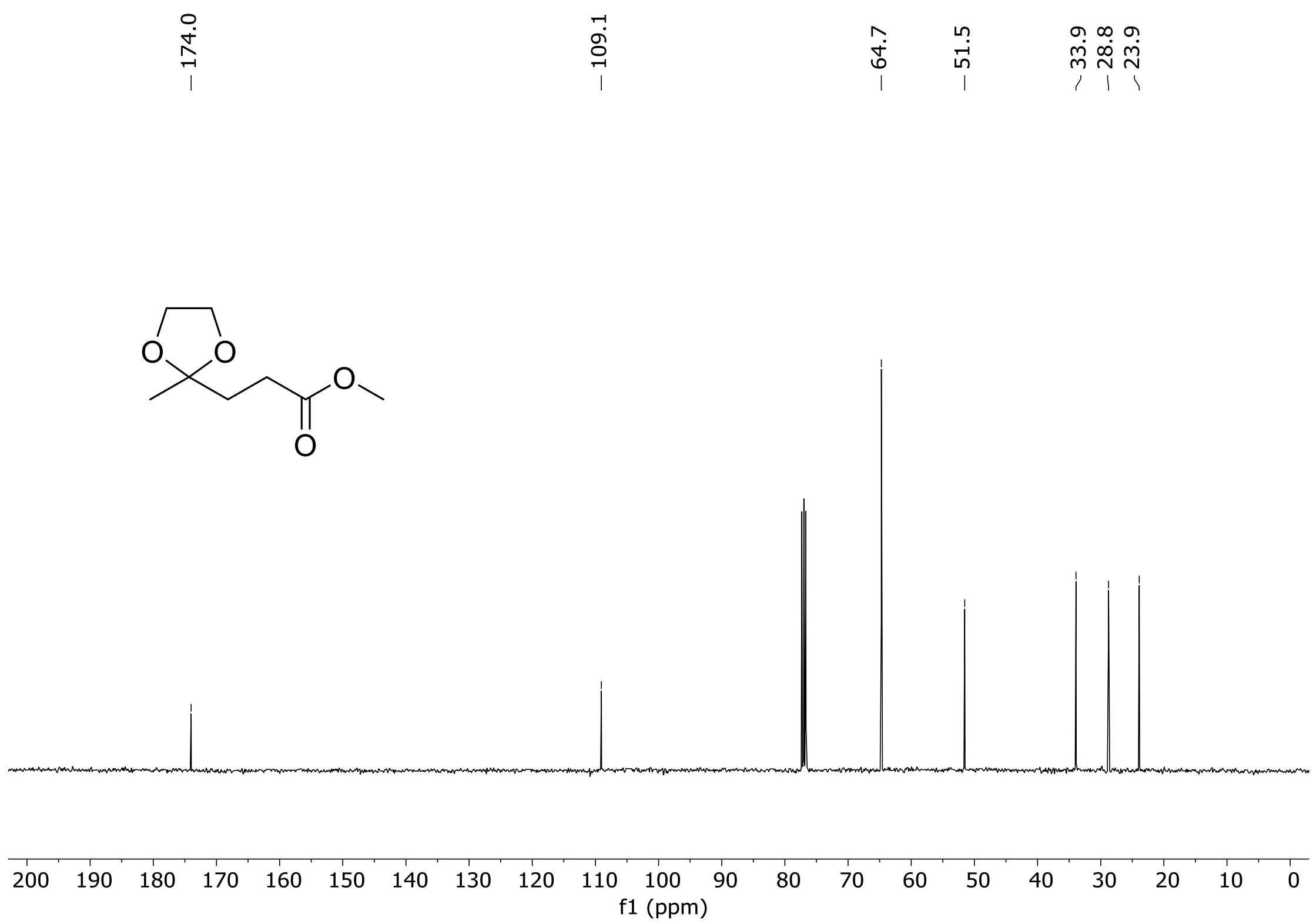

^{13}C-NMR of compound **8i** ($CDCl_3$, 100 MHz).

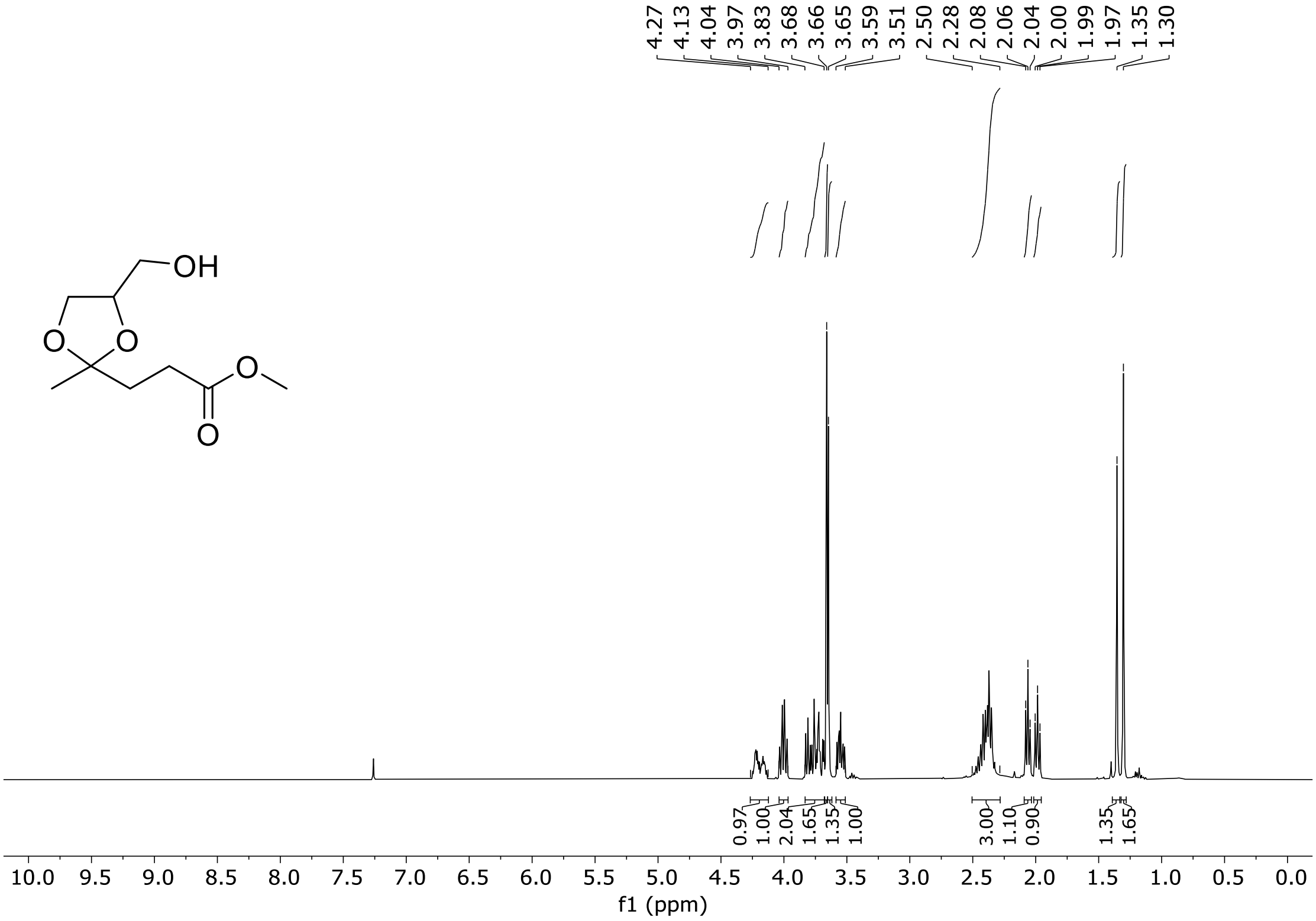

^{1}H-NMR of compound **8j** ($CDCl_3$, 400 MHz).

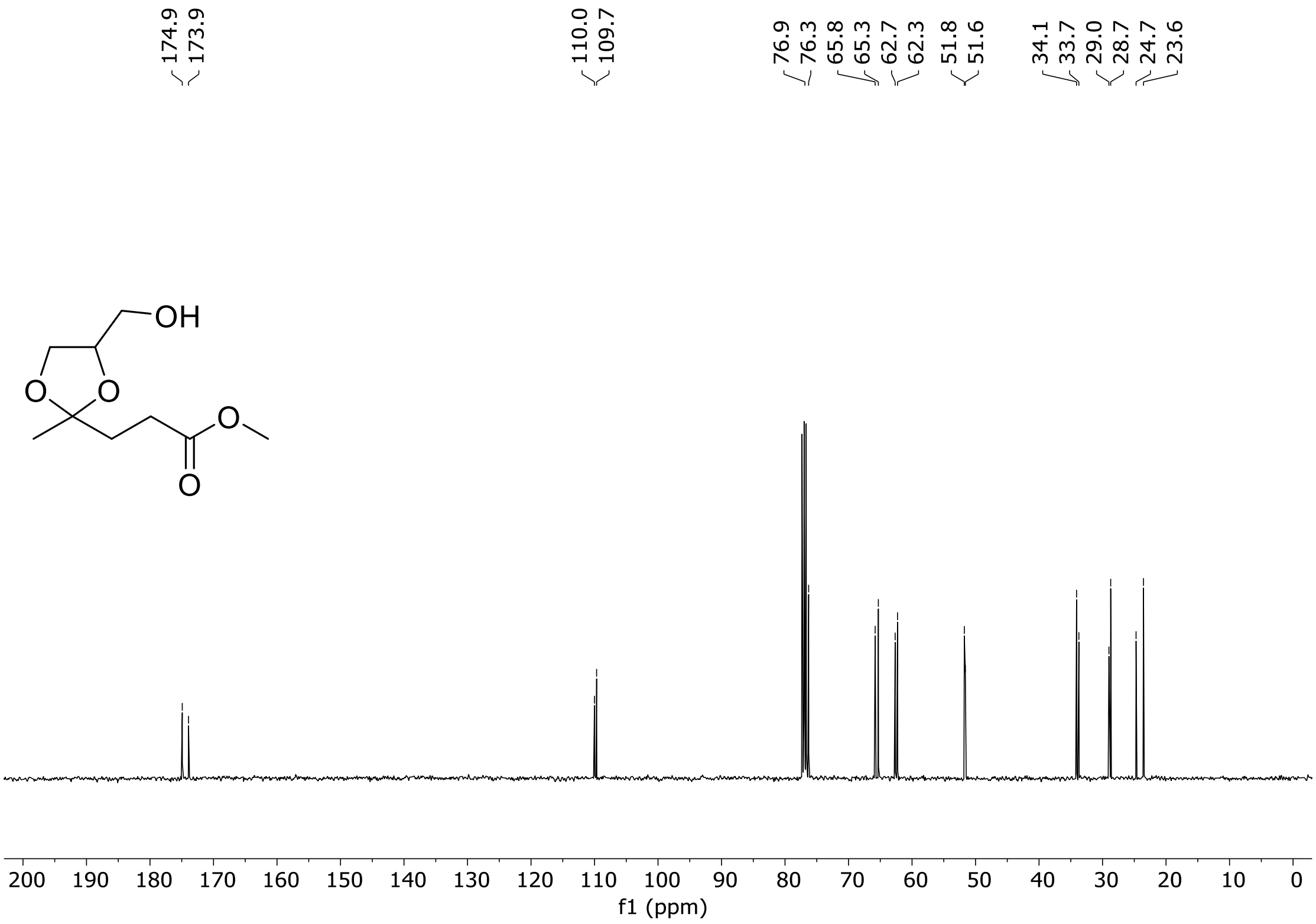

^{13}C-NMR of compound **8j** ($CDCl_3$, 100 MHz).

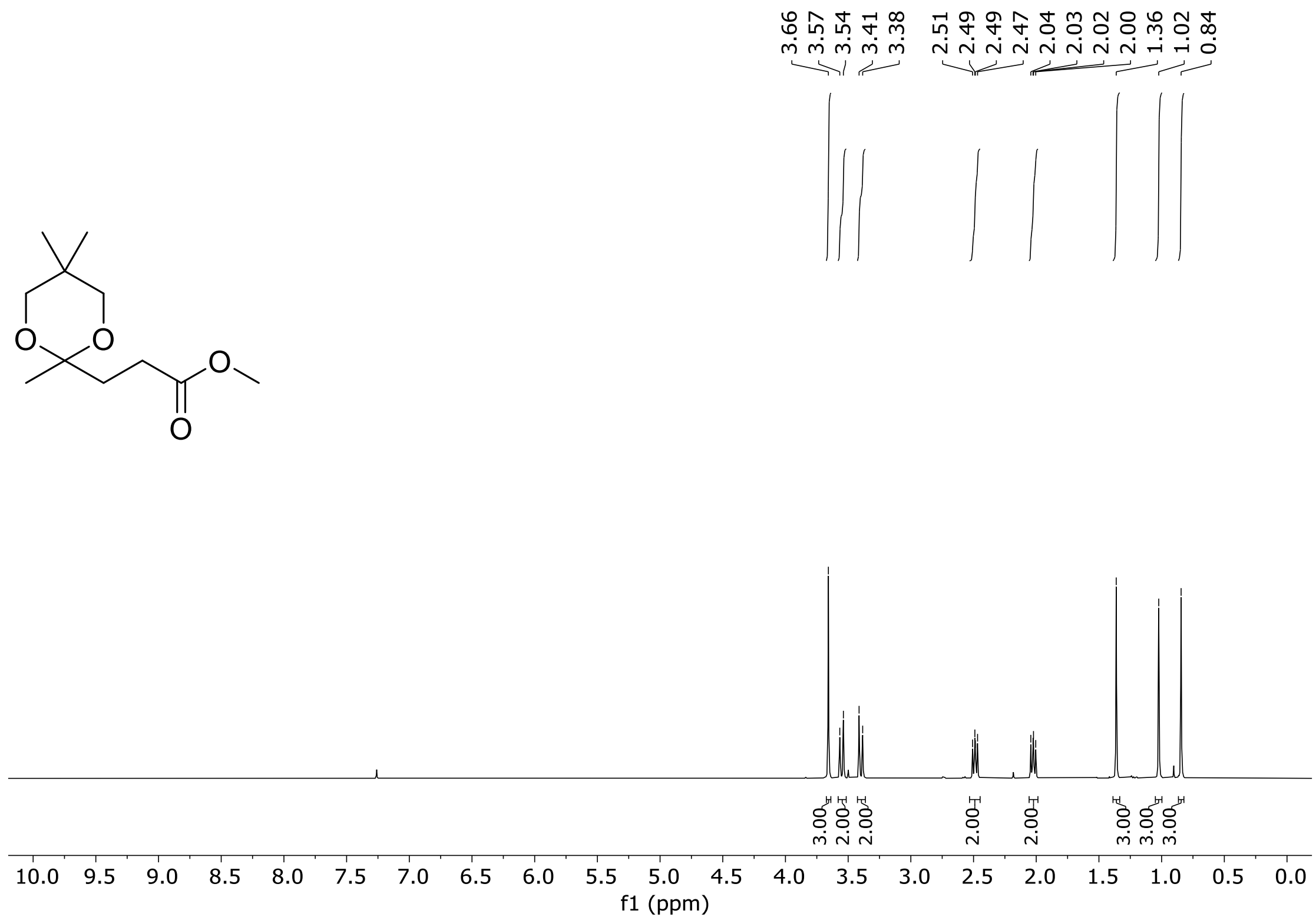

^{1}H-NMR of compound **8k** ($CDCl_3$, 400 MHz).

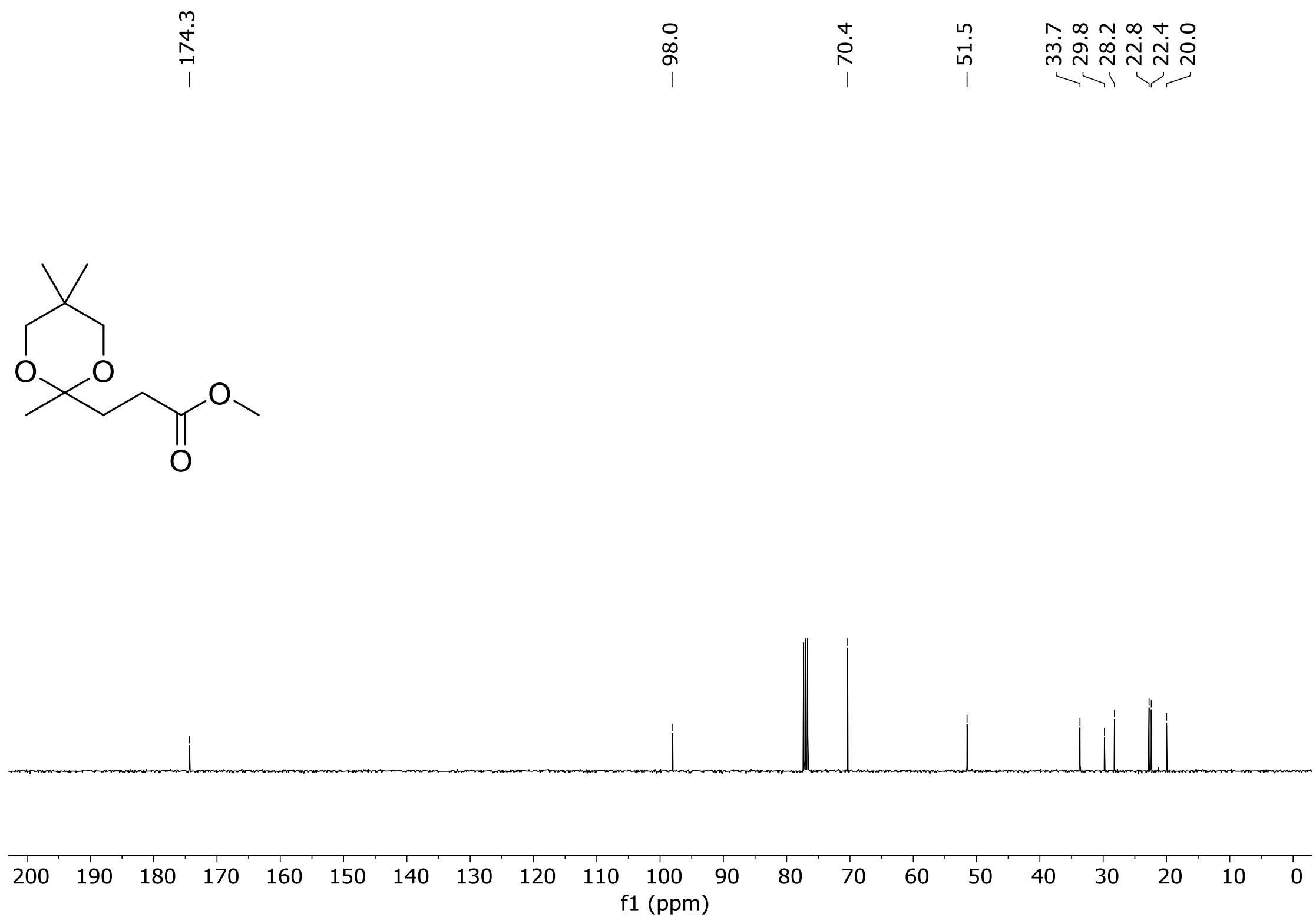

^{13}C-NMR of compound **8k** ($CDCl_3$, 100 MHz).

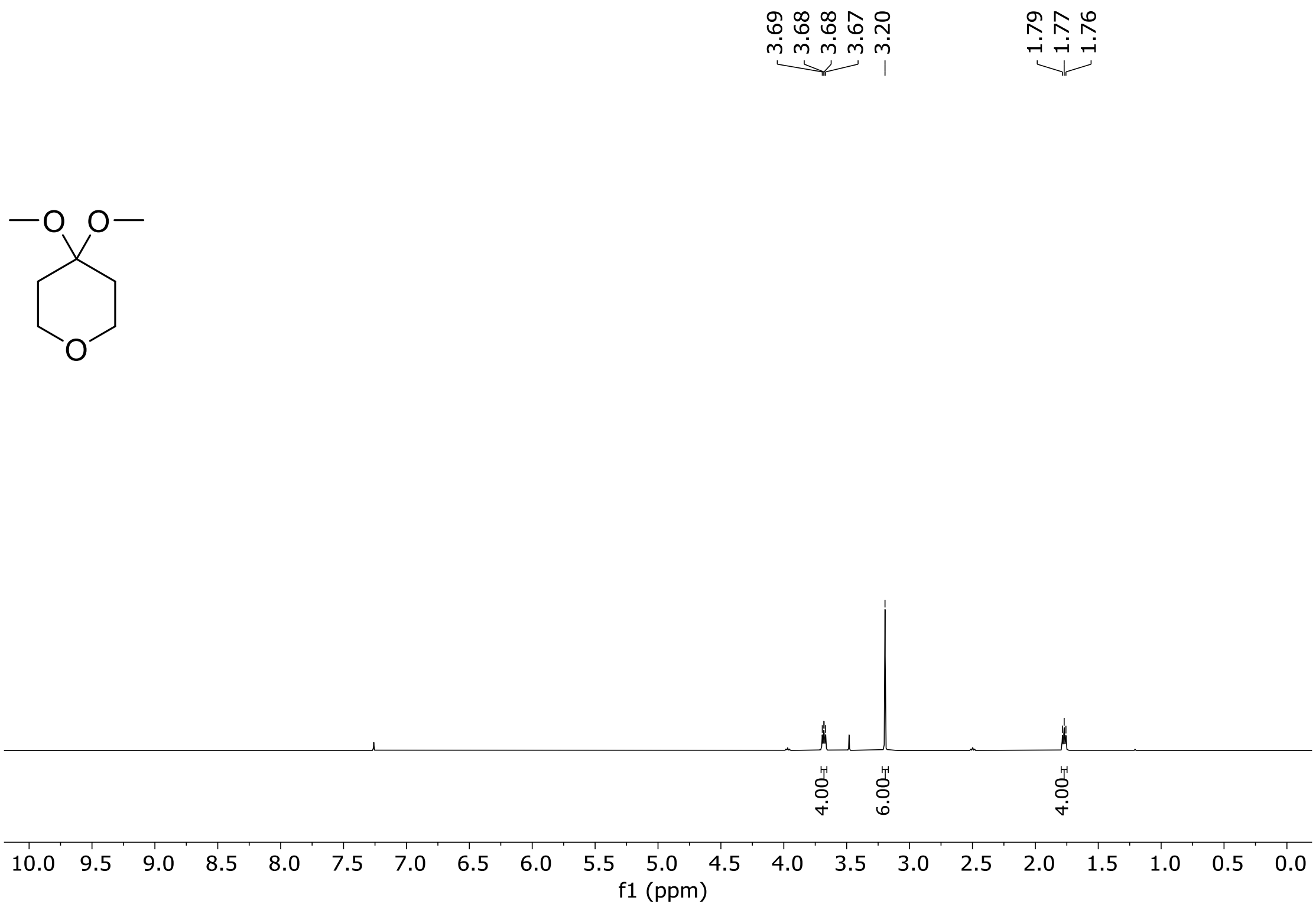

^{1}H-NMR of compound **8l** ($CDCl_3$, 400 MHz).

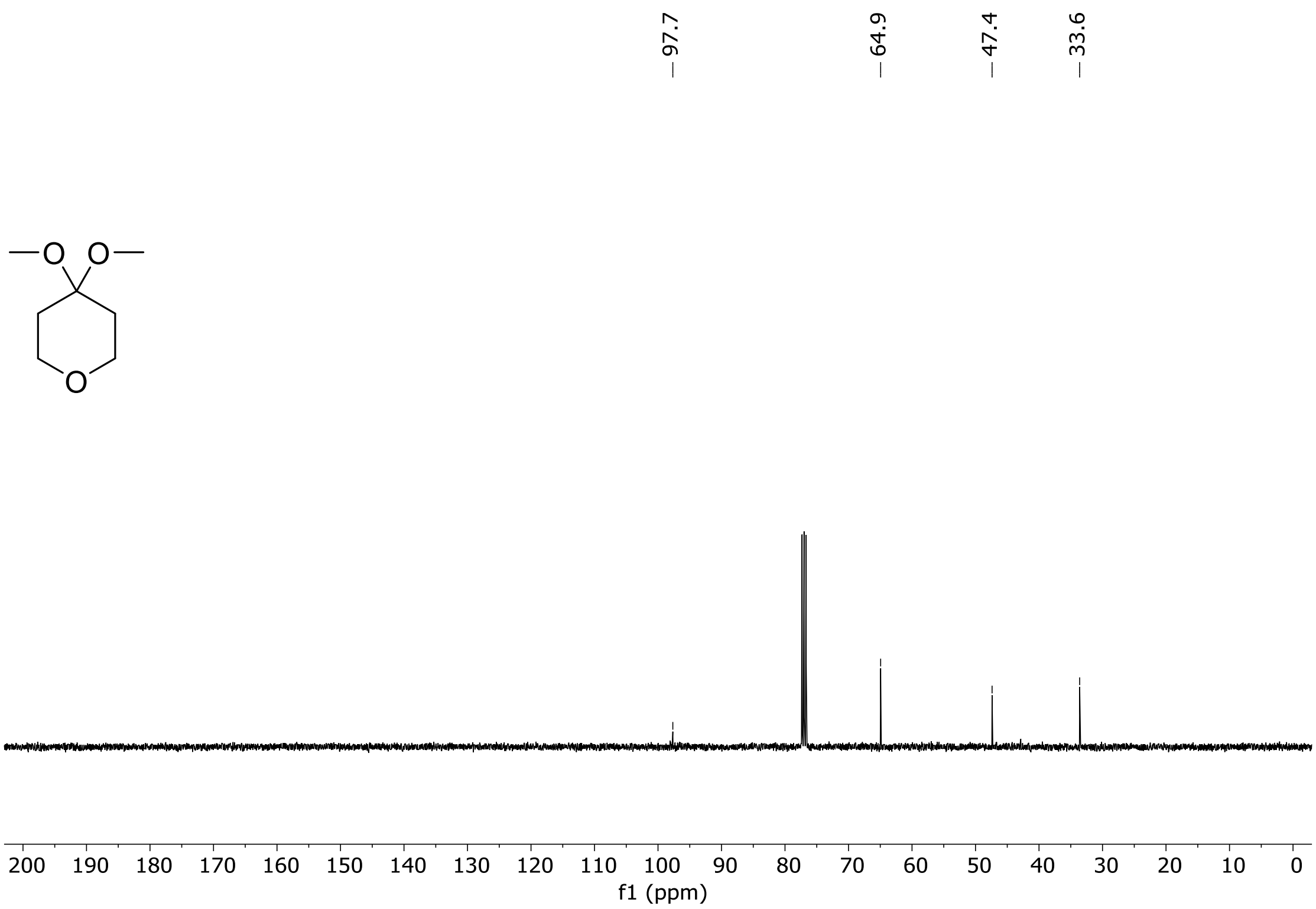

^{13}C-NMR of compound **8l** ($CDCl_3$, 100 MHz).

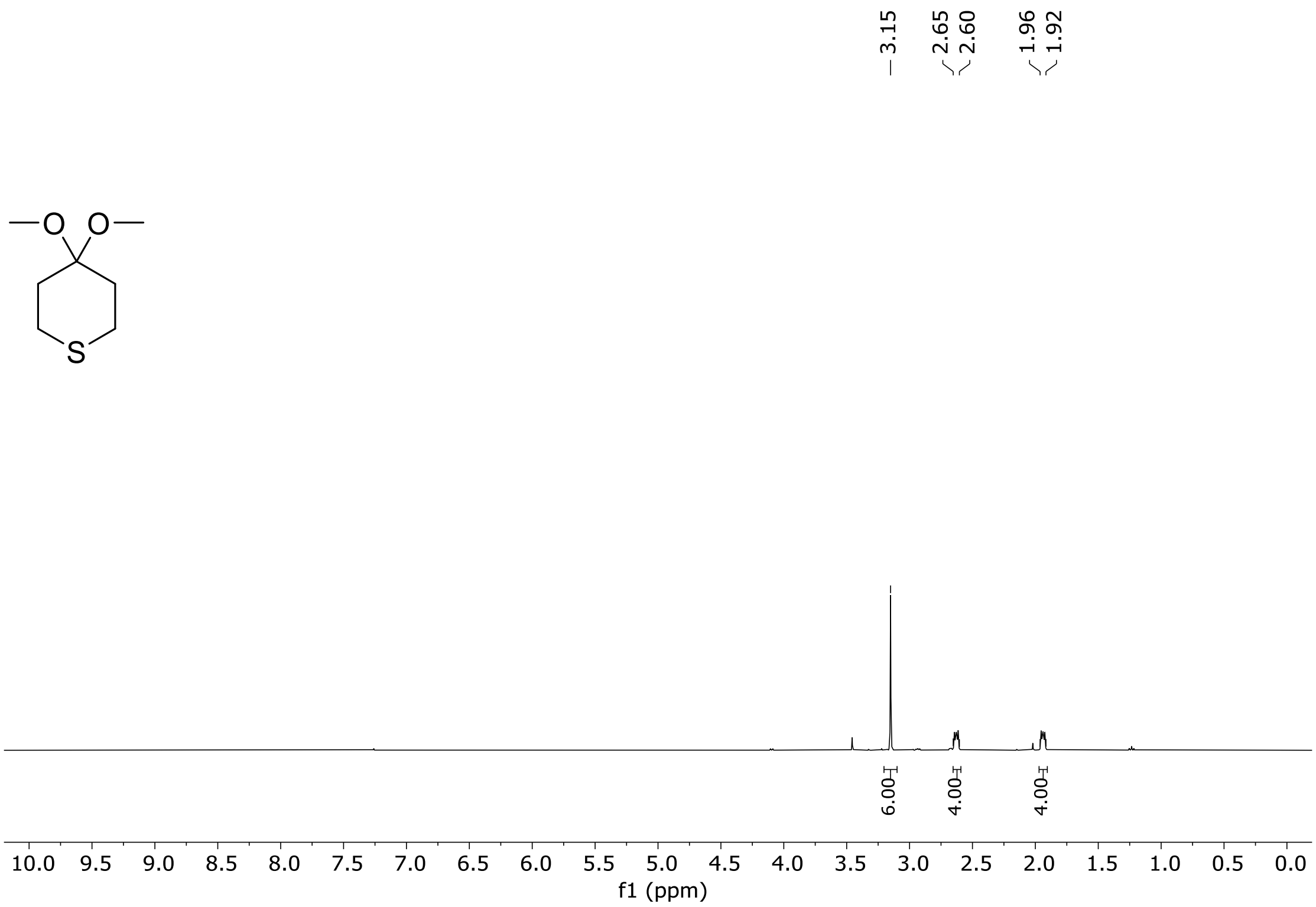

^{1}H-NMR of compound **8m** ($CDCl_3$, 400 MHz).

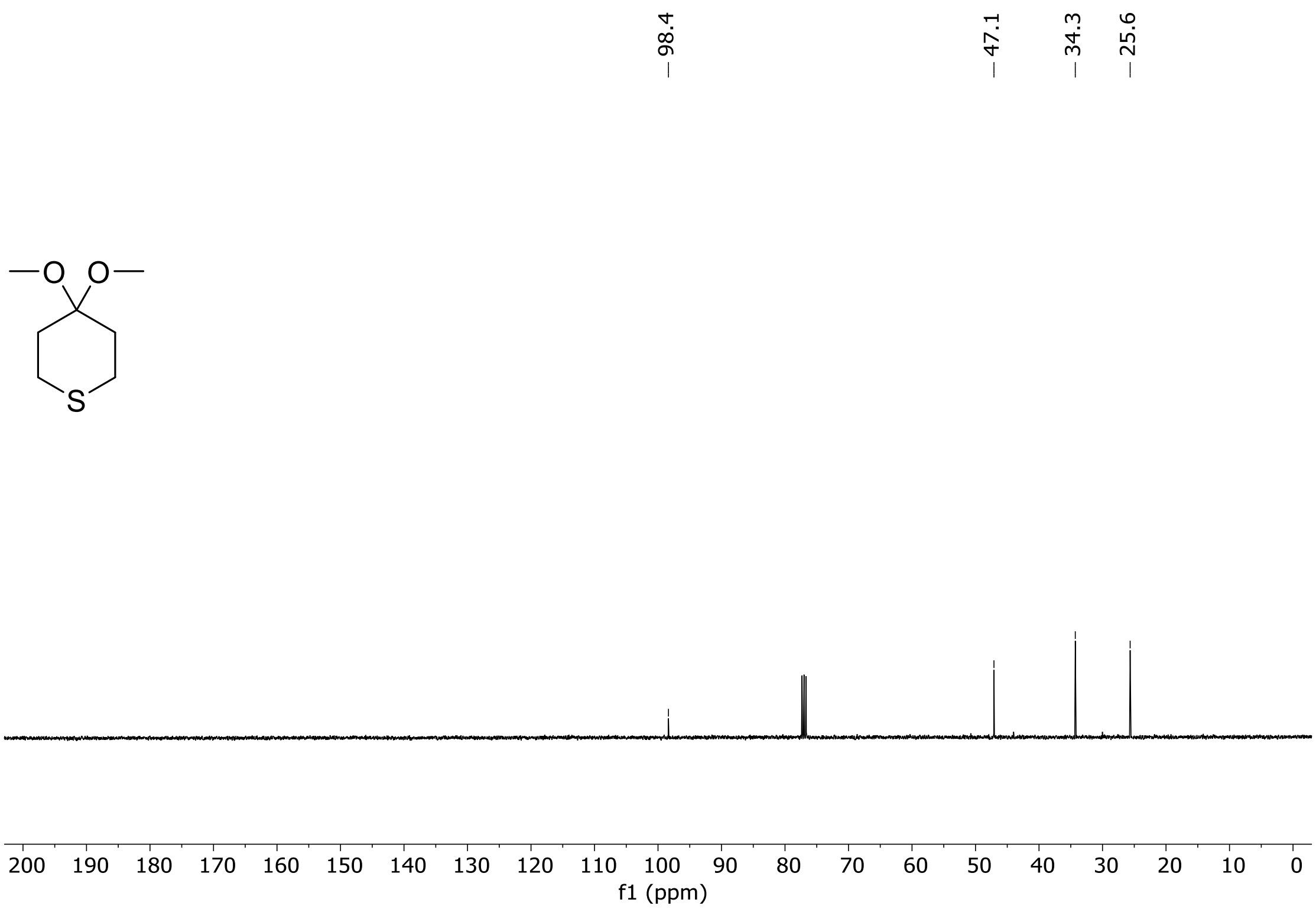

^{13}C-NMR of compound **8m** ($CDCl_3$, 100 MHz).
